# Supplementary material for: Use of drugs for hyperlipidaemia and diabetes and risk of primary and secondary brain tumours: nested case–control studies using the UK Clinical Practice Research Datalink (CPRD)
Source: BMJ Open. 2024 Feb 9;14(2):e072026. doi: 10.1136/bmjopen-2023-072026 (PMC10860117; doi:10.1136/bmjopen-2023-072026)
Supplement: Supplementary data [file bmjopen-2023-072026supp001.pdf]

Read Code	Description	CPRD Medical Code	CPRD Description
B506.00	Malignant neoplasm of choroid	15991	Malignant neoplasm of choroid
B51..00	Malignant neoplasm of brain	18617	Malignant neoplasm of brain
B51..11	Cerebral tumour - malignant	10851	Cerebral tumour - malignant
B510.00	Malignant neoplasm cerebrum (excluding lobes and ventricles)	15711	Malignant neoplasm cerebrum (excluding lobes and ventricles)
B510000	Malignant neoplasm of basal ganglia	48073	Malignant neoplasm of basal ganglia
B510100	Malignant neoplasm of cerebral cortex	61399	Malignant neoplasm of cerebral cortex
B510300		99913	Malignant neoplasm of globus pallidus
B510400	Malignant neoplasm of hypothalamus	70942	Malignant neoplasm of hypothalamus
B510500	Malignant neoplasm of thalamus	62126	Malignant neoplasm of thalamus
B510z00	Malignant neoplasm of cerebrum NOS	54133	Malignant neoplasm of cerebrum NOS
B511.00	Malignant neoplasm of frontal lobe	42426	Malignant neoplasm of frontal lobe
B512.00	Malignant neoplasm of temporal lobe	46792	Malignant neoplasm of temporal lobe
B512000	Malignant neoplasm of hippocampus	67236	Malignant neoplasm of hippocampus
B512z00	Malignant neoplasm of temporal lobe NOS	47556	Malignant neoplasm of temporal lobe NOS
B513.00	Malignant neoplasm of parietal lobe	19226	Malignant neoplasm of parietal lobe
B514.00	Malignant neoplasm of occipital lobe	39088	Malignant neoplasm of occipital lobe
B515.00	Malignant neoplasm of cerebral ventricles	52511	Malignant neoplasm of cerebral ventricles
B515000	Malignant neoplasm of choroid plexus	46789	Malignant neoplasm of choroid plexus
B516.00	Malignant neoplasm of cerebellum	45154	Malignant neoplasm of cerebellum
B517.00	Malignant neoplasm of brain stem	44089	Malignant neoplasm of brain stem
B517000	Malignant neoplasm of cerebral peduncle	64557	Malignant neoplasm of cerebral peduncle
B517100	Malignant neoplasm of medulla oblongata	49132	Malignant neoplasm of medulla oblongata
B517200	Malignant neoplasm of midbrain	93537	Malignant neoplasm of midbrain
B517300	Malignant neoplasm of pons	91240	Malignant neoplasm of pons
B517z00	Malignant neoplasm of brain stem NOS	68641	Malignant neoplasm of brain stem NOS
B51y.00	Malignant neoplasm of other parts of brain	71139	Malignant neoplasm of other parts of brain
B51y000	Malignant neoplasm of corpus callosum	59170	Malignant neoplasm of corpus callosum
B51y200	Malignant neoplasm, overlapping lesion of brain	65241	Malignant neoplasm, overlapping lesion of brain
B51yz00		100733	Malignant neoplasm of other part of brain NOS
B51z.00	Malignant neoplasm of brain NOS	41520	Malignant neoplasm of brain NOS
B521.00	Malignant neoplasm of cerebral meninges	28919	Malignant neoplasm of cerebral meninges
B521200		109473	Malignant neoplasm of cerebral pia mater
B521z00	Malignant neoplasm of cerebral meninges NOS	70104	Malignant neoplasm of cerebral meninges NOS
B52X.00	Malignant neoplasm of meninges, unspecified	49875	Malignant neoplasm of meninges, unspecified

B542.00	Malignant neoplasm pituitary gland and craniopharyngeal duct	59823	Malignant neoplasm pituitary gland and craniopharyngeal duct
B542000	Malignant neoplasm of pituitary gland	8550	Malignant neoplasm of pituitary gland
B542z00	Malig neop pituitary gland or craniopharyngeal duct NOS	59718	Malig neop pituitary gland or craniopharyngeal duct NOS
B543.00	Malignant neoplasm of pineal gland	42460	Malignant neoplasm of pineal gland
B583.00	Secondary malignant neoplasm of brain and spinal cord	33843	Secondary malignant neoplasm of brain and spinal cord
B583000	Secondary malignant neoplasm of brain	5198	Secondary malignant neoplasm of brain
B583200	Cerebral metastasis	5199	Cerebral metastasis
B583z00	Secondary malignant neoplasm of brain or spinal cord NOS	59375	Secondary malignant neoplasm of brain or spinal cord NOS
B8yy300	Carcinoma in situ of pituitary gland	45909	Carcinoma in situ of pituitary gland
BA06.00	Neoplasm of unspecified nature of brain	1044	Neoplasm of unspecified nature of brain
BB9G.00	[M]Infiltrating ductular carcinoma	7319	[M]Infiltrating ductular carcinoma
BBa3.00	[M]Pineoblastoma	50151	[M]Pineoblastoma
BBb..00	[M]Gliomas	12309	[M]Gliomas
BBb0.00	[M]Glioma, malignant	31574	[M]Glioma, malignant
BBb0.11	[M]Glioma NOS	8523	[M]Glioma NOS
BBb0.12	[M]Gliosarcoma	34252	[M]Gliosarcoma
BBb1.00	[M]Gliomatosis cerebri	38551	[M]Gliomatosis cerebri
BBb2.00	[M]Mixed glioma	68808	[M]Mixed glioma
BBb2.11	[M]Mixed glioma	39386	[M]Mixed glioma
BBb3.00	[M]Subependymal glioma	94267	[M]Subependymal glioma
BBb3.11	[M]Subependymal astrocytoma NOS	90487	[M]Subependymal astrocytoma NOS
BBb3.12	[M]Subependymal astrocytoma NOS	28344	[M]Subependymal astrocytoma NOS
BBb4.00	[M]Subependymal giant cell astrocytoma	49168	[M]Subependymal giant cell astrocytoma
BBb8.00	[M]Ependymoma, anaplastic type	52751	[M]Ependymoma, anaplastic type
BBb8.11	[M]Ependymoblastoma	46769	[M]Ependymoblastoma
BBbA.00	[M]Myxopapillary ependymoma	43114	[M]Myxopapillary ependymoma
BBba.00	[M]Primitive neuroectodermal tumour	41695	[M]Primitive neuroectodermal tumour
BBbB.00	[M]Astrocytoma NOS	8547	[M]Astrocytoma NOS
BBbB.11	[M]Astrocytic glioma	27748	[M]Astrocytic glioma
BBbC.00	[M]Astrocytoma, anaplastic type	8328	[M]Astrocytoma, anaplastic type
BBbE.00	[M]Gemistocytic astrocytoma	45531	[M]Gemistocytic astrocytoma
BBbF.00	[M]Fibrillary astrocytoma	27846	[M]Fibrillary astrocytoma
BBbG.00	[M]Pilocytic astrocytoma	30273	[M]Pilocytic astrocytoma
BBbG.11	[M]Juvenile astrocytoma	61783	[M]Juvenile astrocytoma
BBbG.12	[M]Piloid astrocytoma	98800	[M]Piloid astrocytoma
BBbH.00		103047	[M]Spongioblastoma NOS
BBbK.00	[M]Astroblastoma	50235	[M]Astroblastoma
BBbL.00	[M]Glioblastoma NOS	23083	[M]Glioblastoma NOS
BBbL.11	[M]Glioblastoma multiforme	9575	[M]Glioblastoma multiforme
BBbM.00	[M]Giant cell glioblastoma	66064	[M]Giant cell glioblastoma
BBbQ.00	[M]Oligodendroglioma NOS	27744	[M]Oligodendroglioma NOS
BBbR.00	[M]Oligodendroglioma, anaplastic type	49186	[M]Oligodendroglioma, anaplastic type

BBbS.00	[M]Oligodendroblastoma	46404	[M]Oligodendroblastoma
BBbT.00	[M]Medulloblastoma NOS	34763	[M]Medulloblastoma NOS
BBbU.00	[M]Desmoplastic medulloblastoma	65952	[M]Desmoplastic medulloblastoma
BBbV.00	[M]Medullomyoblastoma	31767	[M]Medullomyoblastoma
BBbW.00	[M]Cerebellar sarcoma NOS	37473	[M]Cerebellar sarcoma NOS
BBbZ.00	[M]Pleomorphic xanthoastrocytoma	67587	[M]Pleomorphic xanthoastrocytoma
BBbz.00	[M]Glioma NOS	27653	[M]Glioma NOS
BBc6.00	[M]Ganglioglioma	31629	[M]Ganglioglioma
BBc7.11	[M]Neuroastrocytoma	68479	[M]Neuroastrocytoma
BBd1.00	[M]Meningiomatosis NOS	98677	[M]Meningiomatosis NOS
BBd1.11	[M]Diffuse meningiomatosis	95108	[M]Diffuse meningiomatosis
BBd2.00	[M]Meningioma, malignant	27363	[M]Meningioma, malignant
BBd2.11	[M]Leptomeningeal sarcoma	60347	[M]Leptomeningeal sarcoma
BBd2.12	[M]Meningothelial sarcoma	96798	[M]Meningothelial sarcoma
BBd5.00	[M]Psammomatous meningioma	38870	[M]Psammomatous meningioma
BBd7.11	[M]Angioblastic meningioma	46490	[M]Angioblastic meningioma
BBdB.00		106134	[M]Meningeal sarcomatosis
BBdz.00	[M]Meningioma NOS	47848	[M]Meningioma NOS
BBm0.00	[M]Microglioma	63973	[M]Microglioma
ByuA200	[X]Malignant neoplasm of meninges, unspecified	63925	[X]Malignant neoplasm of meninges, unspecified
ByuA300	[X]Malig neopl, overlap lesion brain & other part of CNS	47633	[X]Malig neopl, overlap lesion brain & other part of CNS

Supplemental table 1 Read and CPRD Medical Codes used to identify primary and secondary brain tumours.

Product Code	Product Name	PPARa Targeting?
25	Simvastatin 20mg tablets	No
28	Atorvastatin 10mg tablets	No
42	Simvastatin 10mg tablets	No
51	Simvastatin 40mg tablets	No
75	Atorvastatin 20mg tablets	No
184	Bezafibrate 200mg tablets	Yes
379	Fluvastatin 20mg capsules	No
490	Pravastatin 10mg tablets	No
602	Bezafibrate 400mg modified-release tablets	Yes
644	Colestyramine 4g oral powder sachets	No
653	Ezetimibe 10mg tablets	No
713	Rosuvastatin 10mg tablets	No
730	Pravastatin 20mg tablets	No
745	Atorvastatin 40mg tablets	No
802	Simvador 40mg tablets (Discovery Pharmaceuticals)	No
818	Simvastatin 20mg/5ml oral solution sugar free	No
1212	Colestipol 5g granules sachets sugar free	No
1214	Bezalip 400mg Tablet (Roche Products Ltd)	Yes
1215	Fenofibrate 100mg Capsule	Yes
1217	Lipantil micro 200 200mg Capsule (Fournier Pharmaceuticals Ltd)	Yes
1219	Pravastatin 40mg tablets	No
1221	Lipostat 10mg tablets (Bristol-Myers Squibb Pharmaceuticals Ltd)	No
1223	Lipostat 40mg tablets (Bristol-Myers Squibb Pharmaceuticals Ltd)	No
1322	Clofibrate 500mg capsules	Yes
1324	Bezalip 200mg Tablet (Roche Products Ltd)	Yes
1477	Atromid -s 500mg Capsule (AstraZeneca UK Ltd)	Yes
1716	Questran 4g oral powder sachets (Bristol-Myers Squibb Pharmaceuticals Ltd)	No
1764	Questran Light 4g oral powder sachets (Bristol-Myers Squibb Pharmaceuticals Ltd)	No
2137	Fluvastatin 40mg capsules	No
2215	Lopid 300mg capsules (Pfizer Ltd)	Yes
2435	Lipantil 100mg Capsule (Fournier Pharmaceuticals Ltd)	Yes
2718	Zocor 10mg tablets (Merck Sharp & Dohme Ltd)	No
2955	Lipitor 40mg tablets (Pfizer Ltd)	No
3089	Ciprofibrate 100mg tablets	Yes
3159	Fenofibrate 200mg capsules	Yes
3318	Gemfibrozil 300mg capsules	Yes
3411	Lipitor 10mg tablets (Pfizer Ltd)	No
3690	Lipostat 20mg tablets (Bristol-Myers Squibb Pharmaceuticals Ltd)	No
4062	Lopid 600mg tablets (Pfizer Ltd)	Yes
4067	Olbetam 250mg capsules (Pfizer Ltd)	No
4920	Fenofibrate micronised 200mg capsules	Yes
4928	Lipantil Micro 200 capsules (BGP Products Ltd)	Yes
5148	Simvastatin 80mg tablets	No
5216	Bezalip mono 400mg Modified-release tablet (Roche Products Ltd)	Yes
5390	Fenofibrate micronised 267mg capsules	Yes
5564	Colestid Orange sachets (Pharmacia Ltd)	No
5775	Atorvastatin 80mg tablets	No
5985	Lescol XL 80mg tablets (Novartis Pharmaceuticals UK Ltd)	No
6120	Ezetrol 10mg tablets (Merck Sharp & Dohme Ltd)	No
6155	Colestyramine with aspartame 4g sugar free powder	No
6168	Zocor 40mg tablets (Merck Sharp & Dohme Ltd)	No
6213	Rosuvastatin 20mg tablets	No

6365	Colestid 5g granules sachets plain (Pfizer Ltd)	No	11815	Simvastatin 20mg with ezetimibe 10mg tablet	No
7196	Zocor 20mg tablets (Merck Sharp & Dohme Ltd)	No	11976	Niaspan 500mg modified-release tablets (Abbott Laboratories Ltd)	No
7347	Crestor 10mg tablets (AstraZeneca UK Ltd)	No	12211	Nicotinic acid 50mg tablets	No
7374	Lipitor 20mg tablets (Pfizer Ltd)	No	13041	Simvador 10mg tablets (Discovery Pharmaceuticals)	No
7540	Lipantil Micro 267 capsules (BGP Products Ltd)	Yes	14219	Simvastatin 80mg / Ezetimibe 10mg tablets	No
7544	Niaspan 750mg modified-release tablets (Abbott Laboratories Ltd)	No	14379	Lipantil Micro 67 capsules (BGP Products Ltd)	Yes
7551	Niaspan 1g modified-release tablets (Abbott Laboratories Ltd)	No	14963	Nicotinic acid 500mg modified-release tablets	No
7552	Simvastatin 20mg / Ezetimibe 10mg tablets	No	15252	Crestor 20mg tablets (AstraZeneca UK Ltd)	No
7554	Rosuvastatin 5mg tablets	No	16186	Inegy 10mg/80mg tablets (Merck Sharp & Dohme Ltd)	No
8082	Gemfibrozil 600mg tablets	Yes	17059	Inegy 10mg/40mg tablets (Merck Sharp & Dohme Ltd)	No
8104	Acipimox 250mg capsules	No	17614	Zimbacol XL 400mg tablets (Archimedes Pharma UK Ltd)	Yes
8380	Lescol 20mg capsules (Novartis Pharmaceuticals UK Ltd)	No	17683	Lipitor 80mg tablets (Pfizer Ltd)	No
8706	Modalim 100mg tablets (Sanofi)	Yes	17688	Crestor 5mg tablets (AstraZeneca UK Ltd)	No
9153	Lescol 40mg capsules (Novartis Pharmaceuticals UK Ltd)	No	17813	Nicotinic acid 100mg Tablet	No
9491	Fenofibrate micronised 67mg capsules	Yes	17824	Nicotinic acid 25mg Tablet	No
9639	Fenofibrate micronised 160mg tablets	Yes	18081	Colestid Orange 5g granules sachets (Pfizer Ltd)	No
9716	Supralip 160mg tablets (BGP Products Ltd)	Yes	18098	Nicotinic acid 375mg + 500mg + 750mg Modified-release tablet	No
9897	Rosuvastatin 40mg tablets	No	18126	Nicotinic acid 1g modified-release tablets	No
9920	Simvador 20mg tablets (Discovery Pharmaceuticals)	No	19938	Colestipol with aspartame granules	No
9930	Crestor 40mg tablets (AstraZeneca UK Ltd)	No	21020	Inegy 10mg/20mg tablets (Merck Sharp & Dohme Ltd)	No
10172	Simvastatin 40mg / Ezetimibe 10mg tablets	No	22579	Zocor 80mg tablets (Merck Sharp & Dohme Ltd)	No
10183	Simvastatin 40mg with ezetimibe 10mg tablet	No	23153	Liparol 400 XL tablets (Ashbourne Pharmaceuticals Ltd)	Yes
10206	Simvastatin 80mg with ezetimibe 10mg tablet	No			
11627	Fluvastatin 80mg modified-release tablets	No			
11785	Colestyramine 4g oral powder sachets sugar free	No			

23153	Liparol 400 XL tablets (Ashbourne Pharmaceuticals Ltd)	No
23634	GEMFIBROZIL	Yes
24009	NICOTINIC ACID 500 MG TAB	No
24084	Colestyramine 4g oral powder sachets sugar free (PLIVA Pharma Ltd)	No
24509	SIMVASTATIN	No
24583	Nicotinic acid 750mg modified-release tablets	No
25018	BEZAFIBRATE	Yes
29213	Bezagen XL 400mg tablets (Mylan Ltd)	Yes
29328	Bezafibrate 200mg tablets (A A H Pharmaceuticals Ltd)	Yes
29438	SIMVASTATIN	No
31221	Bezafibrate 200mg tablets (Mylan Ltd)	Yes
31783	Fenogal 200mg capsules (Genus Pharmaceuticals Ltd)	Yes
31930	Zocor heart-pro 10mg Tablet (McNeil Products Ltd)	No
32110	Colestyramine 4g Sachets (Dominion Pharma)	No
32909	Simvastatin 80mg tablets (A A H Pharmaceuticals Ltd)	No
32921	Pravastatin 10mg Tablet (Dr Reddy's Laboratories (UK) Ltd)	No
33082	Simvastatin 20mg tablets (A A H Pharmaceuticals Ltd)	No
33603	Fibrazate XL 400mg tablets (Sandoz Ltd)	Yes
33944	Bezafibrate 200mg tablets (Teva UK Ltd)	Yes
34181	Bezafibrate 400mg Modified-release tablet (Hillcross Pharmaceuticals Ltd)	Yes
34201	Colestyramine 4g oral powder sachets sugar free (Actavis UK Ltd)	No
34277	Gemfibrozil 600mg tablets (Teva UK Ltd)	Yes
34312	Simvastatin 20mg tablets (Mylan Ltd)	No
34316	Simvastatin 20mg tablets (Teva UK Ltd)	No
34353	Simvastatin 40mg tablets (Mylan Ltd)	No
34366	Simvastatin 20mg tablets (IVAX Pharmaceuticals UK Ltd)	No
34376	Simvastatin 40mg tablets (Teva UK Ltd)	No
34381	Simvastatin 40mg tablets (IVAX Pharmaceuticals UK Ltd)	No
34476	Simvastatin 20mg Tablet (Ratiopharm UK Ltd)	No
34481	Simvastatin 10mg tablets (IVAX Pharmaceuticals UK Ltd)	No
34502	Simvastatin 40mg tablets (A A H Pharmaceuticals Ltd)	No
34535	Simvastatin 10mg tablets (Mylan Ltd)	No
34545	Simvastatin 40mg Tablet (Ratiopharm UK Ltd)	No
34560	Simvastatin 10mg Tablet (Ratiopharm UK Ltd)	No
34746	Simvastatin 20mg Tablet (Niche Generics Ltd)	No
34814	Simvastatin 20mg tablets (Wockhardt UK Ltd)	No
34820	Pravastatin 40mg tablets (A A H Pharmaceuticals Ltd)	No
34879	Simvastatin 40mg Tablet (Niche Generics Ltd)	No
34891	Simvastatin 20mg tablets (Kent Pharmaceuticals Ltd)	No
34907	Simvastatin 40mg tablets (Wockhardt UK Ltd)	No
34955	Simvastatin 10mg tablets (A A H Pharmaceuticals Ltd)	No
34969	Simvastatin 40mg tablets (Actavis UK Ltd)	No
36377	Pravastatin 20mg tablets (Teva UK Ltd)	No
37266	Colesevelam 625mg tablets	No
37434	Simvastatin 40mg tablets (Sandoz Ltd)	No
37953	Cholestagel 625mg tablets (Sanofi)	No

39060	Simvastatin 20mg tablets (Dexcel-Pharma Ltd)	No	45245	Simvastatin 20mg tablets (Actavis UK Ltd)	No
39420	Bezalip Mono 400mg modified-release tablets (Teva UK Ltd)	Yes	45346	Simvastatin 40mg tablets (Arrow Generics Ltd)	No
39576	Bezalip 200mg tablets (Teva UK Ltd)	Yes	46878	Simvastatin 40mg tablets (Almus Pharmaceuticals Ltd)	No
39652	Simvastatin 40mg/5ml oral solution sugar free	No	46956	Simvastatin 80mg tablets (Arrow Generics Ltd)	No
39675	Simvastatin 20mg/5ml Oral suspension (Martindale Pharmaceuticals Ltd)	No	47023	Omega-3 fish oil with glycerol Emulsion for infusion	No
39870	Simvador 80mg tablets (Discovery Pharmaceuticals)	No	47065	Atorvastatin 20mg chewable tablets sugar free	No
40340	Simvastatin 10mg tablets (Teva UK Ltd)	No	47090	Atorvastatin 10mg chewable tablets sugar free	No
40382	Pravastatin 20mg tablets (A A H Pharmaceuticals Ltd)	No	47630	Lipitor 20mg chewable tablets (Pfizer Ltd)	No
40601	Simvastatin 20mg tablets (Ranbaxy (UK) Ltd)	No	47721	Lipitor 10mg chewable tablets (Pfizer Ltd)	No
40729	Tredaptive 1000mg/20mg modified-release tablets (Merck Sharp & Dohme Ltd)	No	47774	Simvastatin 10mg tablets (Arrow Generics Ltd)	No
40885	Nicotinic acid 1g / Laropiprant 20mg modified-release tablets	No	47935	Fenofibrate 200mg Capsule (Teva UK Ltd)	Yes
41396	Fenofibrate micronised 200mg capsules (A A H Pharmaceuticals Ltd)	Yes	47948	Simvastatin 10mg tablets (Tillomed Laboratories Ltd)	No
41657	Simvastatin 80mg tablets (Teva UK Ltd)	No	47988	Pravastatin 40mg tablets (Mylan Ltd)	No
42801	Bezafibrate xl 400mg Modified-release tablet (Generics (UK) Ltd)	Yes	48018	Simvastatin 20mg tablets (Arrow Generics Ltd)	No
43218	Pravastatin 10mg tablets (Teva UK Ltd)	No	48051	Simvastatin 10mg tablets (Kent Pharmaceuticals Ltd)	No
44528	Simvastatin 20mg/5ml oral suspension sugar free (Rosemont Pharmaceuticals Ltd)	No	48058	Simvastatin 10mg tablets (Ranbaxy (UK) Ltd)	No
44650	Simvastatin 40mg tablets (Dexcel-Pharma Ltd)	No	48078	Simvastatin 10mg tablets (Actavis UK Ltd)	No
44878	Ranzolont 10mg tablets (Ranbaxy (UK) Ltd)	No	48097	Pravastatin 40mg tablets (Teva UK Ltd)	No
45219	Simvastatin 40mg tablets (Kent Pharmaceuticals Ltd)	No	48221	Simvastatin 20mg/5ml oral suspension sugar free	No
45235	Simvastatin 20mg tablets (Sandoz Ltd)	No	48346	Atorvastatin 60mg tablets	No
			48431	Simvastatin 40mg/5ml oral suspension sugar free	No
			48518	Atorvastatin 10mg/5ml oral solution	No
			48585	Nicotinic acid 500mg capsules	No
			48867	Simvastatin 40mg tablets (Alliance Healthcare (Distribution) Ltd)	No

48973	Atorvastatin 30mg tablets	No
49061	Simvastatin 40mg tablets (Bristol Laboratories Ltd)	No
49062	Simvastatin 20mg tablets (Alliance Healthcare (Distribution) Ltd)	No
49558	Atorvastatin 20mg tablets (A A H Pharmaceuticals Ltd)	No
49587	Simvastatin 80mg tablets (Almus Pharmaceuticals Ltd)	No
49609	Bezafibrate 400mg Modified-release tablet (Sandoz Ltd)	Yes
49751	Atorvastatin 40mg tablets (Alliance Healthcare (Distribution) Ltd)	No
50071	Fenofibrate 160mg Tablet (Teva UK Ltd)	Yes
50236	Atorvastatin 10mg tablets (Zentiva)	No
50272	Atorvastatin 40mg tablets (Pfizer Ltd)	No
50483	Simvastatin 40mg tablets (Relonchem Ltd)	No
50564	Simvastatin 20mg tablets (Relonchem Ltd)	No
50670	Simvastatin 40mg tablets (Aurobindo Pharma Ltd)	No
50703	Simvastatin 40mg tablets (Accord Healthcare Ltd)	No
50754	Simvastatin 20mg tablets (Medreich Plc)	No
50788	Atorvastatin 20mg tablets (Pfizer Ltd)	No
50790	Atorvastatin 20mg tablets (Dexcel-Pharma Ltd)	No
50882	Simvastatin 40mg tablets (Somex Pharma)	No
50925	Pravastatin 10mg tablets (Sigma Pharmaceuticals Plc)	No
50963	Atorvastatin 40mg tablets (Teva UK Ltd)	No
51085	Simvastatin 10mg tablets (Medreich Plc)	No
51134	Atorvastatin 10mg tablets (A A H Pharmaceuticals Ltd)	No
51155	Natures Aid Omega-3 Fish Oil 1000mg softgels capsules (Natures Aid Ltd)	No
51166	Simvastatin 40mg tablets (Medreich Plc)	No
51200	Atorvastatin 40mg tablets (Arrow Generics Ltd)	No
51233	Simvastatin 10mg tablets (Alliance Healthcare (Distribution) Ltd)	No
51359	Atorvastatin 20mg tablets (Arrow Generics Ltd)	No
51483	Simvastatin 20mg tablets (Aurobindo Pharma Ltd)	No
51622	Atorvastatin 20mg tablets (Consilient Health Ltd)	No
51676	Pravastatin 40mg tablets (Medreich Plc)	No
51715	Simvastatin 10mg tablets (Sigma Pharmaceuticals Plc)	No
51822	Natures Aid Omega-3 Fish Oil 500mg capsules (Natures Aid Ltd)	No
51876	Atorvastatin 40mg tablets (Consilient Health Ltd)	No
51890	Pravastatin 20mg tablets (Medreich Plc)	No
52097	Atorvastatin 40mg tablets (Wockhardt UK Ltd)	No
52098	Simvastatin 40mg tablets (Ranbaxy (UK) Ltd)	No
52168	Atorvastatin 20mg tablets (Aspire Pharma Ltd)	No
52211	Atorvastatin 20mg tablets (Actavis UK Ltd)	No
52257	Simvastatin 20mg tablets (Accord Healthcare Ltd)	No
52397	Atorvastatin 40mg tablets (Dr Reddy's Laboratories (UK) Ltd)	No
52398	Atorvastatin 40mg tablets (A A H Pharmaceuticals Ltd)	No
52459	Atorvastatin 80mg tablets (Actavis UK Ltd)	No
52460	Atorvastatin 40mg tablets (Aspire Pharma Ltd)	No
52625	Simvastatin 10mg tablets (Wockhardt UK Ltd)	No
52676	Simvastatin 10mg/5ml oral suspension	No
52755	Pravastatin 20mg tablets (Alliance Healthcare (Distribution) Ltd)	No

52812	Simvastatin 20mg tablets (Sigma Pharmaceuticals Plc)	No	54435	Pravastatin 40mg tablets (Almus Pharmaceuticals Ltd)	No
52814	Bezafibrate 400mg modified-release tablets (Alliance Healthcare (Distribution) Ltd)	Yes	54493	Simvastatin 10mg tablets (Relonchem Ltd)	No
			54535	Atorvastatin 10mg tablets (Pfizer Ltd)	No
52821	Atorvastatin 80mg tablets (Dr Reddy's Laboratories (UK) Ltd)	No	54606	Simvastatin 20mg/5ml oral suspension sugar free (A A H Pharmaceuticals Ltd)	No
52953	Simvastatin 20mg tablets (Bristol Laboratories Ltd)	No	54607	Pravastatin 20mg tablets (Almus Pharmaceuticals Ltd)	No
52962	Simvastatin 80mg tablets (Medreich Plc)	No			
53087	Simvastatin 20mg tablets (Somex Pharma)	No	54655	Simvastatin 10mg tablets (Accord Healthcare Ltd)	No
53250	Modalim 100mg tablets (Lexon (UK) Ltd)	Yes	54819	Simvastatin 40mg/5ml oral suspension sugar free (Rosemont Pharmaceuticals Ltd)	No
53340	Zocor 40mg tablets (Lexon (UK) Ltd)	No			
53415	Simvastatin 10mg tablets (Aurobindo Pharma Ltd)	No	54947	Simvastatin 20mg tablets (Almus Pharmaceuticals Ltd)	No
53460	Crestor 10mg tablets (DE Pharmaceuticals)	No	54976	Simvastatin 10mg tablets (Somex Pharma)	No
53594	Lipitor 80mg tablets (Mawdsley-Brooks & Company Ltd)	No	54985	Simvastatin 40mg/5ml oral suspension	No
53676	Simvastatin 20mg tablets (Tillomed Laboratories Ltd)	No	54992	Atorvastatin 10mg/5ml oral suspension	No
			55032	Atorvastatin 10mg tablets (Dexcel-Pharma Ltd)	No
53770	Fluvastatin 40mg capsules (A A H Pharmaceuticals Ltd)	No	55034	Atorvastatin 40mg/5ml oral suspension	No
53772	Atorvastatin 80mg tablets (Alliance Healthcare (Distribution) Ltd)	No	55444	Atorvastatin 40mg tablets (Zentiva)	No
			55452	Simvastatin 20mg tablets (Phoenix Healthcare Distribution Ltd)	No
53822	Simvastatin 10mg tablets (Bristol Laboratories Ltd)	No			
53887	Atorvastatin 40mg tablets (Actavis UK Ltd)	No	55727	Atorvastatin 10mg tablets (Actavis UK Ltd)	No
53890	Atorvastatin 80mg tablets (Pfizer Ltd)	No	55912	Pravastatin 40mg tablets (Alliance Healthcare (Distribution) Ltd)	No
53908	Simvastatin 10mg tablets (Dexcel-Pharma Ltd)	No			
53966	Simvastatin 40mg tablets (Phoenix Healthcare Distribution Ltd)	No	56065	Simvastatin 20mg/5ml oral suspension sugar free (Waymade Healthcare Plc)	No
54240	Simvastatin 40mg tablets (Sigma Pharmaceuticals Plc)	No	56097	Atorvastatin 10mg chewable tablets sugar free	No
54266	Simvastatin 20mg/5ml oral suspension	No	56146	Pravastatin 10mg tablets (Waymade Healthcare Plc)	No

56165	Atorvastatin 20mg chewable tablets sugar free	No
56182	Atorvastatin 80mg tablets (Zentiva)	No
56248	Atorvastatin 20mg tablets (Sigma Pharmaceuticals Plc)	No
56481	Zocor 10mg tablets (Sigma Pharmaceuticals Plc)	No
56494	Zocor 20mg tablets (Sigma Pharmaceuticals Plc)	No
56564	Atorvastatin 20mg tablets (Almus Pharmaceuticals Ltd)	No
56607	Pravastatin 20mg tablets (Waymade Healthcare Plc)	No
56735	Pravastatin 20mg tablets (Mylan Ltd)	No
56841	Atorvastatin 40mg tablets (Dexcel-Pharma Ltd)	No
56893	Pravastatin 40mg tablets (Accord Healthcare Ltd)	No
56916	Pravastatin 40mg tablets (PLIVA Pharma Ltd)	No
57108	Pravastatin 40mg tablets (Waymade Healthcare Plc)	No
57117	Atorvastatin 80mg tablets (Waymade Healthcare Plc)	No
57137	Pravastatin 10mg tablets (Almus Pharmaceuticals Ltd)	No
57219	Fenofibrate micronised 200mg capsules (Sandoz Ltd)	Yes
57296	Pravastatin 20mg tablets (Phoenix Healthcare Distribution Ltd)	No
57329	Simvastatin 25mg/5ml oral suspension	No
57348	Atorvastatin 10mg tablets (Consilient Health Ltd)	No
57397	Pravastatin 10mg tablets (Accord Healthcare Ltd)	No
57489	Ciprofibrate 100mg tablets (Zentiva)	Yes
57568	Zocor 10mg tablets (Lexon (UK) Ltd)	No
57763	Rosuvastatin 10mg tablets (Waymade Healthcare Plc)	No
57834	Atorvastatin 40mg tablets (DE Pharmaceuticals)	No
57836	Atorvastatin 80mg tablets (Teva UK Ltd)	No
57999	Crestor 40mg tablets (Lexon (UK) Ltd)	No
58041	Atorvastatin 20mg tablets (Teva UK Ltd)	No
58110	Atorvastatin 20mg tablets (Zentiva)	No
58315	Simvastatin 20mg tablets (Waymade Healthcare Plc)	No
58394	Atorvastatin 20mg tablets (Alliance Healthcare (Distribution) Ltd)	No
58418	Atorvastatin 80mg tablets (A A H Pharmaceuticals Ltd)	No
58617	Rosuvastatin 20mg/5ml oral suspension	No
58635	Bezalip Mono 400mg modified-release tablets (DE Pharmaceuticals)	Yes
58742	Atorvastatin 80mg tablets (Arrow Generics Ltd)	No
58755	Simvastatin 10mg tablets (Phoenix Healthcare Distribution Ltd)	No
58834	Atorvastatin 10mg tablets (DE Pharmaceuticals)	No
58868	Atorvastatin 10mg tablets (Sigma Pharmaceuticals Plc)	No
59002	Bezafibrate 400mg modified-release tablets (DE Pharmaceuticals)	Yes
59272	Atorvastatin 20mg tablets (Waymade Healthcare Plc)	No
59278	Fluvastatin 20mg capsules (Zentiva)	No
59331	Lipitor 10mg tablets (DE Pharmaceuticals)	No
59357	Atorvastatin 10mg tablets (Ranbaxy (UK) Ltd)	No
59446	Atorvastatin 40mg tablets (Almus Pharmaceuticals Ltd)	No

59447	Crestor 20mg tablets (Waymade Healthcare Plc)	No
59452	Rosuvastatin 5mg tablets (Waymade Healthcare Plc)	No
59508	Pravastatin 20mg tablets (Accord Healthcare Ltd)	No
59776	Atorvastatin 80mg tablets (Aspire Pharma Ltd)	No
59859	Atorvastatin 10mg tablets (Teva UK Ltd)	No
60101	Colestyramine 4g oral powder sachets sugar free (Teva UK Ltd)	No
60160	Rosuvastatin 5mg tablets (Mawdsley-Brooks & Company Ltd)	No
60251	Pravastatin 10mg tablets (Sandoz Ltd)	No
60342	Berocca effervescent tablets tropical (Bayer Plc)	No
60385	Bezalip Mono 400mg modified-release tablets (Lexon (UK) Ltd)	Yes
60464	Atorvastatin 20mg/5ml oral suspension	No
60511	Atorvastatin 40mg tablets (Ranbaxy (UK) Ltd)	No
60607	Atorvastatin 80mg tablets (DE Pharmaceuticals)	No
60788	Fenofibrate micronised 267mg capsules (Zentiva)	Yes
60989	Atorvastatin 80mg tablets (Phoenix Healthcare Distribution Ltd)	No
61087	Questran Light 4g oral powder sachets (Mawdsley-Brooks & Company Ltd)	No
61134	Pravastatin 20mg tablets (Sigma Pharmaceuticals Plc)	No
61149	Atorvastatin 10mg tablets (Waymade Healthcare Plc)	No
61155	Simvastatin 40mg/5ml oral suspension sugar free (A A H Pharmaceuticals Ltd)	No
61321	Simvastatin 10mg tablets (Sandoz Ltd)	No
61360	Simvastatin 10mg tablets (Almus Pharmaceuticals Ltd)	No
61665	Simvastatin 10mg tablets (Waymade Healthcare Plc)	No
62137	Simvastatin 40mg tablets (Waymade Healthcare Plc)	No
62148	Fluvastatin 20mg capsules (Actavis UK Ltd)	No
62219	Atorvastatin 20mg tablets (DE Pharmaceuticals)	No
62429	Atorvastatin 20mg tablets (DE Pharmaceuticals)	No
62476	Atorvastatin 80mg tablets (Almus Pharmaceuticals Ltd)	No
62979	Pravastatin 40mg tablets (Kent Pharmaceuticals Ltd)	No
63074	Pravastatin 20mg tablets (PLIVA Pharma Ltd)	No
63140	Atorvastatin 10mg tablets (Alliance Healthcare (Distribution) Ltd)	No
63249	Atorvastatin 80mg tablets (Consilient Health Ltd)	No
63343	Generic Crampex tablets	No
63469	Atorvastatin 30mg tablets (Consilient Health Ltd)	No
63737	Fenofibrate micronised 267mg capsules (Sigma Pharmaceuticals Plc)	Yes
63787	Pravastatin 10mg tablets (Tillomed Laboratories Ltd)	No
64067	Atorvastatin 20mg/5ml oral solution	No
64104	Simvastatin 20mg tablets (Crescent Pharma Ltd)	No
64180	Simvastatin 10mg tablets (Crescent Pharma Ltd)	No
64307	Simvastatin 40mg tablets (Crescent Pharma Ltd)	No
64503	Bezalip Mono 400mg modified-release tablets (Waymade Healthcare Plc)	Yes
64702	Atorvastatin 30mg tablets (A A H Pharmaceuticals Ltd)	No

64810	Atorvastatin 40mg tablets (Phoenix Healthcare Distribution Ltd)	No
64825	Atorvastatin 10mg tablets (Phoenix Healthcare Distribution Ltd)	No
64868	Atorvastatin 40mg tablets (Sigma Pharmaceuticals Plc)	No
64933	Fenofibrate micronised 267mg capsules (Ranbaxy (UK) Ltd)	Yes
64968	Simvastatin 10mg tablets (DE Pharmaceuticals)	No
64984	Fenofibrate micronised 160mg tablets (Phoenix Healthcare Distribution Ltd)	Yes
65181	Simvastatin 40mg tablets (DE Pharmaceuticals)	No
65193	Atorvastatin 20mg tablets (Ranbaxy (UK) Ltd)	No
65572	Fenofibrate micronised 160mg tablets (Genus Pharmaceuticals Ltd)	Yes
65679	Simvastatin 20mg tablets (DE Pharmaceuticals)	No
65901	Simvastatin 40mg tablets (Zentiva)	No
65925	Simvastatin 20mg/5ml oral suspension sugar free (Alliance Healthcare (Distribution) Ltd)	No
66087	Modalim 100mg tablets (Mawdsley-Brooks & Company Ltd)	Yes
66425	Bezafibrate 400mg modified-release tablets (A A H Pharmaceuticals Ltd)	Yes
66505	Fenofibrate 145mg / Simvastatin 40mg tablets	Yes
66564	Bezafibrate 400mg modified-release tablets (Phoenix Healthcare Distribution Ltd)	Yes
66780	Fenofibrate 145mg / Simvastatin 20mg tablets	Yes
66963	Atorvastatin 80mg tablets (Sigma Pharmaceuticals Plc)	No
67098	Simvastatin 10mg tablets (Brown & Burk UK Ltd)	No
67157	Fenofibrate micronised 200mg capsules (Phoenix Healthcare Distribution Ltd)	Yes
67328	Lescol XL 80mg tablets (Mawdsley-Brooks & Company Ltd)	No
67329	Lipantil Micro 267 capsules (DE Pharmaceuticals)	Yes
67402	Atorvastatin 40mg tablets (Kent Pharmaceuticals Ltd)	No
67573	Atorvastatin 10mg tablets (DE Pharmaceuticals)	No
67660	Atorvastatin 80mg tablets (Ranbaxy (UK) Ltd)	No
67745	Simvastatin 10mg tablets (Zentiva)	No
67773	Simvastatin 20mg tablets (Zentiva)	No
67829	Pravastatin 20mg tablets (Sandoz Ltd)	No
67846	Atorvastatin 10mg tablets (Almus Pharmaceuticals Ltd)	No
67883	Lomitapide 5mg capsules	No
68023	Atorvastatin 10mg tablets (Aspire Pharma Ltd)	No
68048	Atorvastatin 20mg tablets (Phoenix Healthcare Distribution Ltd)	No
68156	Pravastatin 10mg tablets (A A H Pharmaceuticals Ltd)	No
68386	Colestyramine 4g oral powder sachets (J M McGill Ltd)	No
68467	Atorvastatin 20mg tablets (Kent Pharmaceuticals Ltd)	No
68563	Simvastatin 40mg tablets (Brown & Burk UK Ltd)	No
68686	Simvastatin 20mg tablets (Genesis Pharmaceuticals Ltd)	No
68785	Atorvastatin 10mg tablets (Mylan Ltd)	No
68827	Atorvastatin 20mg tablets (Mylan Ltd)	No
69093	Atorvastatin 80mg tablets (Wockhardt UK Ltd)	No
69413	Simvastatin 20mg tablets (Brown & Burk UK Ltd)	No

69427	Atorvastatin 40mg tablets (Mylan Ltd)	No

Supplemental table 2: Product codes for drugs used for hyperlipidaemia and fibrate exposure status.

Product Code	Product Name	PPARg Targeting?
23	Metformin 500mg tablets	No
32	Gliclazide 80mg tablets	No
93	Metformin 850mg tablets	No
240	Rifampicin 150mg capsules	No
469	Rosiglitazone 4mg tablets	Yes
479	Acarbose 50mg tablets	No
547	Glipizide 2.5mg tablets	No
548	Pioglitazone 15mg tablets	Yes
735	Metformin 100mg/ml Oral solution	No
1254	Glibenclamide 5mg tablets	No
1964	Diamicron 80mg tablets (Servier Laboratories Ltd)	No
1965	Tolbutamide 500mg tablets	No
2219	Glibenclamide 2.5mg tablets	No
4862	Diabetamide 2.5mg tablets (Ashbourne Pharmaceuticals Ltd)	No
5174	Acarbose 100mg tablets	No
5227	Rosiglitazone 8mg tablets	Yes
5276	Glimepiride 1mg tablets	No
5316	Glimepiride 4mg tablets	No
5353	Glimepiride 2mg tablets	No
5621	Glucobay 50mg tablets (Bayer Plc)	No
5627	Gliclazide 30mg modified-release tablets	No
5636	Glipizide 5mg tablets	No
5678	Nateglinide 120mg tablets	No
5989	Nateglinide 180mg tablets	No
6337	Glimepiride 3mg tablets	No
6855	Avandamet 2mg/500mg tablets (GlaxoSmithKline UK Ltd)	Yes
7048	Metformin 500mg modified-release tablets	No
7166	Glucophage 500mg tablets (Merck Serono Ltd)	No
7284	Amaryl 2mg tablets (Zentiva)	No
7325	Avandamet 4mg/1000mg tablets (GlaxoSmithKline UK Ltd)	Yes
7332	Amaryl 1mg tablets (Zentiva)	No
7375	Rosiglitazone 4mg / Metformin 1g tablets	Yes
7409	Amaryl 3mg tablets (Zentiva)	No
7610	Glucophage 850mg tablets (Merck Serono Ltd)	No
7744	Daonil 5mg tablets (Sanofi)	No
7818	Rifater tablets (Sanofi)	No
7912	Semi-Daonil 2.5mg tablets (Sanofi)	No
8976	Euglucon 2.5mg tablets (Aventis Pharma)	No
9105	Glucobay 100mg tablets (Bayer Plc)	No
9662	Avandia 4mg tablets (GlaxoSmithKline UK Ltd)	Yes
9691	Rifampicin with isoniazid & pyrazinamide tablet	No
9699	Pioglitazone 30mg tablets	Yes
9707	Repaglinide 1mg tablets	No
9748	Repaglinide 2mg tablets	No
9865	Repaglinide 500microgram tablets	No
10051	Pioglitazone 45mg tablets	Yes
11284	Amaryl 4mg tablets (Zentiva)	No
11316	NovoNorm 500microgram tablets (Novo Nordisk Ltd)	No
11321	NovoNorm 1mg tablets (Novo Nordisk Ltd)	No
11366	NovoNorm 2mg tablets (Novo Nordisk Ltd)	No
11483	Nateglinide 60mg tablets	No
11601	Rosiglitazone 2mg / Metformin 500mg tablets	Yes
11604	Rosiglitazone 1mg / Metformin 500mg tablets	Yes
11609	Metformin with rosiglitazone 500mg + 1mg Tablet	Yes
11610	Metformin with rosiglitazone 500mg + 2mg Tablet	Yes
11695	Diamicron 30mg MR tablets (Servier Laboratories Ltd)	No
11717	Rosiglitazone 2mg / Metformin 1g tablets	Yes
11737	Metformin with rosiglitazone 1000mg + 4mg Tablet	Yes
11760	Metformin with rosiglitazone 1000mg + 2mg Tablet	Yes
11946	Tolbutamide 50mg/ml Injection	No
11990	Metformin 500mg/5ml oral solution sugar free	No
12455	Rastinon 500mg Tablet (Hoechst Marion Roussel)	No
12513	Glibenese 5mg tablets (Pfizer Ltd)	No
13331	Euglucon 5mg tablets (Sanofi)	No

13628	Romozin 400mg Tablet (Glaxo Wellcome UK Ltd)	Yes
14164	Avandamet 2mg/1000mg tablets (GlaxoSmithKline UK Ltd)	Yes
15232	Avandia 8mg tablets (GlaxoSmithKline UK Ltd)	Yes
15374	Gliclazide 40mg/5ml oral suspension	No
15955	Starlix 120mg tablets (Novartis Pharmaceuticals UK Ltd)	No
16044	Glucophage SR 500mg tablets (Merck Serono Ltd)	No
16602	Calabren 2.5mg Tablet (Berk Pharmaceuticals Ltd)	No
17343	Gliclazide 80mg tablets (A A H Pharmaceuticals Ltd)	No
17580	Avandamet 1mg/500mg tablets (GlaxoSmithKline UK Ltd)	Yes
17698	Minodiab 5mg tablets (Pfizer Ltd)	No
17706	Minodiab 2.5mg tablets (Pfizer Ltd)	No
17770	Glucagon novo 10mg Injection (Novo Nordisk Ltd)	No
18220	Pioglitazone 15mg / Metformin 850mg tablets	Yes
19472	Actos 45mg tablets (Takeda UK Ltd)	Yes
20287	Actos 15mg tablets (Takeda UK Ltd)	Yes
20889	Actos 30mg tablets (Takeda UK Ltd)	Yes
21424	Glibenclamide 5mg/5ml oral suspension	No
21564	Gliclazide 80mg tablets (Wockhardt UK Ltd)	No
21832	Diabetamide 5mg tablets (Ashbourne Pharmaceuticals Ltd)	No
21892	Diaglyk 80mg tablets (Ashbourne Pharmaceuticals Ltd)	No
22239	Glucagon lilly 1mg Injection (Eli Lilly and Company Ltd)	No
23945	Starlix 60mg tablets (Novartis Pharmaceuticals UK Ltd)	No
25636	Libanil 2.5mg Tablet (Approved Prescription Services Ltd)	No
25678	Glucamet 500mg Tablet (Opus Pharmaceuticals Ltd)	No

26218	Calabren 5mg Tablet (Berk Pharmaceuticals Ltd)	No
26258	Glucamet 850mg Tablet (Opus Pharmaceuticals Ltd)	No
27125	Starlix 180mg tablets (Novartis Pharmaceuticals UK Ltd)	No
27501	Orabet 500mg Tablet (Lagap)	No
28708	Malix 2.5mg Tablet (Lagap)	No
29326	Glipizide 5mg tablets (Mylan Ltd)	No
29939	Gliclazide 80mg tablets (Mylan Ltd)	No
30316	Metformin with pioglitazone 850mg + 15mg Tablet	Yes
30460	Malix 5mg Tablet (Lagap)	No
31077	Competact 15mg/850mg tablets (Takeda UK Ltd)	Yes
31146	Metsol 500mg/5ml oral solution (Kappin Ltd)	No
31212	Gliclazide 80mg tablets (Actavis UK Ltd)	No
31474	Libanil 5mg Tablet (Approved Prescription Services Ltd)	No
33087	Metformin 500mg tablets (Actavis UK Ltd)	No
33562	Duclazide 80mg Tablet (Dumex Ltd)	No
33673	Tolbutamide 500mg tablets (Actavis UK Ltd)	No
33674	Metformin 850mg tablets (A A H Pharmaceuticals Ltd)	No
34004	Metformin 500mg tablets (IVAX Pharmaceuticals UK Ltd)	No
34020	Metformin 850mg tablets (IVAX Pharmaceuticals UK Ltd)	No
34135	Metformin 500mg Tablet (M & A Pharmachem Ltd)	No
34323	Metformin 500mg tablets (A A H Pharmaceuticals Ltd)	No
34399	Gliclazide 80mg tablets (IVAX Pharmaceuticals UK Ltd)	No
34504	Metformin 500mg tablets (Wockhardt UK Ltd)	No
34507	Glibenclamide 2.5mg tablets (Wockhardt UK Ltd)	No
34563	Glibenclamide 5mg tablets (Wockhardt UK Ltd)	No
34598	Metformin 500mg tablets (Mylan Ltd)	No

34676	Glibenclamide 2.5mg tablets (A A H Pharmaceuticals Ltd)	No
34697	Metformin 850mg tablets (Wockhardt UK Ltd)	No
34706	Glibenclamide 2.5mg tablets (IVAX Pharmaceuticals UK Ltd)	No
34742	Metformin 850mg tablets (Teva UK Ltd)	No
34802	Glipizide 5mg tablets (IVAX Pharmaceuticals UK Ltd)	No
34836	Metformin 850mg tablets (Actavis UK Ltd)	No
34917	Metformin 500mg tablets (Teva UK Ltd)	No
34932	Gliclazide 80mg tablets (Genus Pharmaceuticals Ltd)	No
34957	Tolbutamide 500mg tablets (A A H Pharmaceuticals Ltd)	No
35022	Sitagliptin 100mg tablets	No
35144	Byetta 5micrograms/0.02ml solution for injection 1.2ml pre-filled disposable devices (AstraZeneca UK Ltd)	No
35149	Exenatide 10micrograms/0.04ml solution for injection 2.4ml pre-filled disposable devices	No
35150	Byetta 10micrograms/0.04ml solution for injection 2.4ml pre-filled disposable devices (AstraZeneca UK Ltd)	No
35251	Exenatide 5micrograms/0.02ml solution for injection 1.2ml pre-filled disposable devices	No
35462	Januvia 100mg tablets (Merck Sharp & Dohme Ltd)	No
35561	Prandin 2mg tablets (Novo Nordisk Ltd)	No
36774	Prandin 1mg tablets (Novo Nordisk Ltd)	No
36856	Gliclazide 80mg tablets (Sandoz Ltd)	No
36948	Prandin 0.5mg tablets (Novo Nordisk Ltd)	No
37617	Rosiglitazone 2mg tablet	Yes
37874	Vildagliptin 50mg / Metformin 850mg tablets	No
37875	Vildagliptin 50mg tablets	No
37902	Vildagliptin 50mg / Metformin 1g tablets	No
38355	Metformin 750mg modified-release tablets	No
38400	Glucophage SR 750mg tablets (Merck Serono Ltd)	No
38551	Eucreas 50mg/1000mg tablets (Novartis Pharmaceuticals UK Ltd)	No
39149	Galvus 50mg tablets (Novartis Pharmaceuticals UK Ltd)	No
39203	Eucreas 50mg/850mg tablets (Novartis Pharmaceuticals UK Ltd)	No
39560	Bolamyn SR 500mg tablets (Teva UK Ltd)	No
39598	Metformin 1g modified-release tablets	No
39729	Glucophage SR 1000mg tablets (Merck Serono Ltd)	No
39988	Metformin 500mg oral powder sachets sugar free	No
40007	Glucophage 1000mg oral powder sachets (Merck Serono Ltd)	No
40110	Glucophage 500mg oral powder sachets (Merck Serono Ltd)	No
40233	Metformin 1g oral powder sachets sugar free	No
40365	Glimepiride 1mg tablets (Actavis UK Ltd)	No
40425	Nazdol MR 30mg tablets (Teva UK Ltd)	No
40642	Victoza 6mg/ml solution for injection 3ml pre-filled pen (Novo Nordisk Ltd)	No
40693	Liraglutide 6mg/ml solution for injection 3ml pre-filled disposable devices	No
41204	Saxagliptin 5mg tablets	No
41431	Onglyza 5mg tablets (AstraZeneca UK Ltd)	No
41558	Glibenclamide 5mg tablets (Teva UK Ltd)	No
41559	Glibenclamide 5mg tablets (A A H Pharmaceuticals Ltd)	No
41593	Glibenclamide 2.5mg tablets (Teva UK Ltd)	No
42161	Orabet 500mg Tablet (Sandoz Ltd)	No
42790	Gliclazide 80mg Tablet (Merck Generics (UK) Ltd)	No
43065	Gliclazide 40mg tablets	No

43270	Metformin 500mg/5ml oral solution sugar free (Rosemont Pharmaceuticals Ltd)	No
43465	Zicron 40mg tablets (Bristol Laboratories Ltd)	No
43619	Metformin 1g / Sitagliptin 50mg tablets	No
43684	Janumet 50mg/1000mg tablets (Merck Sharp & Dohme Ltd)	No
44250	Metformin 500mg/5ml Oral solution (Hillcross Pharmaceuticals Ltd)	No
44304	Glyconon 500mg Tablet (DDSA Pharmaceuticals Ltd)	No
44473	Edicil MR 30mg tablets (Teva UK Ltd)	No
44738	Niddaryl 1mg tablets (Dee Pharmaceuticals Ltd)	No
45215	Gliclazide 80mg Tablet (Neo Laboratories Ltd)	No
45581	Metabet SR 500mg tablets (Morningside Healthcare Ltd)	No
45775	Saxagliptin 2.5mg tablets	No
45821	Onglyza 2.5mg tablets (AstraZeneca UK Ltd)	No
45831	Dacadis MR 30mg tablets (Mylan Ltd)	No
46458	Exenatide 2mg powder and solvent for prolonged-release suspension for injection vials	No
46469	Bydureon 2mg powder and solvent for prolonged-release suspension for injection vials (AstraZeneca UK Ltd)	No
46665	Linagliptin 5mg tablets	No
46716	Trajenta 5mg tablets (Boehringer Ingelheim Ltd)	No
46927	Tolbutamide 500mg tablets (Teva UK Ltd)	No
46989	Metabet SR 1000mg tablets (Morningside Healthcare Ltd)	No
47074	Gliclazide 80mg/5ml oral suspension	No
47894	Nazdol MR 30mg tablets (Consilient Health Ltd)	No
47939	Glucient SR 500mg tablets (Consilient Health Ltd)	No
48056	Gliclazide 80mg tablets (Sovereign Medical Ltd)	No
48139	Pioglitazone 30mg tablets (A A H Pharmaceuticals Ltd)	Yes
48149	Metformin 500mg tablets (Almus Pharmaceuticals Ltd)	No
48401	Sitagliptin 50mg tablets	No
48533	Sitagliptin 25mg tablets	No
49502	Glucophage SR 500mg tablets (Mawdsley-Brooks & Company Ltd)	No
49738	Metformin 1g modified-release tablets (A A H Pharmaceuticals Ltd)	No
50087	Januvia 50mg tablets (Merck Sharp & Dohme Ltd)	No
50124	Januvia 25mg tablets (Merck Sharp & Dohme Ltd)	No
50570	Glucophage SR 500mg tablets (Lexon (UK) Ltd)	No
50821	Metformin 850mg tablets (Pfizer Ltd)	No
50970	Metformin 500mg tablets (Bristol Laboratories Ltd)	No
51080	Metabet SR 1000mg tablets (Actavis UK Ltd)	No
51135	Metformin 500mg modified-release tablets (A A H Pharmaceuticals Ltd)	No
51527	Metformin 500mg tablets (Boston Healthcare Ltd)	No
51955	Gliclazide 80mg tablets (Accord Healthcare Ltd)	No
52203	Enyglid 0.5mg tablets (Consilient Health Ltd)	No
52221	Diagemet XL 500mg tablets (Genus Pharmaceuticals Ltd)	No
52442	Metformin 500mg tablets (Pfizer Ltd)	No
52634	Glucophage SR 500mg tablets (DE Pharmaceuticals)	No
53288	Gliclazide 30mg modified-release tablets (A A H Pharmaceuticals Ltd)	No
53478	Metformin 500mg modified-release tablets (Kent Pharmaceuticals Ltd)	No
53774	Metabet SR 500mg tablets (Actavis UK Ltd)	No
53867	Metformin 500mg tablets (Zentiva)	No
54150	Jentadueto 2.5mg/850mg tablets (Boehringer Ingelheim Ltd)	No
54182	Dapagliflozin 10mg tablets	No

54203	Forxiga 10mg tablets (AstraZeneca UK Ltd)	No
54265	Dapagliflozin 5mg tablets	No
54442	Metformin (roi) 1000mg Tablet	No
54480	Forxiga 5mg tablets (AstraZeneca UK Ltd)	No
54764	Gliclazide 80mg tablets (Arrow Generics Ltd)	No
54891	Saxagliptin 2.5mg / Metformin 1g tablets	No
54898	Metformin 850mg tablets (Almus Pharmaceuticals Ltd)	No
55270	Duformin 500mg Tablet (Dumex Ltd)	No
55711	Metformin 500mg tablets (Alliance Healthcare (Distribution) Ltd)	No
55739	Metformin 500mg tablets (Tillomed Laboratories Ltd)	No
55862	Gliclazide Oral solution	No
56008	Gliclazide 80mg tablets (Almus Pharmaceuticals Ltd)	No
56208	Pioglitazone 15mg tablets (A A H Pharmaceuticals Ltd)	Yes
56376	Rosiglitazone 4mg with glimepiride 4mg tablet	No
56437	Gliclazide 60mg modified-release tablets	No
56965	Komboglyze 2.5mg/1000mg tablets (AstraZeneca UK Ltd)	No
57147	Bolamyn SR 1000mg tablets (Teva UK Ltd)	No
57457	Metformin 500mg tablets (Aurobindo Pharma Ltd)	No
57601	Daonil 5mg tablets (Dowelhurst Ltd)	No
57659	Pioglitazone 30mg tablets (Actavis UK Ltd)	Yes
57830	Gliclazide 30mg modified-release tablets (Alliance Healthcare (Distribution) Ltd)	No
58051	Metformin 500mg/5ml oral solution	No
58607	Metformin 500mg/5ml oral solution sugar free (Zentiva)	No
59620	Glucophage SR 500mg tablets (Waymade Healthcare Plc)	No
59809	Alogliptin 6.25mg tablets	No
60012	Dapagliflozin 5mg / Metformin 1g tablets	No
60074	Metformin 1g modified-release tablets (Waymade Healthcare Plc)	No
60211	Canagliflozin 100mg tablets	No
60286	Metformin 500mg/5ml oral suspension	No
60430	Invokana 100mg tablets (Janssen-Cilag Ltd)	No
60495	Gliclazide 80mg tablets (Teva UK Ltd)	No
60643	Xigduo 5mg/1000mg tablets (AstraZeneca UK Ltd)	No
60968	Metformin 500mg modified-release tablets (Actavis UK Ltd)	No
61043	Sukkarto SR 1000mg tablets (Morningside Healthcare Ltd)	No
61311	Glimepiride 4mg tablets (Sigma Pharmaceuticals Plc)	No
61559	Sukkarto SR 500mg tablets (Morningside Healthcare Ltd)	No
61925	NovoNorm 500microgram tablets (Waymade Healthcare Plc)	No
61957	Gliclazide 40mg tablets (A A H Pharmaceuticals Ltd)	No
62014	Glimepiride 2mg tablets (Accord Healthcare Ltd)	No
62034	Laaglyda MR 60mg tablets (Consilient Health Ltd)	No
62144	Metformin 500mg modified-release tablets (DE Pharmaceuticals)	No
62265	Metformin 500mg modified-release tablets (Mawdsley-Brooks & Company Ltd)	No
62326	Vipidia 6.25mg tablets (Takeda UK Ltd)	No
62426	Pioglitazone 30mg tablets (Accord Healthcare Ltd)	Yes
62426	Pioglitazone 30mg tablets (Accord Healthcare Ltd)	No
62605	Metformin 850mg tablets (Kent Pharmaceuticals Ltd)	No
62661	Bydureon 2mg powder and solvent for prolonged-release suspension for injection pre-filled pen (AstraZeneca UK Ltd)	No
62824	Metformin 1g modified-release tablets (Actavis UK Ltd)	No
63045	Metformin 850mg tablets (Relonchem Ltd)	No

63046	Pioglitazone 45mg tablets (A A H Pharmaceuticals Ltd)	Yes
63048	Gliclazide 80mg tablets (Alliance Healthcare (Distribution) Ltd)	No
63107	Pioglitazone 45mg tablets (Waymade Healthcare Plc)	Yes
63131	Ziclaseg 30mg modified-release tablets (Lupin (Europe) Ltd)	No
63336	Trulicity 1.5mg/0.5ml solution for injection pre-filled pen (Eli Lilly and Company Ltd)	No
63401	Trulicity 0.75mg/0.5ml solution for injection pre-filled pen (Eli Lilly and Company Ltd)	No
63421	Pioglitazone 30mg tablets (Teva UK Ltd)	Yes
63516	Forxiga 10mg tablets (Waymade Healthcare Plc)	No
63785	Dulaglutide 0.75mg/0.5ml solution for injection pre-filled disposable devices	No
63823	Dulaglutide 1.5mg/0.5ml solution for injection pre-filled disposable devices	No
64217	Jardiance 25mg tablets (Boehringer Ingelheim Ltd)	No
64900	Glidipion 30mg tablets (Actavis UK Ltd)	Yes
64939	Glucient SR 1000mg tablets (Consilient Health Ltd)	No
65083	Synjardy 5mg/1000mg tablets (Boehringer Ingelheim Ltd)	No
65344	Empagliflozin 5mg / Metformin 850mg tablets	No
65562	Pioglitazone 30mg tablets (Alliance Healthcare (Distribution) Ltd)	Yes
65563	Pioglitazone 15mg tablets (Alliance Healthcare (Distribution) Ltd)	Yes
65694	Metformin 500mg modified-release tablets (Waymade Healthcare Plc)	No
65923	Metformin 1g modified-release tablets (Mawdsley-Brooks & Company Ltd)	No
66008	Synjardy 12.5mg/1000mg tablets (Boehringer Ingelheim Ltd)	No
66136	Glucophage SR 1000mg tablets (Waymade Healthcare Plc)	No
66399	Glimepiride 2mg tablets (A A H Pharmaceuticals Ltd)	No
67056	Amaryl 1mg tablets (Lexon (UK) Ltd)	No
67781	Gliclazide 80mg tablets (Milpharm Ltd)	No
68203	Metformin 500mg modified-release tablets (Almus Pharmaceuticals Ltd)	No
68214	Metformin 500mg/5ml oral solution sugar free (A A H Pharmaceuticals Ltd)	No
68289	Glimepiride 4mg tablets (Waymade Healthcare Plc)	No
68389	Metformin 500mg/5ml oral solution sugar free (Pinewood Healthcare)	No
68415	Gliclazide 30mg modified-release tablets (Phoenix Healthcare Distribution Ltd)	No
68636	Metformin 850mg/5ml oral solution sugar free	No
68675	Glimepiride 4mg tablets (Somex Pharma)	No
68819	Gliclazide 80mg tablets (Bristol Laboratories Ltd)	No

Supplemental table 3: Product codes for drugs used for diabetes and glitazone status.

Glitazones Case-Control				
	N = 480	N = 1920		
**Glitazone exposure status**				
**Per-year glitazone exposure**				
**Unexposed**	383 (79.8%)	1460 (76.0%)	Referent	-
**≤ 1 year**	41 (8.5%)	151 (7.9%)	1.11 (0.77, 1.61)	0.59
**1 < years ≤ 2**	23 (4.8%)	89 (4.6%)	1.06 (0.66, 1.71)	0.82
**2 < years ≤ 3**	29 (4.0%)	66 (3.4%)	1.13 (0.67, 1.92)	0.65
**3 < years ≤ 4**	8 (1.7%)	61 (3.2%)	0.52 (0.25, 1.10)	0.07
**4 < years ≤ 5**	2 (0.4%)	41 (2.1%)	0.18 (0.04, 0.77)	0.003
**5 < years ≤ 6**	2 (0.4%)	23 (1.2%)	0.30 (0.07, 1.30)	0.06
**> 6 years**	2 (0.4%)	29 (1.5%)	0.44 (0.10, 1.94)	0.23
**"First drug exposure" status**				
**Glitazone**	4 (0.8%)	13 (0.7%)	1.10 (0.35, 3.44)	0.87
**Other anti-type 2 diabetes drug**	476 (99.2%)	1907 (99.3%)	Referent	-

* Model adjusted for sex, age, IMD, retrospective prescription history (categorised yearly) and mean HbA1c levels. ** OR calculated for per year increase in variable.

Supplemental table 4: Case-control results for brain tumour cases with glitazone exposure in years duration groups and as first drug exposure.

Variable	Cases (%)	Controls (%)	Adjusted OR (95% CI) *	P-value
**Imputed HbA1c levels**				
	**N = 480**	**N = 1920**		
**Glitazone exposure status**				
**Exposed**	97 (20.2%)	460 (24.0%)	0.81 (0.66, 1.00)	0.11
**Unexposed**	383 (79.8%)	1460 (76.0%)	Referent	-
**Mean glitazone exposure duration (longest), days (SD)**	607 (583)	872 (760)	0.87 (0.82, 0.93) **	0.001
**Mean glitazone exposure duration (total), days (SD)**	604 (720)	1002 (823)	0.85 (0.79, 0.91) **	0.00005
**No HbA1c adjustment**				
	**N = 480**	**N = 1920**		
**Glitazone exposure status**				
**Exposed**	97 (20.2%)	460 (24.0%)	0.79 (0.62, 1.02)	0.07
**Unexposed**	383 (79.8%)	1460 (76.0%)	Referent	-
**Mean glitazone exposure duration (longest), days (SD)**	607 (583)	872 (760)	0.87 (0.80, 0.94)	0.0008
**Mean glitazone exposure duration (total), days (SD)**	604 (720)	1002 (823)	0.85 (0.78, 0.92)	0.00004

*** Adjusted for sex, age, IMD and retrospective prescription history. HbA1c adjustment differed according to the analysis.**

**** OR calculated for per year increase in variable.**

Supplemental table 5: Case-control results for brain tumour cases with glitazone exposure with HbA1C imputed or not adjusted in the multivariable models.

Glitazones - 6 months latency period				
	**N = 480**	**N = 1920**		
**Glitazone exposure status**	**Cases (%)**	**Controls (%)**	**Adjusted OR (95% CI) ***	**P-value**
**Exposed**	95 (19.8%)	460 (24.0%)	0.82 (0.64, 1.05)	0.12
**Unexposed**	385 (80.2%)	1460 (76.0%)	Referent	-
**Mean glitazone exposure duration (longest), days (SD)**	574 (581)	770 (651)	0.88 (0.81, 0.96) **	0.002
**Mean glitazone exposure duration (total), days (SD)**	771 (693)	988 (796)	0.89 (0.83, 0.96) **	0.0015
**Glitazones - 12 months latency period**				
	**N = 480**	**N = 1920**		
**Glitazone exposure status**				
**Exposed**	92 (19.2%)	457 (23.8%)	0.79 (0.61, 1.02)	0.07
**Unexposed**	388 (80.8%)	1463 (76.2%)	Referent	-
**Mean glitazone exposure duration (longest), days (SD)**	540 (572)	753 (642)	0.87 (0.79, 0.95) **	0.002
**Mean glitazone exposure duration (total), days (SD)**	670 (690)	958 (774)	0.87 (0.80, 0.94) **	0.0003
**Glitazones - 24 months latency period**				
	**N = 480**	**N = 1920**		
**Glitazone exposure status**				
**Exposed**	86 (17.9%)	449 (23.4%)	0.74 (0.57, 0.96)	0.02
**Unexposed**	394 (82.1%)	1471 (76.6%)	Referent	-
**Mean glitazone exposure duration (longest), days (SD)**	478 (557)	720 (625)	0.83 (0.75, 0.92) **	0.0004
**Mean glitazone exposure duration (total), days (SD)**	593 (672)	901 (730)	0.84 (0.76, 0.91) **	0.000007

Supplemental table 6: Case-control results for brain tumour cases with glitazone exposure for latency periods of 6, 12, and 24 months.
