# Supplementary material for: Use of drugs for hyperlipidaemia and diabetes and risk of primary and secondary brain tumours: nested case–control studies using the UK Clinical Practice Research Datalink (CPRD)
Source: BMJ Open. 2024 Feb 9;14(2):e072026. doi: 10.1136/bmjopen-2023-072026 (PMC10860117; doi:10.1136/bmjopen-2023-072026)
Supplement: Author's manuscript [file bmjopen-2023-072026.draft_revisions.pdf]

BMJ Open is committed to open peer review. As part of this commitment we make the peer review history of every article we publish publicly available.

When an article is published we post the peer reviewers' comments and the authors' responses online. We also post the versions of the paper that were used during peer review. These are the versions that the peer review comments apply to.

The versions of the paper that follow are the versions that were submitted during the peer review process. They are not the versions of record or the final published versions. They should not be cited or distributed as the published version of this manuscript.

BMJ Open is an open access journal and the full, final, typeset and author-corrected version of record of the manuscript is available on our site with no access controls, subscription charges or pay-per-view fees (http://bmjopen.bmj.com).

If you have any questions on BMJ Open's open peer review process please email info.bmjopen@bmj.com

BMJ Open

Use of drugs for hyperlipidaemia and diabetes and risk of primary and secondary brain tumours: nested case control studies using the UK Clinical Practice Research Datalink (CPRD).

Journal:	*BMJ Open*
Manuscript ID	bmjopen-2023-072026
Article Type:	Original research
Date Submitted by the Author:	23-Jan-2023
Complete List of Authors:	Robinson, Jamie W; University of Bristol<br>Martin, Richard; University of Bristol, Social Medicine<br>Ozawa, Mio; University College London<br>Elwenspoek, Martha; NIHR CLAHRC West, CLAHRC West; University of Bristol Medical School, Populations Health Sciences<br>Redaniel, Maria Theresa; University of Bristol, NIHR CLAHRC West; University of Bristol, School of Social and Community Medicine<br>Kurian, Kathreena; University of Bristol; University of Bristol<br>Ben-Shlomo, Yoav; University of Bristol, Department of Population Health Sciences; University Hospitals Bristol NHS Foundation Trust
Keywords:	Epidemiology < ONCOLOGY, Head & neck tumours < ONCOLOGY, CLINICAL PHARMACOLOGY

SCHOLARONE™
Manuscripts

BMJ

I, the Submitting Author has the right to grant and does grant on behalf of all authors of the Work (as defined in the below author licence), an exclusive licence and/or a non-exclusive licence for contributions from authors who are: i) UK Crown employees; ii) where BMJ has agreed a CC-BY licence shall apply, and/or iii) in accordance with the terms applicable for US Federal Government officers or employees acting as part of their official duties; on a worldwide, perpetual, irrevocable, royalty-free basis to BMJ Publishing Group Ltd ("BMJ") its licensees and where the relevant Journal is co-owned by BMJ to the co-owners of the Journal, to publish the Work in this journal and any other BMJ products and to exploit all rights, as set out in our licence.

The Submitting Author accepts and understands that any supply made under these terms is made by BMJ to the Submitting Author unless you are acting as an employee on behalf of your employer or a postgraduate student of an affiliated institution which is paying any applicable article publishing charge ("APC") for Open Access articles. Where the Submitting Author wishes to make the Work available on an Open Access basis (and intends to pay the relevant APC), the terms of reuse of such Open Access shall be governed by a Creative Commons licence – details of these licences and which Creative Commons licence will apply to this Work are set out in our licence referred to above.

Other than as permitted in any relevant BMJ Author's Self Archiving Policies, I confirm this Work has not been accepted for publication elsewhere, is not being considered for publication elsewhere and does not duplicate material already published. I confirm all authors consent to publication of this Work and authorise the granting of this licence.

For peer review only

Use of drugs for hyperlipidaemia and diabetes and risk of primary and secondary brain tumours: nested case control studies using the UK Clinical Practice Research Datalink (CPRD).

Jamie W Robinson[1], Richard M Martin[1,2,3], Mio Ozawa[4], Martha M C Elwenspoek[2,5], Maria Theresa Redaniel[2,5], Kathreena M Kurian[1,6]*, Yoav Ben-Shlomo[2,5]*

1. MRC Integrative Epidemiology Unit, University of Bristol, Bristol, BS8 2BN, UK (JWR, RMM, KMK)
2. Department of Population Health Sciences, University of Bristol, Bristol, BS8 1UD, UK (RMM, MMCE, MTR, YBS)
3. National Institute for Health Research (NIHR) Bristol Biomedical Research Centre, University Hospitals Bristol and Weston NHS Foundation Trust and University of Bristol, Bristol, UK (RMM)
4. Population, Policy and Practice Research and Teaching Department, UCL Great Ormond Street Institute of Child Health, 30 Guilford Street, London, WC1N 1EH (MC)
5. The National Institute for Health Research Applied Research Collaboration West (NIHR ARC West), University Hospitals Bristol NHS Foundation Trust, BS1 2NT Bristol, UK (MMCE, MTR, YBS)
6. Brain Tumour Research Centre, University of Bristol, Bristol, BS10 5NB, UK (KMK)

* Joint senior authors.

Corresponding author: Yoav Ben-Shlomo (y.ben-shlomo@bristol.ac.uk) and Kathreena M Kurian (kathreena.kurian@bristol.ac.uk)

Short title: Drugs for hyperlipidaemia and diabetes and brain tumours

Word Count: Abstract: 255, Text: 3,860

Tables: 3

Supplemental tables: 6

For peer review only - http://bmjopen.bmj.com/site/about/guidelines.xhtml

Abstract

Objectives: Previous studies have suggested that fibrates and glitazones may have a role in brain tumour prevention. We examined if there is support for these observations using primary care records from the UK Clinical Practice Research Datalink (CPRD).

Design: We conducted two nested case-control studies using primary and secondary brain tumours identified within CPRD between 2000 to 2016. We selected cases and controls amongst the population of individuals who had been treated with any anti-diabetic or anti-hyperlipidaemic medication to reduce confounding by indication.

Setting: Adults older than 18 years registered with a General Practitioner in the UK contributing data to CPRD.

Results: We identified 7,496 people with any brain tumour (4,471 primary; 3,025 secondary) in total. After restricting cases and controls to those prescribed any anti-diabetic or anti-hyperlipidaemic medication, there were 1,950 cases and 7,791 controls in the fibrate and 480 cases with 1,920 controls in the glitazone analyses. Longer use of glitazones compared to all other anti-diabetic medications was associated with a reduced risk of primary (adjusted odds ratio [aOR] = 0.89 per year, 95% CI: 0.80, 0.98), secondary (aOR = 0.87 per year, 95% CI: 0.77, 0.99) or combined brain tumours (aOR = 0.88 per year, 95% CI: 0.81, 0.95). There was little evidence that fibrate exposure was associated with risk of either primary or secondary brain tumours.

Conclusions: Longer exposure to glitazones was associated with reduced primary and secondary brain tumour risk. Further basic science and population-based research should explore this finding in greater detail, in terms of replication and mechanistic studies.

Key Words: Brain cancer, glioma, secondary brain tumours, metastasis, fibrates, glitazones, observational epidemiology.

For peer review only - http://bmjopen.bmj.com/site/about/guidelines.xhtml

Strengths and limitations of this study

- Large case control studies using database free of selection bias and generalizable to other high income settings.
- Exposure well measured from medical systems avoiding recall bias
- Design accounted for confounding by indication and time-window bias
- Adjustment for individual socioeconomic status used an ecological proxy and residual confounding possible.
- All secondary brain cancers were combined to maintain statistical power

For peer review only

For peer review only - http://bmjopen.bmj.com/site/about/guidelines.xhtml

Introduction

People diagnosed with primary or secondary (breast, lung, skin melanoma, colorectal and kidney) brain tumours typically have less than a 20% five-year survival rate and brain tumours are responsible for the most years of life lost of any cancer [1]. The development of novel cancer drugs is beset by substantial costs and high failure rates. Re-purposing existing approved drugs for different diseases than originally designed potentially saves the considerable time and costs involved in conducting new clinical trials.

Nuclear hormone receptors (NHR) have crucial roles in cellular homeostasis and have been implicated in the development of cancer. Peroxisome proliferator-activated receptors (PPARs) are a family of ligand-activated transcription factors that regulate the expression of genes and, as a subtype of NHRs, are involved in the control of proliferation and differentiation of cells[2-4]. There are three isoforms of PPARs which are designated as -α, -β/δ and -γ. The genes regulated by these transcription factors are also involved in the transport, metabolism and storage of fatty acids, in inflammatory processes and in the onset of type 2 diabetes mellitus[3, 9, 10]. PPARs have been identified as potential candidates in cancer treatment, with some animal models and early-stage human trials showing beneficial outcomes, including arresting development of precursor cancer cells, decreased serum cancer antigens and tolerance by patients[5-8].

PPAR-α and -γ are clinically significant due to the use of agonistic compounds such as fibrates in hyperlipidaemia and thiazolidinediones (glitazones) in diabetes respectively. Both glitazones [11] and fibrates such as fenofibrate cross the blood-brain barrier, albeit at a slower rate[12]. PPARs are expressed in astrocytes, oligodendrocytes and microglia, and may play a role in growth and differentiation of those cells, including brain tumour stem cells[13]. These drugs have the potential for being repurposed for the prevention of brain cancers given their safety profile and cost.

We hypothesized that PPAR-α and -γ agonists (fibrates and glitazones), would be associated with a reduced risk of developing brain tumours compared to other treatments for hyperlipidaemia and diabetes using the UK Clinical Practice Research Datalink (CPRD), a very large population-based primary care database.

Materials and methods

We used the CPRD database which has data from a network of over 2,000 general practitioners (GPs) from more than 670 practices across the UK. Validated clinical

For peer review only - http://bmjopen.bmj.com/site/about/guidelines.xhtml

information is held on over 16 million registered patients, amounting to circa 7% of the UK population [14] and broadly representative of that population [15, 16]. Types of data held within the CPRD include, but are not limited to, patient demographics, referrals, hospital admissions, clinical diagnoses and drug prescriptions. Data from secondary care or hospital visits are routinely fed back into the electronic records' system via staff at GP practices. CPRD is jointly sponsored by the Medicines and Healthcare products Regulatory Agency (MHRA) and the National Institute of Health Research (NIHR). Data were extracted from CPRD GOLD and linked to ONS death registration data and census data on area deprivation (see below).

Study design and eligible Participants

We undertook two nested case control studies from within the CPRD database as the eligibility criteria (see below) for the fibrate and glitazone analyses were different. All participants in the study had to be 18 years or older and registered within the CPRD between 1st January 2000 to 31st December 2016, the former being the first year that glitazones were licensed within the UK. We specified that eligible participants had to be treated with either (i) an anti-hyperlipidaemic or (ii) anti-diabetic medication to reduce the likelihood of "confounding by indication", a type of confounding which can distort the association between drug exposure and outcome when an indication to prescribe a drug (e.g. diabetes) is also associated with the outcome. All brain tumour cases and controls that were not treated with either these classes of drugs were therefore not included in the sample selection and analyses. The sample size was pragmatic based on all available cases from the dataset rather than based an "a priori" sample size calculation to maximise power.

Cases

Incident brain tumour (primary or secondary) cases were identified using a large list of Read codes (**Supplemental table 1**) and were classified as either of primary or secondary cancers using histopathology [17]. For secondary tumours, we did not have information on the site of primary tumour, but we retrospectively searched for another prior cancer diagnosis to estimate the site of the primary cancer. However, if this had been recorded prior to 1st Jan 2000, or recorded at a non-CPRD participating GP, it may have been missing from our file and thus not captured by our retrospective search.

For peer review only - http://bmjopen.bmj.com/site/about/guidelines.xhtml

Controls

We sampled controls using "incidence density sampling", whereby on the date each case was diagnosed we identified the potential pool of controls who did not have a prior diagnosis of a brain tumour. Up to four controls were randomly selected per case (to maximise power) and were age-frequency matched to cases by 10-year age groups (<20, 20-29, 30-39, 40-49, 50-59, 60-69, 70-79 and ≥80 years) and male/female sex to ensure reasonable balance of these variables across case-control strata.

Both "immortal time" and "time-window" biases are potential hazards in pharmaco-epidemiology studies leading to misleading results [18], though the former is not relevant to our case-control study design [19]. Time-window bias occurs in case-control studies when the method of selecting controls results in an artefactually longer time window for potential exposure to be identified than their matched cases resulting in a spurious inverse (protective) association between the exposure and outcome [18]. To prevent time-window bias, we specified that controls must have the same retrospective duration of potential exposure (any drug treatment for either hyperlipidaemia or diabetes) within CPRD as the matched case, based on the case index date plus or minus six months. For example, if a case had 6.3 years of any drug medication exposure prior to their diagnosis date, then only controls who also had between 5.8 and 6.8 years of any drug exposure over the same secular time period were sampled and hence had the same potential for exposure to either a fibrate or glitazone as cases.

For peer review only

Exposures

All participants were treated with some medication for either type 2 diabetes or hyperlipidaemia. If they received a fibrate or glitazone they were classified as "exposed" whilst all the other drugs used for the management of these diseases were classified as the unexposed comparator. Product codes used to determine exposure status are given in **Supplemental table 2 and Supplemental table 3**. We excluded participants with type 1 diabetes, defined as those participants treated solely with insulin therapy, as these patients are very different to those with the more common type 2 diabetes in terms of causes and duration of dysregulated glycaemic control. Participants who started on oral anti-diabetic medication, but at some point in time also received insulin therapy, were eligible for inclusion. Participants on combination therapy were not excluded (for example, both glitazone plus another anti-diabetic medication); these participants were considered as exposed, if one of the

For peer review only - http://bmjopen.bmj.com/site/about/guidelines.xhtml

therapies was the drug of interest. Similarly, some participants were exposed to both fibrates and glitazones and were considered exposed for both studies (however we tested both these assumption in sensitivity analyses; see below).

Exposure was classified in three ways: (i) "Exposed binary" (Yes/No) indicating that a subject has been ever exposed to a prescription for fibrates or glitazones; (ii) "Longest exposure duration" which was the total uninterrupted time (no breaks of 90 days or more between prescriptions) each participant had been prescribed either fibrates or glitazones. If there was more than one such period, then we took the longest duration; (iii) "Total exposure duration", which was the sum of the total prescription duration (with or without interruptions). Exposure duration for both the longest and total exposure duration were categorised as follows: unexposed, ≤ 1 year, >1 and ≤ 2 years, >2 and ≤ 3 years, >3 and ≤ 4 years , >4 and ≤ 5 years , >5 and ≤ 6 years , >6 and ≤ 7 years, >7 years. Due to small numbers of participants reaching longer exposure durations, any duration exposure periods over 6 years were considered in the longest duration category in the glitazone analysis, and over 7 years as the longest duration category in the fibrate analysis.

Potential Confounders

The following variables were considered as potential confounders that might influence risk of developing a brain tumour and the choice of drug that a doctor might prescribe: age group, sex and socioeconomic status (SES). We used the Index of Multiple Deprivation 2015 (IMD) [20], as an ecological proxy for individual SES. This is a commonly used measure in the UK that uses census data on a wide variety of economic and health factors to derive a postcode-based deprivation ranked score so that that a higher score indicates less deprivation. IMD scores were grouped into five equal sized groups from most (quintile 1) to least deprived areas (quintile 5), so we could adjust for any non-linear associations.

In the glitazone analyses, we also tried to adjust for the severity of diabetes as a confounder, as severity may determine choice of anti-diabetic medications and possible cancer risk. We used glycosylated haemoglobin (HbA1c) levels as a measure of glycaemic control and hence severity of diabetes, where available. We converted and standardised the measures to the International Federation of Clinical Chemistry (IFCC) units (mmols/mol). The mean value was calculated using all available HbA1c readings for a subject. A three-level ordinal variable was created: 1, well controlled diabetes (≤ 58 mmols/mol); 2, sub-optimally controlled diabetes (> 58 mmols/mol and ≤ 75 mmols/mol); 3, poorly controlled diabetes (>

For peer review only - http://bmjopen.bmj.com/site/about/guidelines.xhtml

75 mmols/mol). If there were no measures of the participant's HbA1c levels, we assigned them to the well-controlled group based on the assumption that the clinician did not feel it necessary to monitor the patient's glycaemic control (however, see below for sensitivity analyses that examine this assumption). We attempted to restrict HbA1c readings to only pre-baseline measures; however due to missing data, we had to occasionally include post-baseline HbA1c readings as a proxy measure.

Statistical Methods

Both case-control analyses were conducted using logistic regression to compute odds ratios (OR) and 95% confidence intervals (CI) of exposure to fibrate and glitazone drugs versus other drugs used for the same disease. Unadjusted and multivariable models were run, adjusting for the previously mentioned potential confounders with cases stratified by primary and secondary brain tumours and then combined as all brain tumours if there was no evidence of heterogeneity of effect to enhance statistical power. In addition, we adjusted for total potential exposure duration, defined as the time from either diagnosis or control index date and start of any medication first prescribed for either hyperlipidaemia or diabetes. This was one of the matching variables used to avoid time window bias. Exposure duration was analysed both as a continuous ordinal variable and also as a "dummy" variable to check for any evidence of non-linearity in the pattern of the odds ratios. We used logistic regression rather than conditional logistic models, as the latter may introduce collider bias and instead we used the matching variables as covariates in the unmatched logistic model as the recommended procedure (see reference [21] for detailed justification).

Sensitivity Analyses

The following sensitivity analyses were conducted to check the robustness of our main analyses: (i) Potential selection bias: we reclassified exposure only if the participant's first medication was a fibrate or glitazone. Someone whose first treatment was another drug but was later switched to a glitazone or fibrate in this analysis was classified as unexposed; (ii) Dual exposure: to evaluate if one drug had an effect on risk while the other did not, participants were dropped from the analysis if they were exposed to both a fibrate and a glitazone at the same time; (iii) Latency period: analyses were repeated excluding any exposure for short latencies defined as six, 12 and 24 months prior to the index date; and (iv) Missing HbA1c levels: we used multiple imputation (55 datasets generated, combined using Rubin's rules) to predict missing HbA1c levels based on case control status, age, IMD, sex,

For peer review only - http://bmjopen.bmj.com/site/about/guidelines.xhtml

retrospective prescription history, ever exposure to a glitazone, total glitazone exposure and number of consultations, defined as each day the patient had at least one in-person consultation. The MICE algorithm in R [22] was used to impute missing HbA1c levels. We also examined whether HbA1c predicted case control status both in the complete case as well as the imputation dataset. This is because if there was no evidence of an association with HbA1c then it cannot be a confounder and did not need to be included in the multivariable model, avoiding the issue of missing values. Sensitivity analyses were conducted using all brain tumour cases as there was limited statistical power in the separate primary and secondary cancer analyses.

Patient and Public Involvement

Patients or the public were not involved in the design, or conduct, or reporting, or dissemination plans of our research

Results

For the fibrate analyses, there were a total of 1,950 cases, of which 1,094 were primary and 856 were secondary brain tumours and 7,791 matched controls. For the glitazone analyses there were a total of 480 cases, of which 278 were primary and 202 were secondary brain tumours and 1,920 controls. There was little evidence of any important differences in age group, sex and IMD distribution between cases versus controls in both the fibrate and glitazone datasets (**Table 1**). Cases were more likely to have well-controlled HbA1c but this was because they were more likely to have missing HbA1c values which by default were classified as well-controlled diabetes.

For peer review only

Table 1: Distribution of possible confounders by case control status for both fibrate and glitazone datasets.

Variable	Cases (%)	Controls (%)	Crude OR (95% CI)	P
**Fibrates Case-Control**				
	**N = 1950**	**N = 7791**		
**Male**	1118 (57.3%)	4469 (57.4%)	Referent	-
**Female**	832 (42.7%)	3322 (42.6%)	1.00 (0.91, 1.11)	0.98
**Age, years**				
**< 20**	0 (0.0%)	0 (0.0%)	-	-
**20 – 29**	0 (0.0%)	0 (0.0%)	-	-
**30 – 39**	2 (0.1%)	5 (0.1%)	1.58 (0.31, 8.17)	0.58
**40 – 49**	6 (0.3%)	29 (0.4%)	0.82 (0.34, 1.98)	0.66
**50 – 59**	40 (2.1%)	193 (2.5%)	0.82 (0.58, 1.16)	0.26
**60 – 69**	220 (11.3%)	848 (10.9%)	1.03 (0.87, 1.21)	0.75
**70 – 79**	561 (28.8%)	2279 (29.3%)	0.97 (0.87, 1.09)	0.65
**≥ 80**	1121 (57.5%)	4437 (57.0%)	Referent	-
**IMD, quintile**				
**1, lower SES**	457 (23.4%)	1768 (22.7%)	1.05 (0.91, 1.22)	0.48
**2**	439 (22.5%)	1790 (23.0%)	Referent	-
**3**	430 (22.1%)	1594 (20.5%)	1.10 (0.95, 1.28)	0.21
**4**	331 (17.0%)	1466 (18.8%)	0.92 (0.79, 1.08)	0.31
**5, higher SES**	293 (15.0%)	1173 (15.1%)	1.02 (0.86, 1.20)	0.83
**Glitazones Case-Control**				
	**N = 480**	**N = 1920**		
**Male**	277 (57.7%)	1108 (57.7%)	Referent	-
**Female**	203 (42.3%)	812 (42.3%)	1.00 (0.82, 1.22)	1.00
**Age, years**				
**< 20**	1 (0.2%)	1 (0.1%)	3.93 (0.24, 63.00)	0.33
**20 – 29**	1 (0.2%)	7 (0.4%)	0.56 (0.07, 4.58)	0.59
**30 – 39**	3 (0.6%)	11 (0.6%)	1.07 (0.30, 3.87)	0.92
**40 – 49**	7 (1.5%)	28 (1.5%)	0.98 (0.42, 2.27)	0.97
**50 – 59**	15 (3.1%)	76 (4.0%)	0.78 (0.44, 1.37)	0.38
**60 – 69**	56 (11.7%)	233 (12.1%)	0.94 (0.68, 1.30)	0.73
**70 – 79**	135 (28.1%)	535 (27.9%)	0.99 (0.79, 1.25)	0.94
**≥ 80**	262 (54.6%)	1029 (52.6%)	Referent	-
**IMD, quintile**				
**1, lower SES**	90 (18.8%)	362 (18.9%)	1.33 (0.95, 1.86)	0.09
**2**	114 (23.8%)	376 (19.6%)	1.62 (1.18, 2.24)	0.003
**3**	109 (22.7%)	384 (20.0%)	1.52 (1.10, 2.10)	0.01
**4**	78 (16.3%)	418 (21.8%)	Referent	-
**5, higher SES**	89 (18.5%)	380 (19.8%)	1.26 (0.90, 1.75)	0.18
**Mean prescription history, years (SD)**	2.12 (2.41)	1.99 (2.27)	1.02 (0.98, 1.07) *	0.27
**HbA1c ****				
**1, well controlled**	418 (87.0%)	1490 (77.6%)	Referent	-
**2**	42 (9.0%)	305 (15.9%)	0.49 (0.35, 0.69)	0.001
**3, poorly controlled**	20 (4.0%)	125 (6.5%)	0.57 (0.35, 0.93)	0.02
**Missing (coded as 1)**	338 (70.4%)	979 (51.0%)	-	-

For peer review only - http://bmjopen.bmj.com/site/about/guidelines.xhtml

There was little evidence that any exposure to fibrates was associated with brain tumour risk for either primary (adjusted OR [aOR] 0.99, 95% CI: 0.75, 1.30; P = 0.94), secondary (aOR 0.91, 95% CI: 0.67, 1.23; P = 0.55) or all tumours (aOR 0.98, 95% CI: 0.78, 1.24; P = 0.88) (**Table 2**). We found little evidence for a dose-response relationship of fibrate exposure, either as longest or total duration, with brain tumour risk for primary, secondary or all tumours (aORs varied from 0.95 to 1.01 and all consistent with chance). The sensitivity analysis, which only classified exposed if this was the first prescribed anti-hyperlipidaemia medication, found similar results but with less precision (aOR = 0.92, 95% CI: 0.69, 1.22; P = 0.54)

There was very weak evidence of an inverse association between ever exposure to glitazones (aOR = 0.80, 95% CI: 0.65, 1.07; P = 0.14) and all tumours, which was stronger with secondary tumours (aOR = 0.68, 95% CI: 0.46, 1.02; P = 0.06) rather than primary tumours (aOR = 0.96, 95% CI 0.69, 1.33; P = 0.81) (**Table 3**), though this could have been due to chance. The inverse association was stronger and consistent across both primary and secondary tumours when the exposure was classified as longest duration; primary (aOR per year 0.89, 95% CI: 0.80, 0.98; P = 0.02), secondary (aOR per year 0.87, 95% CI: 0.77, 0.99; P = 0.02) and all brain tumours (aOR per year 0.88, 95% CI: 0.81, 0.95; P = 0.002). Very similar results were seen with total duration of exposure (e.g. aOR per year for all tumours 0.85, 95% CI: 0.79, 0.92; P = 8.00×10^{-5}).

For peer review only - http://bmjopen.bmj.com/site/about/guidelines.xhtml

272 **Table 2: Case-control results for primary, secondary and all brain tumour cases with fibrate exposure.**

Fibrates												
	Primary brain tumours				Secondary brain tumours				All brain tumours			
**Variable**	**Cases (%)**	**Controls (%)**	**Adjusted OR (95% CI) ***	**P**	**Cases (%)**	**Controls (%)**	**Adjusted OR (95% CI) ***	**P**	**Cases (%)**	**Controls (%)**	**Adjusted OR (95% CI) ***	**P**
	N = 1094	N = 4375			N = 856	N = 3416			N = 1950	N = 7791		
**Fibrate exposure status**												
**Exposed**	68 (6.2%)	278 (6.4%)	0.99 (0.75, 1.30)	0.94	56 (6.5%)	245 (7.2%)	0.91 (0.67, 1.23)	0.55	124 (6.4%)	523 (6.7%)	0.98 (0.78, 1.24)	0.88
**Unexposed**	1026 (93.8%)	4097 (93.7%)	Referent	-	800 (93.5%)	3171 (92.8%)	Referent	-	1826 (93.6%)	7268 (93.3%)	Referent	-
**Fibrate longest duration, days (SD) ****	799 (1050)	755 (913)	1.01 (0.94, 1.09)	0.76	676 (913)	878 (1123)	0.95 (0.87, 1.03)	0.16	743 (989)	813 (1018)	0.97 (0.91, 1.03)	0.35
**Fibrate total duration, days (SD) ****	969 (1183)	1073 (1233)	0.99 (0.93, 1.05)	0.64	871 (1232)	1217 (1408)	0.95 (0.88, 1.01)	0.08	925 (1202)	1141 (1319)	0.97 (0.92, 1.01)	0.11

273

274 * Model adjusted for sex, age, IMD, retrospective prescription history (categorised yearly). ** OR calculated for per year increase in variable.

275

For peer review only - http://bmjopen.bmj.com/site/about/guidelines.xhtml

Table 3: Case-control results for primary, secondary and all brain tumour cases with glitazone exposure.

	Primary brain tumours				Secondary brain tumours				All brain tumours			
**Variable**	**Cases (%)**	**Controls (%)**	**Adjusted OR (95% CI) ***	**P**	**Cases (%)**	**Controls (%)**	**Adjusted OR (95% CI) ***	**P**	**Cases (%)**	**Controls (%)**	**Adjusted OR (95% CI) ***	**P**
	N = 278	N = 1112			N = 202	N = 808			N = 480	N = 1920		
**Glitazone exposure status**												
**Exposed**	61 (21.9%)	261 (23.5%)	0.96 (0.69, 1.33)	0.81	36 (17.8%)	199 (24.6%)	0.68 (0.46, 1.02)	0.06	97 (20.2%)	460 (24.0%)	0.80 (0.65, 1.07)	0.14
**Unexposed**	217 (78.1%)	851 (76.5%)	Referent	-	166 (82.2%)	609 (75.4%)	Referent	-	383 (79.8%)	1460 (76.0%)	Referent	-
**Glitazone longest duration, days (SD) ****	551 (599)	851 (754)	0.89 (0.80, 0.98)	0.02	701 (550)	899 (767)	0.87 (0.77, 0.99)	0.02	607 (583)	872 (760)	0.88 (0.81, 0.95)	0.002
**Glitazone total duration, days (SD) ****	684 (695)	1000 (809)	0.90 (0.82, 0.99)	0.02	845 (681)	1073 (842)	0.88 (0.79, 0.98)	0.01	603 (720)	1002 (823)	0.86 (0.79, 0.92)	0.00008

* Model adjusted for sex, age, IMD, retrospective prescription history (categorised yearly) and mean HbA1c levels. ** OR calculated for per year increase in variable.

For peer review only - http://bmjopen.bmj.com/site/about/guidelines.xhtml

Sensitivity analyses

Results from the analysis of duration period as a categorical, instead of a continuous variable, found that the inverse association with glitazone and all tumours only emerged after 3 years of exposure; however, the confidence intervals for the shorter duration periods were sufficiently wide as to not exclude any dose-response effect across time (see **Supplemental table 4**). When we only classified exposed if this was the first prescribed anti-diabetes medication, we found little evidence of an association but with very wide confidence intervals due to fewer exposed cases and controls (aOR = 1.10, 95% CI: 0.35, 3.44; P = 0.87) (see **Supplemental table 4**). Effects were similar (longest duration aOR = 0.85, 95% CI 0.78, 0.93; P = $4.00x10^{-4}$) when investigating the effects of monotherapy

Investigating Missing HbA1c levels

HbA1c results were missing for 54.8% of subjects in the glitazone analysis. We observed similar results for the association of both glitazone exposure status (aOR = 0.81, 95% CI: 0.66, 1.00; P = 0.11) and longest duration of glitazone exposure (aOR per year = 0.87, 95% CI: 0.82, 0.93; P = 0.001) with all brain tumour risk when multiple imputation was used for the missing HbA1c values (**Supplemental table 5**). There was little evidence of an association between poorly versus well-controlled HbA1c and case-control status in both the imputed (aOR = 0.95, 95% CI: 0.69, 1.32; P = 0.80) and complete case analysis (aOR = 1.02, 95% CI: 0.60, 1.73; P = 0.80), suggesting that HbA1C may not be a confounder as it did not predict case control status and therefore adjustment may not be necessary. This observation was supported by the aOR without adjustment for HbA1c which was almost identical to the imputed results so that the odds ratio per year of glitazaone exposure with longest duration was 0.87 (95% CI: 0.80, 0.94; P = 0.0008) (**Supplemental table 5**).

Latency Period

Similar associations of both fibrates and glitazones with all brain tumour risk were observed in the sensitivity analyses with different latency periods. In the glitazone analysis, the inverse association seen in the initial analyses for longest duration was very similar as the latency period increased (aOR after a 6-, 12- and 24-month latency were 0.88, 0.87, 0.83 respectively) and unlikely to be due to chance (**Supplemental table 6**).

Discussion

To the best of our knowledge, this is the first pharmacoepidemiological study of the effects of fibrate and glitazone prescription on risk of brain tumours compared to other treatments for

For peer review only - http://bmjopen.bmj.com/site/about/guidelines.xhtml

hyperlipidaemia and type 2 diabetes. There have been various *in vivo* studies that have suggested fibrate exposure may be protective for brain tumours, specifically gliomas, by modulating PPAR-α inhibition [23-26]. There is similar evidence available for the consideration of glitazones as a treatment option from *in vivo* studies [27-30]. However, prior to this study, there have been no other pharmacoepidemiological studies that examine whether fibrates or glitazones affect brain tumour risk.

In the glitazone case-control analysis, there was a modest inverse effect observed for ever exposure to glitazones, which was seen more strongly in the dose-response analyses, especially after exposure durations of 4 or more years, in both univariable and multivariable models. Repeating our analyses and accounting for various latency periods did not alter our conclusions. Similar inverse associations were seen for both primary and secondary brain tumours, but limited data meant analyses could not test whether there were differences by type of primary cancer for those subjects with secondary brain tumours.

We found little evidence that exposure to fibrates was associated with all, primary or secondary brain tumour risk, in contrast to some previous literature[31]. Sensitivity analyses, investigating a potential dose-response effect, or exposure status based on whether fibrates were the first prescribed drug for treatment, also showed little evidence of an association between fibrate exposure and all brain tumour risk.

The assumption that missing HbA1c levels should be assumed to reflect good glycaemic control was tested using follow-up sensitivity analyses to ensure this did not bias the potential negative association between glitazone exposure and brain tumour risk. However, in both the multiple imputation and complete case analysis we found no association between HbA1c and case control status. Furthermore, the results using both multiple imputation or no adjustment were consistent with the primary analyses.

This study has several strengths and limitations. We used a nested case control design within CPRD, a large and well-established database which has been validated by numerous sources [15, 16, 32]. It should be free of selection bias as almost all UK residents are registered with a GP and the population captured by CPRD-registered practices is representative of the general population [14]. Exposure was well measured as it was recorded from medical systems that used the record to print out prescribed drugs for redemption at a pharmacist. The exposure data were collected prospectively, prior to diagnosis, which will avoid recall bias. However, we have only measured receipt of a prescription and have no data on actual drug intake or

For peer review only

For peer review only - http://bmjopen.bmj.com/site/about/guidelines.xhtml

therapeutic levels. Despite this, as both the response to therapy are monitored the dosage is likely to be adjusted in response to good or bad control of the subjects' lipids and blood sugar levels. Exposure was classified from first prescription of any drug for lipids or diabetes and controls were sampled to have similar total prescription duration to avoid time-window bias, which was also adjusted in the multivariable models. The risk of confounding by indication was minimized by only sampling cases and controls who could have potentially been exposed to fibrates or glitazones because of a clinical indication. However, the consequence of this sampling strategy is that the number of available cases was markedly reduced with reduced statistical power to detect modest effects. Not all potential risk factors for brain tumours were captured by CPRD (e.g. previous irradiation) but if these are not associated with exposure this will not cofound the results. Adjustment for SES was at an ecological rather than individual level but if anything is likely to bias the associations to the null due to non-differential measurement error. We had to combine all secondary brain cancers regardless of the primary cancer. Many of the secondary brain tumour cases had a previous diagnosis of common cancers which metastasise to brain, in particular breast, lung and skin cancers.

Overall longer glitazone exposure was associated with a reduced risk of brain metastasises from these primary sites but larger studies should be conducted to determine whether the association of glitazone exposure and secondary brain tumours is similar or not across these different primary sites. Risk of brain tumours may be greater in populations with either type 2 diabetes or hyperinsulinaemia [33, 34] though further studies are required to confirm these associations. We cannot say if we would have observed the same associations if glitazones were prescribed as a preventative therapy in a high-risk non-diabetic population as the generalizability of our results are limited to those brain tumour cases with type 2 diabetes.

Conclusions

Longer exposure to glitazones was inversely associated with diagnosis of primary and secondary brain tumours, but we found little evidence of an association between fibrates and any type of brain tumour. Our results should be generalizable to other high income settings but may not be generalizable to low middle income populations. Further research is required to investigate whether these findings are replicated using independent datasets, preferably larger in size and/or with better data on glycaemic control and other potential confounders. If the glitazone association is biologically causal, this may lead to a better understanding of pathophysiological mechanisms and potential therapies for the prevention of brain cancers. For example, patients with specific aggressive cancers that have a high probability of brain

For peer review only - http://bmjopen.bmj.com/site/about/guidelines.xhtml

metastases, could be treated with glitazones as a tertiary prevention strategy to prevent or delay secondary spread to the brain. Such a hypothesis could be tested in a future double blind clinical trial if stronger evidence emerges from other studies, given the safety and current use of glitazones for the management of diabetes.

Ethics Approval

The study was approved by the Independent Scientific Advisory Committee (ISAC) for the UK Medicines and Healthcare Products Regulatory Agency (ISAC protocol number: 18_149R). Participant consent is not required for ISAC approved CPRD studies using purely observational data. We assert that all procedures contributing to this work comply with the ethical standards of the relevant national and institutional committees on human experimentation and with the Helsinki Declaration of 1975, as revised in 2008.

Availability of Data and Materials

The Clinical Practice Research Datalink (CPRD) is an electronic healthcare record database open to all researchers. Researchers can apply to access CPRD data, and, if successful, can access the data of their choosing. The CPRD charges researchers and other organisations to access this data. The data that support the findings of this study are available from the Clinical Practice Research Datalink (CPRD) but restrictions apply to the availability of these data, which were used under license for the current study, and so are not publicly available.

Competing Interests

JWR receives funding from Biogen for unrelated research. All other authors declare no conflicts of interest.

Funding

The Medical Research Council (MRC)/University of Bristol Integrative Epidemiology Unit (IEU) is supported by the MRC and the University of Bristol (MC_UU_12013/1, MC_UU_12013/2). JWR is supported by a joint studentship from NHS Southmead Hospital Charity (SOCS/SJ1447). RMM is supported by a Cancer Research UK Programme Grant, the Integrative Cancer Epidemiology Programme (C18281/A29019). JWR and RMM are members of the MRC IEU which is supported by the Medical Research Council and the University of Bristol (MC_UU_12013/1-9). RMM is supported by the National Institute for Health Research (NIHR) Bristol Biomedical Research Centre which is funded by the

For peer review only

National Institute for Health Research and is a partnership between University Hospitals Bristol NHS Trust and Weston NHS Foundation Trust and the University of Bristol. RMM is a National Institute for Health Research Senior Investigator (NIHR202411). The views expressed in this publication are those of the author(s) and not necessarily those of the NIHR or the UK Department of Health and Social Care. This work was supported by funding from the Brain Tumour Bank and Research Fund, Southmead Hospital Charity (Registered Charity Number: 1055900), and University of Bristol Campaigns and Alumni funding. This work was supported by CRUK [grant number C18281/A29019], as part of the Integrative Cancer Epidemiology Programme. MTR, MMCE, and YBS are supported by the NIHR Applied Research Collaboration West (NIHR ARC West). The views expressed in this article are those of the author(s) and not necessarily those of the NIHR or the Department of Health and Social Care.

Author's Contributions

Study hypothesis and design: KMK, RMM, YBS. Extraction of CPRD data: MMCE, MTR. Conceptualisation of analysis plan: RMM, YBS, KMK, MC, MTR Performed data management and statistical analyses: JWR, MC. Writing of the initial version of the manuscript: JWR. All authors read, reviewed and provided feedback for final manuscript.

For peer review only

References

1. **Brain, other CNS and intracranial tumours statistics** [https://www.cancerresearchuk.org/health-professional/cancer-statistics/statistics-by-cancer-type/brain-other-cns-and-intracranial-tumours]

2. Kitamura S, Miyazaki Y, Shinomura Y, Kondo S, Kanayama S, Matsuzawa Y: **Peroxisome proliferator-activated receptor gamma induces growth arrest and differentiation markers of human colon cancer cells**. *Jpn J Cancer Res* 1999, **90**(1):75-80.

3. Poulsen L, Siersbaek M, Mandrup S: **PPARs: fatty acid sensors controlling metabolism**. *Semin Cell Dev Biol* 2012, **23**(6):631-639.

4. Rosen ED, Spiegelman BM: **PPARgamma : a nuclear regulator of metabolism, differentiation, and cell growth**. *J Biol Chem* 2001, **276**(41):37731-37734.

5. Barak Y, Liao D, He W, Ong ES, Nelson MC, Olefsky JM, Boland R, Evans RM: **Effects of peroxisome proliferator-activated receptor delta on placentation, adiposity, and colorectal cancer**. *Proc Natl Acad Sci U S A* 2002, **99**(1):303-308.

6. Demetri GD, Fletcher CD, Mueller E, Sarraf P, Naujoks R, Campbell N, Spiegelman BM, Singer S: **Induction of solid tumor differentiation by the peroxisome proliferator-activated receptor-gamma ligand troglitazone in patients with liposarcoma**. *Proc Natl Acad Sci U S A* 1999, **96**(7):3951-3956.

7. Mueller E, Smith M, Sarraf P, Kroll T, Aiyer A, Kaufman DS, Oh W, Demetri G, Figg WD, Zhou XP *et al*: **Effects of ligand activation of peroxisome proliferator-activated receptor gamma in human prostate cancer**. *Proc Natl Acad Sci U S A* 2000, **97**(20):10990-10995.

8. Smallridge RC, Copland JA, Brose MS, Wadsworth JT, Houvras Y, Menefee ME, Bible KC, Shah MH, Gramza AW, Klopper JP *et al*: **Efatutazone, an oral PPAR-gamma agonist, in combination with paclitaxel in anaplastic thyroid cancer: results of a multicenter phase 1 trial**. *J Clin Endocrinol Metab* 2013, **98**(6):2392-2400.

9. Kliewer SA, Sundseth SS, Jones SA, Brown PJ, Wisely GB, Koble CS, Devchand P, Wahli W, Willson TM, Lenhard JM *et al*: **Fatty acids and eicosanoids regulate gene expression through direct interactions with peroxisome proliferator-activated receptors alpha and gamma**. *Proc Natl Acad Sci U S A* 1997, **94**(9):4318-4323.

10. Varga T, Czimmerer Z, Nagy L: **PPARs are a unique set of fatty acid regulated transcription factors controlling both lipid metabolism and inflammation**. *Biochim Biophys Acta* 2011, **1812**(8):1007-1022.

11. Grommes C, Karlo JC, Caprariello A, Blankenship D, Dechant A, Landreth GE: **The PPARgamma agonist pioglitazone crosses the blood-brain barrier and reduces tumor growth in a human xenograft model**. *Cancer Chemother Pharmacol* 2013, **71**(4):929-936.

12. Deplanque D, Gelé P, Pétrault O, Six I, Furman C, Bouly M, Nion S, Dupuis B, Leys D, Fruchart J-C *et al*: **Peroxisome Proliferator-Activated Receptor-α Activation as a Mechanism of Preventive Neuroprotection Induced by Chronic Fenofibrate Treatment**. *The Journal of Neuroscience* 2003, **23**(15):6264-6271.

13. Youssef J, Badr M: **Peroxisome proliferator-activated receptors and cancer: challenges and opportunities**. *Br J Pharmacol* 2011, **164**(1):68-82.

14. Herrett E, Gallagher AM, Bhaskaran K, Forbes H, Mathur R, van Staa T, Smeeth L: **Data Resource Profile: Clinical Practice Research Datalink (CPRD)**. *Int J Epidemiol* 2015, **44**(3):827-836.

15. Herrett E, Thomas SL, Schoonen WM, Smeeth L, Hall AJ: **Validation and validity of diagnoses in the General Practice Research Database: a systematic review**. *Br J Clin Pharmacol* 2010, **69**(1):4-14.

16. Herrett E, Shah AD, Boggon R, Denaxas S, Smeeth L, van Staa T, Timmis A, Hemingway H: **Completeness and diagnostic validity of recording acute myocardial infarction events in primary care, hospital care, disease registry, and national mortality records: cohort study**. *BMJ* 2013, **346**:f2350.

For peer review only - http://bmjopen.bmj.com/site/about/guidelines.xhtml

17. Louis DN, Ohgaki H, Wiestler OD, Cavenee WK, Burger PC, Jouvet A, Scheithauer BW, Kleihues P: **The 2007 WHO classification of tumours of the central nervous system**. *Acta Neuropathol* 2007, **114**(2):97-109.
18. Suissa S, Dell'aniello S, Vahey S, Renoux C: **Time-window bias in case-control studies: statins and lung cancer**. *Epidemiology* 2011, **22**(2):228-231.
19. Levesque LE, Hanley JA, Kezouh A, Suissa S: **Problem of immortal time bias in cohort studies: example using statins for preventing progression of diabetes**. *BMJ* 2010, **340**:b5087.
20. Government DoCaL: **The English Indices of Deprivation 2015 Statistical Release**. In. Edited by Government DoCaL; 2015.
21. Pearce N: **Analysis of matched case-control studies**. *BMJ* 2016, **352**:i969.
22. Buuren Sv, Groothuis-Oudshoorn K: **mice: Multivariate Imputation by Chained Equations inR**. *Journal of Statistical Software* 2011, **45**(3).
23. Haynes HR, White P, Hares KM, Redondo J, Kemp KC, Singleton WGB, Killick-Cole CL, Stevens JR, Garadi K, Guglani S *et al*: **The transcription factor PPARalpha is overexpressed and is associated with a favourable prognosis in IDH-wildtype primary glioblastoma**. *Histopathology* 2017, **70**(7):1030-1043.
24. Binello E, Mormone E, Emdad L, Kothari H, Germano IM: **Characterization of fenofibrate-mediated anti-proliferative pro-apoptotic effects on high-grade gliomas and anti-invasive effects on glioma stem cells**. *J Neurooncol* 2014, **117**(2):225-234.
25. Wilk A, Urbanska K, Grabacka M, Mullinax J, Marcinkiewicz C, Impastato D, Estrada JJ, Reiss K: **Fenofibrate-induced nuclear translocation of FoxO3A triggers Bim-mediated apoptosis in glioblastoma cells in vitro**. *Cell Cycle* 2012, **11**(14):2660-2671.
26. Wilk A, Wyczechowska D, Zapata A, Dean M, Mullinax J, Marrero L, Parsons C, Peruzzi F, Culicchia F, Ochoa A *et al*: **Molecular mechanisms of fenofibrate-induced metabolic catastrophe and glioblastoma cell death**. *Mol Cell Biol* 2015, **35**(1):182-198.
27. Grommes C, Conway DS, Alshekhlee A, Barnholtz-Sloan JS: **Inverse association of PPARgamma agonists use and high grade glioma development**. *J Neurooncol* 2010, **100**(2):233-239.
28. Grommes C, Landreth GE, Sastre M, Beck M, Feinstein DL, Jacobs AH, Schlegel U, Heneka MT: **Inhibition of in Vivo Glioma Growth and Invasion by Peroxisome Proliferator-Activated Receptor γ Agonist Treatment**. *Molecular Pharmacology* 2006, **70**(5):1524-1533.
29. Perez-Ortiz JM, Tranque P, Vaquero CF, Domingo B, Molina F, Calvo S, Jordan J, Cena V, Llopis J: **Glitazones differentially regulate primary astrocyte and glioma cell survival. Involvement of reactive oxygen species and peroxisome proliferator-activated receptor-gamma**. *J Biol Chem* 2004, **279**(10):8976-8985.
30. Perez-Ortiz JM, Tranque P, Burgos M, Vaquero CF, Llopis J: **Glitazones induce astroglioma cell death by releasing reactive oxygen species from mitochondria: modulation of cytotoxicity by nitric oxide**. *Mol Pharmacol* 2007, **72**(2):407-417.
31. Panigrahy D, Kaipainen A, Huang S, Butterfield CE, Barnes CM, Fannon M, Laforme AM, Chaponis DM, Folkman J, Kieran MW: **PPARalpha agonist fenofibrate suppresses tumor growth through direct and indirect angiogenesis inhibition**. *Proc Natl Acad Sci U S A* 2008, **105**(3):985-990.
32. Jick SS, Kaye JA, Vasilakis-Scaramozza C, Garcia Rodriguez LA, Ruigomez A, Meier CR, Schlienger RG, Black C, Jick H: **Validity of the general practice research database**. *Pharmacotherapy* 2003, **23**(5):686-689.
33. Tong JJ, Tao H, Hui OT, Jian C: **Diabetes mellitus and risk of brain tumors: A meta-analysis**. *Exp Ther Med* 2012, **4**(5):877-882.
34. Alexandru O, Ene L, Purcaru OS, Tache DE, Popescu A, Neamtu OM, Tataranu LG, Georgescu AM, Tudorica V, Zaharia C *et al*: **Plasma levels of glucose and insulin in patients with brain tumors**. *Curr Health Sci J* 2014, **40**(1):27-36.

For peer review only

For peer review only - http://bmjopen.bmj.com/site/about/guidelines.xhtml

Read Code	Description	CPRD Medical Code	CPRD Description
B506.00	Malignant neoplasm of choroid	15991	Malignant neoplasm of choroid
B51..00	Malignant neoplasm of brain	18617	Malignant neoplasm of brain
B51..11	Cerebral tumour - malignant	10851	Cerebral tumour - malignant
B510.00	Malignant neoplasm cerebrum (excluding lobes and ventricles)	15711	Malignant neoplasm cerebrum (excluding lobes and ventricles)
B510000	Malignant neoplasm of basal ganglia	48073	Malignant neoplasm of basal ganglia
B510100	Malignant neoplasm of cerebral cortex	61399	Malignant neoplasm of cerebral cortex
B510300		99913	Malignant neoplasm of globus pallidus
B510400	Malignant neoplasm of hypothalamus	70942	Malignant neoplasm of hypothalamus
B510500	Malignant neoplasm of thalamus	62126	Malignant neoplasm of thalamus
B510z00	Malignant neoplasm of cerebrum NOS	54133	Malignant neoplasm of cerebrum NOS
B511.00	Malignant neoplasm of frontal lobe	42426	Malignant neoplasm of frontal lobe
B512.00	Malignant neoplasm of temporal lobe	46792	Malignant neoplasm of temporal lobe
B512000	Malignant neoplasm of hippocampus	67236	Malignant neoplasm of hippocampus
B512z00	Malignant neoplasm of temporal lobe NOS	47556	Malignant neoplasm of temporal lobe NOS
B513.00	Malignant neoplasm of parietal lobe	19226	Malignant neoplasm of parietal lobe
B514.00	Malignant neoplasm of occipital lobe	39088	Malignant neoplasm of occipital lobe
B515.00	Malignant neoplasm of cerebral ventricles	52511	Malignant neoplasm of cerebral ventricles
B515000	Malignant neoplasm of choroid plexus	46789	Malignant neoplasm of choroid plexus
B516.00	Malignant neoplasm of cerebellum	45154	Malignant neoplasm of cerebellum
B517.00	Malignant neoplasm of brain stem	44089	Malignant neoplasm of brain stem
B517000	Malignant neoplasm of cerebral peduncle	64557	Malignant neoplasm of cerebral peduncle
B517100	Malignant neoplasm of medulla oblongata	49132	Malignant neoplasm of medulla oblongata
B517200	Malignant neoplasm of midbrain	93537	Malignant neoplasm of midbrain
B517300	Malignant neoplasm of pons	91240	Malignant neoplasm of pons
B517z00	Malignant neoplasm of brain stem NOS	68641	Malignant neoplasm of brain stem NOS
B51y.00	Malignant neoplasm of other parts of brain	71139	Malignant neoplasm of other parts of brain
B51y000	Malignant neoplasm of corpus callosum	59170	Malignant neoplasm of corpus callosum
B51y200	Malignant neoplasm, overlapping lesion of brain	65241	Malignant neoplasm, overlapping lesion of brain
B51yz00		100733	Malignant neoplasm of other part of brain NOS
B51z.00	Malignant neoplasm of brain NOS	41520	Malignant neoplasm of brain NOS
B521.00	Malignant neoplasm of cerebral meninges	28919	Malignant neoplasm of cerebral meninges
B521200		109473	Malignant neoplasm of cerebral pia mater
B521z00	Malignant neoplasm of cerebral meninges NOS	70104	Malignant neoplasm of cerebral meninges NOS
B52X.00	Malignant neoplasm of meninges, unspecified	49875	Malignant neoplasm of meninges, unspecified

For peer review only

B542.00	Malignant neoplasm pituitary gland and craniopharyngeal duct	59823	Malignant neoplasm pituitary gland and craniopharyngeal duct
B542000	Malignant neoplasm of pituitary gland	8550	Malignant neoplasm of pituitary gland
B542z00	Malig neop pituitary gland or craniopharyngeal duct NOS	59718	Malig neop pituitary gland or craniopharyngeal duct NOS
B543.00	Malignant neoplasm of pineal gland	42460	Malignant neoplasm of pineal gland
B583.00	Secondary malignant neoplasm of brain and spinal cord	33843	Secondary malignant neoplasm of brain and spinal cord
B583000	Secondary malignant neoplasm of brain	5198	Secondary malignant neoplasm of brain
B583200	Cerebral metastasis	5199	Cerebral metastasis
B583z00	Secondary malignant neoplasm of brain or spinal cord NOS	59375	Secondary malignant neoplasm of brain or spinal cord NOS
B8yy300	Carcinoma in situ of pituitary gland	45909	Carcinoma in situ of pituitary gland
BA06.00	Neoplasm of unspecified nature of brain	1044	Neoplasm of unspecified nature of brain
BB9G.00	[M]Infiltrating ductular carcinoma	7319	[M]Infiltrating ductular carcinoma
BBa3.00	[M]Pineoblastoma	50151	[M]Pineoblastoma
BBb..00	[M]Gliomas	12309	[M]Gliomas
BBb0.00	[M]Glioma, malignant	31574	[M]Glioma, malignant
BBb0.11	[M]Glioma NOS	8523	[M]Glioma NOS
BBb0.12	[M]Gliosarcoma	34252	[M]Gliosarcoma
BBb1.00	[M]Gliomatosis cerebri	38551	[M]Gliomatosis cerebri
BBb2.00	[M]Mixed glioma	68808	[M]Mixed glioma
BBb2.11	[M]Mixed glioma	39386	[M]Mixed glioma
BBb3.00	[M]Subependymal glioma	94267	[M]Subependymal glioma
BBb3.11	[M]Subependymal astrocytoma NOS	90487	[M]Subependymal astrocytoma NOS
BBb3.12	[M]Subependymal astrocytoma NOS	28344	[M]Subependymal astrocytoma NOS
BBb4.00	[M]Subependymal giant cell astrocytoma	49168	[M]Subependymal giant cell astrocytoma
BBb8.00	[M]Ependymoma, anaplastic type	52751	[M]Ependymoma, anaplastic type
BBb8.11	[M]Ependymoblastoma	46769	[M]Ependymoblastoma
BBbA.00	[M]Myxopapillary ependymoma	43114	[M]Myxopapillary ependymoma
BBba.00	[M]Primitive neuroectodermal tumour	41695	[M]Primitive neuroectodermal tumour
BBbB.00	[M]Astrocytoma NOS	8547	[M]Astrocytoma NOS
BBbB.11	[M]Astrocytic glioma	27748	[M]Astrocytic glioma
BBbC.00	[M]Astrocytoma, anaplastic type	8328	[M]Astrocytoma, anaplastic type
BBbE.00	[M]Gemistocytic astrocytoma	45531	[M]Gemistocytic astrocytoma
BBbF.00	[M]Fibrillary astrocytoma	27846	[M]Fibrillary astrocytoma
BBbG.00	[M]Pilocytic astrocytoma	30273	[M]Pilocytic astrocytoma
BBbG.11	[M]Juvenile astrocytoma	61783	[M]Juvenile astrocytoma
BBbG.12	[M]Piloid astrocytoma	98800	[M]Piloid astrocytoma
BBbH.00		103047	[M]Spongioblastoma NOS
BBbK.00	[M]Astroblastoma	50235	[M]Astroblastoma
BBbL.00	[M]Glioblastoma NOS	23083	[M]Glioblastoma NOS
BBbL.11	[M]Glioblastoma multiforme	9575	[M]Glioblastoma multiforme
BBbM.00	[M]Giant cell glioblastoma	66064	[M]Giant cell glioblastoma
BBbQ.00	[M]Oligodendroglioma NOS	27744	[M]Oligodendroglioma NOS
BBbR.00	[M]Oligodendroglioma, anaplastic type	49186	[M]Oligodendroglioma, anaplastic type

For peer review only

BBbS.00	[M]Oligodendroblastoma	46404	[M]Oligodendroblastoma
BBbT.00	[M]Medulloblastoma NOS	34763	[M]Medulloblastoma NOS
BBbU.00	[M]Desmoplastic medulloblastoma	65952	[M]Desmoplastic medulloblastoma
BBbV.00	[M]Medullomyoblastoma	31767	[M]Medullomyoblastoma
BBbW.00	[M]Cerebellar sarcoma NOS	37473	[M]Cerebellar sarcoma NOS
BBbZ.00	[M]Pleomorphic xanthoastrocytoma	67587	[M]Pleomorphic xanthoastrocytoma
BBbz.00	[M]Glioma NOS	27653	[M]Glioma NOS
BBc6.00	[M]Ganglioglioma	31629	[M]Ganglioglioma
BBc7.11	[M]Neuroastrocytoma	68479	[M]Neuroastrocytoma
BBd1.00	[M]Meningiomatosis NOS	98677	[M]Meningiomatosis NOS
BBd1.11	[M]Diffuse meningiomatosis	95108	[M]Diffuse meningiomatosis
BBd2.00	[M]Meningioma, malignant	27363	[M]Meningioma, malignant
BBd2.11	[M]Leptomeningeal sarcoma	60347	[M]Leptomeningeal sarcoma
BBd2.12	[M]Meningothelial sarcoma	96798	[M]Meningothelial sarcoma
BBd5.00	[M]Psammomatous meningioma	38870	[M]Psammomatous meningioma
BBd7.11	[M]Angioblastic meningioma	46490	[M]Angioblastic meningioma
BBdB.00		106134	[M]Meningeal sarcomatosis
BBdz.00	[M]Meningioma NOS	47848	[M]Meningioma NOS
BBm0.00	[M]Microglioma	63973	[M]Microglioma
ByuA200	[X]Malignant neoplasm of meninges, unspecified	63925	[X]Malignant neoplasm of meninges, unspecified
ByuA300	[X]Malig neopl, overlap lesion brain & other part of CNS	47633	[X]Malig neopl, overlap lesion brain & other part of CNS

Supplemental table 1 Read and CPRD Medical Codes used to identify primary and secondary brain tumours.

For peer review only

Product Code	Product Name	PPARa Targeting?
25	Simvastatin 20mg tablets	No
28	Atorvastatin 10mg tablets	No
42	Simvastatin 10mg tablets	No
51	Simvastatin 40mg tablets	No
75	Atorvastatin 20mg tablets	No
184	Bezafibrate 200mg tablets	Yes
379	Fluvastatin 20mg capsules	No
490	Pravastatin 10mg tablets	No
602	Bezafibrate 400mg modified-release tablets	Yes
644	Colestyramine 4g oral powder sachets	No
653	Ezetimibe 10mg tablets	No
713	Rosuvastatin 10mg tablets	No
730	Pravastatin 20mg tablets	No
745	Atorvastatin 40mg tablets	No
802	Simvador 40mg tablets (Discovery Pharmaceuticals)	No
818	Simvastatin 20mg/5ml oral solution sugar free	No
1212	Colestipol 5g granules sachets sugar free	No
1214	Bezalip 400mg Tablet (Roche Products Ltd)	Yes
1215	Fenofibrate 100mg Capsule	Yes
1217	Lipantil micro 200 200mg Capsule (Fournier Pharmaceuticals Ltd)	Yes
1219	Pravastatin 40mg tablets	No
1221	Lipostat 10mg tablets (Bristol-Myers Squibb Pharmaceuticals Ltd)	No
1223	Lipostat 40mg tablets (Bristol-Myers Squibb Pharmaceuticals Ltd)	No
1322	Clofibrate 500mg capsules	Yes
1324	Bezalip 200mg Tablet (Roche Products Ltd)	Yes
1477	Atromid -s 500mg Capsule (AstraZeneca UK Ltd)	Yes
1716	Questran 4g oral powder sachets (Bristol-Myers Squibb Pharmaceuticals Ltd)	No
1764	Questran Light 4g oral powder sachets (Bristol-Myers Squibb Pharmaceuticals Ltd)	No
2137	Fluvastatin 40mg capsules	No
2215	Lopid 300mg capsules (Pfizer Ltd)	Yes
2435	Lipantil 100mg Capsule (Fournier Pharmaceuticals Ltd)	Yes
2718	Zocor 10mg tablets (Merck Sharp & Dohme Ltd)	No
2955	Lipitor 40mg tablets (Pfizer Ltd)	No
3089	Ciprofibrate 100mg tablets	Yes
3159	Fenofibrate 200mg capsules	Yes
3318	Gemfibrozil 300mg capsules	Yes
3411	Lipitor 10mg tablets (Pfizer Ltd)	No
3690	Lipostat 20mg tablets (Bristol-Myers Squibb Pharmaceuticals Ltd)	No
4062	Lopid 600mg tablets (Pfizer Ltd)	Yes
4067	Olbetam 250mg capsules (Pfizer Ltd)	No
4920	Fenofibrate micronised 200mg capsules	Yes
4928	Lipantil Micro 200 capsules (BGP Products Ltd)	Yes
5148	Simvastatin 80mg tablets	No
5216	Bezalip mono 400mg Modified-release tablet (Roche Products Ltd)	Yes
5390	Fenofibrate micronised 267mg capsules	Yes
5564	Colestid Orange sachets (Pharmacia Ltd)	No
5775	Atorvastatin 80mg tablets	No
5985	Lescol XL 80mg tablets (Novartis Pharmaceuticals UK Ltd)	No
6120	Ezetrol 10mg tablets (Merck Sharp & Dohme Ltd)	No
6155	Colestyramine with aspartame 4g sugar free powder	No
6168	Zocor 40mg tablets (Merck Sharp & Dohme Ltd)	No
6213	Rosuvastatin 20mg tablets	No

For peer review only

6365	Colestid 5g granules sachets plain (Pfizer Ltd)	No
7196	Zocor 20mg tablets (Merck Sharp & Dohme Ltd)	No
7347	Crestor 10mg tablets (AstraZeneca UK Ltd)	No
7374	Lipitor 20mg tablets (Pfizer Ltd)	No
7540	Lipantil Micro 267 capsules (BGP Products Ltd)	Yes
7544	Niaspan 750mg modified-release tablets (Abbott Laboratories Ltd)	No
7551	Niaspan 1g modified-release tablets (Abbott Laboratories Ltd)	No
7552	Simvastatin 20mg / Ezetimibe 10mg tablets	No
7554	Rosuvastatin 5mg tablets	No
8082	Gemfibrozil 600mg tablets	Yes
8104	Acipimox 250mg capsules	No
8380	Lescol 20mg capsules (Novartis Pharmaceuticals UK Ltd)	No
8706	Modalim 100mg tablets (Sanofi)	Yes
9153	Lescol 40mg capsules (Novartis Pharmaceuticals UK Ltd)	No
9491	Fenofibrate micronised 67mg capsules	Yes
9639	Fenofibrate micronised 160mg tablets	Yes
9716	Supralip 160mg tablets (BGP Products Ltd)	Yes
9897	Rosuvastatin 40mg tablets	No
9920	Simvador 20mg tablets (Discovery Pharmaceuticals)	No
9930	Crestor 40mg tablets (AstraZeneca UK Ltd)	No
10172	Simvastatin 40mg / Ezetimibe 10mg tablets	No
10183	Simvastatin 40mg with ezetimibe 10mg tablet	No
10206	Simvastatin 80mg with ezetimibe 10mg tablet	No
11627	Fluvastatin 80mg modified-release tablets	No
11785	Colestyramine 4g oral powder sachets sugar free	No
11815	Simvastatin 20mg with ezetimibe 10mg tablet	No
11976	Niaspan 500mg modified-release tablets (Abbott Laboratories Ltd)	No
12211	Nicotinic acid 50mg tablets	No
13041	Simvador 10mg tablets (Discovery Pharmaceuticals)	No
14219	Simvastatin 80mg / Ezetimibe 10mg tablets	No
14379	Lipantil Micro 67 capsules (BGP Products Ltd)	Yes
14963	Nicotinic acid 500mg modified-release tablets	No
15252	Crestor 20mg tablets (AstraZeneca UK Ltd)	No
16186	Inegy 10mg/80mg tablets (Merck Sharp & Dohme Ltd)	No
17059	Inegy 10mg/40mg tablets (Merck Sharp & Dohme Ltd)	No
17614	Zimbacol XL 400mg tablets (Archimedes Pharma UK Ltd)	Yes
17683	Lipitor 80mg tablets (Pfizer Ltd)	No
17688	Crestor 5mg tablets (AstraZeneca UK Ltd)	No
17813	Nicotinic acid 100mg Tablet	No
17824	Nicotinic acid 25mg Tablet	No
18081	Colestid Orange 5g granules sachets (Pfizer Ltd)	No
18098	Nicotinic acid 375mg + 500mg + 750mg Modified-release tablet	No
18126	Nicotinic acid 1g modified-release tablets	No
19938	Colestipol with aspartame granules	No
21020	Inegy 10mg/20mg tablets (Merck Sharp & Dohme Ltd)	No
22579	Zocor 80mg tablets (Merck Sharp & Dohme Ltd)	No
23153	Liparol 400 XL tablets (Ashbourne Pharmaceuticals Ltd)	Yes

23153	Liparol 400 XL tablets (Ashbourne Pharmaceuticals Ltd)	No	34316	Simvastatin 20mg tablets (Teva UK Ltd)	No
23634	GEMFIBROZIL	Yes	34353	Simvastatin 40mg tablets (Mylan Ltd)	No
24009	NICOTINIC ACID 500 MG TAB	No	34366	Simvastatin 20mg tablets (IVAX Pharmaceuticals UK Ltd)	No
24084	Colestyramine 4g oral powder sachets sugar free (PLIVA Pharma Ltd)	No	34376	Simvastatin 40mg tablets (Teva UK Ltd)	No
24509	SIMVASTATIN	No	34381	Simvastatin 40mg tablets (IVAX Pharmaceuticals UK Ltd)	No
24583	Nicotinic acid 750mg modified-release tablets	No	34476	Simvastatin 20mg Tablet (Ratiopharm UK Ltd)	No
25018	BEZAFIBRATE	Yes	34481	Simvastatin 10mg tablets (IVAX Pharmaceuticals UK Ltd)	No
29213	Bezagen XL 400mg tablets (Mylan Ltd)	Yes	34502	Simvastatin 40mg tablets (A A H Pharmaceuticals Ltd)	No
29328	Bezafibrate 200mg tablets (A A H Pharmaceuticals Ltd)	Yes	34535	Simvastatin 10mg tablets (Mylan Ltd)	No
29438	SIMVASTATIN	No	34545	Simvastatin 40mg Tablet (Ratiopharm UK Ltd)	No
31221	Bezafibrate 200mg tablets (Mylan Ltd)	Yes	34560	Simvastatin 10mg Tablet (Ratiopharm UK Ltd)	No
31783	Fenogal 200mg capsules (Genus Pharmaceuticals Ltd)	Yes	34746	Simvastatin 20mg Tablet (Niche Generics Ltd)	No
31930	Zocor heart-pro 10mg Tablet (McNeil Products Ltd)	No	34814	Simvastatin 20mg tablets (Wockhardt UK Ltd)	No
32110	Colestyramine 4g Sachets (Dominion Pharma)	No	34820	Pravastatin 40mg tablets (A A H Pharmaceuticals Ltd)	No
32909	Simvastatin 80mg tablets (A A H Pharmaceuticals Ltd)	No	34879	Simvastatin 40mg Tablet (Niche Generics Ltd)	No
32921	Pravastatin 10mg Tablet (Dr Reddy's Laboratories (UK) Ltd)	No	34891	Simvastatin 20mg tablets (Kent Pharmaceuticals Ltd)	No
33082	Simvastatin 20mg tablets (A A H Pharmaceuticals Ltd)	No	34907	Simvastatin 40mg tablets (Wockhardt UK Ltd)	No
33603	Fibrazate XL 400mg tablets (Sandoz Ltd)	Yes	34955	Simvastatin 10mg tablets (A A H Pharmaceuticals Ltd)	No
33944	Bezafibrate 200mg tablets (Teva UK Ltd)	Yes	34969	Simvastatin 40mg tablets (Actavis UK Ltd)	No
34181	Bezafibrate 400mg Modified-release tablet (Hillcross Pharmaceuticals Ltd)	Yes	36377	Pravastatin 20mg tablets (Teva UK Ltd)	No
34201	Colestyramine 4g oral powder sachets sugar free (Actavis UK Ltd)	No	37266	Colesevelam 625mg tablets	No
34277	Gemfibrozil 600mg tablets (Teva UK Ltd)	Yes	37434	Simvastatin 40mg tablets (Sandoz Ltd)	No
34312	Simvastatin 20mg tablets (Mylan Ltd)	No	37953	Cholestagel 625mg tablets (Sanofi)	No

For peer review only

39060	Simvastatin 20mg tablets (Dexcel-Pharma Ltd)	No
39420	Bezalip Mono 400mg modified-release tablets (Teva UK Ltd)	Yes
39576	Bezalip 200mg tablets (Teva UK Ltd)	Yes
39652	Simvastatin 40mg/5ml oral solution sugar free	No
39675	Simvastatin 20mg/5ml Oral suspension (Martindale Pharmaceuticals Ltd)	No
39870	Simvador 80mg tablets (Discovery Pharmaceuticals)	No
40340	Simvastatin 10mg tablets (Teva UK Ltd)	No
40382	Pravastatin 20mg tablets (A A H Pharmaceuticals Ltd)	No
40601	Simvastatin 20mg tablets (Ranbaxy (UK) Ltd)	No
40729	Tredaptive 1000mg/20mg modified-release tablets (Merck Sharp & Dohme Ltd)	No
40885	Nicotinic acid 1g / Laropiprant 20mg modified-release tablets	No
41396	Fenofibrate micronised 200mg capsules (A A H Pharmaceuticals Ltd)	Yes
41657	Simvastatin 80mg tablets (Teva UK Ltd)	No
42801	Bezafibrate xl 400mg Modified-release tablet (Generics (UK) Ltd)	Yes
43218	Pravastatin 10mg tablets (Teva UK Ltd)	No
44528	Simvastatin 20mg/5ml oral suspension sugar free (Rosemont Pharmaceuticals Ltd)	No
44650	Simvastatin 40mg tablets (Dexcel-Pharma Ltd)	No
44878	Ranzolont 10mg tablets (Ranbaxy (UK) Ltd)	No
45219	Simvastatin 40mg tablets (Kent Pharmaceuticals Ltd)	No
45235	Simvastatin 20mg tablets (Sandoz Ltd)	No
45245	Simvastatin 20mg tablets (Actavis UK Ltd)	No
45346	Simvastatin 40mg tablets (Arrow Generics Ltd)	No
46878	Simvastatin 40mg tablets (Almus Pharmaceuticals Ltd)	No
46956	Simvastatin 80mg tablets (Arrow Generics Ltd)	No
47023	Omega-3 fish oil with glycerol Emulsion for infusion	No
47065	Atorvastatin 20mg chewable tablets sugar free	No
47090	Atorvastatin 10mg chewable tablets sugar free	No
47630	Lipitor 20mg chewable tablets (Pfizer Ltd)	No
47721	Lipitor 10mg chewable tablets (Pfizer Ltd)	No
47774	Simvastatin 10mg tablets (Arrow Generics Ltd)	No
47935	Fenofibrate 200mg Capsule (Teva UK Ltd)	Yes
47948	Simvastatin 10mg tablets (Tillomed Laboratories Ltd)	No
47988	Pravastatin 40mg tablets (Mylan Ltd)	No
48018	Simvastatin 20mg tablets (Arrow Generics Ltd)	No
48051	Simvastatin 10mg tablets (Kent Pharmaceuticals Ltd)	No
48058	Simvastatin 10mg tablets (Ranbaxy (UK) Ltd)	No
48078	Simvastatin 10mg tablets (Actavis UK Ltd)	No
48097	Pravastatin 40mg tablets (Teva UK Ltd)	No
48221	Simvastatin 20mg/5ml oral suspension sugar free	No
48346	Atorvastatin 60mg tablets	No
48431	Simvastatin 40mg/5ml oral suspension sugar free	No
48518	Atorvastatin 10mg/5ml oral solution	No
48585	Nicotinic acid 500mg capsules	No
48867	Simvastatin 40mg tablets (Alliance Healthcare (Distribution) Ltd)	No

For peer review only

48973	Atorvastatin 30mg tablets	No
49061	Simvastatin 40mg tablets (Bristol Laboratories Ltd)	No
49062	Simvastatin 20mg tablets (Alliance Healthcare (Distribution) Ltd)	No
49558	Atorvastatin 20mg tablets (A A H Pharmaceuticals Ltd)	No
49587	Simvastatin 80mg tablets (Almus Pharmaceuticals Ltd)	No
49609	Bezafibrate 400mg Modified-release tablet (Sandoz Ltd)	Yes
49751	Atorvastatin 40mg tablets (Alliance Healthcare (Distribution) Ltd)	No
50071	Fenofibrate 160mg Tablet (Teva UK Ltd)	Yes
50236	Atorvastatin 10mg tablets (Zentiva)	No
50272	Atorvastatin 40mg tablets (Pfizer Ltd)	No
50483	Simvastatin 40mg tablets (Relonchem Ltd)	No
50564	Simvastatin 20mg tablets (Relonchem Ltd)	No
50670	Simvastatin 40mg tablets (Aurobindo Pharma Ltd)	No
50703	Simvastatin 40mg tablets (Accord Healthcare Ltd)	No
50754	Simvastatin 20mg tablets (Medreich Plc)	No
50788	Atorvastatin 20mg tablets (Pfizer Ltd)	No
50790	Atorvastatin 20mg tablets (Dexcel-Pharma Ltd)	No
50882	Simvastatin 40mg tablets (Somex Pharma)	No
50925	Pravastatin 10mg tablets (Sigma Pharmaceuticals Plc)	No
50963	Atorvastatin 40mg tablets (Teva UK Ltd)	No
51085	Simvastatin 10mg tablets (Medreich Plc)	No
51134	Atorvastatin 10mg tablets (A A H Pharmaceuticals Ltd)	No
51155	Natures Aid Omega-3 Fish Oil 1000mg softgels capsules (Natures Aid Ltd)	No
51166	Simvastatin 40mg tablets (Medreich Plc)	No
51200	Atorvastatin 40mg tablets (Arrow Generics Ltd)	No
51233	Simvastatin 10mg tablets (Alliance Healthcare (Distribution) Ltd)	No
51359	Atorvastatin 20mg tablets (Arrow Generics Ltd)	No
51483	Simvastatin 20mg tablets (Aurobindo Pharma Ltd)	No
51622	Atorvastatin 20mg tablets (Consilient Health Ltd)	No
51676	Pravastatin 40mg tablets (Medreich Plc)	No
51715	Simvastatin 10mg tablets (Sigma Pharmaceuticals Plc)	No
51822	Natures Aid Omega-3 Fish Oil 500mg capsules (Natures Aid Ltd)	No
51876	Atorvastatin 40mg tablets (Consilient Health Ltd)	No
51890	Pravastatin 20mg tablets (Medreich Plc)	No
52097	Atorvastatin 40mg tablets (Wockhardt UK Ltd)	No
52098	Simvastatin 40mg tablets (Ranbaxy (UK) Ltd)	No
52168	Atorvastatin 20mg tablets (Aspire Pharma Ltd)	No
52211	Atorvastatin 20mg tablets (Actavis UK Ltd)	No
52257	Simvastatin 20mg tablets (Accord Healthcare Ltd)	No
52397	Atorvastatin 40mg tablets (Dr Reddy's Laboratories (UK) Ltd)	No
52398	Atorvastatin 40mg tablets (A A H Pharmaceuticals Ltd)	No
52459	Atorvastatin 80mg tablets (Actavis UK Ltd)	No
52460	Atorvastatin 40mg tablets (Aspire Pharma Ltd)	No
52625	Simvastatin 10mg tablets (Wockhardt UK Ltd)	No
52676	Simvastatin 10mg/5ml oral suspension	No
52755	Pravastatin 20mg tablets (Alliance Healthcare (Distribution) Ltd)	No

52812	Simvastatin 20mg tablets (Sigma Pharmaceuticals Plc)	No	54435	Pravastatin 40mg tablets (Almus Pharmaceuticals Ltd)	No
52814	Bezafibrate 400mg modified-release tablets (Alliance Healthcare (Distribution) Ltd)	Yes	54493	Simvastatin 10mg tablets (Relonchem Ltd)	No
			54535	Atorvastatin 10mg tablets (Pfizer Ltd)	No
52821	Atorvastatin 80mg tablets (Dr Reddy's Laboratories (UK) Ltd)	No	54606	Simvastatin 20mg/5ml oral suspension sugar free (A A H Pharmaceuticals Ltd)	No
52953	Simvastatin 20mg tablets (Bristol Laboratories Ltd)	No	54607	Pravastatin 20mg tablets (Almus Pharmaceuticals Ltd)	No
52962	Simvastatin 80mg tablets (Medreich Plc)	No			
53087	Simvastatin 20mg tablets (Somex Pharma)	No	54655	Simvastatin 10mg tablets (Accord Healthcare Ltd)	No
53250	Modalim 100mg tablets (Lexon (UK) Ltd)	Yes	54819	Simvastatin 40mg/5ml oral suspension sugar free (Rosemont Pharmaceuticals Ltd)	No
53340	Zocor 40mg tablets (Lexon (UK) Ltd)	No			
53415	Simvastatin 10mg tablets (Aurobindo Pharma Ltd)	No	54947	Simvastatin 20mg tablets (Almus Pharmaceuticals Ltd)	No
53460	Crestor 10mg tablets (DE Pharmaceuticals)	No	54976	Simvastatin 10mg tablets (Somex Pharma)	No
53594	Lipitor 80mg tablets (Mawdsley-Brooks & Company Ltd)	No	54985	Simvastatin 40mg/5ml oral suspension	No
53676	Simvastatin 20mg tablets (Tillomed Laboratories Ltd)	No	54992	Atorvastatin 10mg/5ml oral suspension	No
			55032	Atorvastatin 10mg tablets (Dexcel-Pharma Ltd)	No
53770	Fluvastatin 40mg capsules (A A H Pharmaceuticals Ltd)	No	55034	Atorvastatin 40mg/5ml oral suspension	No
53772	Atorvastatin 80mg tablets (Alliance Healthcare (Distribution) Ltd)	No	55444	Atorvastatin 40mg tablets (Zentiva)	No
			55452	Simvastatin 20mg tablets (Phoenix Healthcare Distribution Ltd)	No
53822	Simvastatin 10mg tablets (Bristol Laboratories Ltd)	No			
53887	Atorvastatin 40mg tablets (Actavis UK Ltd)	No	55727	Atorvastatin 10mg tablets (Actavis UK Ltd)	No
53890	Atorvastatin 80mg tablets (Pfizer Ltd)	No	55912	Pravastatin 40mg tablets (Alliance Healthcare (Distribution) Ltd)	No
53908	Simvastatin 10mg tablets (Dexcel-Pharma Ltd)	No	56065	Simvastatin 20mg/5ml oral suspension sugar free (Waymade Healthcare Plc)	No
53966	Simvastatin 40mg tablets (Phoenix Healthcare Distribution Ltd)	No			
54240	Simvastatin 40mg tablets (Sigma Pharmaceuticals Plc)	No	56097	Atorvastatin 10mg chewable tablets sugar free	No
54266	Simvastatin 20mg/5ml oral suspension	No	56146	Pravastatin 10mg tablets (Waymade Healthcare Plc)	No

For peer review only

56165	Atorvastatin 20mg chewable tablets sugar free	No
56182	Atorvastatin 80mg tablets (Zentiva)	No
56248	Atorvastatin 20mg tablets (Sigma Pharmaceuticals Plc)	No
56481	Zocor 10mg tablets (Sigma Pharmaceuticals Plc)	No
56494	Zocor 20mg tablets (Sigma Pharmaceuticals Plc)	No
56564	Atorvastatin 20mg tablets (Almus Pharmaceuticals Ltd)	No
56607	Pravastatin 20mg tablets (Waymade Healthcare Plc)	No
56735	Pravastatin 20mg tablets (Mylan Ltd)	No
56841	Atorvastatin 40mg tablets (Dexcel-Pharma Ltd)	No
56893	Pravastatin 40mg tablets (Accord Healthcare Ltd)	No
56916	Pravastatin 40mg tablets (PLIVA Pharma Ltd)	No
57108	Pravastatin 40mg tablets (Waymade Healthcare Plc)	No
57117	Atorvastatin 80mg tablets (Waymade Healthcare Plc)	No
57137	Pravastatin 10mg tablets (Almus Pharmaceuticals Ltd)	No
57219	Fenofibrate micronised 200mg capsules (Sandoz Ltd)	Yes
57296	Pravastatin 20mg tablets (Phoenix Healthcare Distribution Ltd)	No
57329	Simvastatin 25mg/5ml oral suspension	No
57348	Atorvastatin 10mg tablets (Consilient Health Ltd)	No
57397	Pravastatin 10mg tablets (Accord Healthcare Ltd)	No
57489	Ciprofibrate 100mg tablets (Zentiva)	Yes
57568	Zocor 10mg tablets (Lexon (UK) Ltd)	No
57763	Rosuvastatin 10mg tablets (Waymade Healthcare Plc)	No
57834	Atorvastatin 40mg tablets (DE Pharmaceuticals)	No
57836	Atorvastatin 80mg tablets (Teva UK Ltd)	No
57999	Crestor 40mg tablets (Lexon (UK) Ltd)	No
58041	Atorvastatin 20mg tablets (Teva UK Ltd)	No
58110	Atorvastatin 20mg tablets (Zentiva)	No
58315	Simvastatin 20mg tablets (Waymade Healthcare Plc)	No
58394	Atorvastatin 20mg tablets (Alliance Healthcare (Distribution) Ltd)	No
58418	Atorvastatin 80mg tablets (A A H Pharmaceuticals Ltd)	No
58617	Rosuvastatin 20mg/5ml oral suspension	No
58635	Bezalip Mono 400mg modified-release tablets (DE Pharmaceuticals)	Yes
58742	Atorvastatin 80mg tablets (Arrow Generics Ltd)	No
58755	Simvastatin 10mg tablets (Phoenix Healthcare Distribution Ltd)	No
58834	Atorvastatin 10mg tablets (DE Pharmaceuticals)	No
58868	Atorvastatin 10mg tablets (Sigma Pharmaceuticals Plc)	No
59002	Bezafibrate 400mg modified-release tablets (DE Pharmaceuticals)	Yes
59272	Atorvastatin 20mg tablets (Waymade Healthcare Plc)	No
59278	Fluvastatin 20mg capsules (Zentiva)	No
59331	Lipitor 10mg tablets (DE Pharmaceuticals)	No
59357	Atorvastatin 10mg tablets (Ranbaxy (UK) Ltd)	No
59446	Atorvastatin 40mg tablets (Almus Pharmaceuticals Ltd)	No

For peer review only

59447	Crestor 20mg tablets (Waymade Healthcare Plc)	No
59452	Rosuvastatin 5mg tablets (Waymade Healthcare Plc)	No
59508	Pravastatin 20mg tablets (Accord Healthcare Ltd)	No
59776	Atorvastatin 80mg tablets (Aspire Pharma Ltd)	No
59859	Atorvastatin 10mg tablets (Teva UK Ltd)	No
60101	Colestyramine 4g oral powder sachets sugar free (Teva UK Ltd)	No
60160	Rosuvastatin 5mg tablets (Mawdsley-Brooks & Company Ltd)	No
60251	Pravastatin 10mg tablets (Sandoz Ltd)	No
60342	Berocca effervescent tablets tropical (Bayer Plc)	No
60385	Bezalip Mono 400mg modified-release tablets (Lexon (UK) Ltd)	Yes
60464	Atorvastatin 20mg/5ml oral suspension	No
60511	Atorvastatin 40mg tablets (Ranbaxy (UK) Ltd)	No
60607	Atorvastatin 80mg tablets (DE Pharmaceuticals)	No
60788	Fenofibrate micronised 267mg capsules (Zentiva)	Yes
60989	Atorvastatin 80mg tablets (Phoenix Healthcare Distribution Ltd)	No
61087	Questran Light 4g oral powder sachets (Mawdsley-Brooks & Company Ltd)	No
61134	Pravastatin 20mg tablets (Sigma Pharmaceuticals Plc)	No
61149	Atorvastatin 10mg tablets (Waymade Healthcare Plc)	No
61155	Simvastatin 40mg/5ml oral suspension sugar free (A A H Pharmaceuticals Ltd)	No
61321	Simvastatin 10mg tablets (Sandoz Ltd)	No
61360	Simvastatin 10mg tablets (Almus Pharmaceuticals Ltd)	No
61665	Simvastatin 10mg tablets (Waymade Healthcare Plc)	No
62137	Simvastatin 40mg tablets (Waymade Healthcare Plc)	No
62148	Fluvastatin 20mg capsules (Actavis UK Ltd)	No
62219	Atorvastatin 20mg tablets (DE Pharmaceuticals)	No
62429	Atorvastatin 20mg tablets (DE Pharmaceuticals)	No
62476	Atorvastatin 80mg tablets (Almus Pharmaceuticals Ltd)	No
62979	Pravastatin 40mg tablets (Kent Pharmaceuticals Ltd)	No
63074	Pravastatin 20mg tablets (PLIVA Pharma Ltd)	No
63140	Atorvastatin 10mg tablets (Alliance Healthcare (Distribution) Ltd)	No
63249	Atorvastatin 80mg tablets (Consilient Health Ltd)	No
63343	Generic Crampex tablets	No
63469	Atorvastatin 30mg tablets (Consilient Health Ltd)	No
63737	Fenofibrate micronised 267mg capsules (Sigma Pharmaceuticals Plc)	Yes
63787	Pravastatin 10mg tablets (Tillomed Laboratories Ltd)	No
64067	Atorvastatin 20mg/5ml oral solution	No
64104	Simvastatin 20mg tablets (Crescent Pharma Ltd)	No
64180	Simvastatin 10mg tablets (Crescent Pharma Ltd)	No
64307	Simvastatin 40mg tablets (Crescent Pharma Ltd)	No
64503	Bezalip Mono 400mg modified-release tablets (Waymade Healthcare Plc)	Yes
64702	Atorvastatin 30mg tablets (A A H Pharmaceuticals Ltd)	No

For peer review only

64810	Atorvastatin 40mg tablets (Phoenix Healthcare Distribution Ltd)	No
64825	Atorvastatin 10mg tablets (Phoenix Healthcare Distribution Ltd)	No
64868	Atorvastatin 40mg tablets (Sigma Pharmaceuticals Plc)	No
64933	Fenofibrate micronised 267mg capsules (Ranbaxy (UK) Ltd)	Yes
64968	Simvastatin 10mg tablets (DE Pharmaceuticals)	No
64984	Fenofibrate micronised 160mg tablets (Phoenix Healthcare Distribution Ltd)	Yes
65181	Simvastatin 40mg tablets (DE Pharmaceuticals)	No
65193	Atorvastatin 20mg tablets (Ranbaxy (UK) Ltd)	No
65572	Fenofibrate micronised 160mg tablets (Genus Pharmaceuticals Ltd)	Yes
65679	Simvastatin 20mg tablets (DE Pharmaceuticals)	No
65901	Simvastatin 40mg tablets (Zentiva)	No
65925	Simvastatin 20mg/5ml oral suspension sugar free (Alliance Healthcare (Distribution) Ltd)	No
66087	Modalim 100mg tablets (Mawdsley-Brooks & Company Ltd)	Yes
66425	Bezafibrate 400mg modified-release tablets (A A H Pharmaceuticals Ltd)	Yes
66505	Fenofibrate 145mg / Simvastatin 40mg tablets	Yes
66564	Bezafibrate 400mg modified-release tablets (Phoenix Healthcare Distribution Ltd)	Yes
66780	Fenofibrate 145mg / Simvastatin 20mg tablets	Yes
66963	Atorvastatin 80mg tablets (Sigma Pharmaceuticals Plc)	No
67098	Simvastatin 10mg tablets (Brown & Burk UK Ltd)	No
67157	Fenofibrate micronised 200mg capsules (Phoenix Healthcare Distribution Ltd)	Yes
67328	Lescol XL 80mg tablets (Mawdsley-Brooks & Company Ltd)	No
67329	Lipantil Micro 267 capsules (DE Pharmaceuticals)	Yes
67402	Atorvastatin 40mg tablets (Kent Pharmaceuticals Ltd)	No
67573	Atorvastatin 10mg tablets (DE Pharmaceuticals)	No
67660	Atorvastatin 80mg tablets (Ranbaxy (UK) Ltd)	No
67745	Simvastatin 10mg tablets (Zentiva)	No
67773	Simvastatin 20mg tablets (Zentiva)	No
67829	Pravastatin 20mg tablets (Sandoz Ltd)	No
67846	Atorvastatin 10mg tablets (Almus Pharmaceuticals Ltd)	No
67883	Lomitapide 5mg capsules	No
68023	Atorvastatin 10mg tablets (Aspire Pharma Ltd)	No
68048	Atorvastatin 20mg tablets (Phoenix Healthcare Distribution Ltd)	No
68156	Pravastatin 10mg tablets (A A H Pharmaceuticals Ltd)	No
68386	Colestyramine 4g oral powder sachets (J M McGill Ltd)	No
68467	Atorvastatin 20mg tablets (Kent Pharmaceuticals Ltd)	No
68563	Simvastatin 40mg tablets (Brown & Burk UK Ltd)	No
68686	Simvastatin 20mg tablets (Genesis Pharmaceuticals Ltd)	No
68785	Atorvastatin 10mg tablets (Mylan Ltd)	No
68827	Atorvastatin 20mg tablets (Mylan Ltd)	No
69093	Atorvastatin 80mg tablets (Wockhardt UK Ltd)	No
69413	Simvastatin 20mg tablets (Brown & Burk UK Ltd)	No

69427	Atorvastatin 40mg tablets (Mylan Ltd)	No

Supplemental table 2: Product codes for drugs used for hyperlipidaemia and fibrate exposure status.

For peer review only

Product Code	Product Name	PPARg Targeting?
23	Metformin 500mg tablets	No
32	Gliclazide 80mg tablets	No
93	Metformin 850mg tablets	No
240	Rifampicin 150mg capsules	No
469	Rosiglitazone 4mg tablets	Yes
479	Acarbose 50mg tablets	No
547	Glipizide 2.5mg tablets	No
548	Pioglitazone 15mg tablets	Yes
735	Metformin 100mg/ml Oral solution	No
1254	Glibenclamide 5mg tablets	No
1964	Diamicron 80mg tablets (Servier Laboratories Ltd)	No
1965	Tolbutamide 500mg tablets	No
2219	Glibenclamide 2.5mg tablets	No
4862	Diabetamide 2.5mg tablets (Ashbourne Pharmaceuticals Ltd)	No
5174	Acarbose 100mg tablets	No
5227	Rosiglitazone 8mg tablets	Yes
5276	Glimepiride 1mg tablets	No
5316	Glimepiride 4mg tablets	No
5353	Glimepiride 2mg tablets	No
5621	Glucobay 50mg tablets (Bayer Plc)	No
5627	Gliclazide 30mg modified-release tablets	No
5636	Glipizide 5mg tablets	No
5678	Nateglinide 120mg tablets	No
5989	Nateglinide 180mg tablets	No
6337	Glimepiride 3mg tablets	No
6855	Avandamet 2mg/500mg tablets (GlaxoSmithKline UK Ltd)	Yes
7048	Metformin 500mg modified-release tablets	No
7166	Glucophage 500mg tablets (Merck Serono Ltd)	No
7284	Amaryl 2mg tablets (Zentiva)	No
7325	Avandamet 4mg/1000mg tablets (GlaxoSmithKline UK Ltd)	Yes
7332	Amaryl 1mg tablets (Zentiva)	No
7375	Rosiglitazone 4mg / Metformin 1g tablets	Yes
7409	Amaryl 3mg tablets (Zentiva)	No
7610	Glucophage 850mg tablets (Merck Serono Ltd)	No
7744	Daonil 5mg tablets (Sanofi)	No
7818	Rifater tablets (Sanofi)	No
7912	Semi-Daonil 2.5mg tablets (Sanofi)	No
8976	Euglucon 2.5mg tablets (Aventis Pharma)	No
9105	Glucobay 100mg tablets (Bayer Plc)	No
9662	Avandia 4mg tablets (GlaxoSmithKline UK Ltd)	Yes
9691	Rifampicin with isoniazid & pyrazinamide tablet	No
9699	Pioglitazone 30mg tablets	Yes
9707	Repaglinide 1mg tablets	No
9748	Repaglinide 2mg tablets	No
9865	Repaglinide 500microgram tablets	No
10051	Pioglitazone 45mg tablets	Yes
11284	Amaryl 4mg tablets (Zentiva)	No
11316	NovoNorm 500microgram tablets (Novo Nordisk Ltd)	No
11321	NovoNorm 1mg tablets (Novo Nordisk Ltd)	No
11366	NovoNorm 2mg tablets (Novo Nordisk Ltd)	No
11483	Nateglinide 60mg tablets	No
11601	Rosiglitazone 2mg / Metformin 500mg tablets	Yes
11604	Rosiglitazone 1mg / Metformin 500mg tablets	Yes
11609	Metformin with rosiglitazone 500mg + 1mg Tablet	Yes
11610	Metformin with rosiglitazone 500mg + 2mg Tablet	Yes
11695	Diamicron 30mg MR tablets (Servier Laboratories Ltd)	No
11717	Rosiglitazone 2mg / Metformin 1g tablets	Yes
11737	Metformin with rosiglitazone 1000mg + 4mg Tablet	Yes
11760	Metformin with rosiglitazone 1000mg + 2mg Tablet	Yes
11946	Tolbutamide 50mg/ml Injection	No
11990	Metformin 500mg/5ml oral solution sugar free	No
12455	Rastinon 500mg Tablet (Hoechst Marion Roussel)	No
12513	Glibenese 5mg tablets (Pfizer Ltd)	No
13331	Euglucon 5mg tablets (Sanofi)	No

For peer review only

For peer review only - http://bmjopen.bmj.com/site/about/guidelines.xhtml

13628	Romozin 400mg Tablet (Glaxo Wellcome UK Ltd)	Yes
14164	Avandamet 2mg/1000mg tablets (GlaxoSmithKline UK Ltd)	Yes
15232	Avandia 8mg tablets (GlaxoSmithKline UK Ltd)	Yes
15374	Gliclazide 40mg/5ml oral suspension	No
15955	Starlix 120mg tablets (Novartis Pharmaceuticals UK Ltd)	No
16044	Glucophage SR 500mg tablets (Merck Serono Ltd)	No
16602	Calabren 2.5mg Tablet (Berk Pharmaceuticals Ltd)	No
17343	Gliclazide 80mg tablets (A A H Pharmaceuticals Ltd)	No
17580	Avandamet 1mg/500mg tablets (GlaxoSmithKline UK Ltd)	Yes
17698	Minodiab 5mg tablets (Pfizer Ltd)	No
17706	Minodiab 2.5mg tablets (Pfizer Ltd)	No
17770	Glucagon novo 10mg Injection (Novo Nordisk Ltd)	No
18220	Pioglitazone 15mg / Metformin 850mg tablets	Yes
19472	Actos 45mg tablets (Takeda UK Ltd)	Yes
20287	Actos 15mg tablets (Takeda UK Ltd)	Yes
20889	Actos 30mg tablets (Takeda UK Ltd)	Yes
21424	Glibenclamide 5mg/5ml oral suspension	No
21564	Gliclazide 80mg tablets (Wockhardt UK Ltd)	No
21832	Diabetamide 5mg tablets (Ashbourne Pharmaceuticals Ltd)	No
21892	Diaglyk 80mg tablets (Ashbourne Pharmaceuticals Ltd)	No
22239	Glucagon lilly 1mg Injection (Eli Lilly and Company Ltd)	No
23945	Starlix 60mg tablets (Novartis Pharmaceuticals UK Ltd)	No
25636	Libanil 2.5mg Tablet (Approved Prescription Services Ltd)	No
25678	Glucamet 500mg Tablet (Opus Pharmaceuticals Ltd)	No
26218	Calabren 5mg Tablet (Berk Pharmaceuticals Ltd)	No
26258	Glucamet 850mg Tablet (Opus Pharmaceuticals Ltd)	No
27125	Starlix 180mg tablets (Novartis Pharmaceuticals UK Ltd)	No
27501	Orabet 500mg Tablet (Lagap)	No
28708	Malix 2.5mg Tablet (Lagap)	No
29326	Glipizide 5mg tablets (Mylan Ltd)	No
29939	Gliclazide 80mg tablets (Mylan Ltd)	No
30316	Metformin with pioglitazone 850mg + 15mg Tablet	Yes
30460	Malix 5mg Tablet (Lagap)	No
31077	Competact 15mg/850mg tablets (Takeda UK Ltd)	Yes
31146	Metsol 500mg/5ml oral solution (Kappin Ltd)	No
31212	Gliclazide 80mg tablets (Actavis UK Ltd)	No
31474	Libanil 5mg Tablet (Approved Prescription Services Ltd)	No
33087	Metformin 500mg tablets (Actavis UK Ltd)	No
33562	Duclazide 80mg Tablet (Dumex Ltd)	No
33673	Tolbutamide 500mg tablets (Actavis UK Ltd)	No
33674	Metformin 850mg tablets (A A H Pharmaceuticals Ltd)	No
34004	Metformin 500mg tablets (IVAX Pharmaceuticals UK Ltd)	No
34020	Metformin 850mg tablets (IVAX Pharmaceuticals UK Ltd)	No
34135	Metformin 500mg Tablet (M & A Pharmachem Ltd)	No
34323	Metformin 500mg tablets (A A H Pharmaceuticals Ltd)	No
34399	Gliclazide 80mg tablets (IVAX Pharmaceuticals UK Ltd)	No
34504	Metformin 500mg tablets (Wockhardt UK Ltd)	No
34507	Glibenclamide 2.5mg tablets (Wockhardt UK Ltd)	No
34563	Glibenclamide 5mg tablets (Wockhardt UK Ltd)	No
34598	Metformin 500mg tablets (Mylan Ltd)	No

For peer review only

34676	Glibenclamide 2.5mg tablets (A A H Pharmaceuticals Ltd)	No
34697	Metformin 850mg tablets (Wockhardt UK Ltd)	No
34706	Glibenclamide 2.5mg tablets (IVAX Pharmaceuticals UK Ltd)	No
34742	Metformin 850mg tablets (Teva UK Ltd)	No
34802	Glipizide 5mg tablets (IVAX Pharmaceuticals UK Ltd)	No
34836	Metformin 850mg tablets (Actavis UK Ltd)	No
34917	Metformin 500mg tablets (Teva UK Ltd)	No
34932	Gliclazide 80mg tablets (Genus Pharmaceuticals Ltd)	No
34957	Tolbutamide 500mg tablets (A A H Pharmaceuticals Ltd)	No
35022	Sitagliptin 100mg tablets	No
35144	Byetta 5micrograms/0.02ml solution for injection 1.2ml pre-filled disposable devices (AstraZeneca UK Ltd)	No
35149	Exenatide 10micrograms/0.04ml solution for injection 2.4ml pre-filled disposable devices	No
35150	Byetta 10micrograms/0.04ml solution for injection 2.4ml pre-filled disposable devices (AstraZeneca UK Ltd)	No
35251	Exenatide 5micrograms/0.02ml solution for injection 1.2ml pre-filled disposable devices	No
35462	Januvia 100mg tablets (Merck Sharp & Dohme Ltd)	No
35561	Prandin 2mg tablets (Novo Nordisk Ltd)	No
36774	Prandin 1mg tablets (Novo Nordisk Ltd)	No
36856	Gliclazide 80mg tablets (Sandoz Ltd)	No
36948	Prandin 0.5mg tablets (Novo Nordisk Ltd)	No
37617	Rosiglitazone 2mg tablet	Yes
37874	Vildagliptin 50mg / Metformin 850mg tablets	No
37875	Vildagliptin 50mg tablets	No
37902	Vildagliptin 50mg / Metformin 1g tablets	No
38355	Metformin 750mg modified-release tablets	No
38400	Glucophage SR 750mg tablets (Merck Serono Ltd)	No
38551	Eucreas 50mg/1000mg tablets (Novartis Pharmaceuticals UK Ltd)	No
39149	Galvus 50mg tablets (Novartis Pharmaceuticals UK Ltd)	No
39203	Eucreas 50mg/850mg tablets (Novartis Pharmaceuticals UK Ltd)	No
39560	Bolamyn SR 500mg tablets (Teva UK Ltd)	No
39598	Metformin 1g modified-release tablets	No
39729	Glucophage SR 1000mg tablets (Merck Serono Ltd)	No
39988	Metformin 500mg oral powder sachets sugar free	No
40007	Glucophage 1000mg oral powder sachets (Merck Serono Ltd)	No
40110	Glucophage 500mg oral powder sachets (Merck Serono Ltd)	No
40233	Metformin 1g oral powder sachets sugar free	No
40365	Glimepiride 1mg tablets (Actavis UK Ltd)	No
40425	Nazdol MR 30mg tablets (Teva UK Ltd)	No
40642	Victoza 6mg/ml solution for injection 3ml pre-filled pen (Novo Nordisk Ltd)	No
40693	Liraglutide 6mg/ml solution for injection 3ml pre-filled disposable devices	No
41204	Saxagliptin 5mg tablets	No
41431	Onglyza 5mg tablets (AstraZeneca UK Ltd)	No
41558	Glibenclamide 5mg tablets (Teva UK Ltd)	No
41559	Glibenclamide 5mg tablets (A A H Pharmaceuticals Ltd)	No
41593	Glibenclamide 2.5mg tablets (Teva UK Ltd)	No
42161	Orabet 500mg Tablet (Sandoz Ltd)	No
42790	Gliclazide 80mg Tablet (Merck Generics (UK) Ltd)	No
43065	Gliclazide 40mg tablets	No

For peer review only

43270	Metformin 500mg/5ml oral solution sugar free (Rosemont Pharmaceuticals Ltd)	No
43465	Zicron 40mg tablets (Bristol Laboratories Ltd)	No
43619	Metformin 1g / Sitagliptin 50mg tablets	No
43684	Janumet 50mg/1000mg tablets (Merck Sharp & Dohme Ltd)	No
44250	Metformin 500mg/5ml Oral solution (Hillcross Pharmaceuticals Ltd)	No
44304	Glyconon 500mg Tablet (DDSA Pharmaceuticals Ltd)	No
44473	Edicil MR 30mg tablets (Teva UK Ltd)	No
44738	Niddaryl 1mg tablets (Dee Pharmaceuticals Ltd)	No
45215	Gliclazide 80mg Tablet (Neo Laboratories Ltd)	No
45581	Metabet SR 500mg tablets (Morningside Healthcare Ltd)	No
45775	Saxagliptin 2.5mg tablets	No
45821	Onglyza 2.5mg tablets (AstraZeneca UK Ltd)	No
45831	Dacadis MR 30mg tablets (Mylan Ltd)	No
46458	Exenatide 2mg powder and solvent for prolonged-release suspension for injection vials	No
46469	Bydureon 2mg powder and solvent for prolonged-release suspension for injection vials (AstraZeneca UK Ltd)	No
46665	Linagliptin 5mg tablets	No
46716	Trajenta 5mg tablets (Boehringer Ingelheim Ltd)	No
46927	Tolbutamide 500mg tablets (Teva UK Ltd)	No
46989	Metabet SR 1000mg tablets (Morningside Healthcare Ltd)	No
47074	Gliclazide 80mg/5ml oral suspension	No
47894	Nazdol MR 30mg tablets (Consilient Health Ltd)	No
47939	Glucient SR 500mg tablets (Consilient Health Ltd)	No
48056	Gliclazide 80mg tablets (Sovereign Medical Ltd)	No
48139	Pioglitazone 30mg tablets (A A H Pharmaceuticals Ltd)	Yes
48149	Metformin 500mg tablets (Almus Pharmaceuticals Ltd)	No
48401	Sitagliptin 50mg tablets	No
48533	Sitagliptin 25mg tablets	No
49502	Glucophage SR 500mg tablets (Mawdsley-Brooks & Company Ltd)	No
49738	Metformin 1g modified-release tablets (A A H Pharmaceuticals Ltd)	No
50087	Januvia 50mg tablets (Merck Sharp & Dohme Ltd)	No
50124	Januvia 25mg tablets (Merck Sharp & Dohme Ltd)	No
50570	Glucophage SR 500mg tablets (Lexon (UK) Ltd)	No
50821	Metformin 850mg tablets (Pfizer Ltd)	No
50970	Metformin 500mg tablets (Bristol Laboratories Ltd)	No
51080	Metabet SR 1000mg tablets (Actavis UK Ltd)	No
51135	Metformin 500mg modified-release tablets (A A H Pharmaceuticals Ltd)	No
51527	Metformin 500mg tablets (Boston Healthcare Ltd)	No
51955	Gliclazide 80mg tablets (Accord Healthcare Ltd)	No
52203	Enyglid 0.5mg tablets (Consilient Health Ltd)	No
52221	Diagemet XL 500mg tablets (Genus Pharmaceuticals Ltd)	No
52442	Metformin 500mg tablets (Pfizer Ltd)	No
52634	Glucophage SR 500mg tablets (DE Pharmaceuticals)	No
53288	Gliclazide 30mg modified-release tablets (A A H Pharmaceuticals Ltd)	No
53478	Metformin 500mg modified-release tablets (Kent Pharmaceuticals Ltd)	No
53774	Metabet SR 500mg tablets (Actavis UK Ltd)	No
53867	Metformin 500mg tablets (Zentiva)	No
54150	Jentadueto 2.5mg/850mg tablets (Boehringer Ingelheim Ltd)	No
54182	Dapagliflozin 10mg tablets	No

For peer review only

54203	Forxiga 10mg tablets (AstraZeneca UK Ltd)	No
54265	Dapagliflozin 5mg tablets	No
54442	Metformin (roi) 1000mg Tablet	No
54480	Forxiga 5mg tablets (AstraZeneca UK Ltd)	No
54764	Gliclazide 80mg tablets (Arrow Generics Ltd)	No
54891	Saxagliptin 2.5mg / Metformin 1g tablets	No
54898	Metformin 850mg tablets (Almus Pharmaceuticals Ltd)	No
55270	Duformin 500mg Tablet (Dumex Ltd)	No
55711	Metformin 500mg tablets (Alliance Healthcare (Distribution) Ltd)	No
55739	Metformin 500mg tablets (Tillomed Laboratories Ltd)	No
55862	Gliclazide Oral solution	No
56008	Gliclazide 80mg tablets (Almus Pharmaceuticals Ltd)	No
56208	Pioglitazone 15mg tablets (A A H Pharmaceuticals Ltd)	Yes
56376	Rosiglitazone 4mg with glimepiride 4mg tablet	No
56437	Gliclazide 60mg modified-release tablets	No
56965	Komboglyze 2.5mg/1000mg tablets (AstraZeneca UK Ltd)	No
57147	Bolamyn SR 1000mg tablets (Teva UK Ltd)	No
57457	Metformin 500mg tablets (Aurobindo Pharma Ltd)	No
57601	Daonil 5mg tablets (Dowelhurst Ltd)	No
57659	Pioglitazone 30mg tablets (Actavis UK Ltd)	Yes
57830	Gliclazide 30mg modified-release tablets (Alliance Healthcare (Distribution) Ltd)	No
58051	Metformin 500mg/5ml oral solution	No
58607	Metformin 500mg/5ml oral solution sugar free (Zentiva)	No
59620	Glucophage SR 500mg tablets (Waymade Healthcare Plc)	No
59809	Alogliptin 6.25mg tablets	No
60012	Dapagliflozin 5mg / Metformin 1g tablets	No
60074	Metformin 1g modified-release tablets (Waymade Healthcare Plc)	No
60211	Canagliflozin 100mg tablets	No
60286	Metformin 500mg/5ml oral suspension	No
60430	Invokana 100mg tablets (Janssen-Cilag Ltd)	No
60495	Gliclazide 80mg tablets (Teva UK Ltd)	No
60643	Xigduo 5mg/1000mg tablets (AstraZeneca UK Ltd)	No
60968	Metformin 500mg modified-release tablets (Actavis UK Ltd)	No
61043	Sukkarto SR 1000mg tablets (Morningside Healthcare Ltd)	No
61311	Glimepiride 4mg tablets (Sigma Pharmaceuticals Plc)	No
61559	Sukkarto SR 500mg tablets (Morningside Healthcare Ltd)	No
61925	NovoNorm 500microgram tablets (Waymade Healthcare Plc)	No
61957	Gliclazide 40mg tablets (A A H Pharmaceuticals Ltd)	No
62014	Glimepiride 2mg tablets (Accord Healthcare Ltd)	No
62034	Laaglyda MR 60mg tablets (Consilient Health Ltd)	No
62144	Metformin 500mg modified-release tablets (DE Pharmaceuticals)	No
62265	Metformin 500mg modified-release tablets (Mawdsley-Brooks & Company Ltd)	No
62326	Vipidia 6.25mg tablets (Takeda UK Ltd)	No
62426	Pioglitazone 30mg tablets (Accord Healthcare Ltd)	Yes
62426	Pioglitazone 30mg tablets (Accord Healthcare Ltd)	No
62605	Metformin 850mg tablets (Kent Pharmaceuticals Ltd)	No
62661	Bydureon 2mg powder and solvent for prolonged-release suspension for injection pre-filled pen (AstraZeneca UK Ltd)	No
62824	Metformin 1g modified-release tablets (Actavis UK Ltd)	No
63045	Metformin 850mg tablets (Relonchem Ltd)	No

For peer review only

63046	Pioglitazone 45mg tablets (A A H Pharmaceuticals Ltd)	Yes	65563	Pioglitazone 15mg tablets (Alliance Healthcare (Distribution) Ltd)	Yes
63048	Gliclazide 80mg tablets (Alliance Healthcare (Distribution) Ltd)	No	65694	Metformin 500mg modified-release tablets (Waymade Healthcare Plc)	No
63107	Pioglitazone 45mg tablets (Waymade Healthcare Plc)	Yes	65923	Metformin 1g modified-release tablets (Mawdsley-Brooks & Company Ltd)	No
63131	Ziclaseg 30mg modified-release tablets (Lupin (Europe) Ltd)	No	66008	Synjardy 12.5mg/1000mg tablets (Boehringer Ingelheim Ltd)	No
63336	Trulicity 1.5mg/0.5ml solution for injection pre-filled pen (Eli Lilly and Company Ltd)	No	66136	Glucophage SR 1000mg tablets (Waymade Healthcare Plc)	No
63401	Trulicity 0.75mg/0.5ml solution for injection pre-filled pen (Eli Lilly and Company Ltd)	No	66399	Glimepiride 2mg tablets (A A H Pharmaceuticals Ltd)	No
63421	Pioglitazone 30mg tablets (Teva UK Ltd)	Yes	67056	Amaryl 1mg tablets (Lexon (UK) Ltd)	No
63516	Forxiga 10mg tablets (Waymade Healthcare Plc)	No	67781	Gliclazide 80mg tablets (Milpharm Ltd)	No
63785	Dulaglutide 0.75mg/0.5ml solution for injection pre-filled disposable devices	No	68203	Metformin 500mg modified-release tablets (Almus Pharmaceuticals Ltd)	No
63823	Dulaglutide 1.5mg/0.5ml solution for injection pre-filled disposable devices	No	68214	Metformin 500mg/5ml oral solution sugar free (A A H Pharmaceuticals Ltd)	No
64217	Jardiance 25mg tablets (Boehringer Ingelheim Ltd)	No	68289	Glimepiride 4mg tablets (Waymade Healthcare Plc)	No
64900	Glidipion 30mg tablets (Actavis UK Ltd)	Yes	68389	Metformin 500mg/5ml oral solution sugar free (Pinewood Healthcare)	No
64939	Glucient SR 1000mg tablets (Consilient Health Ltd)	No	68415	Gliclazide 30mg modified-release tablets (Phoenix Healthcare Distribution Ltd)	No
65083	Synjardy 5mg/1000mg tablets (Boehringer Ingelheim Ltd)	No	68636	Metformin 850mg/5ml oral solution sugar free	No
65344	Empagliflozin 5mg / Metformin 850mg tablets	No	68675	Glimepiride 4mg tablets (Somex Pharma)	No
65562	Pioglitazone 30mg tablets (Alliance Healthcare (Distribution) Ltd)	Yes	68819	Gliclazide 80mg tablets (Bristol Laboratories Ltd)	No

Supplemental table 3: Product codes for drugs used for diabetes and glitazone status.

For peer review only

Glitazones Case-Control				
	**N = 480**	**N = 1920**		
**Glitazone exposure status**				
**Per-year glitazone exposure**				
**Unexposed**	383 (79.8%)	1460 (76.0%)	Referent	-
**≤ 1 year**	41 (8.5%)	151 (7.9%)	1.11 (0.77, 1.61)	0.59
**1 < years ≤ 2**	23 (4.8%)	89 (4.6%)	1.06 (0.66, 1.71)	0.82
**2 < years ≤ 3**	29 (4.0%)	66 (3.4%)	1.13 (0.67, 1.92)	0.65
**3 < years ≤ 4**	8 (1.7%)	61 (3.2%)	0.52 (0.25, 1.10)	0.07
**4 < years ≤ 5**	2 (0.4%)	41 (2.1%)	0.18 (0.04, 0.77)	0.003
**5 < years ≤ 6**	2 (0.4%)	23 (1.2%)	0.30 (0.07, 1.30)	0.06
**> 6 years**	2 (0.4%)	29 (1.5%)	0.44 (0.10, 1.94)	0.23
**"First drug exposure" status**				
**Glitazone**	4 (0.8%)	13 (0.7%)	1.10 (0.35, 3.44)	0.87
**Other anti-type 2 diabetes drug**	476 (99.2%)	1907 (99.3%)	Referent	-

* Model adjusted for sex, age, IMD, retrospective prescription history (categorised yearly) and mean HbA1c levels. ** OR calculated for per year increase in variable.

Supplemental table 4: Case-control results for brain tumour cases with glitazone exposure in years duration groups and as first drug exposure.

For peer review only

Variable	Cases (%)	Controls (%)	Adjusted OR (95% CI) *	P-value
**Imputed HbA1c levels**				
	**N = 480**	**N = 1920**		
**Glitazone exposure status**				
**Exposed**	97 (20.2%)	460 (24.0%)	0.81 (0.66, 1.00)	0.11
**Unexposed**	383 (79.8%)	1460 (76.0%)	Referent	-
**Mean glitazone exposure duration (longest), days (SD)**	607 (583)	872 (760)	0.87 (0.82, 0.93) **	0.001
**Mean glitazone exposure duration (total), days (SD)**	604 (720)	1002 (823)	0.85 (0.79, 0.91) **	0.00005
**No HbA1c adjustment**				
	**N = 480**	**N = 1920**		
**Glitazone exposure status**				
**Exposed**	97 (20.2%)	460 (24.0%)	0.79 (0.62, 1.02)	0.07
**Unexposed**	383 (79.8%)	1460 (76.0%)	Referent	-
**Mean glitazone exposure duration (longest), days (SD)**	607 (583)	872 (760)	0.87 (0.80, 0.94)	0.0008
**Mean glitazone exposure duration (total), days (SD)**	604 (720)	1002 (823)	0.85 (0.78, 0.92)	0.00004

*** Adjusted for sex, age, IMD and retrospective prescription history. HbA1c adjustment differed according to the analysis.**

**** OR calculated for per year increase in variable.**

Supplemental table 5: Case-control results for brain tumour cases with glitazone exposure with HbA1C imputed or not adjusted in the multivariable models.

For peer review only

Glitazones - 6 months latency period	N = 480 Cases (%)	N = 1920 Controls (%)	Adjusted OR (95% CI) *	P-value
**Glitazone exposure status**				
**Exposed**	95 (19.8%)	460 (24.0%)	0.82 (0.64, 1.05)	0.12
**Unexposed**	385 (80.2%)	1460 (76.0%)	Referent	-
**Mean glitazone exposure duration (longest), days (SD)**	574 (581)	770 (651)	0.88 (0.81, 0.96) **	0.002
**Mean glitazone exposure duration (total), days (SD)**	771 (693)	988 (796)	0.89 (0.83, 0.96) **	0.0015
**Glitazones - 12 months latency period**	**N = 480**	**N = 1920**		
**Glitazone exposure status**				
**Exposed**	92 (19.2%)	457 (23.8%)	0.79 (0.61, 1.02)	0.07
**Unexposed**	388 (80.8%)	1463 (76.2%)	Referent	-
**Mean glitazone exposure duration (longest), days (SD)**	540 (572)	753 (642)	0.87 (0.79, 0.95) **	0.002
**Mean glitazone exposure duration (total), days (SD)**	670 (690)	958 (774)	0.87 (0.80, 0.94) **	0.0003
**Glitazones - 24 months latency period**	**N = 480**	**N = 1920**		
**Glitazone exposure status**				
**Exposed**	86 (17.9%)	449 (23.4%)	0.74 (0.57, 0.96)	0.02
**Unexposed**	394 (82.1%)	1471 (76.6%)	Referent	-
**Mean glitazone exposure duration (longest), days (SD)**	478 (557)	720 (625)	0.83 (0.75, 0.92) **	0.0004
**Mean glitazone exposure duration (total), days (SD)**	593 (672)	901 (730)	0.84 (0.76, 0.91) **	0.000007

Supplemental table 6: Case-control results for brain tumour cases with glitazone exposure for latency periods of 6, 12, and 24 months.

For peer review only

STROBE 2007 (v4) checklist of items to be included in reports of observational studies in epidemiology*

Checklist for cohort, case-control, and cross-sectional studies (combined)

Section/Topic	Item #	Recommendation	Reported on page #
**Title and abstract**	1	(*a*) Indicate the study's design with a commonly used term in the title or the abstract	1
		(*b*) Provide in the abstract an informative and balanced summary of what was done and what was found	2
**Introduction**			
Background/rationale	2	Explain the scientific background and rationale for the investigation being reported	5
Objectives	3	State specific objectives, including any pre-specified hypotheses	5
**Methods**			
Study design	4	Present key elements of study design early in the paper	6
Setting	5	Describe the setting, locations, and relevant dates, including periods of recruitment, exposure, follow-up, and data collection	6
Participants	6	(*a*) *Cohort study*—Give the eligibility criteria, and the sources and methods of selection of participants. Describe methods of follow-up<br>*Case-control study*—Give the eligibility criteria, and the sources and methods of case ascertainment and control selection. Give the rationale for the choice of cases and controls<br>*Cross-sectional study*—Give the eligibility criteria, and the sources and methods of selection of participants	6-7
		(*b*) *Cohort study*—For matched studies, give matching criteria and number of exposed and unexposed<br>*Case-control study*—For matched studies, give matching criteria and the number of controls per case	6-7
Variables	7	Clearly define all outcomes, exposures, predictors, potential confounders, and effect modifiers. Give diagnostic criteria, if applicable	6-8
Data sources/ measurement	8*	For each variable of interest, give sources of data and details of methods of assessment (measurement). Describe comparability of assessment methods if there is more than one group	6-8
Bias	9	Describe any efforts to address potential sources of bias	6-7
Study size	10	Explain how the study size was arrived at	6
Quantitative variables	11	Explain how quantitative variables were handled in the analyses. If applicable, describe which groupings were chosen and why	8
Statistical methods	12	(*a*) Describe all statistical methods, including those used to control for confounding	9-10
		(*b*) Describe any methods used to examine subgroups and interactions	9-10
		(*c*) Explain how missing data were addressed	9-10
		(*d*) *Cohort study*—If applicable, explain how loss to follow-up was addressed<br>*Case-control study*—If applicable, explain how matching of cases and controls was addressed	

For peer review only

		Cross-sectional study—If applicable, describe analytical methods taking account of sampling strategy	
		(*e*) Describe any sensitivity analyses	9-10
**Results**			
Participants	13*	(a) Report numbers of individuals at each stage of study—eg numbers potentially eligible, examined for eligibility, confirmed eligible, included in the study, completing follow-up, and analysed	10
		(b) Give reasons for non-participation at each stage	N/A
		(c) Consider use of a flow diagram	N/A
Descriptive data	14*	(a) Give characteristics of study participants (eg demographic, clinical, social) and information on exposures and potential confounders	10 & 18
		(b) Indicate number of participants with missing data for each variable of interest	11
		(c) *Cohort study*—Summarise follow-up time (eg, average and total amount)	
Outcome data	15*	*Cohort study*—Report numbers of outcome events or summary measures over time	
		Case-control study—Report numbers in each exposure category, or summary measures of exposure	18
		Cross-sectional study—Report numbers of outcome events or summary measures	
Main results	16	(*a*) Give unadjusted estimates and, if applicable, confounder-adjusted estimates and their precision (eg, 95% confidence interval). Make clear which confounders were adjusted for and why they were included	19-20
		(*b*) Report category boundaries when continuous variables were categorized	18-20
		(*c*) If relevant, consider translating estimates of relative risk into absolute risk for a meaningful time period	
Other analyses	17	Report other analyses done—eg analyses of subgroups and interactions, and sensitivity analyses	11-12
**Discussion**			
Key results	18	Summarise key results with reference to study objectives	12
Limitations	19	Discuss limitations of the study, taking into account sources of potential bias or imprecision. Discuss both direction and magnitude of any potential bias	13
Interpretation	20	Give a cautious overall interpretation of results considering objectives, limitations, multiplicity of analyses, results from similar studies, and other relevant evidence	14
Generalisability	21	Discuss the generalisability (external validity) of the study results	14
**Other information**			
Funding	22	Give the source of funding and the role of the funders for the present study and, if applicable, for the original study on which the present article is based	3-4

*Give information separately for cases and controls in case-control studies and, if applicable, for exposed and unexposed groups in cohort and cross-sectional studies.

Note: An Explanation and Elaboration article discusses each checklist item and gives methodological background and published examples of transparent reporting. The STROBE checklist is best used in conjunction with this article (freely available on the Web sites of PLoS Medicine at http://www.plosmedicine.org/, Annals of Internal Medicine at http://www.annals.org/, and Epidemiology at http://www.epidem.com/). Information on the STROBE Initiative is available at www.strobe-statement.org.

For peer review only

BMJ Open

Use of drugs for hyperlipidaemia and diabetes and risk of primary and secondary brain tumours: nested case control studies using the UK Clinical Practice Research Datalink (CPRD).

Journal:	*BMJ Open*
Manuscript ID	bmjopen-2023-072026.R1
Article Type:	Original research
Date Submitted by the Author:	28-Nov-2023
Complete List of Authors:	Robinson, Jamie W; University of Bristol<br>Martin, Richard; University of Bristol, Social Medicine<br>Ozawa, Mio; University College London<br>Elwenspoek, Martha; NIHR CLAHRC West, CLAHRC West; University of Bristol Medical School, Populations Health Sciences<br>Redaniel, Maria Theresa; University of Bristol, NIHR CLAHRC West; University of Bristol, School of Social and Community Medicine<br>Kurian, Kathreena; University of Bristol; University of Bristol<br>Ben-Shlomo, Yoav; University of Bristol, Department of Population Health Sciences; University Hospitals Bristol NHS Foundation Trust
**Primary Subject Heading**:	Epidemiology
Secondary Subject Heading:	Oncology
Keywords:	Epidemiology < ONCOLOGY, Head & neck tumours < ONCOLOGY, CLINICAL PHARMACOLOGY, EPIDEMIOLOGIC STUDIES, Neurological oncology < ONCOLOGY

SCHOLARONE™
Manuscripts

BMJ

I, the Submitting Author has the right to grant and does grant on behalf of all authors of the Work (as defined in the below author licence), an exclusive licence and/or a non-exclusive licence for contributions from authors who are: i) UK Crown employees; ii) where BMJ has agreed a CC-BY licence shall apply, and/or iii) in accordance with the terms applicable for US Federal Government officers or employees acting as part of their official duties; on a worldwide, perpetual, irrevocable, royalty-free basis to BMJ Publishing Group Ltd ("BMJ") its licensees and where the relevant Journal is co-owned by BMJ to the co-owners of the Journal, to publish the Work in this journal and any other BMJ products and to exploit all rights, as set out in our licence.

The Submitting Author accepts and understands that any supply made under these terms is made by BMJ to the Submitting Author unless you are acting as an employee on behalf of your employer or a postgraduate student of an affiliated institution which is paying any applicable article publishing charge ("APC") for Open Access articles. Where the Submitting Author wishes to make the Work available on an Open Access basis (and intends to pay the relevant APC), the terms of reuse of such Open Access shall be governed by a Creative Commons licence – details of these licences and which Creative Commons licence will apply to this Work are set out in our licence referred to above.

Other than as permitted in any relevant BMJ Author's Self Archiving Policies, I confirm this Work has not been accepted for publication elsewhere, is not being considered for publication elsewhere and does not duplicate material already published. I confirm all authors consent to publication of this Work and authorise the granting of this licence.

For peer review only

Use of drugs for hyperlipidaemia and diabetes and risk of primary and secondary brain tumours: nested case control studies using the UK Clinical Practice Research Datalink (CPRD).

Jamie W Robinson[1], Richard M Martin[1,2,3], Mio Ozawa[4], Martha M C Elwenspoek[2,5], Maria Theresa Redaniel[2,5], Kathreena M Kurian[1,6]*, Yoav Ben-Shlomo[2,5]*

1. MRC Integrative Epidemiology Unit, University of Bristol, Bristol, BS8 2BN, UK (JWR, RMM, KMK)
2. Department of Population Health Sciences, University of Bristol, Bristol, BS8 1UD, UK (RMM, MMCE, MTR, YBS)
3. National Institute for Health Research (NIHR) Bristol Biomedical Research Centre, University Hospitals Bristol and Weston NHS Foundation Trust and University of Bristol, Bristol, UK (RMM)
4. Population, Policy and Practice Research and Teaching Department, UCL Great Ormond Street Institute of Child Health, 30 Guilford Street, London, WC1N 1EH (MC)
5. The National Institute for Health Research Applied Research Collaboration West (NIHR ARC West), University Hospitals Bristol NHS Foundation Trust, BS1 2NT Bristol, UK (MMCE, MTR, YBS)
6. Brain Tumour Research Centre, University of Bristol, Bristol, BS10 5NB, UK (KMK)

* Joint senior authors.

Corresponding author: Yoav Ben-Shlomo (y.ben-shlomo@bristol.ac.uk) and Kathreena M Kurian (kathreena.kurian@bristol.ac.uk)

Short title: Drugs for hyperlipidaemia and diabetes and brain tumours

Word Count: Abstract: 255, Text: 3,860

Tables: 3

Supplemental tables: 6

For peer review only - http://bmjopen.bmj.com/site/about/guidelines.xhtml

Abstract

Objectives: Previous studies have suggested that fibrates and glitazones may have a role in brain tumour prevention. We examined if there is support for these observations using primary care records from the UK Clinical Practice Research Datalink (CPRD).

Design: We conducted two nested case-control studies using primary and secondary brain tumours identified within CPRD between 2000 to 2016. We selected cases and controls amongst the population of individuals who had been treated with any anti-diabetic or anti-hyperlipidaemic medication to reduce confounding by indication.

Setting: Adults older than 18 years registered with a General Practitioner in the UK contributing data to CPRD.

Results: We identified 7,496 people with any brain tumour (4,471 primary; 3,025 secondary) in total. After restricting cases and controls to those prescribed any anti-diabetic or anti-hyperlipidaemic medication, there were 1,950 cases and 7,791 controls in the fibrate and 480 cases with 1,920 controls in the glitazone analyses. Longer use of glitazones compared to all other anti-diabetic medications was associated with a reduced risk of primary (adjusted odds ratio [aOR] = 0.89 per year, 95% CI: 0.80, 0.98), secondary (aOR = 0.87 per year, 95% CI: 0.77, 0.99) or combined brain tumours (aOR = 0.88 per year, 95% CI: 0.81, 0.95). There was little evidence that fibrate exposure was associated with risk of either primary or secondary brain tumours.

Conclusions: Longer exposure to glitazones was associated with reduced primary and secondary brain tumour risk. Further basic science and population-based research should explore this finding in greater detail, in terms of replication and mechanistic studies.

Key Words: Brain cancer, glioma, secondary brain tumours, metastasis, fibrates, glitazones, observational epidemiology.

For peer review only - http://bmjopen.bmj.com/site/about/guidelines.xhtml

Strengths and limitations of this study

- Large case control studies using database free of selection bias and generalizable to other high income settings.
- Exposure well measured from medical systems avoiding recall bias
- Design accounted for confounding by indication and time-window bias
- Adjustment for individual socioeconomic status used an ecological proxy and residual confounding possible.
- All secondary brain cancers were combined to maintain statistical power

For peer review only

For peer review only - http://bmjopen.bmj.com/site/about/guidelines.xhtml

Introduction

People diagnosed with primary or secondary (breast, lung, skin melanoma, colorectal and kidney) brain tumours typically have less than a 20% five-year survival rate and brain tumours are responsible for the most years of life lost of any cancer [1]. The development of novel cancer drugs is beset by substantial costs and high failure rates. Re-purposing existing approved drugs for different diseases than originally designed potentially saves the considerable time and costs involved in conducting new clinical trials.

Nuclear hormone receptors (NHR) have crucial roles in cellular homeostasis and have been implicated in the development of cancer. Peroxisome proliferator-activated receptors (PPARs) are a family of ligand-activated transcription factors that regulate the expression of genes and, as a subtype of NHRs, are involved in the control of proliferation and differentiation of cells[2-4]. There are three isoforms of PPARs which are designated as -α, -β/δ and -γ. The genes regulated by these transcription factors are also involved in the transport, metabolism and storage of fatty acids, in inflammatory processes and in the onset of type 2 diabetes mellitus[3, 9, 10]. PPARs have been identified as potential candidates in cancer treatment, with some animal models and early-stage human trials showing beneficial outcomes, including arresting development of precursor cancer cells, decreased serum cancer antigens and tolerance by patients[5-8].

PPAR-α and -γ are clinically significant due to the use of agonistic compounds such as fibrates in hyperlipidaemia and thiazolidinediones (glitazones) in diabetes respectively. Both glitazones [11] and fibrates such as fenofibrate cross the blood-brain barrier, albeit at a slower rate[12]. PPARs are expressed in astrocytes, oligodendrocytes and microglia, and may play a role in growth and differentiation of those cells, including brain tumour stem cells[13]. These drugs have the potential for being repurposed for the prevention of brain cancers given their safety profile and cost.

We hypothesized that PPAR-α and -γ agonists (fibrates and glitazones), would be associated with a reduced risk of developing brain tumours compared to other treatments for hyperlipidaemia and diabetes using the UK Clinical Practice Research Datalink (CPRD), a very large population-based primary care database.

Materials and methods

We used the CPRD database which has data from a network of over 2,000 general practitioners (GPs) from more than 670 practices across the UK. Validated clinical

For peer review only

For peer review only - http://bmjopen.bmj.com/site/about/guidelines.xhtml

information is held on over 16 million registered patients, amounting to circa 7% of the UK population [14] and broadly representative of that population [15, 16]. Types of data held within the CPRD include, but are not limited to, patient demographics, referrals, hospital admissions, clinical diagnoses and drug prescriptions. Data from secondary care or hospital visits are routinely fed back into the electronic records' system via staff at GP practices. CPRD is jointly sponsored by the Medicines and Healthcare products Regulatory Agency (MHRA) and the National Institute of Health Research (NIHR). Data were extracted from CPRD GOLD and linked to ONS death registration data and census data on area deprivation (see below).

Study design and eligible Participants

We undertook two nested case control studies from within the CPRD database as the eligibility criteria (see below) for the fibrate and glitazone analyses were different. All participants in the study had to be 18 years or older and registered within the CPRD between 1st January 2000 to 31st December 2016, the former being the first year that glitazones were licensed within the UK. We specified that eligible participants had to be treated with either (i) an anti-hyperlipidaemic or (ii) anti-diabetic medication to reduce the likelihood of "confounding by indication", a type of confounding which can distort the association between drug exposure and outcome when an indication to prescribe a drug (e.g. diabetes) is also associated with the outcome. All brain tumour cases and controls that were not treated with either these classes of drugs were therefore not included in the sample selection and analyses. The sample size was pragmatic based on all available cases from the dataset rather than based an "a priori" sample size calculation to maximise power.

Cases

Incident brain tumour (primary or secondary) cases were identified using a large list of Read codes (**Supplemental table 1**) and were classified as either of primary or secondary cancers using histopathology [17]. For secondary tumours, we did not have information on the site of primary tumour, but we retrospectively searched for another prior cancer diagnosis to estimate the site of the primary cancer. However, if this had been recorded prior to 1st Jan 2000, or recorded at a non-CPRD participating GP, it may have been missing from our file and thus not captured by our retrospective search.

For peer review only - http://bmjopen.bmj.com/site/about/guidelines.xhtml

Controls

We sampled controls using "incidence density sampling", whereby on the date each case was diagnosed we identified the potential pool of controls who did not have a prior diagnosis of a brain tumour. Up to four controls were randomly selected per case (to maximise power) and were age-frequency matched to cases by 10-year age groups (<20, 20-29, 30-39, 40-49, 50-59, 60-69, 70-79 and ≥80 years) and male/female sex to ensure reasonable balance of these variables across case-control strata.

Both "immortal time" and "time-window" biases are potential hazards in pharmaco-epidemiology studies leading to misleading results [18], though the former is not relevant to our case-control study design [19]. Time-window bias occurs in case-control studies when the method of selecting controls results in an artefactually longer time window for potential exposure to be identified than their matched cases resulting in a spurious inverse (protective) association between the exposure and outcome [18]. To prevent time-window bias, we specified that controls must have the same retrospective duration of potential exposure (any drug treatment for either hyperlipidaemia or diabetes) within CPRD as the matched case, based on the case index date plus or minus six months. For example, if a case had 6.3 years of any drug medication exposure prior to their diagnosis date, then only controls who also had between 5.8 and 6.8 years of any drug exposure over the same secular time period were sampled and hence had the same potential for exposure to either a fibrate or glitazone as cases.

Exposures

All participants were treated with some medication for either type 2 diabetes or hyperlipidaemia. If they received a fibrate or glitazone they were classified as "exposed" whilst all the other drugs used for the management of these diseases were classified as the unexposed comparator. Product codes used to determine exposure status are given in **Supplemental table 2 and Supplemental table 3**. We excluded participants with type 1 diabetes, defined as those participants treated solely with insulin therapy, as these patients are very different to those with the more common type 2 diabetes in terms of causes and duration of dysregulated glycaemic control. Participants who started on oral anti-diabetic medication, but at some point in time also received insulin therapy, were eligible for inclusion. Participants on combination therapy were not excluded (for example, both glitazone plus another anti-diabetic medication); these participants were considered as exposed, if one of the therapies was the drug of interest. Similarly, some participants were exposed to both fibrates

For peer review only

For peer review only - http://bmjopen.bmj.com/site/about/guidelines.xhtml

and glitazones and were considered exposed for both studies (however we tested both these assumption in sensitivity analyses; see below).

Exposure was classified in three ways: (i) "Exposed binary" (Yes/No) indicating that a subject has been ever exposed to a prescription for fibrates or glitazones; (ii) "Longest exposure duration" which was the total uninterrupted time (no breaks of 90 days or more between prescriptions) each participant had been prescribed either fibrates or glitazones. If there was more than one such period, then we took the longest duration; (iii) "Total exposure duration", which was the sum of the total prescription duration (with or without interruptions). Exposure duration for both the longest and total exposure duration were categorised as follows: unexposed, ≤ 1 year, >1 and ≤ 2 years, >2 and ≤ 3 years, >3 and ≤ 4 years , >4 and ≤ 5 years , >5 and ≤ 6 years , >6 and ≤ 7 years, >7 years. Due to small numbers of participants reaching longer exposure durations, any duration exposure periods over 6 years were considered in the longest duration category in the glitazone analysis, and over 7 years as the longest duration category in the fibrate analysis.

Potential Confounders

The following variables were considered as potential confounders that might influence risk of developing a brain tumour and the choice of drug that a doctor might prescribe: age group, sex and socioeconomic status (SES). We used the Index of Multiple Deprivation 2015 (IMD) [20], as an ecological proxy for individual SES. This is a commonly used measure in the UK that uses census data on a wide variety of economic and health factors to derive a postcode-based deprivation ranked score so that that a higher score indicates less deprivation. IMD scores were grouped into five equal sized groups from most (quintile 1) to least deprived areas (quintile 5), so we could adjust for any non-linear associations.

In the glitazone analyses, we also tried to adjust for the severity of diabetes as a confounder, as severity may determine choice of anti-diabetic medications and possible cancer risk. We used glycosylated haemoglobin (HbA1c) levels as a measure of glycaemic control and hence severity of diabetes, where available. We converted and standardised the measures to the International Federation of Clinical Chemistry (IFCC) units (mmols/mol). The mean value was calculated using all available HbA1c readings for a subject. A three-level ordinal variable was created: 1, well controlled diabetes (≤ 58 mmols/mol); 2, sub-optimally controlled diabetes (> 58 mmols/mol and ≤ 75 mmols/mol); 3, poorly controlled diabetes (> 75 mmols/mol). If there were no measures of the participant's HbA1c levels, we assigned them to the well-controlled group based on the assumption that the clinician did not feel it

For peer review only - http://bmjopen.bmj.com/site/about/guidelines.xhtml

necessary to monitor the patient's glycaemic control (however, see below for sensitivity analyses that examine this assumption). We attempted to restrict HbA1c readings to only pre-baseline measures; however due to missing data, we had to occasionally include post-baseline HbA1c readings as a proxy measure.

Statistical Methods

Both case-control analyses were conducted using logistic regression to compute odds ratios (OR) and 95% confidence intervals (CI) of exposure to fibrate and glitazone drugs versus other drugs used for the same disease. Unadjusted and multivariable models were run, adjusting for the previously mentioned potential confounders with cases stratified by primary and secondary brain tumours and then combined as all brain tumours if there was no evidence of heterogeneity of effect to enhance statistical power. In addition, we adjusted for total potential exposure duration, defined as the time from either diagnosis or control index date and start of any medication first prescribed for either hyperlipidaemia or diabetes. This was one of the matching variables used to avoid time window bias. Exposure duration was analysed both as a continuous ordinal variable and also as a "dummy" variable to check for any evidence of non-linearity in the pattern of the odds ratios. We used logistic regression rather than conditional logistic models, as the latter may introduce collider bias and instead we used the matching variables as covariates in the unmatched logistic model as the recommended procedure (see reference [21] for detailed justification).

Sensitivity Analyses

The following sensitivity analyses were conducted to check the robustness of our main analyses: (i) Potential selection bias: we reclassified exposure only if the participant's first medication was a fibrate or glitazone. Someone whose first treatment was another drug but was later switched to a glitazone or fibrate in this analysis was classified as unexposed; (ii) Dual exposure: to evaluate if one drug had an effect on risk while the other did not, participants were dropped from the analysis if they were exposed to both a fibrate and a glitazone at the same time; (iii) Latency period: analyses were repeated excluding any exposure for short latencies defined as six, 12 and 24 months prior to the index date; and (iv) Missing HbA1c levels: we used multiple imputation (55 datasets generated, combined using Rubin's rules) to predict missing HbA1c levels based on case control status, age, IMD, sex, retrospective prescription history, ever exposure to a glitazone, total glitazone exposure and number of consultations, defined as each day the patient had at least one in-person consultation. The MICE algorithm in R [22] was used to impute missing HbA1c levels. We

For peer review only - http://bmjopen.bmj.com/site/about/guidelines.xhtml

also examined whether HbA1c predicted case control status both in the complete case as well as the imputation dataset. This is because if there was no evidence of an association with HbA1c then it cannot be a confounder and did not need to be included in the multivariable model, avoiding the issue of missing values. Sensitivity analyses were conducted using all brain tumour cases as there was limited statistical power in the separate primary and secondary cancer analyses.

Patient and Public Involvement

Patients or the public were not involved in the design, or conduct, or reporting, or dissemination plans of our research

Results

For the fibrate analyses, there were a total of 1,950 cases, of which 1,094 were primary and 856 were secondary brain tumours and 7,791 matched controls. For the glitazone analyses there were a total of 480 cases, of which 278 were primary and 202 were secondary brain tumours and 1,920 controls. There was little evidence of any important differences in age group, sex and IMD distribution between cases versus controls in both the fibrate and glitazone datasets (**Table 1**). Cases were more likely to have well-controlled HbA1c but this was because they were more likely to have missing HbA1c values which by default were classified as well-controlled diabetes.

For peer review only - http://bmjopen.bmj.com/site/about/guidelines.xhtml

Table 1: Distribution of possible confounders by case control status for both fibrate and glitazone datasets.

Fibrates Case-Control				
**Variable**	Cases (%)	Controls (%)	Crude OR (95% CI)	P
	**N = 1950**	**N = 7791**		
**Male**	1118 (57.3%)	4469 (57.4%)	Referent	-
**Female**	832 (42.7%)	3322 (42.6%)	1.00 (0.91, 1.11)	0.98
**Age, years**				
**< 20**	0 (0.0%)	0 (0.0%)	-	-
**20 – 29**	0 (0.0%)	0 (0.0%)	-	-
**30 – 39**	2 (0.1%)	5 (0.1%)	1.58 (0.31, 8.17)	0.58
**40 – 49**	6 (0.3%)	29 (0.4%)	0.82 (0.34, 1.98)	0.66
**50 – 59**	40 (2.1%)	193 (2.5%)	0.82 (0.58, 1.16)	0.26
**60 – 69**	220 (11.3%)	848 (10.9%)	1.03 (0.87, 1.21)	0.75
**70 – 79**	561 (28.8%)	2279 (29.3%)	0.97 (0.87, 1.09)	0.65
**≥ 80**	1121 (57.5%)	4437 (57.0%)	Referent	-
**IMD, quintile**				
**1, lower SES**	457 (23.4%)	1768 (22.7%)	1.05 (0.91, 1.22)	0.48
**2**	439 (22.5%)	1790 (23.0%)	Referent	-
**3**	430 (22.1%)	1594 (20.5%)	1.10 (0.95, 1.28)	0.21
**4**	331 (17.0%)	1466 (18.8%)	0.92 (0.79, 1.08)	0.31
**5, higher SES**	293 (15.0%)	1173 (15.1%)	1.02 (0.86, 1.20)	0.83
**Glitazones Case-Control**				
	**N = 480**	**N = 1920**		
**Male**	277 (57.7%)	1108 (57.7%)	Referent	-
**Female**	203 (42.3%)	812 (42.3%)	1.00 (0.82, 1.22)	1.00
**Age, years**				
**< 20**	1 (0.2%)	1 (0.1%)	3.93 (0.24, 63.00)	0.33
**20 – 29**	1 (0.2%)	7 (0.4%)	0.56 (0.07, 4.58)	0.59
**30 – 39**	3 (0.6%)	11 (0.6%)	1.07 (0.30, 3.87)	0.92
**40 – 49**	7 (1.5%)	28 (1.5%)	0.98 (0.42, 2.27)	0.97
**50 – 59**	15 (3.1%)	76 (4.0%)	0.78 (0.44, 1.37)	0.38
**60 – 69**	56 (11.7%)	233 (12.1%)	0.94 (0.68, 1.30)	0.73
**70 – 79**	135 (28.1%)	535 (27.9%)	0.99 (0.79, 1.25)	0.94
**≥ 80**	262 (54.6%)	1029 (52.6%)	Referent	-
**IMD, quintile**				
**1, lower SES**	90 (18.8%)	362 (18.9%)	1.33 (0.95, 1.86)	0.09
**2**	114 (23.8%)	376 (19.6%)	1.62 (1.18, 2.24)	0.003
**3**	109 (22.7%)	384 (20.0%)	1.52 (1.10, 2.10)	0.01
**4**	78 (16.3%)	418 (21.8%)	Referent	-
**5, higher SES**	89 (18.5%)	380 (19.8%)	1.26 (0.90, 1.75)	0.18
**Mean prescription history, years (SD)**	2.12 (2.41)	1.99 (2.27)	1.02 (0.98, 1.07) *	0.27
**HbA1c ***				
**1, well controlled**	418 (87.0%)	1490 (77.6%)	Referent	-
**2**	42 (9.0%)	305 (15.9%)	0.49 (0.35, 0.69)	0.001
**3, poorly controlled**	20 (4.0%)	125 (6.5%)	0.57 (0.35, 0.93)	0.02
**Missing (coded as 1)**	338 (70.4%)	979 (51.0%)	-	-

* HbA1C = glycosylated haemoglobin a measure of glycaemic control used to monitor patients with diabetes.

For peer review only - http://bmjopen.bmj.com/site/about/guidelines.xhtml

There was little evidence that any exposure to fibrates was associated with brain tumour risk for either primary (adjusted OR [aOR] 0.99, 95% CI: 0.75, 1.30; P = 0.94), secondary (aOR 0.91, 95% CI: 0.67, 1.23; P = 0.55) or all tumours (aOR 0.98, 95% CI: 0.78, 1.24; P = 0.88) (**Table 2**). We found little evidence for a dose-response relationship of fibrate exposure, either as longest or total duration, with brain tumour risk for primary, secondary or all tumours (aORs varied from 0.95 to 1.01 and all consistent with chance). The sensitivity analysis, which only classified exposed if this was the first prescribed anti-hyperlipidaemia medication, found similar results but with less precision (aOR = 0.92, 95% CI: 0.69, 1.22; P = 0.54)

There was very weak evidence of an inverse association between ever exposure to glitazones (aOR = 0.80, 95% CI: 0.65, 1.07; P = 0.14) and all tumours, which was stronger with secondary tumours (aOR = 0.68, 95% CI: 0.46, 1.02; P = 0.06) rather than primary tumours (aOR = 0.96, 95% CI 0.69, 1.33; P = 0.81) (**Table 3**), though this could have been due to chance. The inverse association was stronger and consistent across both primary and secondary tumours when the exposure was classified as longest duration; primary (aOR per year 0.89, 95% CI: 0.80, 0.98; P = 0.02), secondary (aOR per year 0.87, 95% CI: 0.77, 0.99; P = 0.02) and all brain tumours (aOR per year 0.88, 95% CI: 0.81, 0.95; P = 0.002). Very similar results were seen with total duration of exposure (e.g. aOR per year for all tumours 0.85, 95% CI: 0.79, 0.92; P = 8.00×10^{-5}).

For peer review only

For peer review only - http://bmjopen.bmj.com/site/about/guidelines.xhtml

Table 2: Case-control results for primary, secondary and all brain tumour cases with fibrate exposure.

Fibrates												
	Primary brain tumours				Secondary brain tumours				All brain tumours			
**Variable**	**Cases (%)**	**Controls (%)**	**Adjusted OR (95% CI) ***	**P**	**Cases (%)**	**Controls (%)**	**Adjusted OR (95% CI) ***	**P**	**Cases (%)**	**Controls (%)**	**Adjusted OR (95% CI) ***	**P**
	N = 1094	N = 4375			N = 856	N = 3416			N = 1950	N = 7791		
**Fibrate exposure status**												
**Exposed**	68 (6.2%)	278 (6.4%)	0.99 (0.75, 1.30)	0.94	56 (6.5%)	245 (7.2%)	0.91 (0.67, 1.23)	0.55	124 (6.4%)	523 (6.7%)	0.98 (0.78, 1.24)	0.88
**Unexposed**	1026 (93.8%)	4097 (93.7%)	Referent	-	800 (93.5%)	3171 (92.8%)	Referent	-	1826 (93.6%)	7268 (93.3%)	Referent	-
**Fibrate longest duration, days (SD) ****	799 (1050)	755 (913)	1.01 (0.94, 1.09)	0.76	676 (913)	878 (1123)	0.95 (0.87, 1.03)	0.16	743 (989)	813 (1018)	0.97 (0.91, 1.03)	0.35
**Fibrate total duration, days (SD) ****	969 (1183)	1073 (1233)	0.99 (0.93, 1.05)	0.64	871 (1232)	1217 (1408)	0.95 (0.88, 1.01)	0.08	925 (1202)	1141 (1319)	0.97 (0.92, 1.01)	0.11

* Model adjusted for sex, age, IMD, retrospective prescription history (categorised yearly). ** OR calculated for per year increase in variable.

For peer review only - http://bmjopen.bmj.com/site/about/guidelines.xhtml

Table 3: Case-control results for primary, secondary and all brain tumour cases with glitazone exposure.

	Primary brain tumours				Secondary brain tumours				All brain tumours			
**Variable**	**Cases (%)**	**Controls (%)**	**Adjusted OR (95% CI) ***	**P**	**Cases (%)**	**Controls (%)**	**Adjusted OR (95% CI) ***	**P**	**Cases (%)**	**Controls (%)**	**Adjusted OR (95% CI) ***	**P**
	N = 278	N = 1112			N = 202	N = 808			N = 480	N = 1920		
**Glitazone exposure status**												
**Exposed**	61 (21.9%)	261 (23.5%)	0.96 (0.69, 1.33)	0.81	36 (17.8%)	199 (24.6%)	0.68 (0.46, 1.02)	0.06	97 (20.2%)	460 (24.0%)	0.80 (0.65, 1.07)	0.14
**Unexposed**	217 (78.1%)	851 (76.5%)	Referent	-	166 (82.2%)	609 (75.4%)	Referent	-	383 (79.8%)	1460 (76.0%)	Referent	-
**Glitazone longest duration, days (SD) ****	551 (599)	851 (754)	0.89 (0.80, 0.98)	0.02	701 (550)	899 (767)	0.87 (0.77, 0.99)	0.02	607 (583)	872 (760)	0.88 (0.81, 0.95)	0.002
**Glitazone total duration, days (SD) ****	684 (695)	1000 (809)	0.90 (0.82, 0.99)	0.02	845 (681)	1073 (842)	0.88 (0.79, 0.98)	0.01	603 (720)	1002 (823)	0.86 (0.79, 0.92)	0.00008

* Model adjusted for sex, age, IMD, retrospective prescription history (categorised yearly) and mean HbA1c levels. ** OR calculated for per year increase in variable.

For peer review only - http://bmjopen.bmj.com/site/about/guidelines.xhtml

Sensitivity analyses

Results from the analysis of duration period as a categorical, instead of a continuous variable, found that the inverse association with glitazone and all tumours only emerged after 3 years of exposure; however, the confidence intervals for the shorter duration periods were sufficiently wide as to not exclude any dose-response effect across time (see **Supplemental table 4**). When we only classified exposed if this was the first prescribed anti-diabetes medication, we found little evidence of an association but with very wide confidence intervals due to fewer exposed cases and controls (aOR = 1.10, 95% CI: 0.35, 3.44; P = 0.87) (see **Supplemental table 4**). Effects were similar (longest duration aOR = 0.85, 95% CI 0.78, 0.93; P = 4.00x10^{-4}) when investigating the effects of monotherapy

Investigating Missing HbA1c levels

HbA1c results were missing for 54.8% of subjects in the glitazone analysis. We observed similar results for the association of both glitazone exposure status (aOR = 0.81, 95% CI: 0.66, 1.00; P = 0.11) and longest duration of glitazone exposure (aOR per year = 0.87, 95% CI: 0.82, 0.93; P = 0.001) with all brain tumour risk when multiple imputation was used for the missing HbA1c values (**Supplemental table 5**). There was little evidence of an association between poorly versus well-controlled HbA1c and case-control status in both the imputed (aOR = 0.95, 95% CI: 0.69, 1.32; P = 0.80) and complete case analysis (aOR = 1.02, 95% CI: 0.60, 1.73; P = 0.80), suggesting that HbA1C may not be a confounder as it did not predict case control status and therefore adjustment may not be necessary. This observation was supported by the aOR without adjustment for HbA1c which was almost identical to the imputed results so that the odds ratio per year of glitazaone exposure with longest duration was 0.87 (95% CI: 0.80, 0.94; P = 0.0008) (**Supplemental table 5**).

Latency Period

Similar associations of both fibrates and glitazones with all brain tumour risk were observed in the sensitivity analyses with different latency periods. In the glitazone analysis, the inverse association seen in the initial analyses for longest duration was very similar as the latency period increased (aOR after a 6-, 12- and 24-month latency were 0.88, 0.87, 0.83 respectively) and unlikely to be due to chance (**Supplemental table 6**).

Discussion

To the best of our knowledge, this is the first pharmacoepidemiological study of the effects of fibrate and glitazone prescription on risk of brain tumours compared to other treatments for

For peer review only

For peer review only - http://bmjopen.bmj.com/site/about/guidelines.xhtml

hyperlipidaemia and type 2 diabetes. There have been various *in vivo* studies that have suggested fibrate exposure may be protective for brain tumours, specifically gliomas, by modulating PPAR-α inhibition [23-26]. There is similar evidence available for the consideration of glitazones as a treatment option from *in vivo* studies [27-30]. However, prior to this study, there have been no other pharmacoepidemiological studies that examine whether fibrates or glitazones affect brain tumour risk.

In the glitazone case-control analysis, there was a modest inverse effect observed for ever exposure to glitazones, which was seen more strongly in the dose-response analyses, especially after exposure durations of 4 or more years, in both univariable and multivariable models. Repeating our analyses and accounting for various latency periods did not alter our conclusions. Similar inverse associations were seen for both primary and secondary brain tumours, but limited data meant analyses could not test whether there were differences by type of primary cancer for those subjects with secondary brain tumours.

We found little evidence that exposure to fibrates was associated with all, primary or secondary brain tumour risk, in contrast to some previous literature[31]. Sensitivity analyses, investigating a potential dose-response effect, or exposure status based on whether fibrates were the first prescribed drug for treatment, also showed little evidence of an association between fibrate exposure and all brain tumour risk.

The assumption that missing HbA1c levels should be assumed to reflect good glycaemic control was tested using follow-up sensitivity analyses to ensure this did not bias the potential negative association between glitazone exposure and brain tumour risk. However, in both the multiple imputation and complete case analysis we found no association between HbA1c and case control status. Furthermore, the results using both multiple imputation or no adjustment were consistent with the primary analyses.

This study has several strengths and limitations. We used a nested case control design within CPRD, a large and well-established database which has been validated by numerous sources [15, 16, 32]. It should be free of selection bias as almost all UK residents are registered with a GP and the population captured by CPRD-registered practices is representative of the general population [14]. Exposure was well measured as it was recorded from medical systems that used the record to print out prescribed drugs for redemption at a pharmacist. The exposure data were collected prospectively, prior to diagnosis, which will avoid recall bias. However, we have only measured receipt of a prescription and have no data on actual drug intake or

For peer review only - http://bmjopen.bmj.com/site/about/guidelines.xhtml

therapeutic levels. Despite this, as both the response to therapy are monitored the dosage is likely to be adjusted in response to good or bad control of the subjects' lipids and blood sugar levels. Exposure was classified from first prescription of any drug for lipids or diabetes and controls were sampled to have similar total prescription duration to avoid time-window bias, which was also adjusted in the multivariable models. The risk of confounding by indication was minimized by only sampling cases and controls who could have potentially been exposed to fibrates or glitazones because of a clinical indication. However, the consequence of this sampling strategy is that the number of available cases was markedly reduced with reduced statistical power to detect modest effects. Survivor bias is unlikely as all cases were included from diagnosis regardless of subsequent life expectancy and there is no evidence that the exposures themselves effect mortality risk. Not all potential risk factors for brain tumours were captured by CPRD (e.g. previous irradiation) but if these are not associated with exposure this will not cofound the results. Adjustment for SES was at an ecological rather than individual level but if anything is likely to bias the associations to the null due to non-differential measurement error. We had to combine all secondary brain cancers regardless of the primary cancer. Many of the secondary brain tumour cases had a previous diagnosis of common cancers which metastasise to brain, in particular breast, lung and skin cancers.

Overall longer glitazone exposure was associated with a reduced risk of brain metastasises from these primary sites but larger studies should be conducted to determine whether the association of glitazone exposure and secondary brain tumours is similar or not across these different primary sites. Risk of brain tumours may be greater in populations with either type 2 diabetes or hyperinsulinaemia [33, 34] though further studies are required to confirm these associations. We cannot say if we would have observed the same associations if glitazones were prescribed as a preventative therapy in a high-risk non-diabetic population as the generalizability of our results are limited to those brain tumour cases with type 2 diabetes.

Conclusions

Longer exposure to glitazones was inversely associated with diagnosis of primary and secondary brain tumours, but we found little evidence of an association between fibrates and any type of brain tumour. Our results should be generalizable to other high income settings but may not be generalizable to low middle income populations. Further research is required to investigate whether these findings are replicated using independent datasets, preferably larger in size and/or with better data on glycaemic control and other potential confounders. If the glitazone association is biologically causal, this may lead to a better understanding of

For peer review only - http://bmjopen.bmj.com/site/about/guidelines.xhtml

pathophysiological mechanisms and potential therapies for the prevention of brain cancers. For example, patients with specific aggressive cancers that have a high probability of brain metastases, could be treated with glitazones as a tertiary prevention strategy to prevent or delay secondary spread to the brain. Such a hypothesis could be tested in a future double blind clinical trial if stronger evidence emerges from other studies, given the safety and current use of glitazones for the management of diabetes.

Ethics Approval

The study was approved by the Independent Scientific Advisory Committee (ISAC) for the UK Medicines and Healthcare Products Regulatory Agency (ISAC protocol number: 18_149R). Participant consent is not required for ISAC approved CPRD studies using purely observational data. We assert that all procedures contributing to this work comply with the ethical standards of the relevant national and institutional committees on human experimentation and with the Helsinki Declaration of 1975, as revised in 2008.

Availability of Data and Materials

The Clinical Practice Research Datalink (CPRD) is an electronic healthcare record database open to all researchers. Researchers can apply to access CPRD data, and, if successful, can access the data of their choosing. The CPRD charges researchers and other organisations to access this data. The data that support the findings of this study are available from the Clinical Practice Research Datalink (CPRD) but restrictions apply to the availability of these data, which were used under license for the current study, and so are not publicly available.

Competing Interests

JWR receives funding from Biogen for unrelated research. All other authors declare no conflicts of interest.

Funding

The Medical Research Council (MRC)/University of Bristol Integrative Epidemiology Unit (IEU) is supported by the MRC and the University of Bristol (MC_UU_12013/1, MC_UU_12013/2). JWR is supported by a joint studentship from NHS Southmead Hospital Charity (SOCS/SJ1447). RMM is supported by a Cancer Research UK Programme Grant, the Integrative Cancer Epidemiology Programme (C18281/A29019). JWR and RMM are members of the MRC IEU which is supported by the Medical Research Council and the

For peer review only

University of Bristol (MC_UU_12013/1-9). RMM is supported by the National Institute for Health Research (NIHR) Bristol Biomedical Research Centre which is funded by the National Institute for Health Research and is a partnership between University Hospitals Bristol NHS Trust and Weston NHS Foundation Trust and the University of Bristol. RMM is a National Institute for Health Research Senior Investigator (NIHR202411). The views expressed in this publication are those of the author(s) and not necessarily those of the NIHR or the UK Department of Health and Social Care. This work was supported by funding from the Brain Tumour Bank and Research Fund, Southmead Hospital Charity (Registered Charity Number: 1055900), and University of Bristol Campaigns and Alumni funding. This work was supported by CRUK [grant number C18281/A29019], as part of the Integrative Cancer Epidemiology Programme. MTR, MMCE, and YBS are supported by the NIHR Applied Research Collaboration West (NIHR ARC West). The views expressed in this article are those of the author(s) and not necessarily those of the NIHR or the Department of Health and Social Care.

Author's Contributions

Study hypothesis and design: KMK, RMM, YBS. Extraction of CPRD data: MMCE, MTR. Conceptualisation of analysis plan: RMM, YBS, KMK, MC, MTR Performed data management and statistical analyses: JWR, MC. Writing of the initial versions of the manuscript: JWR followed by YBS. All authors read, reviewed and provided feedback for final manuscript.

For peer review only

For peer review only - http://bmjopen.bmj.com/site/about/guidelines.xhtml

References

1. **Brain, other CNS and intracranial tumours statistics** [https://www.cancerresearchuk.org/health-professional/cancer-statistics/statistics-by-cancer-type/brain-other-cns-and-intracranial-tumours]
2. Kitamura S, Miyazaki Y, Shinomura Y, Kondo S, Kanayama S, Matsuzawa Y: **Peroxisome proliferator-activated receptor gamma induces growth arrest and differentiation markers of human colon cancer cells**. *Jpn J Cancer Res* 1999, **90**(1):75-80.
3. Poulsen L, Siersbaek M, Mandrup S: **PPARs: fatty acid sensors controlling metabolism**. *Semin Cell Dev Biol* 2012, **23**(6):631-639.
4. Rosen ED, Spiegelman BM: **PPARgamma : a nuclear regulator of metabolism, differentiation, and cell growth**. *J Biol Chem* 2001, **276**(41):37731-37734.
5. Barak Y, Liao D, He W, Ong ES, Nelson MC, Olefsky JM, Boland R, Evans RM: **Effects of peroxisome proliferator-activated receptor delta on placentation, adiposity, and colorectal cancer**. *Proc Natl Acad Sci U S A* 2002, **99**(1):303-308.
6. Demetri GD, Fletcher CD, Mueller E, Sarraf P, Naujoks R, Campbell N, Spiegelman BM, Singer S: **Induction of solid tumor differentiation by the peroxisome proliferator-activated receptor-gamma ligand troglitazone in patients with liposarcoma**. *Proc Natl Acad Sci U S A* 1999, **96**(7):3951-3956.
7. Mueller E, Smith M, Sarraf P, Kroll T, Aiyer A, Kaufman DS, Oh W, Demetri G, Figg WD, Zhou XP *et al*: **Effects of ligand activation of peroxisome proliferator-activated receptor gamma in human prostate cancer**. *Proc Natl Acad Sci U S A* 2000, **97**(20):10990-10995.
8. Smallridge RC, Copland JA, Brose MS, Wadsworth JT, Houvras Y, Menefee ME, Bible KC, Shah MH, Gramza AW, Klopper JP *et al*: **Efatutazone, an oral PPAR-gamma agonist, in combination with paclitaxel in anaplastic thyroid cancer: results of a multicenter phase 1 trial**. *J Clin Endocrinol Metab* 2013, **98**(6):2392-2400.
9. Kliewer SA, Sundseth SS, Jones SA, Brown PJ, Wisely GB, Koble CS, Devchand P, Wahli W, Willson TM, Lenhard JM *et al*: **Fatty acids and eicosanoids regulate gene expression through direct interactions with peroxisome proliferator-activated receptors alpha and gamma**. *Proc Natl Acad Sci U S A* 1997, **94**(9):4318-4323.
10. Varga T, Czimmerer Z, Nagy L: **PPARs are a unique set of fatty acid regulated transcription factors controlling both lipid metabolism and inflammation**. *Biochim Biophys Acta* 2011, **1812**(8):1007-1022.
11. Grommes C, Karlo JC, Caprariello A, Blankenship D, Dechant A, Landreth GE: **The PPARgamma agonist pioglitazone crosses the blood-brain barrier and reduces tumor growth in a human xenograft model**. *Cancer Chemother Pharmacol* 2013, **71**(4):929-936.
12. Deplanque D, Gelé P, Pétrault O, Six I, Furman C, Bouly M, Nion S, Dupuis B, Leys D, Fruchart J-C *et al*: **Peroxisome Proliferator-Activated Receptor-α Activation as a Mechanism of Preventive Neuroprotection Induced by Chronic Fenofibrate Treatment**. *The Journal of Neuroscience* 2003, **23**(15):6264-6271.
13. Youssef J, Badr M: **Peroxisome proliferator-activated receptors and cancer: challenges and opportunities**. *Br J Pharmacol* 2011, **164**(1):68-82.
14. Herrett E, Gallagher AM, Bhaskaran K, Forbes H, Mathur R, van Staa T, Smeeth L: **Data Resource Profile: Clinical Practice Research Datalink (CPRD)**. *Int J Epidemiol* 2015, **44**(3):827-836.
15. Herrett E, Thomas SL, Schoonen WM, Smeeth L, Hall AJ: **Validation and validity of diagnoses in the General Practice Research Database: a systematic review**. *Br J Clin Pharmacol* 2010, **69**(1):4-14.
16. Herrett E, Shah AD, Boggon R, Denaxas S, Smeeth L, van Staa T, Timmis A, Hemingway H: **Completeness and diagnostic validity of recording acute myocardial infarction events in primary care, hospital care, disease registry, and national mortality records: cohort study**. *BMJ* 2013, **346**:f2350.

For peer review only - http://bmjopen.bmj.com/site/about/guidelines.xhtml

17. Louis DN, Ohgaki H, Wiestler OD, Cavenee WK, Burger PC, Jouvet A, Scheithauer BW, Kleihues P: **The 2007 WHO classification of tumours of the central nervous system**. *Acta Neuropathol* 2007, **114**(2):97-109.
18. Suissa S, Dell'aniello S, Vahey S, Renoux C: **Time-window bias in case-control studies: statins and lung cancer**. *Epidemiology* 2011, **22**(2):228-231.
19. Levesque LE, Hanley JA, Kezouh A, Suissa S: **Problem of immortal time bias in cohort studies: example using statins for preventing progression of diabetes**. *BMJ* 2010, **340**:b5087.
20. Government DoCaL: **The English Indices of Deprivation 2015 Statistical Release**. In. Edited by Government DoCaL; 2015.
21. Pearce N: **Analysis of matched case-control studies**. *BMJ* 2016, **352**:i969.
22. Buuren Sv, Groothuis-Oudshoorn K: **mice: Multivariate Imputation by Chained Equations inR**. *Journal of Statistical Software* 2011, **45**(3).
23. Haynes HR, White P, Hares KM, Redondo J, Kemp KC, Singleton WGB, Killick-Cole CL, Stevens JR, Garadi K, Guglani S *et al*: **The transcription factor PPARalpha is overexpressed and is associated with a favourable prognosis in IDH-wildtype primary glioblastoma**. *Histopathology* 2017, **70**(7):1030-1043.
24. Binello E, Mormone E, Emdad L, Kothari H, Germano IM: **Characterization of fenofibrate-mediated anti-proliferative pro-apoptotic effects on high-grade gliomas and anti-invasive effects on glioma stem cells**. *J Neurooncol* 2014, **117**(2):225-234.
25. Wilk A, Urbanska K, Grabacka M, Mullinax J, Marcinkiewicz C, Impastato D, Estrada JJ, Reiss K: **Fenofibrate-induced nuclear translocation of FoxO3A triggers Bim-mediated apoptosis in glioblastoma cells in vitro**. *Cell Cycle* 2012, **11**(14):2660-2671.
26. Wilk A, Wyczechowska D, Zapata A, Dean M, Mullinax J, Marrero L, Parsons C, Peruzzi F, Culicchia F, Ochoa A *et al*: **Molecular mechanisms of fenofibrate-induced metabolic catastrophe and glioblastoma cell death**. *Mol Cell Biol* 2015, **35**(1):182-198.
27. Grommes C, Conway DS, Alshekhlee A, Barnholtz-Sloan JS: **Inverse association of PPARgamma agonists use and high grade glioma development**. *J Neurooncol* 2010, **100**(2):233-239.
28. Grommes C, Landreth GE, Sastre M, Beck M, Feinstein DL, Jacobs AH, Schlegel U, Heneka MT: **Inhibition of in Vivo Glioma Growth and Invasion by Peroxisome Proliferator-Activated Receptor γ Agonist Treatment**. *Molecular Pharmacology* 2006, **70**(5):1524-1533.
29. Perez-Ortiz JM, Tranque P, Vaquero CF, Domingo B, Molina F, Calvo S, Jordan J, Cena V, Llopis J: **Glitazones differentially regulate primary astrocyte and glioma cell survival. Involvement of reactive oxygen species and peroxisome proliferator-activated receptor-gamma**. *J Biol Chem* 2004, **279**(10):8976-8985.
30. Perez-Ortiz JM, Tranque P, Burgos M, Vaquero CF, Llopis J: **Glitazones induce astroglioma cell death by releasing reactive oxygen species from mitochondria: modulation of cytotoxicity by nitric oxide**. *Mol Pharmacol* 2007, **72**(2):407-417.
31. Panigrahy D, Kaipainen A, Huang S, Butterfield CE, Barnes CM, Fannon M, Laforme AM, Chaponis DM, Folkman J, Kieran MW: **PPARalpha agonist fenofibrate suppresses tumor growth through direct and indirect angiogenesis inhibition**. *Proc Natl Acad Sci U S A* 2008, **105**(3):985-990.
32. Jick SS, Kaye JA, Vasilakis-Scaramozza C, Garcia Rodriguez LA, Ruigomez A, Meier CR, Schlienger RG, Black C, Jick H: **Validity of the general practice research database**. *Pharmacotherapy* 2003, **23**(5):686-689.
33. Tong JJ, Tao H, Hui OT, Jian C: **Diabetes mellitus and risk of brain tumors: A meta-analysis**. *Exp Ther Med* 2012, **4**(5):877-882.
34. Alexandru O, Ene L, Purcaru OS, Tache DE, Popescu A, Neamtu OM, Tataranu LG, Georgescu AM, Tudorica V, Zaharia C *et al*: **Plasma levels of glucose and insulin in patients with brain tumors**. *Curr Health Sci J* 2014, **40**(1):27-36.

For peer review only

For peer review only - http://bmjopen.bmj.com/site/about/guidelines.xhtml

Read Code	Description	CPRD Medical Code	CPRD Description
B506.00	Malignant neoplasm of choroid	15991	Malignant neoplasm of choroid
B51..00	Malignant neoplasm of brain	18617	Malignant neoplasm of brain
B51..11	Cerebral tumour - malignant	10851	Cerebral tumour - malignant
B510.00	Malignant neoplasm cerebrum (excluding lobes and ventricles)	15711	Malignant neoplasm cerebrum (excluding lobes and ventricles)
B510000	Malignant neoplasm of basal ganglia	48073	Malignant neoplasm of basal ganglia
B510100	Malignant neoplasm of cerebral cortex	61399	Malignant neoplasm of cerebral cortex
B510300		99913	Malignant neoplasm of globus pallidus
B510400	Malignant neoplasm of hypothalamus	70942	Malignant neoplasm of hypothalamus
B510500	Malignant neoplasm of thalamus	62126	Malignant neoplasm of thalamus
B510z00	Malignant neoplasm of cerebrum NOS	54133	Malignant neoplasm of cerebrum NOS
B511.00	Malignant neoplasm of frontal lobe	42426	Malignant neoplasm of frontal lobe
B512.00	Malignant neoplasm of temporal lobe	46792	Malignant neoplasm of temporal lobe
B512000	Malignant neoplasm of hippocampus	67236	Malignant neoplasm of hippocampus
B512z00	Malignant neoplasm of temporal lobe NOS	47556	Malignant neoplasm of temporal lobe NOS
B513.00	Malignant neoplasm of parietal lobe	19226	Malignant neoplasm of parietal lobe
B514.00	Malignant neoplasm of occipital lobe	39088	Malignant neoplasm of occipital lobe
B515.00	Malignant neoplasm of cerebral ventricles	52511	Malignant neoplasm of cerebral ventricles
B515000	Malignant neoplasm of choroid plexus	46789	Malignant neoplasm of choroid plexus
B516.00	Malignant neoplasm of cerebellum	45154	Malignant neoplasm of cerebellum
B517.00	Malignant neoplasm of brain stem	44089	Malignant neoplasm of brain stem
B517000	Malignant neoplasm of cerebral peduncle	64557	Malignant neoplasm of cerebral peduncle
B517100	Malignant neoplasm of medulla oblongata	49132	Malignant neoplasm of medulla oblongata
B517200	Malignant neoplasm of midbrain	93537	Malignant neoplasm of midbrain
B517300	Malignant neoplasm of pons	91240	Malignant neoplasm of pons
B517z00	Malignant neoplasm of brain stem NOS	68641	Malignant neoplasm of brain stem NOS
B51y.00	Malignant neoplasm of other parts of brain	71139	Malignant neoplasm of other parts of brain
B51y000	Malignant neoplasm of corpus callosum	59170	Malignant neoplasm of corpus callosum
B51y200	Malignant neoplasm, overlapping lesion of brain	65241	Malignant neoplasm, overlapping lesion of brain
B51yz00		100733	Malignant neoplasm of other part of brain NOS
B51z.00	Malignant neoplasm of brain NOS	41520	Malignant neoplasm of brain NOS
B521.00	Malignant neoplasm of cerebral meninges	28919	Malignant neoplasm of cerebral meninges
B521200		109473	Malignant neoplasm of cerebral pia mater
B521z00	Malignant neoplasm of cerebral meninges NOS	70104	Malignant neoplasm of cerebral meninges NOS
B52X.00	Malignant neoplasm of meninges, unspecified	49875	Malignant neoplasm of meninges, unspecified

For peer review only

B542.00	Malignant neoplasm pituitary gland and craniopharyngeal duct	59823	Malignant neoplasm pituitary gland and craniopharyngeal duct
B542000	Malignant neoplasm of pituitary gland	8550	Malignant neoplasm of pituitary gland
B542z00	Malig neop pituitary gland or craniopharyngeal duct NOS	59718	Malig neop pituitary gland or craniopharyngeal duct NOS
B543.00	Malignant neoplasm of pineal gland	42460	Malignant neoplasm of pineal gland
B583.00	Secondary malignant neoplasm of brain and spinal cord	33843	Secondary malignant neoplasm of brain and spinal cord
B583000	Secondary malignant neoplasm of brain	5198	Secondary malignant neoplasm of brain
B583200	Cerebral metastasis	5199	Cerebral metastasis
B583z00	Secondary malignant neoplasm of brain or spinal cord NOS	59375	Secondary malignant neoplasm of brain or spinal cord NOS
B8yy300	Carcinoma in situ of pituitary gland	45909	Carcinoma in situ of pituitary gland
BA06.00	Neoplasm of unspecified nature of brain	1044	Neoplasm of unspecified nature of brain
BB9G.00	[M]Infiltrating ductular carcinoma	7319	[M]Infiltrating ductular carcinoma
BBa3.00	[M]Pineoblastoma	50151	[M]Pineoblastoma
BBb..00	[M]Gliomas	12309	[M]Gliomas
BBb0.00	[M]Glioma, malignant	31574	[M]Glioma, malignant
BBb0.11	[M]Glioma NOS	8523	[M]Glioma NOS
BBb0.12	[M]Gliosarcoma	34252	[M]Gliosarcoma
BBb1.00	[M]Gliomatosis cerebri	38551	[M]Gliomatosis cerebri
BBb2.00	[M]Mixed glioma	68808	[M]Mixed glioma
BBb2.11	[M]Mixed glioma	39386	[M]Mixed glioma
BBb3.00	[M]Subependymal glioma	94267	[M]Subependymal glioma
BBb3.11	[M]Subependymal astrocytoma NOS	90487	[M]Subependymal astrocytoma NOS
BBb3.12	[M]Subependymal astrocytoma NOS	28344	[M]Subependymal astrocytoma NOS
BBb4.00	[M]Subependymal giant cell astrocytoma	49168	[M]Subependymal giant cell astrocytoma
BBb8.00	[M]Ependymoma, anaplastic type	52751	[M]Ependymoma, anaplastic type
BBb8.11	[M]Ependymoblastoma	46769	[M]Ependymoblastoma
BBbA.00	[M]Myxopapillary ependymoma	43114	[M]Myxopapillary ependymoma
BBba.00	[M]Primitive neuroectodermal tumour	41695	[M]Primitive neuroectodermal tumour
BBbB.00	[M]Astrocytoma NOS	8547	[M]Astrocytoma NOS
BBbB.11	[M]Astrocytic glioma	27748	[M]Astrocytic glioma
BBbC.00	[M]Astrocytoma, anaplastic type	8328	[M]Astrocytoma, anaplastic type
BBbE.00	[M]Gemistocytic astrocytoma	45531	[M]Gemistocytic astrocytoma
BBbF.00	[M]Fibrillary astrocytoma	27846	[M]Fibrillary astrocytoma
BBbG.00	[M]Pilocytic astrocytoma	30273	[M]Pilocytic astrocytoma
BBbG.11	[M]Juvenile astrocytoma	61783	[M]Juvenile astrocytoma
BBbG.12	[M]Piloid astrocytoma	98800	[M]Piloid astrocytoma
BBbH.00		103047	[M]Spongioblastoma NOS
BBbK.00	[M]Astroblastoma	50235	[M]Astroblastoma
BBbL.00	[M]Glioblastoma NOS	23083	[M]Glioblastoma NOS
BBbL.11	[M]Glioblastoma multiforme	9575	[M]Glioblastoma multiforme
BBbM.00	[M]Giant cell glioblastoma	66064	[M]Giant cell glioblastoma
BBbQ.00	[M]Oligodendroglioma NOS	27744	[M]Oligodendroglioma NOS
BBbR.00	[M]Oligodendroglioma, anaplastic type	49186	[M]Oligodendroglioma, anaplastic type

For peer review only

BBbS.00	[M]Oligodendroblastoma	46404	[M]Oligodendroblastoma
BBbT.00	[M]Medulloblastoma NOS	34763	[M]Medulloblastoma NOS
BBbU.00	[M]Desmoplastic medulloblastoma	65952	[M]Desmoplastic medulloblastoma
BBbV.00	[M]Medullomyoblastoma	31767	[M]Medullomyoblastoma
BBbW.00	[M]Cerebellar sarcoma NOS	37473	[M]Cerebellar sarcoma NOS
BBbZ.00	[M]Pleomorphic xanthoastrocytoma	67587	[M]Pleomorphic xanthoastrocytoma
BBbz.00	[M]Glioma NOS	27653	[M]Glioma NOS
BBc6.00	[M]Ganglioglioma	31629	[M]Ganglioglioma
BBc7.11	[M]Neuroastrocytoma	68479	[M]Neuroastrocytoma
BBd1.00	[M]Meningiomatosis NOS	98677	[M]Meningiomatosis NOS
BBd1.11	[M]Diffuse meningiomatosis	95108	[M]Diffuse meningiomatosis
BBd2.00	[M]Meningioma, malignant	27363	[M]Meningioma, malignant
BBd2.11	[M]Leptomeningeal sarcoma	60347	[M]Leptomeningeal sarcoma
BBd2.12	[M]Meningothelial sarcoma	96798	[M]Meningothelial sarcoma
BBd5.00	[M]Psammomatous meningioma	38870	[M]Psammomatous meningioma
BBd7.11	[M]Angioblastic meningioma	46490	[M]Angioblastic meningioma
BBdB.00		106134	[M]Meningeal sarcomatosis
BBdz.00	[M]Meningioma NOS	47848	[M]Meningioma NOS
BBm0.00	[M]Microglioma	63973	[M]Microglioma
ByuA200	[X]Malignant neoplasm of meninges, unspecified	63925	[X]Malignant neoplasm of meninges, unspecified
ByuA300	[X]Malig neopl, overlap lesion brain & other part of CNS	47633	[X]Malig neopl, overlap lesion brain & other part of CNS

Supplemental table 1 Read and CPRD Medical Codes used to identify primary and secondary brain tumours.

For peer review only

Product Code	Product Name	PPARa Targeting?
25	Simvastatin 20mg tablets	No
28	Atorvastatin 10mg tablets	No
42	Simvastatin 10mg tablets	No
51	Simvastatin 40mg tablets	No
75	Atorvastatin 20mg tablets	No
184	Bezafibrate 200mg tablets	Yes
379	Fluvastatin 20mg capsules	No
490	Pravastatin 10mg tablets	No
602	Bezafibrate 400mg modified-release tablets	Yes
644	Colestyramine 4g oral powder sachets	No
653	Ezetimibe 10mg tablets	No
713	Rosuvastatin 10mg tablets	No
730	Pravastatin 20mg tablets	No
745	Atorvastatin 40mg tablets	No
802	Simvador 40mg tablets (Discovery Pharmaceuticals)	No
818	Simvastatin 20mg/5ml oral solution sugar free	No
1212	Colestipol 5g granules sachets sugar free	No
1214	Bezalip 400mg Tablet (Roche Products Ltd)	Yes
1215	Fenofibrate 100mg Capsule	Yes
1217	Lipantil micro 200 200mg Capsule (Fournier Pharmaceuticals Ltd)	Yes
1219	Pravastatin 40mg tablets	No
1221	Lipostat 10mg tablets (Bristol-Myers Squibb Pharmaceuticals Ltd)	No
1223	Lipostat 40mg tablets (Bristol-Myers Squibb Pharmaceuticals Ltd)	No
1322	Clofibrate 500mg capsules	Yes
1324	Bezalip 200mg Tablet (Roche Products Ltd)	Yes
1477	Atromid -s 500mg Capsule (AstraZeneca UK Ltd)	Yes
1716	Questran 4g oral powder sachets (Bristol-Myers Squibb Pharmaceuticals Ltd)	No
1764	Questran Light 4g oral powder sachets (Bristol-Myers Squibb Pharmaceuticals Ltd)	No
2137	Fluvastatin 40mg capsules	No
2215	Lopid 300mg capsules (Pfizer Ltd)	Yes
2435	Lipantil 100mg Capsule (Fournier Pharmaceuticals Ltd)	Yes
2718	Zocor 10mg tablets (Merck Sharp & Dohme Ltd)	No
2955	Lipitor 40mg tablets (Pfizer Ltd)	No
3089	Ciprofibrate 100mg tablets	Yes
3159	Fenofibrate 200mg capsules	Yes
3318	Gemfibrozil 300mg capsules	Yes
3411	Lipitor 10mg tablets (Pfizer Ltd)	No
3690	Lipostat 20mg tablets (Bristol-Myers Squibb Pharmaceuticals Ltd)	No
4062	Lopid 600mg tablets (Pfizer Ltd)	Yes
4067	Olbetam 250mg capsules (Pfizer Ltd)	No
4920	Fenofibrate micronised 200mg capsules	Yes
4928	Lipantil Micro 200 capsules (BGP Products Ltd)	Yes
5148	Simvastatin 80mg tablets	No
5216	Bezalip mono 400mg Modified-release tablet (Roche Products Ltd)	Yes
5390	Fenofibrate micronised 267mg capsules	Yes
5564	Colestid Orange sachets (Pharmacia Ltd)	No
5775	Atorvastatin 80mg tablets	No
5985	Lescol XL 80mg tablets (Novartis Pharmaceuticals UK Ltd)	No
6120	Ezetrol 10mg tablets (Merck Sharp & Dohme Ltd)	No
6155	Colestyramine with aspartame 4g sugar free powder	No
6168	Zocor 40mg tablets (Merck Sharp & Dohme Ltd)	No
6213	Rosuvastatin 20mg tablets	No

For peer review only

6365	Colestid 5g granules sachets plain (Pfizer Ltd)	No
7196	Zocor 20mg tablets (Merck Sharp & Dohme Ltd)	No
7347	Crestor 10mg tablets (AstraZeneca UK Ltd)	No
7374	Lipitor 20mg tablets (Pfizer Ltd)	No
7540	Lipantil Micro 267 capsules (BGP Products Ltd)	Yes
7544	Niaspan 750mg modified-release tablets (Abbott Laboratories Ltd)	No
7551	Niaspan 1g modified-release tablets (Abbott Laboratories Ltd)	No
7552	Simvastatin 20mg / Ezetimibe 10mg tablets	No
7554	Rosuvastatin 5mg tablets	No
8082	Gemfibrozil 600mg tablets	Yes
8104	Acipimox 250mg capsules	No
8380	Lescol 20mg capsules (Novartis Pharmaceuticals UK Ltd)	No
8706	Modalim 100mg tablets (Sanofi)	Yes
9153	Lescol 40mg capsules (Novartis Pharmaceuticals UK Ltd)	No
9491	Fenofibrate micronised 67mg capsules	Yes
9639	Fenofibrate micronised 160mg tablets	Yes
9716	Supralip 160mg tablets (BGP Products Ltd)	Yes
9897	Rosuvastatin 40mg tablets	No
9920	Simvador 20mg tablets (Discovery Pharmaceuticals)	No
9930	Crestor 40mg tablets (AstraZeneca UK Ltd)	No
10172	Simvastatin 40mg / Ezetimibe 10mg tablets	No
10183	Simvastatin 40mg with ezetimibe 10mg tablet	No
10206	Simvastatin 80mg with ezetimibe 10mg tablet	No
11627	Fluvastatin 80mg modified-release tablets	No
11785	Colestyramine 4g oral powder sachets sugar free	No
11815	Simvastatin 20mg with ezetimibe 10mg tablet	No
11976	Niaspan 500mg modified-release tablets (Abbott Laboratories Ltd)	No
12211	Nicotinic acid 50mg tablets	No
13041	Simvador 10mg tablets (Discovery Pharmaceuticals)	No
14219	Simvastatin 80mg / Ezetimibe 10mg tablets	No
14379	Lipantil Micro 67 capsules (BGP Products Ltd)	Yes
14963	Nicotinic acid 500mg modified-release tablets	No
15252	Crestor 20mg tablets (AstraZeneca UK Ltd)	No
16186	Inegy 10mg/80mg tablets (Merck Sharp & Dohme Ltd)	No
17059	Inegy 10mg/40mg tablets (Merck Sharp & Dohme Ltd)	No
17614	Zimbacol XL 400mg tablets (Archimedes Pharma UK Ltd)	Yes
17683	Lipitor 80mg tablets (Pfizer Ltd)	No
17688	Crestor 5mg tablets (AstraZeneca UK Ltd)	No
17813	Nicotinic acid 100mg Tablet	No
17824	Nicotinic acid 25mg Tablet	No
18081	Colestid Orange 5g granules sachets (Pfizer Ltd)	No
18098	Nicotinic acid 375mg + 500mg + 750mg Modified-release tablet	No
18126	Nicotinic acid 1g modified-release tablets	No
19938	Colestipol with aspartame granules	No
21020	Inegy 10mg/20mg tablets (Merck Sharp & Dohme Ltd)	No
22579	Zocor 80mg tablets (Merck Sharp & Dohme Ltd)	No
23153	Liparol 400 XL tablets (Ashbourne Pharmaceuticals Ltd)	Yes

23153	Liparol 400 XL tablets (Ashbourne Pharmaceuticals Ltd)	No
23634	GEMFIBROZIL	Yes
24009	NICOTINIC ACID 500 MG TAB	No
24084	Colestyramine 4g oral powder sachets sugar free (PLIVA Pharma Ltd)	No
24509	SIMVASTATIN	No
24583	Nicotinic acid 750mg modified-release tablets	No
25018	BEZAFIBRATE	Yes
29213	Bezagen XL 400mg tablets (Mylan Ltd)	Yes
29328	Bezafibrate 200mg tablets (A A H Pharmaceuticals Ltd)	Yes
29438	SIMVASTATIN	No
31221	Bezafibrate 200mg tablets (Mylan Ltd)	Yes
31783	Fenogal 200mg capsules (Genus Pharmaceuticals Ltd)	Yes
31930	Zocor heart-pro 10mg Tablet (McNeil Products Ltd)	No
32110	Colestyramine 4g Sachets (Dominion Pharma)	No
32909	Simvastatin 80mg tablets (A A H Pharmaceuticals Ltd)	No
32921	Pravastatin 10mg Tablet (Dr Reddy's Laboratories (UK) Ltd)	No
33082	Simvastatin 20mg tablets (A A H Pharmaceuticals Ltd)	No
33603	Fibrazate XL 400mg tablets (Sandoz Ltd)	Yes
33944	Bezafibrate 200mg tablets (Teva UK Ltd)	Yes
34181	Bezafibrate 400mg Modified-release tablet (Hillcross Pharmaceuticals Ltd)	Yes
34201	Colestyramine 4g oral powder sachets sugar free (Actavis UK Ltd)	No
34277	Gemfibrozil 600mg tablets (Teva UK Ltd)	Yes
34312	Simvastatin 20mg tablets (Mylan Ltd)	No
34316	Simvastatin 20mg tablets (Teva UK Ltd)	No
34353	Simvastatin 40mg tablets (Mylan Ltd)	No
34366	Simvastatin 20mg tablets (IVAX Pharmaceuticals UK Ltd)	No
34376	Simvastatin 40mg tablets (Teva UK Ltd)	No
34381	Simvastatin 40mg tablets (IVAX Pharmaceuticals UK Ltd)	No
34476	Simvastatin 20mg Tablet (Ratiopharm UK Ltd)	No
34481	Simvastatin 10mg tablets (IVAX Pharmaceuticals UK Ltd)	No
34502	Simvastatin 40mg tablets (A A H Pharmaceuticals Ltd)	No
34535	Simvastatin 10mg tablets (Mylan Ltd)	No
34545	Simvastatin 40mg Tablet (Ratiopharm UK Ltd)	No
34560	Simvastatin 10mg Tablet (Ratiopharm UK Ltd)	No
34746	Simvastatin 20mg Tablet (Niche Generics Ltd)	No
34814	Simvastatin 20mg tablets (Wockhardt UK Ltd)	No
34820	Pravastatin 40mg tablets (A A H Pharmaceuticals Ltd)	No
34879	Simvastatin 40mg Tablet (Niche Generics Ltd)	No
34891	Simvastatin 20mg tablets (Kent Pharmaceuticals Ltd)	No
34907	Simvastatin 40mg tablets (Wockhardt UK Ltd)	No
34955	Simvastatin 10mg tablets (A A H Pharmaceuticals Ltd)	No
34969	Simvastatin 40mg tablets (Actavis UK Ltd)	No
36377	Pravastatin 20mg tablets (Teva UK Ltd)	No
37266	Colesevelam 625mg tablets	No
37434	Simvastatin 40mg tablets (Sandoz Ltd)	No
37953	Cholestagel 625mg tablets (Sanofi)	No

For peer review only

39060	Simvastatin 20mg tablets (Dexcel-Pharma Ltd)	No
39420	Bezalip Mono 400mg modified-release tablets (Teva UK Ltd)	Yes
39576	Bezalip 200mg tablets (Teva UK Ltd)	Yes
39652	Simvastatin 40mg/5ml oral solution sugar free	No
39675	Simvastatin 20mg/5ml Oral suspension (Martindale Pharmaceuticals Ltd)	No
39870	Simvador 80mg tablets (Discovery Pharmaceuticals)	No
40340	Simvastatin 10mg tablets (Teva UK Ltd)	No
40382	Pravastatin 20mg tablets (A A H Pharmaceuticals Ltd)	No
40601	Simvastatin 20mg tablets (Ranbaxy (UK) Ltd)	No
40729	Tredaptive 1000mg/20mg modified-release tablets (Merck Sharp & Dohme Ltd)	No
40885	Nicotinic acid 1g / Laropiprant 20mg modified-release tablets	No
41396	Fenofibrate micronised 200mg capsules (A A H Pharmaceuticals Ltd)	Yes
41657	Simvastatin 80mg tablets (Teva UK Ltd)	No
42801	Bezafibrate xl 400mg Modified-release tablet (Generics (UK) Ltd)	Yes
43218	Pravastatin 10mg tablets (Teva UK Ltd)	No
44528	Simvastatin 20mg/5ml oral suspension sugar free (Rosemont Pharmaceuticals Ltd)	No
44650	Simvastatin 40mg tablets (Dexcel-Pharma Ltd)	No
44878	Ranzolont 10mg tablets (Ranbaxy (UK) Ltd)	No
45219	Simvastatin 40mg tablets (Kent Pharmaceuticals Ltd)	No
45235	Simvastatin 20mg tablets (Sandoz Ltd)	No
45245	Simvastatin 20mg tablets (Actavis UK Ltd)	No
45346	Simvastatin 40mg tablets (Arrow Generics Ltd)	No
46878	Simvastatin 40mg tablets (Almus Pharmaceuticals Ltd)	No
46956	Simvastatin 80mg tablets (Arrow Generics Ltd)	No
47023	Omega-3 fish oil with glycerol Emulsion for infusion	No
47065	Atorvastatin 20mg chewable tablets sugar free	No
47090	Atorvastatin 10mg chewable tablets sugar free	No
47630	Lipitor 20mg chewable tablets (Pfizer Ltd)	No
47721	Lipitor 10mg chewable tablets (Pfizer Ltd)	No
47774	Simvastatin 10mg tablets (Arrow Generics Ltd)	No
47935	Fenofibrate 200mg Capsule (Teva UK Ltd)	Yes
47948	Simvastatin 10mg tablets (Tillomed Laboratories Ltd)	No
47988	Pravastatin 40mg tablets (Mylan Ltd)	No
48018	Simvastatin 20mg tablets (Arrow Generics Ltd)	No
48051	Simvastatin 10mg tablets (Kent Pharmaceuticals Ltd)	No
48058	Simvastatin 10mg tablets (Ranbaxy (UK) Ltd)	No
48078	Simvastatin 10mg tablets (Actavis UK Ltd)	No
48097	Pravastatin 40mg tablets (Teva UK Ltd)	No
48221	Simvastatin 20mg/5ml oral suspension sugar free	No
48346	Atorvastatin 60mg tablets	No
48431	Simvastatin 40mg/5ml oral suspension sugar free	No
48518	Atorvastatin 10mg/5ml oral solution	No
48585	Nicotinic acid 500mg capsules	No
48867	Simvastatin 40mg tablets (Alliance Healthcare (Distribution) Ltd)	No

For peer review only

48973	Atorvastatin 30mg tablets	No
49061	Simvastatin 40mg tablets (Bristol Laboratories Ltd)	No
49062	Simvastatin 20mg tablets (Alliance Healthcare (Distribution) Ltd)	No
49558	Atorvastatin 20mg tablets (A A H Pharmaceuticals Ltd)	No
49587	Simvastatin 80mg tablets (Almus Pharmaceuticals Ltd)	No
49609	Bezafibrate 400mg Modified-release tablet (Sandoz Ltd)	Yes
49751	Atorvastatin 40mg tablets (Alliance Healthcare (Distribution) Ltd)	No
50071	Fenofibrate 160mg Tablet (Teva UK Ltd)	Yes
50236	Atorvastatin 10mg tablets (Zentiva)	No
50272	Atorvastatin 40mg tablets (Pfizer Ltd)	No
50483	Simvastatin 40mg tablets (Relonchem Ltd)	No
50564	Simvastatin 20mg tablets (Relonchem Ltd)	No
50670	Simvastatin 40mg tablets (Aurobindo Pharma Ltd)	No
50703	Simvastatin 40mg tablets (Accord Healthcare Ltd)	No
50754	Simvastatin 20mg tablets (Medreich Plc)	No
50788	Atorvastatin 20mg tablets (Pfizer Ltd)	No
50790	Atorvastatin 20mg tablets (Dexcel-Pharma Ltd)	No
50882	Simvastatin 40mg tablets (Somex Pharma)	No
50925	Pravastatin 10mg tablets (Sigma Pharmaceuticals Plc)	No
50963	Atorvastatin 40mg tablets (Teva UK Ltd)	No
51085	Simvastatin 10mg tablets (Medreich Plc)	No
51134	Atorvastatin 10mg tablets (A A H Pharmaceuticals Ltd)	No
51155	Natures Aid Omega-3 Fish Oil 1000mg softgels capsules (Natures Aid Ltd)	No
51166	Simvastatin 40mg tablets (Medreich Plc)	No
51200	Atorvastatin 40mg tablets (Arrow Generics Ltd)	No
51233	Simvastatin 10mg tablets (Alliance Healthcare (Distribution) Ltd)	No
51359	Atorvastatin 20mg tablets (Arrow Generics Ltd)	No
51483	Simvastatin 20mg tablets (Aurobindo Pharma Ltd)	No
51622	Atorvastatin 20mg tablets (Consilient Health Ltd)	No
51676	Pravastatin 40mg tablets (Medreich Plc)	No
51715	Simvastatin 10mg tablets (Sigma Pharmaceuticals Plc)	No
51822	Natures Aid Omega-3 Fish Oil 500mg capsules (Natures Aid Ltd)	No
51876	Atorvastatin 40mg tablets (Consilient Health Ltd)	No
51890	Pravastatin 20mg tablets (Medreich Plc)	No
52097	Atorvastatin 40mg tablets (Wockhardt UK Ltd)	No
52098	Simvastatin 40mg tablets (Ranbaxy (UK) Ltd)	No
52168	Atorvastatin 20mg tablets (Aspire Pharma Ltd)	No
52211	Atorvastatin 20mg tablets (Actavis UK Ltd)	No
52257	Simvastatin 20mg tablets (Accord Healthcare Ltd)	No
52397	Atorvastatin 40mg tablets (Dr Reddy's Laboratories (UK) Ltd)	No
52398	Atorvastatin 40mg tablets (A A H Pharmaceuticals Ltd)	No
52459	Atorvastatin 80mg tablets (Actavis UK Ltd)	No
52460	Atorvastatin 40mg tablets (Aspire Pharma Ltd)	No
52625	Simvastatin 10mg tablets (Wockhardt UK Ltd)	No
52676	Simvastatin 10mg/5ml oral suspension	No
52755	Pravastatin 20mg tablets (Alliance Healthcare (Distribution) Ltd)	No

For peer review only

52812	Simvastatin 20mg tablets (Sigma Pharmaceuticals Plc)	No
52814	Bezafibrate 400mg modified-release tablets (Alliance Healthcare (Distribution) Ltd)	Yes
52821	Atorvastatin 80mg tablets (Dr Reddy's Laboratories (UK) Ltd)	No
52953	Simvastatin 20mg tablets (Bristol Laboratories Ltd)	No
52962	Simvastatin 80mg tablets (Medreich Plc)	No
53087	Simvastatin 20mg tablets (Somex Pharma)	No
53250	Modalim 100mg tablets (Lexon (UK) Ltd)	Yes
53340	Zocor 40mg tablets (Lexon (UK) Ltd)	No
53415	Simvastatin 10mg tablets (Aurobindo Pharma Ltd)	No
53460	Crestor 10mg tablets (DE Pharmaceuticals)	No
53594	Lipitor 80mg tablets (Mawdsley-Brooks & Company Ltd)	No
53676	Simvastatin 20mg tablets (Tillomed Laboratories Ltd)	No
53770	Fluvastatin 40mg capsules (A A H Pharmaceuticals Ltd)	No
53772	Atorvastatin 80mg tablets (Alliance Healthcare (Distribution) Ltd)	No
53822	Simvastatin 10mg tablets (Bristol Laboratories Ltd)	No
53887	Atorvastatin 40mg tablets (Actavis UK Ltd)	No
53890	Atorvastatin 80mg tablets (Pfizer Ltd)	No
53908	Simvastatin 10mg tablets (Dexcel-Pharma Ltd)	No
53966	Simvastatin 40mg tablets (Phoenix Healthcare Distribution Ltd)	No
54240	Simvastatin 40mg tablets (Sigma Pharmaceuticals Plc)	No
54266	Simvastatin 20mg/5ml oral suspension	No
54435	Pravastatin 40mg tablets (Almus Pharmaceuticals Ltd)	No
54493	Simvastatin 10mg tablets (Relonchem Ltd)	No
54535	Atorvastatin 10mg tablets (Pfizer Ltd)	No
54606	Simvastatin 20mg/5ml oral suspension sugar free (A A H Pharmaceuticals Ltd)	No
54607	Pravastatin 20mg tablets (Almus Pharmaceuticals Ltd)	No
54655	Simvastatin 10mg tablets (Accord Healthcare Ltd)	No
54819	Simvastatin 40mg/5ml oral suspension sugar free (Rosemont Pharmaceuticals Ltd)	No
54947	Simvastatin 20mg tablets (Almus Pharmaceuticals Ltd)	No
54976	Simvastatin 10mg tablets (Somex Pharma)	No
54985	Simvastatin 40mg/5ml oral suspension	No
54992	Atorvastatin 10mg/5ml oral suspension	No
55032	Atorvastatin 10mg tablets (Dexcel-Pharma Ltd)	No
55034	Atorvastatin 40mg/5ml oral suspension	No
55444	Atorvastatin 40mg tablets (Zentiva)	No
55452	Simvastatin 20mg tablets (Phoenix Healthcare Distribution Ltd)	No
55727	Atorvastatin 10mg tablets (Actavis UK Ltd)	No
55912	Pravastatin 40mg tablets (Alliance Healthcare (Distribution) Ltd)	No
56065	Simvastatin 20mg/5ml oral suspension sugar free (Waymade Healthcare Plc)	No
56097	Atorvastatin 10mg chewable tablets sugar free	No
56146	Pravastatin 10mg tablets (Waymade Healthcare Plc)	No

56165	Atorvastatin 20mg chewable tablets sugar free	No
56182	Atorvastatin 80mg tablets (Zentiva)	No
56248	Atorvastatin 20mg tablets (Sigma Pharmaceuticals Plc)	No
56481	Zocor 10mg tablets (Sigma Pharmaceuticals Plc)	No
56494	Zocor 20mg tablets (Sigma Pharmaceuticals Plc)	No
56564	Atorvastatin 20mg tablets (Almus Pharmaceuticals Ltd)	No
56607	Pravastatin 20mg tablets (Waymade Healthcare Plc)	No
56735	Pravastatin 20mg tablets (Mylan Ltd)	No
56841	Atorvastatin 40mg tablets (Dexcel-Pharma Ltd)	No
56893	Pravastatin 40mg tablets (Accord Healthcare Ltd)	No
56916	Pravastatin 40mg tablets (PLIVA Pharma Ltd)	No
57108	Pravastatin 40mg tablets (Waymade Healthcare Plc)	No
57117	Atorvastatin 80mg tablets (Waymade Healthcare Plc)	No
57137	Pravastatin 10mg tablets (Almus Pharmaceuticals Ltd)	No
57219	Fenofibrate micronised 200mg capsules (Sandoz Ltd)	Yes
57296	Pravastatin 20mg tablets (Phoenix Healthcare Distribution Ltd)	No
57329	Simvastatin 25mg/5ml oral suspension	No
57348	Atorvastatin 10mg tablets (Consilient Health Ltd)	No
57397	Pravastatin 10mg tablets (Accord Healthcare Ltd)	No
57489	Ciprofibrate 100mg tablets (Zentiva)	Yes
57568	Zocor 10mg tablets (Lexon (UK) Ltd)	No
57763	Rosuvastatin 10mg tablets (Waymade Healthcare Plc)	No
57834	Atorvastatin 40mg tablets (DE Pharmaceuticals)	No
57836	Atorvastatin 80mg tablets (Teva UK Ltd)	No
57999	Crestor 40mg tablets (Lexon (UK) Ltd)	No
58041	Atorvastatin 20mg tablets (Teva UK Ltd)	No
58110	Atorvastatin 20mg tablets (Zentiva)	No
58315	Simvastatin 20mg tablets (Waymade Healthcare Plc)	No
58394	Atorvastatin 20mg tablets (Alliance Healthcare (Distribution) Ltd)	No
58418	Atorvastatin 80mg tablets (A A H Pharmaceuticals Ltd)	No
58617	Rosuvastatin 20mg/5ml oral suspension	No
58635	Bezalip Mono 400mg modified-release tablets (DE Pharmaceuticals)	Yes
58742	Atorvastatin 80mg tablets (Arrow Generics Ltd)	No
58755	Simvastatin 10mg tablets (Phoenix Healthcare Distribution Ltd)	No
58834	Atorvastatin 10mg tablets (DE Pharmaceuticals)	No
58868	Atorvastatin 10mg tablets (Sigma Pharmaceuticals Plc)	No
59002	Bezafibrate 400mg modified-release tablets (DE Pharmaceuticals)	Yes
59272	Atorvastatin 20mg tablets (Waymade Healthcare Plc)	No
59278	Fluvastatin 20mg capsules (Zentiva)	No
59331	Lipitor 10mg tablets (DE Pharmaceuticals)	No
59357	Atorvastatin 10mg tablets (Ranbaxy (UK) Ltd)	No
59446	Atorvastatin 40mg tablets (Almus Pharmaceuticals Ltd)	No

For peer review only

59447	Crestor 20mg tablets (Waymade Healthcare Plc)	No
59452	Rosuvastatin 5mg tablets (Waymade Healthcare Plc)	No
59508	Pravastatin 20mg tablets (Accord Healthcare Ltd)	No
59776	Atorvastatin 80mg tablets (Aspire Pharma Ltd)	No
59859	Atorvastatin 10mg tablets (Teva UK Ltd)	No
60101	Colestyramine 4g oral powder sachets sugar free (Teva UK Ltd)	No
60160	Rosuvastatin 5mg tablets (Mawdsley-Brooks & Company Ltd)	No
60251	Pravastatin 10mg tablets (Sandoz Ltd)	No
60342	Berocca effervescent tablets tropical (Bayer Plc)	No
60385	Bezalip Mono 400mg modified-release tablets (Lexon (UK) Ltd)	Yes
60464	Atorvastatin 20mg/5ml oral suspension	No
60511	Atorvastatin 40mg tablets (Ranbaxy (UK) Ltd)	No
60607	Atorvastatin 80mg tablets (DE Pharmaceuticals)	No
60788	Fenofibrate micronised 267mg capsules (Zentiva)	Yes
60989	Atorvastatin 80mg tablets (Phoenix Healthcare Distribution Ltd)	No
61087	Questran Light 4g oral powder sachets (Mawdsley-Brooks & Company Ltd)	No
61134	Pravastatin 20mg tablets (Sigma Pharmaceuticals Plc)	No
61149	Atorvastatin 10mg tablets (Waymade Healthcare Plc)	No
61155	Simvastatin 40mg/5ml oral suspension sugar free (A A H Pharmaceuticals Ltd)	No
61321	Simvastatin 10mg tablets (Sandoz Ltd)	No
61360	Simvastatin 10mg tablets (Almus Pharmaceuticals Ltd)	No
61665	Simvastatin 10mg tablets (Waymade Healthcare Plc)	No
62137	Simvastatin 40mg tablets (Waymade Healthcare Plc)	No
62148	Fluvastatin 20mg capsules (Actavis UK Ltd)	No
62219	Atorvastatin 20mg tablets (DE Pharmaceuticals)	No
62429	Atorvastatin 20mg tablets (DE Pharmaceuticals)	No
62476	Atorvastatin 80mg tablets (Almus Pharmaceuticals Ltd)	No
62979	Pravastatin 40mg tablets (Kent Pharmaceuticals Ltd)	No
63074	Pravastatin 20mg tablets (PLIVA Pharma Ltd)	No
63140	Atorvastatin 10mg tablets (Alliance Healthcare (Distribution) Ltd)	No
63249	Atorvastatin 80mg tablets (Consilient Health Ltd)	No
63343	Generic Crampex tablets	No
63469	Atorvastatin 30mg tablets (Consilient Health Ltd)	No
63737	Fenofibrate micronised 267mg capsules (Sigma Pharmaceuticals Plc)	Yes
63787	Pravastatin 10mg tablets (Tillomed Laboratories Ltd)	No
64067	Atorvastatin 20mg/5ml oral solution	No
64104	Simvastatin 20mg tablets (Crescent Pharma Ltd)	No
64180	Simvastatin 10mg tablets (Crescent Pharma Ltd)	No
64307	Simvastatin 40mg tablets (Crescent Pharma Ltd)	No
64503	Bezalip Mono 400mg modified-release tablets (Waymade Healthcare Plc)	Yes
64702	Atorvastatin 30mg tablets (A A H Pharmaceuticals Ltd)	No

64810	Atorvastatin 40mg tablets (Phoenix Healthcare Distribution Ltd)	No
64825	Atorvastatin 10mg tablets (Phoenix Healthcare Distribution Ltd)	No
64868	Atorvastatin 40mg tablets (Sigma Pharmaceuticals Plc)	No
64933	Fenofibrate micronised 267mg capsules (Ranbaxy (UK) Ltd)	Yes
64968	Simvastatin 10mg tablets (DE Pharmaceuticals)	No
64984	Fenofibrate micronised 160mg tablets (Phoenix Healthcare Distribution Ltd)	Yes
65181	Simvastatin 40mg tablets (DE Pharmaceuticals)	No
65193	Atorvastatin 20mg tablets (Ranbaxy (UK) Ltd)	No
65572	Fenofibrate micronised 160mg tablets (Genus Pharmaceuticals Ltd)	Yes
65679	Simvastatin 20mg tablets (DE Pharmaceuticals)	No
65901	Simvastatin 40mg tablets (Zentiva)	No
65925	Simvastatin 20mg/5ml oral suspension sugar free (Alliance Healthcare (Distribution) Ltd)	No
66087	Modalim 100mg tablets (Mawdsley-Brooks & Company Ltd)	Yes
66425	Bezafibrate 400mg modified-release tablets (A A H Pharmaceuticals Ltd)	Yes
66505	Fenofibrate 145mg / Simvastatin 40mg tablets	Yes
66564	Bezafibrate 400mg modified-release tablets (Phoenix Healthcare Distribution Ltd)	Yes
66780	Fenofibrate 145mg / Simvastatin 20mg tablets	Yes
66963	Atorvastatin 80mg tablets (Sigma Pharmaceuticals Plc)	No
67098	Simvastatin 10mg tablets (Brown & Burk UK Ltd)	No

67157	Fenofibrate micronised 200mg capsules (Phoenix Healthcare Distribution Ltd)	Yes
67328	Lescol XL 80mg tablets (Mawdsley-Brooks & Company Ltd)	No
67329	Lipantil Micro 267 capsules (DE Pharmaceuticals)	Yes
67402	Atorvastatin 40mg tablets (Kent Pharmaceuticals Ltd)	No
67573	Atorvastatin 10mg tablets (DE Pharmaceuticals)	No
67660	Atorvastatin 80mg tablets (Ranbaxy (UK) Ltd)	No
67745	Simvastatin 10mg tablets (Zentiva)	No
67773	Simvastatin 20mg tablets (Zentiva)	No
67829	Pravastatin 20mg tablets (Sandoz Ltd)	No
67846	Atorvastatin 10mg tablets (Almus Pharmaceuticals Ltd)	No
67883	Lomitapide 5mg capsules	No
68023	Atorvastatin 10mg tablets (Aspire Pharma Ltd)	No
68048	Atorvastatin 20mg tablets (Phoenix Healthcare Distribution Ltd)	No
68156	Pravastatin 10mg tablets (A A H Pharmaceuticals Ltd)	No
68386	Colestyramine 4g oral powder sachets (J M McGill Ltd)	No
68467	Atorvastatin 20mg tablets (Kent Pharmaceuticals Ltd)	No
68563	Simvastatin 40mg tablets (Brown & Burk UK Ltd)	No
68686	Simvastatin 20mg tablets (Genesis Pharmaceuticals Ltd)	No
68785	Atorvastatin 10mg tablets (Mylan Ltd)	No
68827	Atorvastatin 20mg tablets (Mylan Ltd)	No
69093	Atorvastatin 80mg tablets (Wockhardt UK Ltd)	No
69413	Simvastatin 20mg tablets (Brown & Burk UK Ltd)	No

For peer review only

69427	Atorvastatin 40mg tablets (Mylan Ltd)	No

Supplemental table 2: Product codes for drugs used for hyperlipidaemia and fibrate exposure status.

For peer review only

Product Code	Product Name	PPARg Targeting?
23	Metformin 500mg tablets	No
32	Gliclazide 80mg tablets	No
93	Metformin 850mg tablets	No
240	Rifampicin 150mg capsules	No
469	Rosiglitazone 4mg tablets	Yes
479	Acarbose 50mg tablets	No
547	Glipizide 2.5mg tablets	No
548	Pioglitazone 15mg tablets	Yes
735	Metformin 100mg/ml Oral solution	No
1254	Glibenclamide 5mg tablets	No
1964	Diamicron 80mg tablets (Servier Laboratories Ltd)	No
1965	Tolbutamide 500mg tablets	No
2219	Glibenclamide 2.5mg tablets	No
4862	Diabetamide 2.5mg tablets (Ashbourne Pharmaceuticals Ltd)	No
5174	Acarbose 100mg tablets	No
5227	Rosiglitazone 8mg tablets	Yes
5276	Glimepiride 1mg tablets	No
5316	Glimepiride 4mg tablets	No
5353	Glimepiride 2mg tablets	No
5621	Glucobay 50mg tablets (Bayer Plc)	No
5627	Gliclazide 30mg modified-release tablets	No
5636	Glipizide 5mg tablets	No
5678	Nateglinide 120mg tablets	No
5989	Nateglinide 180mg tablets	No
6337	Glimepiride 3mg tablets	No
6855	Avandamet 2mg/500mg tablets (GlaxoSmithKline UK Ltd)	Yes
7048	Metformin 500mg modified-release tablets	No
7166	Glucophage 500mg tablets (Merck Serono Ltd)	No
7284	Amaryl 2mg tablets (Zentiva)	No
7325	Avandamet 4mg/1000mg tablets (GlaxoSmithKline UK Ltd)	Yes
7332	Amaryl 1mg tablets (Zentiva)	No
7375	Rosiglitazone 4mg / Metformin 1g tablets	Yes
7409	Amaryl 3mg tablets (Zentiva)	No
7610	Glucophage 850mg tablets (Merck Serono Ltd)	No
7744	Daonil 5mg tablets (Sanofi)	No
7818	Rifater tablets (Sanofi)	No
7912	Semi-Daonil 2.5mg tablets (Sanofi)	No
8976	Euglucon 2.5mg tablets (Aventis Pharma)	No
9105	Glucobay 100mg tablets (Bayer Plc)	No
9662	Avandia 4mg tablets (GlaxoSmithKline UK Ltd)	Yes
9691	Rifampicin with isoniazid & pyrazinamide tablet	No
9699	Pioglitazone 30mg tablets	Yes
9707	Repaglinide 1mg tablets	No
9748	Repaglinide 2mg tablets	No
9865	Repaglinide 500microgram tablets	No
10051	Pioglitazone 45mg tablets	Yes
11284	Amaryl 4mg tablets (Zentiva)	No
11316	NovoNorm 500microgram tablets (Novo Nordisk Ltd)	No
11321	NovoNorm 1mg tablets (Novo Nordisk Ltd)	No
11366	NovoNorm 2mg tablets (Novo Nordisk Ltd)	No
11483	Nateglinide 60mg tablets	No
11601	Rosiglitazone 2mg / Metformin 500mg tablets	Yes
11604	Rosiglitazone 1mg / Metformin 500mg tablets	Yes
11609	Metformin with rosiglitazone 500mg + 1mg Tablet	Yes
11610	Metformin with rosiglitazone 500mg + 2mg Tablet	Yes
11695	Diamicron 30mg MR tablets (Servier Laboratories Ltd)	No
11717	Rosiglitazone 2mg / Metformin 1g tablets	Yes
11737	Metformin with rosiglitazone 1000mg + 4mg Tablet	Yes
11760	Metformin with rosiglitazone 1000mg + 2mg Tablet	Yes
11946	Tolbutamide 50mg/ml Injection	No
11990	Metformin 500mg/5ml oral solution sugar free	No
12455	Rastinon 500mg Tablet (Hoechst Marion Roussel)	No
12513	Glibenese 5mg tablets (Pfizer Ltd)	No
13331	Euglucon 5mg tablets (Sanofi)	No

For peer review only

13628	Romozin 400mg Tablet (Glaxo Wellcome UK Ltd)	Yes
14164	Avandamet 2mg/1000mg tablets (GlaxoSmithKline UK Ltd)	Yes
15232	Avandia 8mg tablets (GlaxoSmithKline UK Ltd)	Yes
15374	Gliclazide 40mg/5ml oral suspension	No
15955	Starlix 120mg tablets (Novartis Pharmaceuticals UK Ltd)	No
16044	Glucophage SR 500mg tablets (Merck Serono Ltd)	No
16602	Calabren 2.5mg Tablet (Berk Pharmaceuticals Ltd)	No
17343	Gliclazide 80mg tablets (A A H Pharmaceuticals Ltd)	No
17580	Avandamet 1mg/500mg tablets (GlaxoSmithKline UK Ltd)	Yes
17698	Minodiab 5mg tablets (Pfizer Ltd)	No
17706	Minodiab 2.5mg tablets (Pfizer Ltd)	No
17770	Glucagon novo 10mg Injection (Novo Nordisk Ltd)	No
18220	Pioglitazone 15mg / Metformin 850mg tablets	Yes
19472	Actos 45mg tablets (Takeda UK Ltd)	Yes
20287	Actos 15mg tablets (Takeda UK Ltd)	Yes
20889	Actos 30mg tablets (Takeda UK Ltd)	Yes
21424	Glibenclamide 5mg/5ml oral suspension	No
21564	Gliclazide 80mg tablets (Wockhardt UK Ltd)	No
21832	Diabetamide 5mg tablets (Ashbourne Pharmaceuticals Ltd)	No
21892	Diaglyk 80mg tablets (Ashbourne Pharmaceuticals Ltd)	No
22239	Glucagon lilly 1mg Injection (Eli Lilly and Company Ltd)	No
23945	Starlix 60mg tablets (Novartis Pharmaceuticals UK Ltd)	No
25636	Libanil 2.5mg Tablet (Approved Prescription Services Ltd)	No
25678	Glucamet 500mg Tablet (Opus Pharmaceuticals Ltd)	No
26218	Calabren 5mg Tablet (Berk Pharmaceuticals Ltd)	No
26258	Glucamet 850mg Tablet (Opus Pharmaceuticals Ltd)	No
27125	Starlix 180mg tablets (Novartis Pharmaceuticals UK Ltd)	No
27501	Orabet 500mg Tablet (Lagap)	No
28708	Malix 2.5mg Tablet (Lagap)	No
29326	Glipizide 5mg tablets (Mylan Ltd)	No
29939	Gliclazide 80mg tablets (Mylan Ltd)	No
30316	Metformin with pioglitazone 850mg + 15mg Tablet	Yes
30460	Malix 5mg Tablet (Lagap)	No
31077	Competact 15mg/850mg tablets (Takeda UK Ltd)	Yes
31146	Metsol 500mg/5ml oral solution (Kappin Ltd)	No
31212	Gliclazide 80mg tablets (Actavis UK Ltd)	No
31474	Libanil 5mg Tablet (Approved Prescription Services Ltd)	No
33087	Metformin 500mg tablets (Actavis UK Ltd)	No
33562	Duclazide 80mg Tablet (Dumex Ltd)	No
33673	Tolbutamide 500mg tablets (Actavis UK Ltd)	No
33674	Metformin 850mg tablets (A A H Pharmaceuticals Ltd)	No
34004	Metformin 500mg tablets (IVAX Pharmaceuticals UK Ltd)	No
34020	Metformin 850mg tablets (IVAX Pharmaceuticals UK Ltd)	No
34135	Metformin 500mg Tablet (M & A Pharmachem Ltd)	No
34323	Metformin 500mg tablets (A A H Pharmaceuticals Ltd)	No
34399	Gliclazide 80mg tablets (IVAX Pharmaceuticals UK Ltd)	No
34504	Metformin 500mg tablets (Wockhardt UK Ltd)	No
34507	Glibenclamide 2.5mg tablets (Wockhardt UK Ltd)	No
34563	Glibenclamide 5mg tablets (Wockhardt UK Ltd)	No
34598	Metformin 500mg tablets (Mylan Ltd)	No

For peer review only

34676	Glibenclamide 2.5mg tablets (A A H Pharmaceuticals Ltd)	No
34697	Metformin 850mg tablets (Wockhardt UK Ltd)	No
34706	Glibenclamide 2.5mg tablets (IVAX Pharmaceuticals UK Ltd)	No
34742	Metformin 850mg tablets (Teva UK Ltd)	No
34802	Glipizide 5mg tablets (IVAX Pharmaceuticals UK Ltd)	No
34836	Metformin 850mg tablets (Actavis UK Ltd)	No
34917	Metformin 500mg tablets (Teva UK Ltd)	No
34932	Gliclazide 80mg tablets (Genus Pharmaceuticals Ltd)	No
34957	Tolbutamide 500mg tablets (A A H Pharmaceuticals Ltd)	No
35022	Sitagliptin 100mg tablets	No
35144	Byetta 5micrograms/0.02ml solution for injection 1.2ml pre-filled disposable devices (AstraZeneca UK Ltd)	No
35149	Exenatide 10micrograms/0.04ml solution for injection 2.4ml pre-filled disposable devices	No
35150	Byetta 10micrograms/0.04ml solution for injection 2.4ml pre-filled disposable devices (AstraZeneca UK Ltd)	No
35251	Exenatide 5micrograms/0.02ml solution for injection 1.2ml pre-filled disposable devices	No
35462	Januvia 100mg tablets (Merck Sharp & Dohme Ltd)	No
35561	Prandin 2mg tablets (Novo Nordisk Ltd)	No
36774	Prandin 1mg tablets (Novo Nordisk Ltd)	No
36856	Gliclazide 80mg tablets (Sandoz Ltd)	No
36948	Prandin 0.5mg tablets (Novo Nordisk Ltd)	No
37617	Rosiglitazone 2mg tablet	Yes
37874	Vildagliptin 50mg / Metformin 850mg tablets	No
37875	Vildagliptin 50mg tablets	No
37902	Vildagliptin 50mg / Metformin 1g tablets	No
38355	Metformin 750mg modified-release tablets	No
38400	Glucophage SR 750mg tablets (Merck Serono Ltd)	No
38551	Eucreas 50mg/1000mg tablets (Novartis Pharmaceuticals UK Ltd)	No
39149	Galvus 50mg tablets (Novartis Pharmaceuticals UK Ltd)	No
39203	Eucreas 50mg/850mg tablets (Novartis Pharmaceuticals UK Ltd)	No
39560	Bolamyn SR 500mg tablets (Teva UK Ltd)	No
39598	Metformin 1g modified-release tablets	No
39729	Glucophage SR 1000mg tablets (Merck Serono Ltd)	No
39988	Metformin 500mg oral powder sachets sugar free	No
40007	Glucophage 1000mg oral powder sachets (Merck Serono Ltd)	No
40110	Glucophage 500mg oral powder sachets (Merck Serono Ltd)	No
40233	Metformin 1g oral powder sachets sugar free	No
40365	Glimepiride 1mg tablets (Actavis UK Ltd)	No
40425	Nazdol MR 30mg tablets (Teva UK Ltd)	No
40642	Victoza 6mg/ml solution for injection 3ml pre-filled pen (Novo Nordisk Ltd)	No
40693	Liraglutide 6mg/ml solution for injection 3ml pre-filled disposable devices	No
41204	Saxagliptin 5mg tablets	No
41431	Onglyza 5mg tablets (AstraZeneca UK Ltd)	No
41558	Glibenclamide 5mg tablets (Teva UK Ltd)	No
41559	Glibenclamide 5mg tablets (A A H Pharmaceuticals Ltd)	No
41593	Glibenclamide 2.5mg tablets (Teva UK Ltd)	No
42161	Orabet 500mg Tablet (Sandoz Ltd)	No
42790	Gliclazide 80mg Tablet (Merck Generics (UK) Ltd)	No
43065	Gliclazide 40mg tablets	No

For peer review only

43270	Metformin 500mg/5ml oral solution sugar free (Rosemont Pharmaceuticals Ltd)	No
43465	Zicron 40mg tablets (Bristol Laboratories Ltd)	No
43619	Metformin 1g / Sitagliptin 50mg tablets	No
43684	Janumet 50mg/1000mg tablets (Merck Sharp & Dohme Ltd)	No
44250	Metformin 500mg/5ml Oral solution (Hillcross Pharmaceuticals Ltd)	No
44304	Glyconon 500mg Tablet (DDSA Pharmaceuticals Ltd)	No
44473	Edicil MR 30mg tablets (Teva UK Ltd)	No
44738	Niddaryl 1mg tablets (Dee Pharmaceuticals Ltd)	No
45215	Gliclazide 80mg Tablet (Neo Laboratories Ltd)	No
45581	Metabet SR 500mg tablets (Morningside Healthcare Ltd)	No
45775	Saxagliptin 2.5mg tablets	No
45821	Onglyza 2.5mg tablets (AstraZeneca UK Ltd)	No
45831	Dacadis MR 30mg tablets (Mylan Ltd)	No
46458	Exenatide 2mg powder and solvent for prolonged-release suspension for injection vials	No
46469	Bydureon 2mg powder and solvent for prolonged-release suspension for injection vials (AstraZeneca UK Ltd)	No
46665	Linagliptin 5mg tablets	No
46716	Trajenta 5mg tablets (Boehringer Ingelheim Ltd)	No
46927	Tolbutamide 500mg tablets (Teva UK Ltd)	No
46989	Metabet SR 1000mg tablets (Morningside Healthcare Ltd)	No
47074	Gliclazide 80mg/5ml oral suspension	No
47894	Nazdol MR 30mg tablets (Consilient Health Ltd)	No
47939	Glucient SR 500mg tablets (Consilient Health Ltd)	No
48056	Gliclazide 80mg tablets (Sovereign Medical Ltd)	No
48139	Pioglitazone 30mg tablets (A A H Pharmaceuticals Ltd)	Yes
48149	Metformin 500mg tablets (Almus Pharmaceuticals Ltd)	No
48401	Sitagliptin 50mg tablets	No
48533	Sitagliptin 25mg tablets	No
49502	Glucophage SR 500mg tablets (Mawdsley-Brooks & Company Ltd)	No
49738	Metformin 1g modified-release tablets (A A H Pharmaceuticals Ltd)	No
50087	Januvia 50mg tablets (Merck Sharp & Dohme Ltd)	No
50124	Januvia 25mg tablets (Merck Sharp & Dohme Ltd)	No
50570	Glucophage SR 500mg tablets (Lexon (UK) Ltd)	No
50821	Metformin 850mg tablets (Pfizer Ltd)	No
50970	Metformin 500mg tablets (Bristol Laboratories Ltd)	No
51080	Metabet SR 1000mg tablets (Actavis UK Ltd)	No
51135	Metformin 500mg modified-release tablets (A A H Pharmaceuticals Ltd)	No
51527	Metformin 500mg tablets (Boston Healthcare Ltd)	No
51955	Gliclazide 80mg tablets (Accord Healthcare Ltd)	No
52203	Enyglid 0.5mg tablets (Consilient Health Ltd)	No
52221	Diagemet XL 500mg tablets (Genus Pharmaceuticals Ltd)	No
52442	Metformin 500mg tablets (Pfizer Ltd)	No
52634	Glucophage SR 500mg tablets (DE Pharmaceuticals)	No
53288	Gliclazide 30mg modified-release tablets (A A H Pharmaceuticals Ltd)	No
53478	Metformin 500mg modified-release tablets (Kent Pharmaceuticals Ltd)	No
53774	Metabet SR 500mg tablets (Actavis UK Ltd)	No
53867	Metformin 500mg tablets (Zentiva)	No
54150	Jentadueto 2.5mg/850mg tablets (Boehringer Ingelheim Ltd)	No
54182	Dapagliflozin 10mg tablets	No

For peer review only

54203	Forxiga 10mg tablets (AstraZeneca UK Ltd)	No
54265	Dapagliflozin 5mg tablets	No
54442	Metformin (roi) 1000mg Tablet	No
54480	Forxiga 5mg tablets (AstraZeneca UK Ltd)	No
54764	Gliclazide 80mg tablets (Arrow Generics Ltd)	No
54891	Saxagliptin 2.5mg / Metformin 1g tablets	No
54898	Metformin 850mg tablets (Almus Pharmaceuticals Ltd)	No
55270	Duformin 500mg Tablet (Dumex Ltd)	No
55711	Metformin 500mg tablets (Alliance Healthcare (Distribution) Ltd)	No
55739	Metformin 500mg tablets (Tillomed Laboratories Ltd)	No
55862	Gliclazide Oral solution	No
56008	Gliclazide 80mg tablets (Almus Pharmaceuticals Ltd)	No
56208	Pioglitazone 15mg tablets (A A H Pharmaceuticals Ltd)	Yes
56376	Rosiglitazone 4mg with glimepiride 4mg tablet	No
56437	Gliclazide 60mg modified-release tablets	No
56965	Komboglyze 2.5mg/1000mg tablets (AstraZeneca UK Ltd)	No
57147	Bolamyn SR 1000mg tablets (Teva UK Ltd)	No
57457	Metformin 500mg tablets (Aurobindo Pharma Ltd)	No
57601	Daonil 5mg tablets (Dowelhurst Ltd)	No
57659	Pioglitazone 30mg tablets (Actavis UK Ltd)	Yes
57830	Gliclazide 30mg modified-release tablets (Alliance Healthcare (Distribution) Ltd)	No
58051	Metformin 500mg/5ml oral solution	No
58607	Metformin 500mg/5ml oral solution sugar free (Zentiva)	No
59620	Glucophage SR 500mg tablets (Waymade Healthcare Plc)	No
59809	Alogliptin 6.25mg tablets	No
60012	Dapagliflozin 5mg / Metformin 1g tablets	No
60074	Metformin 1g modified-release tablets (Waymade Healthcare Plc)	No
60211	Canagliflozin 100mg tablets	No
60286	Metformin 500mg/5ml oral suspension	No
60430	Invokana 100mg tablets (Janssen-Cilag Ltd)	No
60495	Gliclazide 80mg tablets (Teva UK Ltd)	No
60643	Xigduo 5mg/1000mg tablets (AstraZeneca UK Ltd)	No
60968	Metformin 500mg modified-release tablets (Actavis UK Ltd)	No
61043	Sukkarto SR 1000mg tablets (Morningside Healthcare Ltd)	No
61311	Glimepiride 4mg tablets (Sigma Pharmaceuticals Plc)	No
61559	Sukkarto SR 500mg tablets (Morningside Healthcare Ltd)	No
61925	NovoNorm 500microgram tablets (Waymade Healthcare Plc)	No
61957	Gliclazide 40mg tablets (A A H Pharmaceuticals Ltd)	No
62014	Glimepiride 2mg tablets (Accord Healthcare Ltd)	No
62034	Laaglyda MR 60mg tablets (Consilient Health Ltd)	No
62144	Metformin 500mg modified-release tablets (DE Pharmaceuticals)	No
62265	Metformin 500mg modified-release tablets (Mawdsley-Brooks & Company Ltd)	No
62326	Vipidia 6.25mg tablets (Takeda UK Ltd)	No
62426	Pioglitazone 30mg tablets (Accord Healthcare Ltd)	Yes
62426	Pioglitazone 30mg tablets (Accord Healthcare Ltd)	No
62605	Metformin 850mg tablets (Kent Pharmaceuticals Ltd)	No
62661	Bydureon 2mg powder and solvent for prolonged-release suspension for injection pre-filled pen (AstraZeneca UK Ltd)	No
62824	Metformin 1g modified-release tablets (Actavis UK Ltd)	No
63045	Metformin 850mg tablets (Relonchem Ltd)	No

For peer review only

63046	Pioglitazone 45mg tablets (A A H Pharmaceuticals Ltd)	Yes
63048	Gliclazide 80mg tablets (Alliance Healthcare (Distribution) Ltd)	No
63107	Pioglitazone 45mg tablets (Waymade Healthcare Plc)	Yes
63131	Ziclaseg 30mg modified-release tablets (Lupin (Europe) Ltd)	No
63336	Trulicity 1.5mg/0.5ml solution for injection pre-filled pen (Eli Lilly and Company Ltd)	No
63401	Trulicity 0.75mg/0.5ml solution for injection pre-filled pen (Eli Lilly and Company Ltd)	No
63421	Pioglitazone 30mg tablets (Teva UK Ltd)	Yes
63516	Forxiga 10mg tablets (Waymade Healthcare Plc)	No
63785	Dulaglutide 0.75mg/0.5ml solution for injection pre-filled disposable devices	No
63823	Dulaglutide 1.5mg/0.5ml solution for injection pre-filled disposable devices	No
64217	Jardiance 25mg tablets (Boehringer Ingelheim Ltd)	No
64900	Glidipion 30mg tablets (Actavis UK Ltd)	Yes
64939	Glucient SR 1000mg tablets (Consilient Health Ltd)	No
65083	Synjardy 5mg/1000mg tablets (Boehringer Ingelheim Ltd)	No
65344	Empagliflozin 5mg / Metformin 850mg tablets	No
65562	Pioglitazone 30mg tablets (Alliance Healthcare (Distribution) Ltd)	Yes
65563	Pioglitazone 15mg tablets (Alliance Healthcare (Distribution) Ltd)	Yes
65694	Metformin 500mg modified-release tablets (Waymade Healthcare Plc)	No
65923	Metformin 1g modified-release tablets (Mawdsley-Brooks & Company Ltd)	No
66008	Synjardy 12.5mg/1000mg tablets (Boehringer Ingelheim Ltd)	No
66136	Glucophage SR 1000mg tablets (Waymade Healthcare Plc)	No
66399	Glimepiride 2mg tablets (A A H Pharmaceuticals Ltd)	No
67056	Amaryl 1mg tablets (Lexon (UK) Ltd)	No
67781	Gliclazide 80mg tablets (Milpharm Ltd)	No
68203	Metformin 500mg modified-release tablets (Almus Pharmaceuticals Ltd)	No
68214	Metformin 500mg/5ml oral solution sugar free (A A H Pharmaceuticals Ltd)	No
68289	Glimepiride 4mg tablets (Waymade Healthcare Plc)	No
68389	Metformin 500mg/5ml oral solution sugar free (Pinewood Healthcare)	No
68415	Gliclazide 30mg modified-release tablets (Phoenix Healthcare Distribution Ltd)	No
68636	Metformin 850mg/5ml oral solution sugar free	No
68675	Glimepiride 4mg tablets (Somex Pharma)	No
68819	Gliclazide 80mg tablets (Bristol Laboratories Ltd)	No

For peer review only

Supplemental table 3: Product codes for drugs used for diabetes and glitazone status.

Glitazones Case-Control				
	**N = 480**	**N = 1920**		
**Glitazone exposure status**				
**Per-year glitazone exposure**				
**Unexposed**	383 (79.8%)	1460 (76.0%)	Referent	-
**≤ 1 year**	41 (8.5%)	151 (7.9%)	1.11 (0.77, 1.61)	0.59
**1 < years ≤ 2**	23 (4.8%)	89 (4.6%)	1.06 (0.66, 1.71)	0.82
**2 < years ≤ 3**	29 (4.0%)	66 (3.4%)	1.13 (0.67, 1.92)	0.65
**3 < years ≤ 4**	8 (1.7%)	61 (3.2%)	0.52 (0.25, 1.10)	0.07
**4 < years ≤ 5**	2 (0.4%)	41 (2.1%)	0.18 (0.04, 0.77)	0.003
**5 < years ≤ 6**	2 (0.4%)	23 (1.2%)	0.30 (0.07, 1.30)	0.06
**> 6 years**	2 (0.4%)	29 (1.5%)	0.44 (0.10, 1.94)	0.23
**"First drug exposure" status**				
**Glitazone**	4 (0.8%)	13 (0.7%)	1.10 (0.35, 3.44)	0.87
**Other anti-type 2 diabetes drug**	476 (99.2%)	1907 (99.3%)	Referent	-

* Model adjusted for sex, age, IMD, retrospective prescription history (categorised yearly) and mean HbA1c levels. ** OR calculated for per year increase in variable.

Supplemental table 4: Case-control results for brain tumour cases with glitazone exposure in years duration groups and as first drug exposure.

For peer review only

Variable	Cases (%)	Controls (%)	Adjusted OR (95% CI) *	P-value
	**Imputed HbA1c levels**			
	**N = 480**	**N = 1920**		
**Glitazone exposure status**				
**Exposed**	97 (20.2%)	460 (24.0%)	0.81 (0.66, 1.00)	0.11
**Unexposed**	383 (79.8%)	1460 (76.0%)	Referent	-
**Mean glitazone exposure duration (longest), days (SD)**	607 (583)	872 (760)	0.87 (0.82, 0.93) **	0.001
**Mean glitazone exposure duration (total), days (SD)**	604 (720)	1002 (823)	0.85 (0.79, 0.91) **	0.00005
	**No HbA1c adjustment**			
	**N = 480**	**N = 1920**		
**Glitazone exposure status**				
**Exposed**	97 (20.2%)	460 (24.0%)	0.79 (0.62, 1.02)	0.07
**Unexposed**	383 (79.8%)	1460 (76.0%)	Referent	-
**Mean glitazone exposure duration (longest), days (SD)**	607 (583)	872 (760)	0.87 (0.80, 0.94)	0.0008
**Mean glitazone exposure duration (total), days (SD)**	604 (720)	1002 (823)	0.85 (0.78, 0.92)	0.00004

*** Adjusted for sex, age, IMD and retrospective prescription history. HbA1c adjustment differed according to the analysis.**
**** OR calculated for per year increase in variable.**

Supplemental table 5: Case-control results for brain tumour cases with glitazone exposure with HbA1C imputed or not adjusted in the multivariable models.

For peer review only

**Glitazones - 6 months latency period**				
	**N = 480**	**N = 1920**		
**Glitazone exposure status**	**Cases (%)**	**Controls (%)**	**Adjusted OR (95% CI) ***	**P-value**
**Exposed**	95 (19.8%)	460 (24.0%)	0.82 (0.64, 1.05)	0.12
**Unexposed**	385 (80.2%)	1460 (76.0%)	Referent	-
**Mean glitazone exposure duration (longest), days (SD)**	574 (581)	770 (651)	0.88 (0.81, 0.96) **	0.002
**Mean glitazone exposure duration (total), days (SD)**	771 (693)	988 (796)	0.89 (0.83, 0.96) **	0.0015
**Glitazones - 12 months latency period**				
	**N = 480**	**N = 1920**		
**Glitazone exposure status**				
**Exposed**	92 (19.2%)	457 (23.8%)	0.79 (0.61, 1.02)	0.07
**Unexposed**	388 (80.8%)	1463 (76.2%)	Referent	-
**Mean glitazone exposure duration (longest), days (SD)**	540 (572)	753 (642)	0.87 (0.79, 0.95) **	0.002
**Mean glitazone exposure duration (total), days (SD)**	670 (690)	958 (774)	0.87 (0.80, 0.94) **	0.0003
**Glitazones - 24 months latency period**				
	**N = 480**	**N = 1920**		
**Glitazone exposure status**				
**Exposed**	86 (17.9%)	449 (23.4%)	0.74 (0.57, 0.96)	0.02
**Unexposed**	394 (82.1%)	1471 (76.6%)	Referent	-
**Mean glitazone exposure duration (longest), days (SD)**	478 (557)	720 (625)	0.83 (0.75, 0.92) **	0.0004
**Mean glitazone exposure duration (total), days (SD)**	593 (672)	901 (730)	0.84 (0.76, 0.91) **	0.000007

Supplemental table 6: Case-control results for brain tumour cases with glitazone exposure for latency periods of 6, 12, and 24 months.

For peer review only

STROBE 2007 (v4) checklist of items to be included in reports of observational studies in epidemiology*
Checklist for cohort, case-control, and cross-sectional studies (combined)

Section/Topic	Item #	Recommendation	Reported on page #
**Title and abstract**	1	(*a*) Indicate the study's design with a commonly used term in the title or the abstract	1
		(*b*) Provide in the abstract an informative and balanced summary of what was done and what was found	2
**Introduction**			
Background/rationale	2	Explain the scientific background and rationale for the investigation being reported	5
Objectives	3	State specific objectives, including any pre-specified hypotheses	5
**Methods**			
Study design	4	Present key elements of study design early in the paper	6
Setting	5	Describe the setting, locations, and relevant dates, including periods of recruitment, exposure, follow-up, and data collection	6
Participants	6	(*a*) *Cohort study*—Give the eligibility criteria, and the sources and methods of selection of participants. Describe methods of follow-up<br>*Case-control study*—Give the eligibility criteria, and the sources and methods of case ascertainment and control selection. Give the rationale for the choice of cases and controls<br>*Cross-sectional study*—Give the eligibility criteria, and the sources and methods of selection of participants	6-7
		(*b*) *Cohort study*—For matched studies, give matching criteria and number of exposed and unexposed<br>*Case-control study*—For matched studies, give matching criteria and the number of controls per case	6-7
Variables	7	Clearly define all outcomes, exposures, predictors, potential confounders, and effect modifiers. Give diagnostic criteria, if applicable	6-8
Data sources/ measurement	8*	For each variable of interest, give sources of data and details of methods of assessment (measurement). Describe comparability of assessment methods if there is more than one group	6-8
Bias	9	Describe any efforts to address potential sources of bias	6-7
Study size	10	Explain how the study size was arrived at	6
Quantitative variables	11	Explain how quantitative variables were handled in the analyses. If applicable, describe which groupings were chosen and why	8
Statistical methods	12	(*a*) Describe all statistical methods, including those used to control for confounding	9-10
		(*b*) Describe any methods used to examine subgroups and interactions	9-10
		(*c*) Explain how missing data were addressed	9-10
		(*d*) *Cohort study*—If applicable, explain how loss to follow-up was addressed<br>*Case-control study*—If applicable, explain how matching of cases and controls was addressed	

For peer review only

		Cross-sectional study—If applicable, describe analytical methods taking account of sampling strategy	
		(*e*) Describe any sensitivity analyses	9-10
**Results**			
Participants	13*	(a) Report numbers of individuals at each stage of study—eg numbers potentially eligible, examined for eligibility, confirmed eligible, included in the study, completing follow-up, and analysed	10
		(b) Give reasons for non-participation at each stage	N/A
		(c) Consider use of a flow diagram	N/A
Descriptive data	14*	(a) Give characteristics of study participants (eg demographic, clinical, social) and information on exposures and potential confounders	10 & 18
		(b) Indicate number of participants with missing data for each variable of interest	11
		(c) *Cohort study*—Summarise follow-up time (eg, average and total amount)	
Outcome data	15*	*Cohort study*—Report numbers of outcome events or summary measures over time	
		Case-control study—Report numbers in each exposure category, or summary measures of exposure	18
		Cross-sectional study—Report numbers of outcome events or summary measures	
Main results	16	(*a*) Give unadjusted estimates and, if applicable, confounder-adjusted estimates and their precision (eg, 95% confidence interval). Make clear which confounders were adjusted for and why they were included	19-20
		(*b*) Report category boundaries when continuous variables were categorized	18-20
		(*c*) If relevant, consider translating estimates of relative risk into absolute risk for a meaningful time period	
Other analyses	17	Report other analyses done—eg analyses of subgroups and interactions, and sensitivity analyses	11-12
**Discussion**			
Key results	18	Summarise key results with reference to study objectives	12
Limitations	19	Discuss limitations of the study, taking into account sources of potential bias or imprecision. Discuss both direction and magnitude of any potential bias	13
Interpretation	20	Give a cautious overall interpretation of results considering objectives, limitations, multiplicity of analyses, results from similar studies, and other relevant evidence	14
Generalisability	21	Discuss the generalisability (external validity) of the study results	14
**Other information**			
Funding	22	Give the source of funding and the role of the funders for the present study and, if applicable, for the original study on which the present article is based	3-4

*Give information separately for cases and controls in case-control studies and, if applicable, for exposed and unexposed groups in cohort and cross-sectional studies.

Note: An Explanation and Elaboration article discusses each checklist item and gives methodological background and published examples of transparent reporting. The STROBE checklist is best used in conjunction with this article (freely available on the Web sites of PLoS Medicine at http://www.plosmedicine.org/, Annals of Internal Medicine at http://www.annals.org/, and Epidemiology at http://www.epidem.com/). Information on the STROBE Initiative is available at www.strobe-statement.org.

For peer review only

Use of drugs for hyperlipidaemia and diabetes and risk of primary and secondary brain tumours: nested case control studies using the UK Clinical Practice Research Datalink (CPRD).

Journal:	*BMJ Open*
Manuscript ID	bmjopen-2023-072026.R2
Article Type:	Original research
Date Submitted by the Author:	04-Dec-2023
Complete List of Authors:	Robinson, Jamie W; University of Bristol<br>Martin, Richard; University of Bristol Medical School, Population Health Sciences<br>Ozawa, Mio; University College London<br>Elwenspoek, Martha; NIHR CLAHRC West, CLAHRC West; University of Bristol Medical School, Populations Health Sciences<br>Redaniel, Maria Theresa; University of Bristol, NIHR CLAHRC West; University of Bristol, School of Social and Community Medicine<br>Kurian, Kathreena; University of Bristol Medical School, MRC Integrative Epidemiology Unit; University of Bristol, Brain Tumour Research Centre<br>Ben-Shlomo, Yoav; University of Bristol, Department of Population Health Sciences; University Hospitals Bristol NHS Foundation Trust
**Primary Subject Heading**:	Epidemiology
Secondary Subject Heading:	Oncology
Keywords:	Epidemiology < ONCOLOGY, Head & neck tumours < ONCOLOGY, CLINICAL PHARMACOLOGY, EPIDEMIOLOGIC STUDIES, Neurological oncology < ONCOLOGY

SCHOLARONE™
Manuscripts

I, the Submitting Author has the right to grant and does grant on behalf of all authors of the Work (as defined in the below author licence), an exclusive licence and/or a non-exclusive licence for contributions from authors who are: i) UK Crown employees; ii) where BMJ has agreed a CC-BY licence shall apply, and/or iii) in accordance with the terms applicable for US Federal Government officers or employees acting as part of their official duties; on a worldwide, perpetual, irrevocable, royalty-free basis to BMJ Publishing Group Ltd ("BMJ") its licensees and where the relevant Journal is co-owned by BMJ to the co-owners of the Journal, to publish the Work in this journal and any other BMJ products and to exploit all rights, as set out in our licence.

The Submitting Author accepts and understands that any supply made under these terms is made by BMJ to the Submitting Author unless you are acting as an employee on behalf of your employer or a postgraduate student of an affiliated institution which is paying any applicable article publishing charge ("APC") for Open Access articles. Where the Submitting Author wishes to make the Work available on an Open Access basis (and intends to pay the relevant APC), the terms of reuse of such Open Access shall be governed by a Creative Commons licence – details of these licences and which Creative Commons licence will apply to this Work are set out in our licence referred to above.

Other than as permitted in any relevant BMJ Author's Self Archiving Policies, I confirm this Work has not been accepted for publication elsewhere, is not being considered for publication elsewhere and does not duplicate material already published. I confirm all authors consent to publication of this Work and authorise the granting of this licence.

For peer review only

Use of drugs for hyperlipidaemia and diabetes and risk of primary and secondary brain tumours: nested case control studies using the UK Clinical Practice Research Datalink (CPRD).

Jamie W Robinson[1], Richard M Martin[1,2,3], Mio Ozawa[4], Martha M C Elwenspoek[2,5], Maria Theresa Redaniel[2,5], Kathreena M Kurian[1,6]*, Yoav Ben-Shlomo[2,5]*

1. MRC Integrative Epidemiology Unit, University of Bristol, Bristol, BS8 2BN, UK (JWR, RMM, KMK)
2. Department of Population Health Sciences, University of Bristol, Bristol, BS8 1UD, UK (RMM, MMCE, MTR, YBS)
3. National Institute for Health Research (NIHR) Bristol Biomedical Research Centre, University Hospitals Bristol and Weston NHS Foundation Trust and University of Bristol, Bristol, UK (RMM)
4. Population, Policy and Practice Research and Teaching Department, UCL Great Ormond Street Institute of Child Health, 30 Guilford Street, London, WC1N 1EH (MC)
5. The National Institute for Health Research Applied Research Collaboration West (NIHR ARC West), University Hospitals Bristol NHS Foundation Trust, BS1 2NT Bristol, UK (MMCE, MTR, YBS)
6. Brain Tumour Research Centre, University of Bristol, Bristol, BS10 5NB, UK (KMK)

* Joint senior authors.

Corresponding author: Yoav Ben-Shlomo (y.ben-shlomo@bristol.ac.uk) and Kathreena M Kurian (kathreena.kurian@bristol.ac.uk)

Short title: Drugs for hyperlipidaemia and diabetes and brain tumours

Word Count: Abstract: 255, Text: 3,860

Tables: 3

Supplemental tables: 6

For peer review only - http://bmjopen.bmj.com/site/about/guidelines.xhtml

Abstract

Objectives: Previous studies have suggested that fibrates and glitazones may have a role in brain tumour prevention. We examined if there is support for these observations using primary care records from the UK Clinical Practice Research Datalink (CPRD).

Design: We conducted two nested case-control studies using primary and secondary brain tumours identified within CPRD between 2000 to 2016. We selected cases and controls amongst the population of individuals who had been treated with any anti-diabetic or anti-hyperlipidaemic medication to reduce confounding by indication.

Setting: Adults older than 18 years registered with a General Practitioner in the UK contributing data to CPRD.

Results: We identified 7,496 people with any brain tumour (4,471 primary; 3,025 secondary) in total. After restricting cases and controls to those prescribed any anti-diabetic or anti-hyperlipidaemic medication, there were 1,950 cases and 7,791 controls in the fibrate and 480 cases with 1,920 controls in the glitazone analyses. Longer use of glitazones compared to all other anti-diabetic medications was associated with a reduced risk of primary (adjusted odds ratio [aOR] = 0.89 per year, 95% CI: 0.80, 0.98), secondary (aOR = 0.87 per year, 95% CI: 0.77, 0.99) or combined brain tumours (aOR = 0.88 per year, 95% CI: 0.81, 0.95). There was little evidence that fibrate exposure was associated with risk of either primary or secondary brain tumours.

Conclusions: Longer exposure to glitazones was associated with reduced primary and secondary brain tumour risk. Further basic science and population-based research should explore this finding in greater detail, in terms of replication and mechanistic studies.

Key Words: Brain cancer, glioma, secondary brain tumours, metastasis, fibrates, glitazones, observational epidemiology.

For peer review only

Strengths and limitations of this study

- Large case control studies using database free of selection bias and generalizable to other high income settings.
- Exposure well measured from medical systems avoiding recall bias
- Design accounted for confounding by indication and time-window bias
- Adjustment for individual socioeconomic status used an ecological proxy and residual confounding possible.
- All secondary brain cancers were combined to maintain statistical power

For peer review only

For peer review only - http://bmjopen.bmj.com/site/about/guidelines.xhtml

Introduction

People diagnosed with primary or secondary (breast, lung, skin melanoma, colorectal and kidney) brain tumours typically have less than a 20% five-year survival rate and brain tumours are responsible for the most years of life lost of any cancer [1]. The development of novel cancer drugs is beset by substantial costs and high failure rates. Re-purposing existing approved drugs for different diseases than originally designed potentially saves the considerable time and costs involved in conducting new clinical trials.

Nuclear hormone receptors (NHR) have crucial roles in cellular homeostasis and have been implicated in the development of cancer. Peroxisome proliferator-activated receptors (PPARs) are a family of ligand-activated transcription factors that regulate the expression of genes and, as a subtype of NHRs, are involved in the control of proliferation and differentiation of cells[2-4]. There are three isoforms of PPARs which are designated as -α, -β/δ and -γ. PPARs have been identified as potential candidates in cancer treatment, with some animal models and early-stage human trials showing beneficial outcomes, including arresting development of precursor cancer cells, decreased serum cancer antigens and tolerance by patients[5-8]. The genes regulated by these transcription factors are also involved in the transport, metabolism and storage of fatty acids, in inflammatory processes and in the onset of type 2 diabetes mellitus[3, 9, 10].

PPAR-α and -γ are clinically significant due to the use of agonistic compounds such as fibrates in hyperlipidaemia and thiazolidinediones (glitazones) in diabetes respectively. Both glitazones [11] and fibrates such as fenofibrate cross the blood-brain barrier, albeit at a slower rate[12]. PPARs are expressed in astrocytes, oligodendrocytes and microglia, and may play a role in growth and differentiation of those cells, including brain tumour stem cells[13]. These drugs, tumours, given their safety profile and cost, have the potential for being repurposed for the prevention of brain cancers and may also reduce the risk of secondary tumours by inhibiting angiogenesis.

We hypothesized that PPAR-α and -γ agonists (fibrates and glitazones), would be associated with a reduced risk of developing brain tumours compared to other treatments for hyperlipidaemia and diabetes using the UK Clinical Practice Research Datalink (CPRD), a very large population-based primary care database.

Materials and methods

For peer review only - http://bmjopen.bmj.com/site/about/guidelines.xhtml

We used the CPRD database which has data from a network of over 2,000 general practitioners (GPs) from more than 670 practices across the UK. Validated clinical information is held on over 16 million registered patients, amounting to circa 7% of the UK population [14] and broadly representative of that population [15, 16]. Types of data held within the CPRD include, but are not limited to, patient demographics, referrals, hospital admissions, clinical diagnoses and drug prescriptions. Data from secondary care or hospital visits are routinely fed back into the electronic records' system via staff at GP practices. CPRD is jointly sponsored by the Medicines and Healthcare products Regulatory Agency (MHRA) and the National Institute of Health Research (NIHR). Data were extracted from CPRD GOLD and linked to ONS death registration data and census data on area deprivation (see below).

Study design and eligible Participants

We undertook two nested case control studies from within the CPRD database as the eligibility criteria (see below) for the fibrate and glitazone analyses were different. All participants in the study had to be 18 years or older and registered within the CPRD between 1st January 2000 to 31st December 2016, the former being the first year that glitazones were licensed within the UK. We specified that eligible participants had to be treated with either (i) an anti-hyperlipidaemic or (ii) anti-diabetic medication to reduce the likelihood of "confounding by indication", a type of confounding which can distort the association between drug exposure and outcome when an indication to prescribe a drug (e.g. diabetes) is also associated with the outcome. All brain tumour cases and controls that were not treated with either these classes of drugs were therefore not included in the sample selection and analyses. The sample size was pragmatic based on all available cases from the dataset rather than based an "a priori" sample size calculation to maximise power.

For peer review only

Cases

Incident brain tumour (primary or secondary) cases were identified using a large list of Read codes (**Supplemental table 1**) and were classified as either of primary or secondary cancers using histopathology [17]. For secondary tumours, we did not have information on the site of primary tumour, but we retrospectively searched for another prior cancer diagnosis to estimate the site of the primary cancer. However, if this had been recorded prior to 1st Jan 2000, or recorded at a non-CPRD participating GP, it may have been missing from our file and thus not captured by our retrospective search.

For peer review only - http://bmjopen.bmj.com/site/about/guidelines.xhtml

Controls

We sampled controls using “incidence density sampling”, whereby on the date each case was diagnosed we identified the potential pool of controls who did not have a prior diagnosis of a brain tumour. Up to four controls were randomly selected per case (to maximise power) and were age-frequency matched to cases by 10-year age groups (<20, 20-29, 30-39, 40-49, 50-59, 60-69, 70-79 and ≥80 years) and male/female sex to ensure reasonable balance of these variables across case-control strata.

Both “immortal time” and “time-window” biases are potential hazards in pharmaco-epidemiology studies leading to misleading results [18], though the former is not relevant to our case-control study design [19]. Time-window bias occurs in case-control studies when the method of selecting controls results in an artefactually longer time window for potential exposure to be identified than their matched cases resulting in a spurious inverse (protective) association between the exposure and outcome [18]. To prevent time-window bias, we specified that controls must have the same retrospective duration of potential exposure (any drug treatment for either hyperlipidaemia or diabetes) within CPRD as the matched case, based on the case index date plus or minus six months. For example, if a case had 6.3 years of any drug medication exposure prior to their diagnosis date, then only controls who also had between 5.8 and 6.8 years of any drug exposure over the same secular time period were sampled and hence had the same potential for exposure to either a fibrate or glitazone as cases.

For peer review only

Exposures

All participants were treated with some medication for either type 2 diabetes or hyperlipidaemia. If they received a fibrate or glitazone they were classified as “exposed” whilst all the other drugs used for the management of these diseases were classified as the unexposed comparator. Product codes used to determine exposure status are given in **Supplemental table 2 and Supplemental table 3**. We excluded participants with type 1 diabetes, defined as those participants treated solely with insulin therapy, as these patients are very different to those with the more common type 2 diabetes in terms of causes and duration of dysregulated glycaemic control. Participants who started on oral anti-diabetic medication, but at some point in time also received insulin therapy, were eligible for inclusion. Participants on combination therapy were not excluded (for example, both glitazone plus another anti-diabetic medication); these participants were considered as exposed, if one of the therapies was the drug of interest. Similarly, some participants were exposed to both fibrates

For peer review only - http://bmjopen.bmj.com/site/about/guidelines.xhtml

and glitazones and were considered exposed for both studies (however we tested both these assumption in sensitivity analyses; see below).

Exposure was classified in three ways: (i) "Exposed binary" (Yes/No) indicating that a subject has been ever exposed to a prescription for fibrates or glitazones; (ii) "Longest exposure duration" which was the total uninterrupted time (no breaks of 90 days or more between prescriptions) each participant had been prescribed either fibrates or glitazones. If there was more than one such period, then we took the longest duration; (iii) "Total exposure duration", which was the sum of the total prescription duration (with or without interruptions). Exposure duration for both the longest and total exposure duration were categorised as follows: unexposed, ≤ 1 year, >1 and ≤ 2 years, >2 and ≤ 3 years, >3 and ≤ 4 years , >4 and ≤ 5 years , >5 and ≤ 6 years , >6 and ≤ 7 years, >7 years. Due to small numbers of participants reaching longer exposure durations, any duration exposure periods over 6 years were considered in the longest duration category in the glitazone analysis, and over 7 years as the longest duration category in the fibrate analysis.

Potential Confounders

The following variables were considered as potential confounders that might influence risk of developing a brain tumour and the choice of drug that a doctor might prescribe: age group, sex and socioeconomic status (SES). We used the Index of Multiple Deprivation 2015 (IMD) [20], as an ecological proxy for individual SES. This is a commonly used measure in the UK that uses census data on a wide variety of economic and health factors to derive a postcode-based deprivation ranked score so that that a higher score indicates less deprivation. IMD scores were grouped into five equal sized groups from most (quintile 1) to least deprived areas (quintile 5), so we could adjust for any non-linear associations.

In the glitazone analyses, we also tried to adjust for the severity of diabetes as a confounder, as severity may determine choice of anti-diabetic medications and possible cancer risk. We used glycosylated haemoglobin (HbA1c) levels as a measure of glycaemic control and hence severity of diabetes, where available. We converted and standardised the measures to the International Federation of Clinical Chemistry (IFCC) units (mmols/mol). The mean value was calculated using all available HbA1c readings for a subject. A three-level ordinal variable was created: 1, well controlled diabetes (≤ 58 mmols/mol); 2, sub-optimally controlled diabetes (> 58 mmols/mol and ≤ 75 mmols/mol); 3, poorly controlled diabetes (> 75 mmols/mol). If there were no measures of the participant's HbA1c levels, we assigned them to the well-controlled group based on the assumption that the clinician did not feel it

For peer review only - http://bmjopen.bmj.com/site/about/guidelines.xhtml

necessary to monitor the patient's glycaemic control (however, see below for sensitivity analyses that examine this assumption). We attempted to restrict HbA1c readings to only pre-baseline measures; however due to missing data, we had to occasionally include post-baseline HbA1c readings as a proxy measure.

Statistical Methods

Both case-control analyses were conducted using logistic regression to compute odds ratios (OR) and 95% confidence intervals (CI) of exposure to fibrate and glitazone drugs versus other drugs used for the same disease. Unadjusted and multivariable models were run, adjusting for the previously mentioned potential confounders with cases stratified by primary and secondary brain tumours and then combined as all brain tumours if there was no evidence of heterogeneity of effect to enhance statistical power. In addition, we adjusted for total potential exposure duration, defined as the time from either diagnosis or control index date and start of any medication first prescribed for either hyperlipidaemia or diabetes. This was one of the matching variables used to avoid time window bias. Exposure duration was analysed both as a continuous ordinal variable and also as a "dummy" variable to check for any evidence of non-linearity in the pattern of the odds ratios. We used logistic regression rather than conditional logistic models, as the latter may introduce collider bias and instead we used the matching variables as covariates in the unmatched logistic model as the recommended procedure (see reference [21] for detailed justification).

Sensitivity Analyses

The following sensitivity analyses were conducted to check the robustness of our main analyses: (i) Potential selection bias: we reclassified exposure only if the participant's first medication was a fibrate or glitazone. Someone whose first treatment was another drug but was later switched to a glitazone or fibrate in this analysis was classified as unexposed; (ii) Dual exposure: to evaluate if one drug had an effect on risk while the other did not, participants were dropped from the analysis if they were exposed to both a fibrate and a glitazone at the same time; (iii) Latency period: analyses were repeated excluding any exposure for short latencies defined as six, 12 and 24 months prior to the index date; and (iv) Missing HbA1c levels: we used multiple imputation (55 datasets generated, combined using Rubin's rules) to predict missing HbA1c levels based on case control status, age, IMD, sex, retrospective prescription history, ever exposure to a glitazone, total glitazone exposure and number of consultations, defined as each day the patient had at least one in-person consultation. The MICE algorithm in R [22] was used to impute missing HbA1c levels. We

For peer review only - http://bmjopen.bmj.com/site/about/guidelines.xhtml

also examined whether HbA1c predicted case control status both in the complete case as well as the imputation dataset. This is because if there was no evidence of an association with HbA1c then it cannot be a confounder and did not need to be included in the multivariable model, avoiding the issue of missing values. Sensitivity analyses were conducted using all brain tumour cases as there was limited statistical power in the separate primary and secondary cancer analyses.

Patient and Public Involvement

Patients or the public were not involved in the design, or conduct, or reporting, or dissemination plans of our research

Results

For the fibrate analyses, there were a total of 1,950 cases, of which 1,094 were primary and 856 were secondary brain tumours and 7,791 matched controls. For the glitazone analyses there were a total of 480 cases, of which 278 were primary and 202 were secondary brain tumours and 1,920 controls. There was little evidence of any important differences in age group, sex and IMD distribution between cases versus controls in both the fibrate and glitazone datasets (**Table 1**). Cases were more likely to have well-controlled HbA1c but this was because they were more likely to have missing HbA1c values which by default were classified as well-controlled diabetes.

For peer review only

Table 1: Distribution of possible confounders by case control status for both fibrate and glitazone datasets.

Fibrates Case-Control				
**Variable**	Cases (%)	Controls (%)	Crude OR (95% CI)	P
	**N = 1950**	**N = 7791**		
**Male**	1118 (57.3%)	4469 (57.4%)	Referent	-
**Female**	832 (42.7%)	3322 (42.6%)	1.00 (0.91, 1.11)	0.98
**Age, years**				
**< 20**	0 (0.0%)	0 (0.0%)	-	-
**20 – 29**	0 (0.0%)	0 (0.0%)	-	-
**30 – 39**	2 (0.1%)	5 (0.1%)	1.58 (0.31, 8.17)	0.58
**40 – 49**	6 (0.3%)	29 (0.4%)	0.82 (0.34, 1.98)	0.66
**50 – 59**	40 (2.1%)	193 (2.5%)	0.82 (0.58, 1.16)	0.26
**60 – 69**	220 (11.3%)	848 (10.9%)	1.03 (0.87, 1.21)	0.75
**70 – 79**	561 (28.8%)	2279 (29.3%)	0.97 (0.87, 1.09)	0.65
**≥ 80**	1121 (57.5%)	4437 (57.0%)	Referent	-
**IMD, quintile**				
**1, lower SES**	457 (23.4%)	1768 (22.7%)	1.05 (0.91, 1.22)	0.48
**2**	439 (22.5%)	1790 (23.0%)	Referent	-
**3**	430 (22.1%)	1594 (20.5%)	1.10 (0.95, 1.28)	0.21
**4**	331 (17.0%)	1466 (18.8%)	0.92 (0.79, 1.08)	0.31
**5, higher SES**	293 (15.0%)	1173 (15.1%)	1.02 (0.86, 1.20)	0.83
**Glitazones Case-Control**				
	**N = 480**	**N = 1920**		
**Male**	277 (57.7%)	1108 (57.7%)	Referent	-
**Female**	203 (42.3%)	812 (42.3%)	1.00 (0.82, 1.22)	1.00
**Age, years**				
**< 20**	1 (0.2%)	1 (0.1%)	3.93 (0.24, 63.00)	0.33
**20 – 29**	1 (0.2%)	7 (0.4%)	0.56 (0.07, 4.58)	0.59
**30 – 39**	3 (0.6%)	11 (0.6%)	1.07 (0.30, 3.87)	0.92
**40 – 49**	7 (1.5%)	28 (1.5%)	0.98 (0.42, 2.27)	0.97
**50 – 59**	15 (3.1%)	76 (4.0%)	0.78 (0.44, 1.37)	0.38
**60 – 69**	56 (11.7%)	233 (12.1%)	0.94 (0.68, 1.30)	0.73
**70 – 79**	135 (28.1%)	535 (27.9%)	0.99 (0.79, 1.25)	0.94
**≥ 80**	262 (54.6%)	1029 (52.6%)	Referent	-
**IMD, quintile**				
**1, lower SES**	90 (18.8%)	362 (18.9%)	1.33 (0.95, 1.86)	0.09
**2**	114 (23.8%)	376 (19.6%)	1.62 (1.18, 2.24)	0.003
**3**	109 (22.7%)	384 (20.0%)	1.52 (1.10, 2.10)	0.01
**4**	78 (16.3%)	418 (21.8%)	Referent	-
**5, higher SES**	89 (18.5%)	380 (19.8%)	1.26 (0.90, 1.75)	0.18
**Mean prescription history, years (SD)**	2.12 (2.41)	1.99 (2.27)	1.02 (0.98, 1.07) *	0.27
**HbA1c ***				
**1, well controlled**	418 (87.0%)	1490 (77.6%)	Referent	-
**2**	42 (9.0%)	305 (15.9%)	0.49 (0.35, 0.69)	0.001
**3, poorly controlled**	20 (4.0%)	125 (6.5%)	0.57 (0.35, 0.93)	0.02
**Missing (coded as 1)**	338 (70.4%)	979 (51.0%)	-	-

* HbA1C = glycosylated haemoglobin a measure of glycaemic control used to monitor patients with diabetes.

For peer review only - http://bmjopen.bmj.com/site/about/guidelines.xhtml

There was little evidence that any exposure to fibrates was associated with brain tumour risk for either primary (adjusted OR [aOR] 0.99, 95% CI: 0.75, 1.30; P = 0.94), secondary (aOR 0.91, 95% CI: 0.67, 1.23; P = 0.55) or all tumours (aOR 0.98, 95% CI: 0.78, 1.24; P = 0.88) (**Table 2**). We found little evidence for a dose-response relationship of fibrate exposure, either as longest or total duration, with brain tumour risk for primary, secondary or all tumours (aORs varied from 0.95 to 1.01 and all consistent with chance). The sensitivity analysis, which only classified exposed if this was the first prescribed anti-hyperlipidaemia medication, found similar results but with less precision (aOR = 0.92, 95% CI: 0.69, 1.22; P = 0.54)

There was very weak evidence of an inverse association between ever exposure to glitazones (aOR = 0.80, 95% CI: 0.65, 1.07; P = 0.14) and all tumours, which was stronger with secondary tumours (aOR = 0.68, 95% CI: 0.46, 1.02; P = 0.06) rather than primary tumours (aOR = 0.96, 95% CI 0.69, 1.33; P = 0.81) (**Table 3**), though this could have been due to chance. The inverse association was stronger and consistent across both primary and secondary tumours when the exposure was classified as longest duration; primary (aOR per year 0.89, 95% CI: 0.80, 0.98; P = 0.02), secondary (aOR per year 0.87, 95% CI: 0.77, 0.99; P = 0.02) and all brain tumours (aOR per year 0.88, 95% CI: 0.81, 0.95; P = 0.002). Very similar results were seen with total duration of exposure (e.g. aOR per year for all tumours 0.85, 95% CI: 0.79, 0.92; P = 8.00×10^{-5}).

For peer review only - http://bmjopen.bmj.com/site/about/guidelines.xhtml

Table 2: Case-control results for primary, secondary and all brain tumour cases with fibrate exposure.

Fibrates												
	Primary brain tumours				Secondary brain tumours				All brain tumours			
**Variable**	**Cases (%)**	**Controls (%)**	**Adjusted OR (95% CI) ***	**P**	**Cases (%)**	**Controls (%)**	**Adjusted OR (95% CI) ***	**P**	**Cases (%)**	**Controls (%)**	**Adjusted OR (95% CI) ***	**P**
	N = 1094	N = 4375			N = 856	N = 3416			N = 1950	N = 7791		
**Fibrate exposure status**												
**Exposed**	68 (6.2%)	278 (6.4%)	0.99 (0.75, 1.30)	0.94	56 (6.5%)	245 (7.2%)	0.91 (0.67, 1.23)	0.55	124 (6.4%)	523 (6.7%)	0.98 (0.78, 1.24)	0.88
**Unexposed**	1026 (93.8%)	4097 (93.7%)	Referent	-	800 (93.5%)	3171 (92.8%)	Referent	-	1826 (93.6%)	7268 (93.3%)	Referent	-
**Fibrate longest duration, days (SD) ****	799 (1050)	755 (913)	1.01 (0.94, 1.09)	0.76	676 (913)	878 (1123)	0.95 (0.87, 1.03)	0.16	743 (989)	813 (1018)	0.97 (0.91, 1.03)	0.35
**Fibrate total duration, days (SD) ****	969 (1183)	1073 (1233)	0.99 (0.93, 1.05)	0.64	871 (1232)	1217 (1408)	0.95 (0.88, 1.01)	0.08	925 (1202)	1141 (1319)	0.97 (0.92, 1.01)	0.11

* Model adjusted for sex, age, IMD, retrospective prescription history (categorised yearly). ** OR calculated for per year increase in variable.

For peer review only - http://bmjopen.bmj.com/site/about/guidelines.xhtml

Table 3: Case-control results for primary, secondary and all brain tumour cases with glitazone exposure.

	Primary brain tumours				Secondary brain tumours				All brain tumours			
**Variable**	**Cases (%)**	**Controls (%)**	**Adjusted OR (95% CI) ***	**P**	**Cases (%)**	**Controls (%)**	**Adjusted OR (95% CI) ***	**P**	**Cases (%)**	**Controls (%)**	**Adjusted OR (95% CI) ***	**P**
	N = 278	N = 1112			N = 202	N = 808			N = 480	N = 1920		
**Glitazone exposure status**												
**Exposed**	61 (21.9%)	261 (23.5%)	0.96 (0.69, 1.33)	0.81	36 (17.8%)	199 (24.6%)	0.68 (0.46, 1.02)	0.06	97 (20.2%)	460 (24.0%)	0.80 (0.65, 1.07)	0.14
**Unexposed**	217 (78.1%)	851 (76.5%)	Referent	-	166 (82.2%)	609 (75.4%)	Referent	-	383 (79.8%)	1460 (76.0%)	Referent	-
**Glitazone longest duration, days (SD) ****	551 (599)	851 (754)	0.89 (0.80, 0.98)	0.02	701 (550)	899 (767)	0.87 (0.77, 0.99)	0.02	607 (583)	872 (760)	0.88 (0.81, 0.95)	0.002
**Glitazone total duration, days (SD) ****	684 (695)	1000 (809)	0.90 (0.82, 0.99)	0.02	845 (681)	1073 (842)	0.88 (0.79, 0.98)	0.01	603 (720)	1002 (823)	0.86 (0.79, 0.92)	0.00008

* Model adjusted for sex, age, IMD, retrospective prescription history (categorised yearly) and mean HbA1c levels. ** OR calculated for per year increase in variable.

For peer review only - http://bmjopen.bmj.com/site/about/guidelines.xhtml

Sensitivity analyses

Results from the analysis of duration period as a categorical, instead of a continuous variable, found that the inverse association with glitazone and all tumours only emerged after 3 years of exposure; however, the confidence intervals for the shorter duration periods were sufficiently wide as to not exclude any dose-response effect across time (see **Supplemental table 4**). When we only classified exposed if this was the first prescribed anti-diabetes medication, we found little evidence of an association but with very wide confidence intervals due to fewer exposed cases and controls (aOR = 1.10, 95% CI: 0.35, 3.44; P = 0.87) (see **Supplemental table 4**). Effects were similar (longest duration aOR = 0.85, 95% CI 0.78, 0.93; P = 4.00×10^{-4}) when investigating the effects of monotherapy

Investigating Missing HbA1c levels

HbA1c results were missing for 54.8% of subjects in the glitazone analysis. We observed similar results for the association of both glitazone exposure status (aOR = 0.81, 95% CI: 0.66, 1.00; P = 0.11) and longest duration of glitazone exposure (aOR per year = 0.87, 95% CI: 0.82, 0.93; P = 0.001) with all brain tumour risk when multiple imputation was used for the missing HbA1c values (**Supplemental table 5**). There was little evidence of an association between poorly versus well-controlled HbA1c and case-control status in both the imputed (aOR = 0.95, 95% CI: 0.69, 1.32; P = 0.80) and complete case analysis (aOR = 1.02, 95% CI: 0.60, 1.73; P = 0.80), suggesting that HbA1C may not be a confounder as it did not predict case control status and therefore adjustment may not be necessary. This observation was supported by the aOR without adjustment for HbA1c which was almost identical to the imputed results so that the odds ratio per year of glitazaone exposure with longest duration was 0.87 (95% CI: 0.80, 0.94; P = 0.0008) (**Supplemental table 5**).

Latency Period

Similar associations of both fibrates and glitazones with all brain tumour risk were observed in the sensitivity analyses with different latency periods. In the glitazone analysis, the inverse association seen in the initial analyses for longest duration was very similar as the latency period increased (aOR after a 6-, 12- and 24-month latency were 0.88, 0.87, 0.83 respectively) and unlikely to be due to chance (**Supplemental table 6**).

Discussion

To the best of our knowledge, this is the first pharmacoepidemiological study of the effects of fibrate and glitazone prescription on risk of brain tumours compared to other treatments for

For peer review only - http://bmjopen.bmj.com/site/about/guidelines.xhtml

hyperlipidaemia and type 2 diabetes. There have been various *in vivo* studies that have suggested fibrate exposure may be protective for brain tumours, specifically gliomas, by modulating PPAR-α inhibition [23-26]. There is similar evidence available for the consideration of glitazones as a treatment option from *in vivo* studies [27-30]. However, prior to this study, there have been no other pharmacoepidemiological studies that examine whether fibrates or glitazones affect brain tumour risk.

In the glitazone case-control analysis, there was a modest inverse effect observed for ever exposure to glitazones, which was seen more strongly in the dose-response analyses, especially after exposure durations of 4 or more years, in both univariable and multivariable models. Repeating our analyses and accounting for various latency periods did not alter our conclusions. Similar inverse associations were seen for both primary and secondary brain tumours, but limited data meant analyses could not test whether there were differences by type of primary cancer for those subjects with secondary brain tumours.

We found little evidence that exposure to fibrates was associated with all, primary or secondary brain tumour risk, in contrast to some previous literature[31]. Sensitivity analyses, investigating a potential dose-response effect, or exposure status based on whether fibrates were the first prescribed drug for treatment, also showed little evidence of an association between fibrate exposure and all brain tumour risk.

The assumption that missing HbA1c levels should be assumed to reflect good glycaemic control was tested using follow-up sensitivity analyses to ensure this did not bias the potential negative association between glitazone exposure and brain tumour risk. However, in both the multiple imputation and complete case analysis we found no association between HbA1c and case control status. Furthermore, the results using both multiple imputation or no adjustment were consistent with the primary analyses.

This study has several strengths and limitations. We used a nested case control design within CPRD, a large and well-established database which has been validated by numerous sources [15, 16, 32]. It should be free of selection bias as almost all UK residents are registered with a GP and the population captured by CPRD-registered practices is representative of the general population [14]. Exposure was well measured as it was recorded from medical systems that used the record to print out prescribed drugs for redemption at a pharmacist. The exposure data were collected prospectively, prior to diagnosis, which will avoid recall bias. However, we have only measured receipt of a prescription and have no data on actual drug intake or

For peer review only - http://bmjopen.bmj.com/site/about/guidelines.xhtml

therapeutic levels. Despite this, as both the response to therapy are monitored the dosage is likely to be adjusted in response to good or bad control of the subjects' lipids and blood sugar levels. Exposure was classified from first prescription of any drug for lipids or diabetes and controls were sampled to have similar total prescription duration to avoid time-window bias, which was also adjusted in the multivariable models. The risk of confounding by indication was minimized by only sampling cases and controls who could have potentially been exposed to fibrates or glitazones because of a clinical indication. However, the consequence of this sampling strategy is that the number of available cases was markedly reduced with reduced statistical power to detect modest effects. Survivor bias is unlikely as all cases were included from diagnosis regardless of subsequent life expectancy and there is no evidence that the exposures themselves effect mortality risk. Not all potential risk factors for brain tumours were captured by CPRD (e.g. previous irradiation) but if these are not associated with exposure this will not cofound the results. Adjustment for SES was at an ecological rather than individual level but if anything is likely to bias the associations to the null due to non-differential measurement error. We had to combine all secondary brain cancers regardless of the primary cancer due to limited sample size so that there would have been no power to test the hypothesis for specific types of secondary brain tumours. Many of the secondary brain tumour cases had a previous diagnosis of common cancers which metastasise to brain, in particular breast, lung and skin cancers.

Overall longer glitazone exposure was associated with a reduced risk of brain metastasises from these primary sites but larger studies should be conducted to determine whether the association of glitazone exposure and secondary brain tumours is similar or not across these different primary sites. Risk of brain tumours may be greater in populations with either type 2 diabetes or hyperinsulinaemia [33, 34] though further studies are required to confirm these associations. It is therefore possible that glitazone may reduce risk of both primary and/or secondary brain tumours by improved glycaemic control. In addition, mice injected with Lewis Lung cancer which had been resected were less likely to develop metastatic disease after being treated with rosiglitazone compared to placebo possibly by its antiangiogenic effect. [35]. We cannot say if we would have observed the same associations if glitazones were prescribed as a preventative therapy in a high-risk non-diabetic population as the generalizability of our results are limited to those brain tumour cases with type 2 diabetes.

For peer review only - http://bmjopen.bmj.com/site/about/guidelines.xhtml

Conclusions

Longer exposure to glitazones was inversely associated with diagnosis of primary and secondary brain tumours, but we found little evidence of an association between fibrates and any type of brain tumour. Our results should be generalizable to other high income settings but may not be generalizable to low middle income populations. Further research is required to investigate whether these findings are replicated using independent datasets, preferably larger in size and/or with better data on glycaemic control and other potential confounders. If the glitazone association is biologically causal, this may lead to a better understanding of pathophysiological mechanisms and potential therapies for the prevention of brain cancers. For example, patients with specific aggressive cancers that have a high probability of brain metastases, could be treated with glitazones as a tertiary prevention strategy to prevent or delay secondary spread to the brain. Such a hypothesis could be tested in a future double blind clinical trial if stronger evidence emerges from other studies, given the safety and current use of glitazones for the management of diabetes.

Ethics Approval

The study was approved by the Independent Scientific Advisory Committee (ISAC) for the UK Medicines and Healthcare Products Regulatory Agency (ISAC protocol number: 18_149R). Participant consent is not required for ISAC approved CPRD studies using purely observational data. We assert that all procedures contributing to this work comply with the ethical standards of the relevant national and institutional committees on human experimentation and with the Helsinki Declaration of 1975, as revised in 2008.

Availability of Data and Materials

The Clinical Practice Research Datalink (CPRD) is an electronic healthcare record database open to all researchers. Researchers can apply to access CPRD data, and, if successful, can access the data of their choosing. The CPRD charges researchers and other organisations to access this data. The data that support the findings of this study are available from the Clinical Practice Research Datalink (CPRD) but restrictions apply to the availability of these data, which were used under license for the current study, and so are not publicly available.

Competing Interests

JWR receives funding from Biogen for unrelated research. All other authors declare no conflicts of interest.

For peer review only - http://bmjopen.bmj.com/site/about/guidelines.xhtml

Funding

The Medical Research Council (MRC)/University of Bristol Integrative Epidemiology Unit (IEU) is supported by the MRC and the University of Bristol (MC_UU_12013/1, MC_UU_12013/2). JWR is supported by a joint studentship from NHS Southmead Hospital Charity (SOCS/SJ1447). RMM is supported by a Cancer Research UK Programme Grant, the Integrative Cancer Epidemiology Programme (C18281/A29019). JWR and RMM are members of the MRC IEU which is supported by the Medical Research Council and the University of Bristol (MC_UU_12013/1-9). RMM is supported by the National Institute for Health Research (NIHR) Bristol Biomedical Research Centre which is funded by the National Institute for Health Research and is a partnership between University Hospitals Bristol NHS Trust and Weston NHS Foundation Trust and the University of Bristol. RMM is a National Institute for Health Research Senior Investigator (NIHR202411). The views expressed in this publication are those of the author(s) and not necessarily those of the NIHR or the UK Department of Health and Social Care. This work was supported by funding from the Brain Tumour Bank and Research Fund, Southmead Hospital Charity (Registered Charity Number: 1055900), and University of Bristol Campaigns and Alumni funding. This work was supported by CRUK [grant number C18281/A29019], as part of the Integrative Cancer Epidemiology Programme. MTR, MMCE, and YBS are supported by the NIHR Applied Research Collaboration West (NIHR ARC West). The views expressed in this article are those of the author(s) and not necessarily those of the NIHR or the Department of Health and Social Care.

Author's Contributions

Study hypothesis and design: KMK, RMM, YBS. Extraction of CPRD data: MMCE, MTR. Conceptualisation of analysis plan: RMM, YBS, KMK, MC, MTR Performed data management and statistical analyses: JWR, MC. Writing of the initial versions of the manuscript: JWR followed by YBS. All authors read, reviewed and provided feedback for final manuscript.

For peer review only - http://bmjopen.bmj.com/site/about/guidelines.xhtml

For peer review only

References

1. **Brain, other CNS and intracranial tumours statistics** [https://www.cancerresearchuk.org/health-professional/cancer-statistics/statistics-by-cancer-type/brain-other-cns-and-intracranial-tumours]

2. Kitamura S, Miyazaki Y, Shinomura Y, Kondo S, Kanayama S, Matsuzawa Y: **Peroxisome proliferator-activated receptor gamma induces growth arrest and differentiation markers of human colon cancer cells**. *Jpn J Cancer Res* 1999, **90**(1):75-80.

3. Poulsen L, Siersbaek M, Mandrup S: **PPARs: fatty acid sensors controlling metabolism**. *Semin Cell Dev Biol* 2012, **23**(6):631-639.

4. Rosen ED, Spiegelman BM: **PPARgamma : a nuclear regulator of metabolism, differentiation, and cell growth**. *J Biol Chem* 2001, **276**(41):37731-37734.

5. Barak Y, Liao D, He W, Ong ES, Nelson MC, Olefsky JM, Boland R, Evans RM: **Effects of peroxisome proliferator-activated receptor delta on placentation, adiposity, and colorectal cancer**. *Proc Natl Acad Sci U S A* 2002, **99**(1):303-308.

6. Demetri GD, Fletcher CD, Mueller E, Sarraf P, Naujoks R, Campbell N, Spiegelman BM, Singer S: **Induction of solid tumor differentiation by the peroxisome proliferator-activated receptor-gamma ligand troglitazone in patients with liposarcoma**. *Proc Natl Acad Sci U S A* 1999, **96**(7):3951-3956.

7. Mueller E, Smith M, Sarraf P, Kroll T, Aiyer A, Kaufman DS, Oh W, Demetri G, Figg WD, Zhou XP *et al*: **Effects of ligand activation of peroxisome proliferator-activated receptor gamma in human prostate cancer**. *Proc Natl Acad Sci U S A* 2000, **97**(20):10990-10995.

8. Smallridge RC, Copland JA, Brose MS, Wadsworth JT, Houvras Y, Menefee ME, Bible KC, Shah MH, Gramza AW, Klopper JP *et al*: **Efatutazone, an oral PPAR-gamma agonist, in combination with paclitaxel in anaplastic thyroid cancer: results of a multicenter phase 1 trial**. *J Clin Endocrinol Metab* 2013, **98**(6):2392-2400.

9. Kliewer SA, Sundseth SS, Jones SA, Brown PJ, Wisely GB, Koble CS, Devchand P, Wahli W, Willson TM, Lenhard JM *et al*: **Fatty acids and eicosanoids regulate gene expression through direct interactions with peroxisome proliferator-activated receptors alpha and gamma**. *Proc Natl Acad Sci U S A* 1997, **94**(9):4318-4323.

10. Varga T, Czimmerer Z, Nagy L: **PPARs are a unique set of fatty acid regulated transcription factors controlling both lipid metabolism and inflammation**. *Biochim Biophys Acta* 2011, **1812**(8):1007-1022.

11. Grommes C, Karlo JC, Caprariello A, Blankenship D, Dechant A, Landreth GE: **The PPARgamma agonist pioglitazone crosses the blood-brain barrier and reduces tumor growth in a human xenograft model**. *Cancer Chemother Pharmacol* 2013, **71**(4):929-936.

12. Deplanque D, Gelé P, Pétrault O, Six I, Furman C, Bouly M, Nion S, Dupuis B, Leys D, Fruchart J-C *et al*: **Peroxisome Proliferator-Activated Receptor-α Activation as a Mechanism of Preventive Neuroprotection Induced by Chronic Fenofibrate Treatment**. *The Journal of Neuroscience* 2003, **23**(15):6264-6271.

13. Youssef J, Badr M: **Peroxisome proliferator-activated receptors and cancer: challenges and opportunities**. *Br J Pharmacol* 2011, **164**(1):68-82.

14. Herrett E, Gallagher AM, Bhaskaran K, Forbes H, Mathur R, van Staa T, Smeeth L: **Data Resource Profile: Clinical Practice Research Datalink (CPRD)**. *Int J Epidemiol* 2015, **44**(3):827-836.

15. Herrett E, Thomas SL, Schoonen WM, Smeeth L, Hall AJ: **Validation and validity of diagnoses in the General Practice Research Database: a systematic review**. *Br J Clin Pharmacol* 2010, **69**(1):4-14.

16. Herrett E, Shah AD, Boggon R, Denaxas S, Smeeth L, van Staa T, Timmis A, Hemingway H: **Completeness and diagnostic validity of recording acute myocardial infarction events in primary care, hospital care, disease registry, and national mortality records: cohort study**. *BMJ* 2013, **346**:f2350.

For peer review only - http://bmjopen.bmj.com/site/about/guidelines.xhtml

17. Louis DN, Ohgaki H, Wiestler OD, Cavenee WK, Burger PC, Jouvet A, Scheithauer BW, Kleihues P: **The 2007 WHO classification of tumours of the central nervous system**. *Acta Neuropathol* 2007, **114**(2):97-109.
18. Suissa S, Dell'aniello S, Vahey S, Renoux C: **Time-window bias in case-control studies: statins and lung cancer**. *Epidemiology* 2011, **22**(2):228-231.
19. Levesque LE, Hanley JA, Kezouh A, Suissa S: **Problem of immortal time bias in cohort studies: example using statins for preventing progression of diabetes**. *BMJ* 2010, **340**:b5087.
20. Government DoCaL: **The English Indices of Deprivation 2015 Statistical Release**. In. Edited by Government DoCaL; 2015.
21. Pearce N: **Analysis of matched case-control studies**. *BMJ* 2016, **352**:i969.
22. Buuren Sv, Groothuis-Oudshoorn K: **mice: Multivariate Imputation by Chained Equations inR**. *Journal of Statistical Software* 2011, **45**(3).
23. Haynes HR, White P, Hares KM, Redondo J, Kemp KC, Singleton WGB, Killick-Cole CL, Stevens JR, Garadi K, Guglani S *et al*: **The transcription factor PPARalpha is overexpressed and is associated with a favourable prognosis in IDH-wildtype primary glioblastoma**. *Histopathology* 2017, **70**(7):1030-1043.
24. Binello E, Mormone E, Emdad L, Kothari H, Germano IM: **Characterization of fenofibrate-mediated anti-proliferative pro-apoptotic effects on high-grade gliomas and anti-invasive effects on glioma stem cells**. *J Neurooncol* 2014, **117**(2):225-234.
25. Wilk A, Urbanska K, Grabacka M, Mullinax J, Marcinkiewicz C, Impastato D, Estrada JJ, Reiss K: **Fenofibrate-induced nuclear translocation of FoxO3A triggers Bim-mediated apoptosis in glioblastoma cells in vitro**. *Cell Cycle* 2012, **11**(14):2660-2671.
26. Wilk A, Wyczechowska D, Zapata A, Dean M, Mullinax J, Marrero L, Parsons C, Peruzzi F, Culicchia F, Ochoa A *et al*: **Molecular mechanisms of fenofibrate-induced metabolic catastrophe and glioblastoma cell death**. *Mol Cell Biol* 2015, **35**(1):182-198.
27. Grommes C, Conway DS, Alshekhlee A, Barnholtz-Sloan JS: **Inverse association of PPARgamma agonists use and high grade glioma development**. *J Neurooncol* 2010, **100**(2):233-239.
28. Grommes C, Landreth GE, Sastre M, Beck M, Feinstein DL, Jacobs AH, Schlegel U, Heneka MT: **Inhibition of in Vivo Glioma Growth and Invasion by Peroxisome Proliferator-Activated Receptor γ Agonist Treatment**. *Molecular Pharmacology* 2006, **70**(5):1524-1533.
29. Perez-Ortiz JM, Tranque P, Vaquero CF, Domingo B, Molina F, Calvo S, Jordan J, Cena V, Llopis J: **Glitazones differentially regulate primary astrocyte and glioma cell survival. Involvement of reactive oxygen species and peroxisome proliferator-activated receptor-gamma**. *J Biol Chem* 2004, **279**(10):8976-8985.
30. Perez-Ortiz JM, Tranque P, Burgos M, Vaquero CF, Llopis J: **Glitazones induce astroglioma cell death by releasing reactive oxygen species from mitochondria: modulation of cytotoxicity by nitric oxide**. *Mol Pharmacol* 2007, **72**(2):407-417.
31. Panigrahy D, Kaipainen A, Huang S, Butterfield CE, Barnes CM, Fannon M, Laforme AM, Chaponis DM, Folkman J, Kieran MW: **PPARalpha agonist fenofibrate suppresses tumor growth through direct and indirect angiogenesis inhibition**. *Proc Natl Acad Sci U S A* 2008, **105**(3):985-990.
32. Jick SS, Kaye JA, Vasilakis-Scaramozza C, Garcia Rodriguez LA, Ruigomez A, Meier CR, Schlienger RG, Black C, Jick H: **Validity of the general practice research database**. *Pharmacotherapy* 2003, **23**(5):686-689.
33. Tong JJ, Tao H, Hui OT, Jian C: **Diabetes mellitus and risk of brain tumors: A meta-analysis**. *Exp Ther Med* 2012, **4**(5):877-882.
34. Alexandru O, Ene L, Purcaru OS, Tache DE, Popescu A, Neamtu OM, Tataranu LG, Georgescu AM, Tudorica V, Zaharia C *et al*: **Plasma levels of glucose and insulin in patients with brain tumors**. *Curr Health Sci J* 2014, **40**(1):27-36.

For peer review only

For peer review only - http://bmjopen.bmj.com/site/about/guidelines.xhtml

536 35. Panigraphy D, Singer S, Shen LQ, Butterfield CE, Freedman DA, Chen EJ, Moses MA, Kilroy S, Duensing S, Fletcher C *et al*: **PPARγ ligands inhibit primary tumor growth and metastasis by inhibiting angiogenesis.** *J. Clin. Invest*. 2002, **110**:923–932. doi:10.1172/JCI200215634

For peer review only

For peer review only - http://bmjopen.bmj.com/site/about/guidelines.xhtml

Read Code	Description	CPRD Medical Code	CPRD Description
B506.00	Malignant neoplasm of choroid	15991	Malignant neoplasm of choroid
B51..00	Malignant neoplasm of brain	18617	Malignant neoplasm of brain
B51..11	Cerebral tumour - malignant	10851	Cerebral tumour - malignant
B510.00	Malignant neoplasm cerebrum (excluding lobes and ventricles)	15711	Malignant neoplasm cerebrum (excluding lobes and ventricles)
B510000	Malignant neoplasm of basal ganglia	48073	Malignant neoplasm of basal ganglia
B510100	Malignant neoplasm of cerebral cortex	61399	Malignant neoplasm of cerebral cortex
B510300		99913	Malignant neoplasm of globus pallidus
B510400	Malignant neoplasm of hypothalamus	70942	Malignant neoplasm of hypothalamus
B510500	Malignant neoplasm of thalamus	62126	Malignant neoplasm of thalamus
B510z00	Malignant neoplasm of cerebrum NOS	54133	Malignant neoplasm of cerebrum NOS
B511.00	Malignant neoplasm of frontal lobe	42426	Malignant neoplasm of frontal lobe
B512.00	Malignant neoplasm of temporal lobe	46792	Malignant neoplasm of temporal lobe
B512000	Malignant neoplasm of hippocampus	67236	Malignant neoplasm of hippocampus
B512z00	Malignant neoplasm of temporal lobe NOS	47556	Malignant neoplasm of temporal lobe NOS
B513.00	Malignant neoplasm of parietal lobe	19226	Malignant neoplasm of parietal lobe
B514.00	Malignant neoplasm of occipital lobe	39088	Malignant neoplasm of occipital lobe
B515.00	Malignant neoplasm of cerebral ventricles	52511	Malignant neoplasm of cerebral ventricles
B515000	Malignant neoplasm of choroid plexus	46789	Malignant neoplasm of choroid plexus
B516.00	Malignant neoplasm of cerebellum	45154	Malignant neoplasm of cerebellum
B517.00	Malignant neoplasm of brain stem	44089	Malignant neoplasm of brain stem
B517000	Malignant neoplasm of cerebral peduncle	64557	Malignant neoplasm of cerebral peduncle
B517100	Malignant neoplasm of medulla oblongata	49132	Malignant neoplasm of medulla oblongata
B517200	Malignant neoplasm of midbrain	93537	Malignant neoplasm of midbrain
B517300	Malignant neoplasm of pons	91240	Malignant neoplasm of pons
B517z00	Malignant neoplasm of brain stem NOS	68641	Malignant neoplasm of brain stem NOS
B51y.00	Malignant neoplasm of other parts of brain	71139	Malignant neoplasm of other parts of brain
B51y000	Malignant neoplasm of corpus callosum	59170	Malignant neoplasm of corpus callosum
B51y200	Malignant neoplasm, overlapping lesion of brain	65241	Malignant neoplasm, overlapping lesion of brain
B51yz00		100733	Malignant neoplasm of other part of brain NOS
B51z.00	Malignant neoplasm of brain NOS	41520	Malignant neoplasm of brain NOS
B521.00	Malignant neoplasm of cerebral meninges	28919	Malignant neoplasm of cerebral meninges
B521200		109473	Malignant neoplasm of cerebral pia mater
B521z00	Malignant neoplasm of cerebral meninges NOS	70104	Malignant neoplasm of cerebral meninges NOS
B52X.00	Malignant neoplasm of meninges, unspecified	49875	Malignant neoplasm of meninges, unspecified

For peer review only

B542.00	Malignant neoplasm pituitary gland and craniopharyngeal duct	59823	Malignant neoplasm pituitary gland and craniopharyngeal duct
B542000	Malignant neoplasm of pituitary gland	8550	Malignant neoplasm of pituitary gland
B542z00	Malig neop pituitary gland or craniopharyngeal duct NOS	59718	Malig neop pituitary gland or craniopharyngeal duct NOS
B543.00	Malignant neoplasm of pineal gland	42460	Malignant neoplasm of pineal gland
B583.00	Secondary malignant neoplasm of brain and spinal cord	33843	Secondary malignant neoplasm of brain and spinal cord
B583000	Secondary malignant neoplasm of brain	5198	Secondary malignant neoplasm of brain
B583200	Cerebral metastasis	5199	Cerebral metastasis
B583z00	Secondary malignant neoplasm of brain or spinal cord NOS	59375	Secondary malignant neoplasm of brain or spinal cord NOS
B8yy300	Carcinoma in situ of pituitary gland	45909	Carcinoma in situ of pituitary gland
BA06.00	Neoplasm of unspecified nature of brain	1044	Neoplasm of unspecified nature of brain
BB9G.00	[M]Infiltrating ductular carcinoma	7319	[M]Infiltrating ductular carcinoma
BBa3.00	[M]Pineoblastoma	50151	[M]Pineoblastoma
BBb..00	[M]Gliomas	12309	[M]Gliomas
BBb0.00	[M]Glioma, malignant	31574	[M]Glioma, malignant
BBb0.11	[M]Glioma NOS	8523	[M]Glioma NOS
BBb0.12	[M]Gliosarcoma	34252	[M]Gliosarcoma
BBb1.00	[M]Gliomatosis cerebri	38551	[M]Gliomatosis cerebri
BBb2.00	[M]Mixed glioma	68808	[M]Mixed glioma
BBb2.11	[M]Mixed glioma	39386	[M]Mixed glioma
BBb3.00	[M]Subependymal glioma	94267	[M]Subependymal glioma
BBb3.11	[M]Subependymal astrocytoma NOS	90487	[M]Subependymal astrocytoma NOS
BBb3.12	[M]Subependymal astrocytoma NOS	28344	[M]Subependymal astrocytoma NOS
BBb4.00	[M]Subependymal giant cell astrocytoma	49168	[M]Subependymal giant cell astrocytoma
BBb8.00	[M]Ependymoma, anaplastic type	52751	[M]Ependymoma, anaplastic type
BBb8.11	[M]Ependymoblastoma	46769	[M]Ependymoblastoma
BBbA.00	[M]Myxopapillary ependymoma	43114	[M]Myxopapillary ependymoma
BBba.00	[M]Primitive neuroectodermal tumour	41695	[M]Primitive neuroectodermal tumour
BBbB.00	[M]Astrocytoma NOS	8547	[M]Astrocytoma NOS
BBbB.11	[M]Astrocytic glioma	27748	[M]Astrocytic glioma
BBbC.00	[M]Astrocytoma, anaplastic type	8328	[M]Astrocytoma, anaplastic type
BBbE.00	[M]Gemistocytic astrocytoma	45531	[M]Gemistocytic astrocytoma
BBbF.00	[M]Fibrillary astrocytoma	27846	[M]Fibrillary astrocytoma
BBbG.00	[M]Pilocytic astrocytoma	30273	[M]Pilocytic astrocytoma
BBbG.11	[M]Juvenile astrocytoma	61783	[M]Juvenile astrocytoma
BBbG.12	[M]Piloid astrocytoma	98800	[M]Piloid astrocytoma
BBbH.00		103047	[M]Spongioblastoma NOS
BBbK.00	[M]Astroblastoma	50235	[M]Astroblastoma
BBbL.00	[M]Glioblastoma NOS	23083	[M]Glioblastoma NOS
BBbL.11	[M]Glioblastoma multiforme	9575	[M]Glioblastoma multiforme
BBbM.00	[M]Giant cell glioblastoma	66064	[M]Giant cell glioblastoma
BBbQ.00	[M]Oligodendroglioma NOS	27744	[M]Oligodendroglioma NOS
BBbR.00	[M]Oligodendroglioma, anaplastic type	49186	[M]Oligodendroglioma, anaplastic type

For peer review

BBbS.00	[M]Oligodendroblastoma	46404	[M]Oligodendroblastoma
BBbT.00	[M]Medulloblastoma NOS	34763	[M]Medulloblastoma NOS
BBbU.00	[M]Desmoplastic medulloblastoma	65952	[M]Desmoplastic medulloblastoma
BBbV.00	[M]Medullomyoblastoma	31767	[M]Medullomyoblastoma
BBbW.00	[M]Cerebellar sarcoma NOS	37473	[M]Cerebellar sarcoma NOS
BBbZ.00	[M]Pleomorphic xanthoastrocytoma	67587	[M]Pleomorphic xanthoastrocytoma
BBbz.00	[M]Glioma NOS	27653	[M]Glioma NOS
BBc6.00	[M]Ganglioglioma	31629	[M]Ganglioglioma
BBc7.11	[M]Neuroastrocytoma	68479	[M]Neuroastrocytoma
BBd1.00	[M]Meningiomatosis NOS	98677	[M]Meningiomatosis NOS
BBd1.11	[M]Diffuse meningiomatosis	95108	[M]Diffuse meningiomatosis
BBd2.00	[M]Meningioma, malignant	27363	[M]Meningioma, malignant
BBd2.11	[M]Leptomeningeal sarcoma	60347	[M]Leptomeningeal sarcoma
BBd2.12	[M]Meningothelial sarcoma	96798	[M]Meningothelial sarcoma
BBd5.00	[M]Psammomatous meningioma	38870	[M]Psammomatous meningioma
BBd7.11	[M]Angioblastic meningioma	46490	[M]Angioblastic meningioma
BBdB.00		106134	[M]Meningeal sarcomatosis
BBdz.00	[M]Meningioma NOS	47848	[M]Meningioma NOS
BBm0.00	[M]Microglioma	63973	[M]Microglioma
ByuA200	[X]Malignant neoplasm of meninges, unspecified	63925	[X]Malignant neoplasm of meninges, unspecified
ByuA300	[X]Malig neopl, overlap lesion brain & other part of CNS	47633	[X]Malig neopl, overlap lesion brain & other part of CNS

Supplemental table 1 Read and CPRD Medical Codes used to identify primary and secondary brain tumours.

For peer review only

Product Code	Product Name	PPARa Targeting?
25	Simvastatin 20mg tablets	No
28	Atorvastatin 10mg tablets	No
42	Simvastatin 10mg tablets	No
51	Simvastatin 40mg tablets	No
75	Atorvastatin 20mg tablets	No
184	Bezafibrate 200mg tablets	Yes
379	Fluvastatin 20mg capsules	No
490	Pravastatin 10mg tablets	No
602	Bezafibrate 400mg modified-release tablets	Yes
644	Colestyramine 4g oral powder sachets	No
653	Ezetimibe 10mg tablets	No
713	Rosuvastatin 10mg tablets	No
730	Pravastatin 20mg tablets	No
745	Atorvastatin 40mg tablets	No
802	Simvador 40mg tablets (Discovery Pharmaceuticals)	No
818	Simvastatin 20mg/5ml oral solution sugar free	No
1212	Colestipol 5g granules sachets sugar free	No
1214	Bezalip 400mg Tablet (Roche Products Ltd)	Yes
1215	Fenofibrate 100mg Capsule	Yes
1217	Lipantil micro 200 200mg Capsule (Fournier Pharmaceuticals Ltd)	Yes
1219	Pravastatin 40mg tablets	No
1221	Lipostat 10mg tablets (Bristol-Myers Squibb Pharmaceuticals Ltd)	No
1223	Lipostat 40mg tablets (Bristol-Myers Squibb Pharmaceuticals Ltd)	No
1322	Clofibrate 500mg capsules	Yes
1324	Bezalip 200mg Tablet (Roche Products Ltd)	Yes
1477	Atromid -s 500mg Capsule (AstraZeneca UK Ltd)	Yes
1716	Questran 4g oral powder sachets (Bristol-Myers Squibb Pharmaceuticals Ltd)	No
1764	Questran Light 4g oral powder sachets (Bristol-Myers Squibb Pharmaceuticals Ltd)	No
2137	Fluvastatin 40mg capsules	No
2215	Lopid 300mg capsules (Pfizer Ltd)	Yes
2435	Lipantil 100mg Capsule (Fournier Pharmaceuticals Ltd)	Yes
2718	Zocor 10mg tablets (Merck Sharp & Dohme Ltd)	No
2955	Lipitor 40mg tablets (Pfizer Ltd)	No
3089	Ciprofibrate 100mg tablets	Yes
3159	Fenofibrate 200mg capsules	Yes
3318	Gemfibrozil 300mg capsules	Yes
3411	Lipitor 10mg tablets (Pfizer Ltd)	No
3690	Lipostat 20mg tablets (Bristol-Myers Squibb Pharmaceuticals Ltd)	No
4062	Lopid 600mg tablets (Pfizer Ltd)	Yes
4067	Olbetam 250mg capsules (Pfizer Ltd)	No
4920	Fenofibrate micronised 200mg capsules	Yes
4928	Lipantil Micro 200 capsules (BGP Products Ltd)	Yes
5148	Simvastatin 80mg tablets	No
5216	Bezalip mono 400mg Modified-release tablet (Roche Products Ltd)	Yes
5390	Fenofibrate micronised 267mg capsules	Yes
5564	Colestid Orange sachets (Pharmacia Ltd)	No
5775	Atorvastatin 80mg tablets	No
5985	Lescol XL 80mg tablets (Novartis Pharmaceuticals UK Ltd)	No
6120	Ezetrol 10mg tablets (Merck Sharp & Dohme Ltd)	No
6155	Colestyramine with aspartame 4g sugar free powder	No
6168	Zocor 40mg tablets (Merck Sharp & Dohme Ltd)	No
6213	Rosuvastatin 20mg tablets	No

For peer review only

For peer review only - http://bmjopen.bmj.com/site/about/guidelines.xhtml

6365	Colestid 5g granules sachets plain (Pfizer Ltd)	No	11815	Simvastatin 20mg with ezetimibe 10mg tablet	No
7196	Zocor 20mg tablets (Merck Sharp & Dohme Ltd)	No	11976	Niaspan 500mg modified-release tablets (Abbott Laboratories Ltd)	No
7347	Crestor 10mg tablets (AstraZeneca UK Ltd)	No	12211	Nicotinic acid 50mg tablets	No
7374	Lipitor 20mg tablets (Pfizer Ltd)	No	13041	Simvador 10mg tablets (Discovery Pharmaceuticals)	No
7540	Lipantil Micro 267 capsules (BGP Products Ltd)	Yes	14219	Simvastatin 80mg / Ezetimibe 10mg tablets	No
7544	Niaspan 750mg modified-release tablets (Abbott Laboratories Ltd)	No	14379	Lipantil Micro 67 capsules (BGP Products Ltd)	Yes
			14963	Nicotinic acid 500mg modified-release tablets	No
7551	Niaspan 1g modified-release tablets (Abbott Laboratories Ltd)	No	15252	Crestor 20mg tablets (AstraZeneca UK Ltd)	No
7552	Simvastatin 20mg / Ezetimibe 10mg tablets	No	16186	Inegy 10mg/80mg tablets (Merck Sharp & Dohme Ltd)	No
7554	Rosuvastatin 5mg tablets	No			
8082	Gemfibrozil 600mg tablets	Yes	17059	Inegy 10mg/40mg tablets (Merck Sharp & Dohme Ltd)	No
8104	Acipimox 250mg capsules	No			
8380	Lescol 20mg capsules (Novartis Pharmaceuticals UK Ltd)	No	17614	Zimbacol XL 400mg tablets (Archimedes Pharma UK Ltd)	Yes
8706	Modalim 100mg tablets (Sanofi)	Yes	17683	Lipitor 80mg tablets (Pfizer Ltd)	No
9153	Lescol 40mg capsules (Novartis Pharmaceuticals UK Ltd)	No	17688	Crestor 5mg tablets (AstraZeneca UK Ltd)	No
9491	Fenofibrate micronised 67mg capsules	Yes	17813	Nicotinic acid 100mg Tablet	No
9639	Fenofibrate micronised 160mg tablets	Yes	17824	Nicotinic acid 25mg Tablet	No
			18081	Colestid Orange 5g granules sachets (Pfizer Ltd)	No
9716	Supralip 160mg tablets (BGP Products Ltd)	Yes			
9897	Rosuvastatin 40mg tablets	No	18098	Nicotinic acid 375mg + 500mg + 750mg Modified-release tablet	No
9920	Simvador 20mg tablets (Discovery Pharmaceuticals)	No			
			18126	Nicotinic acid 1g modified-release tablets	No
9930	Crestor 40mg tablets (AstraZeneca UK Ltd)	No	19938	Colestipol with aspartame granules	No
10172	Simvastatin 40mg / Ezetimibe 10mg tablets	No	21020	Inegy 10mg/20mg tablets (Merck Sharp & Dohme Ltd)	No
10183	Simvastatin 40mg with ezetimibe 10mg tablet	No			
10206	Simvastatin 80mg with ezetimibe 10mg tablet	No	22579	Zocor 80mg tablets (Merck Sharp & Dohme Ltd)	No
11627	Fluvastatin 80mg modified-release tablets	No	23153	Liparol 400 XL tablets (Ashbourne Pharmaceuticals Ltd)	Yes
11785	Colestyramine 4g oral powder sachets sugar free	No			

23153	Liparol 400 XL tablets (Ashbourne Pharmaceuticals Ltd)	No	34316	Simvastatin 20mg tablets (Teva UK Ltd)	No
23634	GEMFIBROZIL	Yes	34353	Simvastatin 40mg tablets (Mylan Ltd)	No
24009	NICOTINIC ACID 500 MG TAB	No	34366	Simvastatin 20mg tablets (IVAX Pharmaceuticals UK Ltd)	No
24084	Colestyramine 4g oral powder sachets sugar free (PLIVA Pharma Ltd)	No	34376	Simvastatin 40mg tablets (Teva UK Ltd)	No
24509	SIMVASTATIN	No	34381	Simvastatin 40mg tablets (IVAX Pharmaceuticals UK Ltd)	No
24583	Nicotinic acid 750mg modified-release tablets	No	34476	Simvastatin 20mg Tablet (Ratiopharm UK Ltd)	No
25018	BEZAFIBRATE	Yes	34481	Simvastatin 10mg tablets (IVAX Pharmaceuticals UK Ltd)	No
29213	Bezagen XL 400mg tablets (Mylan Ltd)	Yes	34502	Simvastatin 40mg tablets (A A H Pharmaceuticals Ltd)	No
29328	Bezafibrate 200mg tablets (A A H Pharmaceuticals Ltd)	Yes	34535	Simvastatin 10mg tablets (Mylan Ltd)	No
29438	SIMVASTATIN	No	34545	Simvastatin 40mg Tablet (Ratiopharm UK Ltd)	No
31221	Bezafibrate 200mg tablets (Mylan Ltd)	Yes	34560	Simvastatin 10mg Tablet (Ratiopharm UK Ltd)	No
31783	Fenogal 200mg capsules (Genus Pharmaceuticals Ltd)	Yes	34746	Simvastatin 20mg Tablet (Niche Generics Ltd)	No
31930	Zocor heart-pro 10mg Tablet (McNeil Products Ltd)	No	34814	Simvastatin 20mg tablets (Wockhardt UK Ltd)	No
32110	Colestyramine 4g Sachets (Dominion Pharma)	No	34820	Pravastatin 40mg tablets (A A H Pharmaceuticals Ltd)	No
32909	Simvastatin 80mg tablets (A A H Pharmaceuticals Ltd)	No	34879	Simvastatin 40mg Tablet (Niche Generics Ltd)	No
32921	Pravastatin 10mg Tablet (Dr Reddy's Laboratories (UK) Ltd)	No	34891	Simvastatin 20mg tablets (Kent Pharmaceuticals Ltd)	No
33082	Simvastatin 20mg tablets (A A H Pharmaceuticals Ltd)	No	34907	Simvastatin 40mg tablets (Wockhardt UK Ltd)	No
33603	Fibrazate XL 400mg tablets (Sandoz Ltd)	Yes	34955	Simvastatin 10mg tablets (A A H Pharmaceuticals Ltd)	No
33944	Bezafibrate 200mg tablets (Teva UK Ltd)	Yes	34969	Simvastatin 40mg tablets (Actavis UK Ltd)	No
34181	Bezafibrate 400mg Modified-release tablet (Hillcross Pharmaceuticals Ltd)	Yes	36377	Pravastatin 20mg tablets (Teva UK Ltd)	No
34201	Colestyramine 4g oral powder sachets sugar free (Actavis UK Ltd)	No	37266	Colesevelam 625mg tablets	No
34277	Gemfibrozil 600mg tablets (Teva UK Ltd)	Yes	37434	Simvastatin 40mg tablets (Sandoz Ltd)	No
34312	Simvastatin 20mg tablets (Mylan Ltd)	No	37953	Cholestagel 625mg tablets (Sanofi)	No

For peer review only

39060	Simvastatin 20mg tablets (Dexcel-Pharma Ltd)	No
39420	Bezalip Mono 400mg modified-release tablets (Teva UK Ltd)	Yes
39576	Bezalip 200mg tablets (Teva UK Ltd)	Yes
39652	Simvastatin 40mg/5ml oral solution sugar free	No
39675	Simvastatin 20mg/5ml Oral suspension (Martindale Pharmaceuticals Ltd)	No
39870	Simvador 80mg tablets (Discovery Pharmaceuticals)	No
40340	Simvastatin 10mg tablets (Teva UK Ltd)	No
40382	Pravastatin 20mg tablets (A A H Pharmaceuticals Ltd)	No
40601	Simvastatin 20mg tablets (Ranbaxy (UK) Ltd)	No
40729	Tredaptive 1000mg/20mg modified-release tablets (Merck Sharp & Dohme Ltd)	No
40885	Nicotinic acid 1g / Laropiprant 20mg modified-release tablets	No
41396	Fenofibrate micronised 200mg capsules (A A H Pharmaceuticals Ltd)	Yes
41657	Simvastatin 80mg tablets (Teva UK Ltd)	No
42801	Bezafibrate xl 400mg Modified-release tablet (Generics (UK) Ltd)	Yes
43218	Pravastatin 10mg tablets (Teva UK Ltd)	No
44528	Simvastatin 20mg/5ml oral suspension sugar free (Rosemont Pharmaceuticals Ltd)	No
44650	Simvastatin 40mg tablets (Dexcel-Pharma Ltd)	No
44878	Ranzolont 10mg tablets (Ranbaxy (UK) Ltd)	No
45219	Simvastatin 40mg tablets (Kent Pharmaceuticals Ltd)	No
45235	Simvastatin 20mg tablets (Sandoz Ltd)	No
45245	Simvastatin 20mg tablets (Actavis UK Ltd)	No
45346	Simvastatin 40mg tablets (Arrow Generics Ltd)	No
46878	Simvastatin 40mg tablets (Almus Pharmaceuticals Ltd)	No
46956	Simvastatin 80mg tablets (Arrow Generics Ltd)	No
47023	Omega-3 fish oil with glycerol Emulsion for infusion	No
47065	Atorvastatin 20mg chewable tablets sugar free	No
47090	Atorvastatin 10mg chewable tablets sugar free	No
47630	Lipitor 20mg chewable tablets (Pfizer Ltd)	No
47721	Lipitor 10mg chewable tablets (Pfizer Ltd)	No
47774	Simvastatin 10mg tablets (Arrow Generics Ltd)	No
47935	Fenofibrate 200mg Capsule (Teva UK Ltd)	Yes
47948	Simvastatin 10mg tablets (Tillomed Laboratories Ltd)	No
47988	Pravastatin 40mg tablets (Mylan Ltd)	No
48018	Simvastatin 20mg tablets (Arrow Generics Ltd)	No
48051	Simvastatin 10mg tablets (Kent Pharmaceuticals Ltd)	No
48058	Simvastatin 10mg tablets (Ranbaxy (UK) Ltd)	No
48078	Simvastatin 10mg tablets (Actavis UK Ltd)	No
48097	Pravastatin 40mg tablets (Teva UK Ltd)	No
48221	Simvastatin 20mg/5ml oral suspension sugar free	No
48346	Atorvastatin 60mg tablets	No
48431	Simvastatin 40mg/5ml oral suspension sugar free	No
48518	Atorvastatin 10mg/5ml oral solution	No
48585	Nicotinic acid 500mg capsules	No
48867	Simvastatin 40mg tablets (Alliance Healthcare (Distribution) Ltd)	No

For peer review only

48973	Atorvastatin 30mg tablets	No
49061	Simvastatin 40mg tablets (Bristol Laboratories Ltd)	No
49062	Simvastatin 20mg tablets (Alliance Healthcare (Distribution) Ltd)	No
49558	Atorvastatin 20mg tablets (A A H Pharmaceuticals Ltd)	No
49587	Simvastatin 80mg tablets (Almus Pharmaceuticals Ltd)	No
49609	Bezafibrate 400mg Modified-release tablet (Sandoz Ltd)	Yes
49751	Atorvastatin 40mg tablets (Alliance Healthcare (Distribution) Ltd)	No
50071	Fenofibrate 160mg Tablet (Teva UK Ltd)	Yes
50236	Atorvastatin 10mg tablets (Zentiva)	No
50272	Atorvastatin 40mg tablets (Pfizer Ltd)	No
50483	Simvastatin 40mg tablets (Relonchem Ltd)	No
50564	Simvastatin 20mg tablets (Relonchem Ltd)	No
50670	Simvastatin 40mg tablets (Aurobindo Pharma Ltd)	No
50703	Simvastatin 40mg tablets (Accord Healthcare Ltd)	No
50754	Simvastatin 20mg tablets (Medreich Plc)	No
50788	Atorvastatin 20mg tablets (Pfizer Ltd)	No
50790	Atorvastatin 20mg tablets (Dexcel-Pharma Ltd)	No
50882	Simvastatin 40mg tablets (Somex Pharma)	No
50925	Pravastatin 10mg tablets (Sigma Pharmaceuticals Plc)	No
50963	Atorvastatin 40mg tablets (Teva UK Ltd)	No
51085	Simvastatin 10mg tablets (Medreich Plc)	No
51134	Atorvastatin 10mg tablets (A A H Pharmaceuticals Ltd)	No
51155	Natures Aid Omega-3 Fish Oil 1000mg softgels capsules (Natures Aid Ltd)	No

51166	Simvastatin 40mg tablets (Medreich Plc)	No
51200	Atorvastatin 40mg tablets (Arrow Generics Ltd)	No
51233	Simvastatin 10mg tablets (Alliance Healthcare (Distribution) Ltd)	No
51359	Atorvastatin 20mg tablets (Arrow Generics Ltd)	No
51483	Simvastatin 20mg tablets (Aurobindo Pharma Ltd)	No
51622	Atorvastatin 20mg tablets (Consilient Health Ltd)	No
51676	Pravastatin 40mg tablets (Medreich Plc)	No
51715	Simvastatin 10mg tablets (Sigma Pharmaceuticals Plc)	No
51822	Natures Aid Omega-3 Fish Oil 500mg capsules (Natures Aid Ltd)	No
51876	Atorvastatin 40mg tablets (Consilient Health Ltd)	No
51890	Pravastatin 20mg tablets (Medreich Plc)	No
52097	Atorvastatin 40mg tablets (Wockhardt UK Ltd)	No
52098	Simvastatin 40mg tablets (Ranbaxy (UK) Ltd)	No
52168	Atorvastatin 20mg tablets (Aspire Pharma Ltd)	No
52211	Atorvastatin 20mg tablets (Actavis UK Ltd)	No
52257	Simvastatin 20mg tablets (Accord Healthcare Ltd)	No
52397	Atorvastatin 40mg tablets (Dr Reddy's Laboratories (UK) Ltd)	No
52398	Atorvastatin 40mg tablets (A A H Pharmaceuticals Ltd)	No
52459	Atorvastatin 80mg tablets (Actavis UK Ltd)	No
52460	Atorvastatin 40mg tablets (Aspire Pharma Ltd)	No
52625	Simvastatin 10mg tablets (Wockhardt UK Ltd)	No
52676	Simvastatin 10mg/5ml oral suspension	No
52755	Pravastatin 20mg tablets (Alliance Healthcare (Distribution) Ltd)	No

For peer review only

52812	Simvastatin 20mg tablets (Sigma Pharmaceuticals Plc)	No
52814	Bezafibrate 400mg modified-release tablets (Alliance Healthcare (Distribution) Ltd)	Yes
52821	Atorvastatin 80mg tablets (Dr Reddy's Laboratories (UK) Ltd)	No
52953	Simvastatin 20mg tablets (Bristol Laboratories Ltd)	No
52962	Simvastatin 80mg tablets (Medreich Plc)	No
53087	Simvastatin 20mg tablets (Somex Pharma)	No
53250	Modalim 100mg tablets (Lexon (UK) Ltd)	Yes
53340	Zocor 40mg tablets (Lexon (UK) Ltd)	No
53415	Simvastatin 10mg tablets (Aurobindo Pharma Ltd)	No
53460	Crestor 10mg tablets (DE Pharmaceuticals)	No
53594	Lipitor 80mg tablets (Mawdsley-Brooks & Company Ltd)	No
53676	Simvastatin 20mg tablets (Tillomed Laboratories Ltd)	No
53770	Fluvastatin 40mg capsules (A A H Pharmaceuticals Ltd)	No
53772	Atorvastatin 80mg tablets (Alliance Healthcare (Distribution) Ltd)	No
53822	Simvastatin 10mg tablets (Bristol Laboratories Ltd)	No
53887	Atorvastatin 40mg tablets (Actavis UK Ltd)	No
53890	Atorvastatin 80mg tablets (Pfizer Ltd)	No
53908	Simvastatin 10mg tablets (Dexcel-Pharma Ltd)	No
53966	Simvastatin 40mg tablets (Phoenix Healthcare Distribution Ltd)	No
54240	Simvastatin 40mg tablets (Sigma Pharmaceuticals Plc)	No
54266	Simvastatin 20mg/5ml oral suspension	No
54435	Pravastatin 40mg tablets (Almus Pharmaceuticals Ltd)	No
54493	Simvastatin 10mg tablets (Relonchem Ltd)	No
54535	Atorvastatin 10mg tablets (Pfizer Ltd)	No
54606	Simvastatin 20mg/5ml oral suspension sugar free (A A H Pharmaceuticals Ltd)	No
54607	Pravastatin 20mg tablets (Almus Pharmaceuticals Ltd)	No
54655	Simvastatin 10mg tablets (Accord Healthcare Ltd)	No
54819	Simvastatin 40mg/5ml oral suspension sugar free (Rosemont Pharmaceuticals Ltd)	No
54947	Simvastatin 20mg tablets (Almus Pharmaceuticals Ltd)	No
54976	Simvastatin 10mg tablets (Somex Pharma)	No
54985	Simvastatin 40mg/5ml oral suspension	No
54992	Atorvastatin 10mg/5ml oral suspension	No
55032	Atorvastatin 10mg tablets (Dexcel-Pharma Ltd)	No
55034	Atorvastatin 40mg/5ml oral suspension	No
55444	Atorvastatin 40mg tablets (Zentiva)	No
55452	Simvastatin 20mg tablets (Phoenix Healthcare Distribution Ltd)	No
55727	Atorvastatin 10mg tablets (Actavis UK Ltd)	No
55912	Pravastatin 40mg tablets (Alliance Healthcare (Distribution) Ltd)	No
56065	Simvastatin 20mg/5ml oral suspension sugar free (Waymade Healthcare Plc)	No
56097	Atorvastatin 10mg chewable tablets sugar free	No
56146	Pravastatin 10mg tablets (Waymade Healthcare Plc)	No

For peer review only

56165	Atorvastatin 20mg chewable tablets sugar free	No
56182	Atorvastatin 80mg tablets (Zentiva)	No
56248	Atorvastatin 20mg tablets (Sigma Pharmaceuticals Plc)	No
56481	Zocor 10mg tablets (Sigma Pharmaceuticals Plc)	No
56494	Zocor 20mg tablets (Sigma Pharmaceuticals Plc)	No
56564	Atorvastatin 20mg tablets (Almus Pharmaceuticals Ltd)	No
56607	Pravastatin 20mg tablets (Waymade Healthcare Plc)	No
56735	Pravastatin 20mg tablets (Mylan Ltd)	No
56841	Atorvastatin 40mg tablets (Dexcel-Pharma Ltd)	No
56893	Pravastatin 40mg tablets (Accord Healthcare Ltd)	No
56916	Pravastatin 40mg tablets (PLIVA Pharma Ltd)	No
57108	Pravastatin 40mg tablets (Waymade Healthcare Plc)	No
57117	Atorvastatin 80mg tablets (Waymade Healthcare Plc)	No
57137	Pravastatin 10mg tablets (Almus Pharmaceuticals Ltd)	No
57219	Fenofibrate micronised 200mg capsules (Sandoz Ltd)	Yes
57296	Pravastatin 20mg tablets (Phoenix Healthcare Distribution Ltd)	No
57329	Simvastatin 25mg/5ml oral suspension	No
57348	Atorvastatin 10mg tablets (Consilient Health Ltd)	No
57397	Pravastatin 10mg tablets (Accord Healthcare Ltd)	No
57489	Ciprofibrate 100mg tablets (Zentiva)	Yes
57568	Zocor 10mg tablets (Lexon (UK) Ltd)	No
57763	Rosuvastatin 10mg tablets (Waymade Healthcare Plc)	No
57834	Atorvastatin 40mg tablets (DE Pharmaceuticals)	No
57836	Atorvastatin 80mg tablets (Teva UK Ltd)	No
57999	Crestor 40mg tablets (Lexon (UK) Ltd)	No
58041	Atorvastatin 20mg tablets (Teva UK Ltd)	No
58110	Atorvastatin 20mg tablets (Zentiva)	No
58315	Simvastatin 20mg tablets (Waymade Healthcare Plc)	No
58394	Atorvastatin 20mg tablets (Alliance Healthcare (Distribution) Ltd)	No
58418	Atorvastatin 80mg tablets (A A H Pharmaceuticals Ltd)	No
58617	Rosuvastatin 20mg/5ml oral suspension	No
58635	Bezalip Mono 400mg modified-release tablets (DE Pharmaceuticals)	Yes
58742	Atorvastatin 80mg tablets (Arrow Generics Ltd)	No
58755	Simvastatin 10mg tablets (Phoenix Healthcare Distribution Ltd)	No
58834	Atorvastatin 10mg tablets (DE Pharmaceuticals)	No
58868	Atorvastatin 10mg tablets (Sigma Pharmaceuticals Plc)	No
59002	Bezafibrate 400mg modified-release tablets (DE Pharmaceuticals)	Yes
59272	Atorvastatin 20mg tablets (Waymade Healthcare Plc)	No
59278	Fluvastatin 20mg capsules (Zentiva)	No
59331	Lipitor 10mg tablets (DE Pharmaceuticals)	No
59357	Atorvastatin 10mg tablets (Ranbaxy (UK) Ltd)	No
59446	Atorvastatin 40mg tablets (Almus Pharmaceuticals Ltd)	No

For peer review only

59447	Crestor 20mg tablets (Waymade Healthcare Plc)	No	61360	Simvastatin 10mg tablets (Almus Pharmaceuticals Ltd)	No
59452	Rosuvastatin 5mg tablets (Waymade Healthcare Plc)	No	61665	Simvastatin 10mg tablets (Waymade Healthcare Plc)	No
59508	Pravastatin 20mg tablets (Accord Healthcare Ltd)	No	62137	Simvastatin 40mg tablets (Waymade Healthcare Plc)	No
59776	Atorvastatin 80mg tablets (Aspire Pharma Ltd)	No	62148	Fluvastatin 20mg capsules (Actavis UK Ltd)	No
59859	Atorvastatin 10mg tablets (Teva UK Ltd)	No	62219	Atorvastatin 20mg tablets (DE Pharmaceuticals)	No
60101	Colestyramine 4g oral powder sachets sugar free (Teva UK Ltd)	No	62429	Atorvastatin 20mg tablets (DE Pharmaceuticals)	No
60160	Rosuvastatin 5mg tablets (Mawdsley-Brooks & Company Ltd)	No	62476	Atorvastatin 80mg tablets (Almus Pharmaceuticals Ltd)	No
60251	Pravastatin 10mg tablets (Sandoz Ltd)	No	62979	Pravastatin 40mg tablets (Kent Pharmaceuticals Ltd)	No
60342	Berocca effervescent tablets tropical (Bayer Plc)	No	63074	Pravastatin 20mg tablets (PLIVA Pharma Ltd)	No
60385	Bezalip Mono 400mg modified-release tablets (Lexon (UK) Ltd)	Yes	63140	Atorvastatin 10mg tablets (Alliance Healthcare (Distribution) Ltd)	No
60464	Atorvastatin 20mg/5ml oral suspension	No	63249	Atorvastatin 80mg tablets (Consilient Health Ltd)	No
60511	Atorvastatin 40mg tablets (Ranbaxy (UK) Ltd)	No	63343	Generic Crampex tablets	No
60607	Atorvastatin 80mg tablets (DE Pharmaceuticals)	No	63469	Atorvastatin 30mg tablets (Consilient Health Ltd)	No
60788	Fenofibrate micronised 267mg capsules (Zentiva)	Yes	63737	Fenofibrate micronised 267mg capsules (Sigma Pharmaceuticals Plc)	Yes
60989	Atorvastatin 80mg tablets (Phoenix Healthcare Distribution Ltd)	No	63787	Pravastatin 10mg tablets (Tillomed Laboratories Ltd)	No
61087	Questran Light 4g oral powder sachets (Mawdsley-Brooks & Company Ltd)	No	64067	Atorvastatin 20mg/5ml oral solution	No
61134	Pravastatin 20mg tablets (Sigma Pharmaceuticals Plc)	No	64104	Simvastatin 20mg tablets (Crescent Pharma Ltd)	No
61149	Atorvastatin 10mg tablets (Waymade Healthcare Plc)	No	64180	Simvastatin 10mg tablets (Crescent Pharma Ltd)	No
61155	Simvastatin 40mg/5ml oral suspension sugar free (A A H Pharmaceuticals Ltd)	No	64307	Simvastatin 40mg tablets (Crescent Pharma Ltd)	No
61321	Simvastatin 10mg tablets (Sandoz Ltd)	No	64503	Bezalip Mono 400mg modified-release tablets (Waymade Healthcare Plc)	Yes
			64702	Atorvastatin 30mg tablets (A A H Pharmaceuticals Ltd)	No

For peer review only

64810	Atorvastatin 40mg tablets (Phoenix Healthcare Distribution Ltd)	No
64825	Atorvastatin 10mg tablets (Phoenix Healthcare Distribution Ltd)	No
64868	Atorvastatin 40mg tablets (Sigma Pharmaceuticals Plc)	No
64933	Fenofibrate micronised 267mg capsules (Ranbaxy (UK) Ltd)	Yes
64968	Simvastatin 10mg tablets (DE Pharmaceuticals)	No
64984	Fenofibrate micronised 160mg tablets (Phoenix Healthcare Distribution Ltd)	Yes
65181	Simvastatin 40mg tablets (DE Pharmaceuticals)	No
65193	Atorvastatin 20mg tablets (Ranbaxy (UK) Ltd)	No
65572	Fenofibrate micronised 160mg tablets (Genus Pharmaceuticals Ltd)	Yes
65679	Simvastatin 20mg tablets (DE Pharmaceuticals)	No
65901	Simvastatin 40mg tablets (Zentiva)	No
65925	Simvastatin 20mg/5ml oral suspension sugar free (Alliance Healthcare (Distribution) Ltd)	No
66087	Modalim 100mg tablets (Mawdsley-Brooks & Company Ltd)	Yes
66425	Bezafibrate 400mg modified-release tablets (A A H Pharmaceuticals Ltd)	Yes
66505	Fenofibrate 145mg / Simvastatin 40mg tablets	Yes
66564	Bezafibrate 400mg modified-release tablets (Phoenix Healthcare Distribution Ltd)	Yes
66780	Fenofibrate 145mg / Simvastatin 20mg tablets	Yes
66963	Atorvastatin 80mg tablets (Sigma Pharmaceuticals Plc)	No
67098	Simvastatin 10mg tablets (Brown & Burk UK Ltd)	No
67157	Fenofibrate micronised 200mg capsules (Phoenix Healthcare Distribution Ltd)	Yes
67328	Lescol XL 80mg tablets (Mawdsley-Brooks & Company Ltd)	No
67329	Lipantil Micro 267 capsules (DE Pharmaceuticals)	Yes
67402	Atorvastatin 40mg tablets (Kent Pharmaceuticals Ltd)	No
67573	Atorvastatin 10mg tablets (DE Pharmaceuticals)	No
67660	Atorvastatin 80mg tablets (Ranbaxy (UK) Ltd)	No
67745	Simvastatin 10mg tablets (Zentiva)	No
67773	Simvastatin 20mg tablets (Zentiva)	No
67829	Pravastatin 20mg tablets (Sandoz Ltd)	No
67846	Atorvastatin 10mg tablets (Almus Pharmaceuticals Ltd)	No
67883	Lomitapide 5mg capsules	No
68023	Atorvastatin 10mg tablets (Aspire Pharma Ltd)	No
68048	Atorvastatin 20mg tablets (Phoenix Healthcare Distribution Ltd)	No
68156	Pravastatin 10mg tablets (A A H Pharmaceuticals Ltd)	No
68386	Colestyramine 4g oral powder sachets (J M McGill Ltd)	No
68467	Atorvastatin 20mg tablets (Kent Pharmaceuticals Ltd)	No
68563	Simvastatin 40mg tablets (Brown & Burk UK Ltd)	No
68686	Simvastatin 20mg tablets (Genesis Pharmaceuticals Ltd)	No
68785	Atorvastatin 10mg tablets (Mylan Ltd)	No
68827	Atorvastatin 20mg tablets (Mylan Ltd)	No
69093	Atorvastatin 80mg tablets (Wockhardt UK Ltd)	No
69413	Simvastatin 20mg tablets (Brown & Burk UK Ltd)	No

For peer review only

69427	Atorvastatin 40mg tablets (Mylan Ltd)	No

Supplemental table 2: Product codes for drugs used for hyperlipidaemia and fibrate exposure status.

For peer review only

Product Code	Product Name	PPARg Targeting?
23	Metformin 500mg tablets	No
32	Gliclazide 80mg tablets	No
93	Metformin 850mg tablets	No
240	Rifampicin 150mg capsules	No
469	Rosiglitazone 4mg tablets	Yes
479	Acarbose 50mg tablets	No
547	Glipizide 2.5mg tablets	No
548	Pioglitazone 15mg tablets	Yes
735	Metformin 100mg/ml Oral solution	No
1254	Glibenclamide 5mg tablets	No
1964	Diamicron 80mg tablets (Servier Laboratories Ltd)	No
1965	Tolbutamide 500mg tablets	No
2219	Glibenclamide 2.5mg tablets	No
4862	Diabetamide 2.5mg tablets (Ashbourne Pharmaceuticals Ltd)	No
5174	Acarbose 100mg tablets	No
5227	Rosiglitazone 8mg tablets	Yes
5276	Glimepiride 1mg tablets	No
5316	Glimepiride 4mg tablets	No
5353	Glimepiride 2mg tablets	No
5621	Glucobay 50mg tablets (Bayer Plc)	No
5627	Gliclazide 30mg modified-release tablets	No
5636	Glipizide 5mg tablets	No
5678	Nateglinide 120mg tablets	No
5989	Nateglinide 180mg tablets	No
6337	Glimepiride 3mg tablets	No
6855	Avandamet 2mg/500mg tablets (GlaxoSmithKline UK Ltd)	Yes
7048	Metformin 500mg modified-release tablets	No
7166	Glucophage 500mg tablets (Merck Serono Ltd)	No
7284	Amaryl 2mg tablets (Zentiva)	No
7325	Avandamet 4mg/1000mg tablets (GlaxoSmithKline UK Ltd)	Yes
7332	Amaryl 1mg tablets (Zentiva)	No
7375	Rosiglitazone 4mg / Metformin 1g tablets	Yes
7409	Amaryl 3mg tablets (Zentiva)	No
7610	Glucophage 850mg tablets (Merck Serono Ltd)	No
7744	Daonil 5mg tablets (Sanofi)	No
7818	Rifater tablets (Sanofi)	No
7912	Semi-Daonil 2.5mg tablets (Sanofi)	No
8976	Euglucon 2.5mg tablets (Aventis Pharma)	No
9105	Glucobay 100mg tablets (Bayer Plc)	No
9662	Avandia 4mg tablets (GlaxoSmithKline UK Ltd)	Yes
9691	Rifampicin with isoniazid & pyrazinamide tablet	No
9699	Pioglitazone 30mg tablets	Yes
9707	Repaglinide 1mg tablets	No
9748	Repaglinide 2mg tablets	No
9865	Repaglinide 500microgram tablets	No
10051	Pioglitazone 45mg tablets	Yes
11284	Amaryl 4mg tablets (Zentiva)	No
11316	NovoNorm 500microgram tablets (Novo Nordisk Ltd)	No
11321	NovoNorm 1mg tablets (Novo Nordisk Ltd)	No
11366	NovoNorm 2mg tablets (Novo Nordisk Ltd)	No
11483	Nateglinide 60mg tablets	No
11601	Rosiglitazone 2mg / Metformin 500mg tablets	Yes
11604	Rosiglitazone 1mg / Metformin 500mg tablets	Yes
11609	Metformin with rosiglitazone 500mg + 1mg Tablet	Yes
11610	Metformin with rosiglitazone 500mg + 2mg Tablet	Yes
11695	Diamicron 30mg MR tablets (Servier Laboratories Ltd)	No
11717	Rosiglitazone 2mg / Metformin 1g tablets	Yes
11737	Metformin with rosiglitazone 1000mg + 4mg Tablet	Yes
11760	Metformin with rosiglitazone 1000mg + 2mg Tablet	Yes
11946	Tolbutamide 50mg/ml Injection	No
11990	Metformin 500mg/5ml oral solution sugar free	No
12455	Rastinon 500mg Tablet (Hoechst Marion Roussel)	No
12513	Glibenese 5mg tablets (Pfizer Ltd)	No
13331	Euglucon 5mg tablets (Sanofi)	No

For peer review only

13628	Romozin 400mg Tablet (Glaxo Wellcome UK Ltd)	Yes	26218	Calabren 5mg Tablet (Berk Pharmaceuticals Ltd)	No
14164	Avandamet 2mg/1000mg tablets (GlaxoSmithKline UK Ltd)	Yes	26258	Glucamet 850mg Tablet (Opus Pharmaceuticals Ltd)	No
15232	Avandia 8mg tablets (GlaxoSmithKline UK Ltd)	Yes	27125	Starlix 180mg tablets (Novartis Pharmaceuticals UK Ltd)	No
15374	Gliclazide 40mg/5ml oral suspension	No	27501	Orabet 500mg Tablet (Lagap)	No
15955	Starlix 120mg tablets (Novartis Pharmaceuticals UK Ltd)	No	28708	Malix 2.5mg Tablet (Lagap)	No
16044	Glucophage SR 500mg tablets (Merck Serono Ltd)	No	29326	Glipizide 5mg tablets (Mylan Ltd)	No
16602	Calabren 2.5mg Tablet (Berk Pharmaceuticals Ltd)	No	29939	Gliclazide 80mg tablets (Mylan Ltd)	No
17343	Gliclazide 80mg tablets (A A H Pharmaceuticals Ltd)	No	30316	Metformin with pioglitazone 850mg + 15mg Tablet	Yes
17580	Avandamet 1mg/500mg tablets (GlaxoSmithKline UK Ltd)	Yes	30460	Malix 5mg Tablet (Lagap)	No
17698	Minodiab 5mg tablets (Pfizer Ltd)	No	31077	Competact 15mg/850mg tablets (Takeda UK Ltd)	Yes
17706	Minodiab 2.5mg tablets (Pfizer Ltd)	No	31146	Metsol 500mg/5ml oral solution (Kappin Ltd)	No
17770	Glucagon novo 10mg Injection (Novo Nordisk Ltd)	No	31212	Gliclazide 80mg tablets (Actavis UK Ltd)	No
18220	Pioglitazone 15mg / Metformin 850mg tablets	Yes	31474	Libanil 5mg Tablet (Approved Prescription Services Ltd)	No
19472	Actos 45mg tablets (Takeda UK Ltd)	Yes	33087	Metformin 500mg tablets (Actavis UK Ltd)	No
20287	Actos 15mg tablets (Takeda UK Ltd)	Yes	33562	Duclazide 80mg Tablet (Dumex Ltd)	No
20889	Actos 30mg tablets (Takeda UK Ltd)	Yes	33673	Tolbutamide 500mg tablets (Actavis UK Ltd)	No
21424	Glibenclamide 5mg/5ml oral suspension	No	33674	Metformin 850mg tablets (A A H Pharmaceuticals Ltd)	No
21564	Gliclazide 80mg tablets (Wockhardt UK Ltd)	No	34004	Metformin 500mg tablets (IVAX Pharmaceuticals UK Ltd)	No
21832	Diabetamide 5mg tablets (Ashbourne Pharmaceuticals Ltd)	No	34020	Metformin 850mg tablets (IVAX Pharmaceuticals UK Ltd)	No
21892	Diaglyk 80mg tablets (Ashbourne Pharmaceuticals Ltd)	No	34135	Metformin 500mg Tablet (M & A Pharmachem Ltd)	No
22239	Glucagon lilly 1mg Injection (Eli Lilly and Company Ltd)	No	34323	Metformin 500mg tablets (A A H Pharmaceuticals Ltd)	No
23945	Starlix 60mg tablets (Novartis Pharmaceuticals UK Ltd)	No	34399	Gliclazide 80mg tablets (IVAX Pharmaceuticals UK Ltd)	No
25636	Libanil 2.5mg Tablet (Approved Prescription Services Ltd)	No	34504	Metformin 500mg tablets (Wockhardt UK Ltd)	No
25678	Glucamet 500mg Tablet (Opus Pharmaceuticals Ltd)	No	34507	Glibenclamide 2.5mg tablets (Wockhardt UK Ltd)	No
			34563	Glibenclamide 5mg tablets (Wockhardt UK Ltd)	No
			34598	Metformin 500mg tablets (Mylan Ltd)	No

For peer review only

34676	Glibenclamide 2.5mg tablets (A A H Pharmaceuticals Ltd)	No
34697	Metformin 850mg tablets (Wockhardt UK Ltd)	No
34706	Glibenclamide 2.5mg tablets (IVAX Pharmaceuticals UK Ltd)	No
34742	Metformin 850mg tablets (Teva UK Ltd)	No
34802	Glipizide 5mg tablets (IVAX Pharmaceuticals UK Ltd)	No
34836	Metformin 850mg tablets (Actavis UK Ltd)	No
34917	Metformin 500mg tablets (Teva UK Ltd)	No
34932	Gliclazide 80mg tablets (Genus Pharmaceuticals Ltd)	No
34957	Tolbutamide 500mg tablets (A A H Pharmaceuticals Ltd)	No
35022	Sitagliptin 100mg tablets	No
35144	Byetta 5micrograms/0.02ml solution for injection 1.2ml pre-filled disposable devices (AstraZeneca UK Ltd)	No
35149	Exenatide 10micrograms/0.04ml solution for injection 2.4ml pre-filled disposable devices	No
35150	Byetta 10micrograms/0.04ml solution for injection 2.4ml pre-filled disposable devices (AstraZeneca UK Ltd)	No
35251	Exenatide 5micrograms/0.02ml solution for injection 1.2ml pre-filled disposable devices	No
35462	Januvia 100mg tablets (Merck Sharp & Dohme Ltd)	No
35561	Prandin 2mg tablets (Novo Nordisk Ltd)	No
36774	Prandin 1mg tablets (Novo Nordisk Ltd)	No
36856	Gliclazide 80mg tablets (Sandoz Ltd)	No
36948	Prandin 0.5mg tablets (Novo Nordisk Ltd)	No
37617	Rosiglitazone 2mg tablet	Yes
37874	Vildagliptin 50mg / Metformin 850mg tablets	No
37875	Vildagliptin 50mg tablets	No
37902	Vildagliptin 50mg / Metformin 1g tablets	No
38355	Metformin 750mg modified-release tablets	No
38400	Glucophage SR 750mg tablets (Merck Serono Ltd)	No
38551	Eucreas 50mg/1000mg tablets (Novartis Pharmaceuticals UK Ltd)	No
39149	Galvus 50mg tablets (Novartis Pharmaceuticals UK Ltd)	No
39203	Eucreas 50mg/850mg tablets (Novartis Pharmaceuticals UK Ltd)	No
39560	Bolamyn SR 500mg tablets (Teva UK Ltd)	No
39598	Metformin 1g modified-release tablets	No
39729	Glucophage SR 1000mg tablets (Merck Serono Ltd)	No
39988	Metformin 500mg oral powder sachets sugar free	No
40007	Glucophage 1000mg oral powder sachets (Merck Serono Ltd)	No
40110	Glucophage 500mg oral powder sachets (Merck Serono Ltd)	No
40233	Metformin 1g oral powder sachets sugar free	No
40365	Glimepiride 1mg tablets (Actavis UK Ltd)	No
40425	Nazdol MR 30mg tablets (Teva UK Ltd)	No
40642	Victoza 6mg/ml solution for injection 3ml pre-filled pen (Novo Nordisk Ltd)	No
40693	Liraglutide 6mg/ml solution for injection 3ml pre-filled disposable devices	No
41204	Saxagliptin 5mg tablets	No
41431	Onglyza 5mg tablets (AstraZeneca UK Ltd)	No
41558	Glibenclamide 5mg tablets (Teva UK Ltd)	No
41559	Glibenclamide 5mg tablets (A A H Pharmaceuticals Ltd)	No
41593	Glibenclamide 2.5mg tablets (Teva UK Ltd)	No
42161	Orabet 500mg Tablet (Sandoz Ltd)	No
42790	Gliclazide 80mg Tablet (Merck Generics (UK) Ltd)	No
43065	Gliclazide 40mg tablets	No

For peer review only

43270	Metformin 500mg/5ml oral solution sugar free (Rosemont Pharmaceuticals Ltd)	No
43465	Zicron 40mg tablets (Bristol Laboratories Ltd)	No
43619	Metformin 1g / Sitagliptin 50mg tablets	No
43684	Janumet 50mg/1000mg tablets (Merck Sharp & Dohme Ltd)	No
44250	Metformin 500mg/5ml Oral solution (Hillcross Pharmaceuticals Ltd)	No
44304	Glyconon 500mg Tablet (DDSA Pharmaceuticals Ltd)	No
44473	Edicil MR 30mg tablets (Teva UK Ltd)	No
44738	Niddaryl 1mg tablets (Dee Pharmaceuticals Ltd)	No
45215	Gliclazide 80mg Tablet (Neo Laboratories Ltd)	No
45581	Metabet SR 500mg tablets (Morningside Healthcare Ltd)	No
45775	Saxagliptin 2.5mg tablets	No
45821	Onglyza 2.5mg tablets (AstraZeneca UK Ltd)	No
45831	Dacadis MR 30mg tablets (Mylan Ltd)	No
46458	Exenatide 2mg powder and solvent for prolonged-release suspension for injection vials	No
46469	Bydureon 2mg powder and solvent for prolonged-release suspension for injection vials (AstraZeneca UK Ltd)	No
46665	Linagliptin 5mg tablets	No
46716	Trajenta 5mg tablets (Boehringer Ingelheim Ltd)	No
46927	Tolbutamide 500mg tablets (Teva UK Ltd)	No
46989	Metabet SR 1000mg tablets (Morningside Healthcare Ltd)	No
47074	Gliclazide 80mg/5ml oral suspension	No
47894	Nazdol MR 30mg tablets (Consilient Health Ltd)	No
47939	Glucient SR 500mg tablets (Consilient Health Ltd)	No
48056	Gliclazide 80mg tablets (Sovereign Medical Ltd)	No
48139	Pioglitazone 30mg tablets (A A H Pharmaceuticals Ltd)	Yes
48149	Metformin 500mg tablets (Almus Pharmaceuticals Ltd)	No
48401	Sitagliptin 50mg tablets	No
48533	Sitagliptin 25mg tablets	No
49502	Glucophage SR 500mg tablets (Mawdsley-Brooks & Company Ltd)	No
49738	Metformin 1g modified-release tablets (A A H Pharmaceuticals Ltd)	No
50087	Januvia 50mg tablets (Merck Sharp & Dohme Ltd)	No
50124	Januvia 25mg tablets (Merck Sharp & Dohme Ltd)	No
50570	Glucophage SR 500mg tablets (Lexon (UK) Ltd)	No
50821	Metformin 850mg tablets (Pfizer Ltd)	No
50970	Metformin 500mg tablets (Bristol Laboratories Ltd)	No
51080	Metabet SR 1000mg tablets (Actavis UK Ltd)	No
51135	Metformin 500mg modified-release tablets (A A H Pharmaceuticals Ltd)	No
51527	Metformin 500mg tablets (Boston Healthcare Ltd)	No
51955	Gliclazide 80mg tablets (Accord Healthcare Ltd)	No
52203	Enyglid 0.5mg tablets (Consilient Health Ltd)	No
52221	Diagemet XL 500mg tablets (Genus Pharmaceuticals Ltd)	No
52442	Metformin 500mg tablets (Pfizer Ltd)	No
52634	Glucophage SR 500mg tablets (DE Pharmaceuticals)	No
53288	Gliclazide 30mg modified-release tablets (A A H Pharmaceuticals Ltd)	No
53478	Metformin 500mg modified-release tablets (Kent Pharmaceuticals Ltd)	No
53774	Metabet SR 500mg tablets (Actavis UK Ltd)	No
53867	Metformin 500mg tablets (Zentiva)	No
54150	Jentadueto 2.5mg/850mg tablets (Boehringer Ingelheim Ltd)	No
54182	Dapagliflozin 10mg tablets	No

For peer review only

54203	Forxiga 10mg tablets (AstraZeneca UK Ltd)	No
54265	Dapagliflozin 5mg tablets	No
54442	Metformin (roi) 1000mg Tablet	No
54480	Forxiga 5mg tablets (AstraZeneca UK Ltd)	No
54764	Gliclazide 80mg tablets (Arrow Generics Ltd)	No
54891	Saxagliptin 2.5mg / Metformin 1g tablets	No
54898	Metformin 850mg tablets (Almus Pharmaceuticals Ltd)	No
55270	Duformin 500mg Tablet (Dumex Ltd)	No
55711	Metformin 500mg tablets (Alliance Healthcare (Distribution) Ltd)	No
55739	Metformin 500mg tablets (Tillomed Laboratories Ltd)	No
55862	Gliclazide Oral solution	No
56008	Gliclazide 80mg tablets (Almus Pharmaceuticals Ltd)	No
56208	Pioglitazone 15mg tablets (A A H Pharmaceuticals Ltd)	Yes
56376	Rosiglitazone 4mg with glimepiride 4mg tablet	No
56437	Gliclazide 60mg modified-release tablets	No
56965	Komboglyze 2.5mg/1000mg tablets (AstraZeneca UK Ltd)	No
57147	Bolamyn SR 1000mg tablets (Teva UK Ltd)	No
57457	Metformin 500mg tablets (Aurobindo Pharma Ltd)	No
57601	Daonil 5mg tablets (Dowelhurst Ltd)	No
57659	Pioglitazone 30mg tablets (Actavis UK Ltd)	Yes
57830	Gliclazide 30mg modified-release tablets (Alliance Healthcare (Distribution) Ltd)	No
58051	Metformin 500mg/5ml oral solution	No
58607	Metformin 500mg/5ml oral solution sugar free (Zentiva)	No
59620	Glucophage SR 500mg tablets (Waymade Healthcare Plc)	No
59809	Alogliptin 6.25mg tablets	No
60012	Dapagliflozin 5mg / Metformin 1g tablets	No
60074	Metformin 1g modified-release tablets (Waymade Healthcare Plc)	No
60211	Canagliflozin 100mg tablets	No
60286	Metformin 500mg/5ml oral suspension	No
60430	Invokana 100mg tablets (Janssen-Cilag Ltd)	No
60495	Gliclazide 80mg tablets (Teva UK Ltd)	No
60643	Xigduo 5mg/1000mg tablets (AstraZeneca UK Ltd)	No
60968	Metformin 500mg modified-release tablets (Actavis UK Ltd)	No
61043	Sukkarto SR 1000mg tablets (Morningside Healthcare Ltd)	No
61311	Glimepiride 4mg tablets (Sigma Pharmaceuticals Plc)	No
61559	Sukkarto SR 500mg tablets (Morningside Healthcare Ltd)	No
61925	NovoNorm 500microgram tablets (Waymade Healthcare Plc)	No
61957	Gliclazide 40mg tablets (A A H Pharmaceuticals Ltd)	No
62014	Glimepiride 2mg tablets (Accord Healthcare Ltd)	No
62034	Laaglyda MR 60mg tablets (Consilient Health Ltd)	No
62144	Metformin 500mg modified-release tablets (DE Pharmaceuticals)	No
62265	Metformin 500mg modified-release tablets (Mawdsley-Brooks & Company Ltd)	No
62326	Vipidia 6.25mg tablets (Takeda UK Ltd)	No
62426	Pioglitazone 30mg tablets (Accord Healthcare Ltd)	Yes
62426	Pioglitazone 30mg tablets (Accord Healthcare Ltd)	No
62605	Metformin 850mg tablets (Kent Pharmaceuticals Ltd)	No
62661	Bydureon 2mg powder and solvent for prolonged-release suspension for injection pre-filled pen (AstraZeneca UK Ltd)	No
62824	Metformin 1g modified-release tablets (Actavis UK Ltd)	No
63045	Metformin 850mg tablets (Relonchem Ltd)	No

For peer review only

63046	Pioglitazone 45mg tablets (A A H Pharmaceuticals Ltd)	Yes
63048	Gliclazide 80mg tablets (Alliance Healthcare (Distribution) Ltd)	No
63107	Pioglitazone 45mg tablets (Waymade Healthcare Plc)	Yes
63131	Ziclaseg 30mg modified-release tablets (Lupin (Europe) Ltd)	No
63336	Trulicity 1.5mg/0.5ml solution for injection pre-filled pen (Eli Lilly and Company Ltd)	No
63401	Trulicity 0.75mg/0.5ml solution for injection pre-filled pen (Eli Lilly and Company Ltd)	No
63421	Pioglitazone 30mg tablets (Teva UK Ltd)	Yes
63516	Forxiga 10mg tablets (Waymade Healthcare Plc)	No
63785	Dulaglutide 0.75mg/0.5ml solution for injection pre-filled disposable devices	No
63823	Dulaglutide 1.5mg/0.5ml solution for injection pre-filled disposable devices	No
64217	Jardiance 25mg tablets (Boehringer Ingelheim Ltd)	No
64900	Glidipion 30mg tablets (Actavis UK Ltd)	Yes
64939	Glucient SR 1000mg tablets (Consilient Health Ltd)	No
65083	Synjardy 5mg/1000mg tablets (Boehringer Ingelheim Ltd)	No
65344	Empagliflozin 5mg / Metformin 850mg tablets	No
65562	Pioglitazone 30mg tablets (Alliance Healthcare (Distribution) Ltd)	Yes
65563	Pioglitazone 15mg tablets (Alliance Healthcare (Distribution) Ltd)	Yes
65694	Metformin 500mg modified-release tablets (Waymade Healthcare Plc)	No
65923	Metformin 1g modified-release tablets (Mawdsley-Brooks & Company Ltd)	No
66008	Synjardy 12.5mg/1000mg tablets (Boehringer Ingelheim Ltd)	No
66136	Glucophage SR 1000mg tablets (Waymade Healthcare Plc)	No
66399	Glimepiride 2mg tablets (A A H Pharmaceuticals Ltd)	No
67056	Amaryl 1mg tablets (Lexon (UK) Ltd)	No
67781	Gliclazide 80mg tablets (Milpharm Ltd)	No
68203	Metformin 500mg modified-release tablets (Almus Pharmaceuticals Ltd)	No
68214	Metformin 500mg/5ml oral solution sugar free (A A H Pharmaceuticals Ltd)	No
68289	Glimepiride 4mg tablets (Waymade Healthcare Plc)	No
68389	Metformin 500mg/5ml oral solution sugar free (Pinewood Healthcare)	No
68415	Gliclazide 30mg modified-release tablets (Phoenix Healthcare Distribution Ltd)	No
68636	Metformin 850mg/5ml oral solution sugar free	No
68675	Glimepiride 4mg tablets (Somex Pharma)	No
68819	Gliclazide 80mg tablets (Bristol Laboratories Ltd)	No

Supplemental table 3: Product codes for drugs used for diabetes and glitazone status.

For peer review only

Glitazones Case-Control				
	**N = 480**	**N = 1920**		
**Glitazone exposure status**				
**Per-year glitazone exposure**				
**Unexposed**	383 (79.8%)	1460 (76.0%)	Referent	-
**≤ 1 year**	41 (8.5%)	151 (7.9%)	1.11 (0.77, 1.61)	0.59
**1 < years ≤ 2**	23 (4.8%)	89 (4.6%)	1.06 (0.66, 1.71)	0.82
**2 < years ≤ 3**	29 (4.0%)	66 (3.4%)	1.13 (0.67, 1.92)	0.65
**3 < years ≤ 4**	8 (1.7%)	61 (3.2%)	0.52 (0.25, 1.10)	0.07
**4 < years ≤ 5**	2 (0.4%)	41 (2.1%)	0.18 (0.04, 0.77)	0.003
**5 < years ≤ 6**	2 (0.4%)	23 (1.2%)	0.30 (0.07, 1.30)	0.06
**> 6 years**	2 (0.4%)	29 (1.5%)	0.44 (0.10, 1.94)	0.23
**"First drug exposure" status**				
**Glitazone**	4 (0.8%)	13 (0.7%)	1.10 (0.35, 3.44)	0.87
**Other anti-type 2 diabetes drug**	476 (99.2%)	1907 (99.3%)	Referent	-

* Model adjusted for sex, age, IMD, retrospective prescription history (categorised yearly) and mean HbA1c levels. ** OR calculated for per year increase in variable.

Supplemental table 4: Case-control results for brain tumour cases with glitazone exposure in years duration groups and as first drug exposure.

For peer review only

Variable	Cases (%)	Controls (%)	Adjusted OR (95% CI) *	P-value
**Imputed HbA1c levels**				
	**N = 480**	**N = 1920**		
**Glitazone exposure status**				
**Exposed**	97 (20.2%)	460 (24.0%)	0.81 (0.66, 1.00)	0.11
**Unexposed**	383 (79.8%)	1460 (76.0%)	Referent	-
**Mean glitazone exposure duration (longest), days (SD)**	607 (583)	872 (760)	0.87 (0.82, 0.93) **	0.001
**Mean glitazone exposure duration (total), days (SD)**	604 (720)	1002 (823)	0.85 (0.79, 0.91) **	0.00005
**No HbA1c adjustment**				
	**N = 480**	**N = 1920**		
**Glitazone exposure status**				
**Exposed**	97 (20.2%)	460 (24.0%)	0.79 (0.62, 1.02)	0.07
**Unexposed**	383 (79.8%)	1460 (76.0%)	Referent	-
**Mean glitazone exposure duration (longest), days (SD)**	607 (583)	872 (760)	0.87 (0.80, 0.94)	0.0008
**Mean glitazone exposure duration (total), days (SD)**	604 (720)	1002 (823)	0.85 (0.78, 0.92)	0.00004

*** Adjusted for sex, age, IMD and retrospective prescription history. HbA1c adjustment differed according to the analysis.**
**** OR calculated for per year increase in variable.**

Supplemental table 5: Case-control results for brain tumour cases with glitazone exposure with HbA1C imputed or not adjusted in the multivariable models.

For peer review only

**Glitazones - 6 months latency period**				
	**N = 480**	**N = 1920**		
**Glitazone exposure status**	**Cases (%)**	**Controls (%)**	**Adjusted OR (95% CI) ***	**P-value**
**Exposed**	95 (19.8%)	460 (24.0%)	0.82 (0.64, 1.05)	0.12
**Unexposed**	385 (80.2%)	1460 (76.0%)	Referent	-
**Mean glitazone exposure duration (longest), days (SD)**	574 (581)	770 (651)	0.88 (0.81, 0.96) **	0.002
**Mean glitazone exposure duration (total), days (SD)**	771 (693)	988 (796)	0.89 (0.83, 0.96) **	0.0015
**Glitazones - 12 months latency period**				
	**N = 480**	**N = 1920**		
**Glitazone exposure status**				
**Exposed**	92 (19.2%)	457 (23.8%)	0.79 (0.61, 1.02)	0.07
**Unexposed**	388 (80.8%)	1463 (76.2%)	Referent	-
**Mean glitazone exposure duration (longest), days (SD)**	540 (572)	753 (642)	0.87 (0.79, 0.95) **	0.002
**Mean glitazone exposure duration (total), days (SD)**	670 (690)	958 (774)	0.87 (0.80, 0.94) **	0.0003
**Glitazones - 24 months latency period**				
	**N = 480**	**N = 1920**		
**Glitazone exposure status**				
**Exposed**	86 (17.9%)	449 (23.4%)	0.74 (0.57, 0.96)	0.02
**Unexposed**	394 (82.1%)	1471 (76.6%)	Referent	-
**Mean glitazone exposure duration (longest), days (SD)**	478 (557)	720 (625)	0.83 (0.75, 0.92) **	0.0004
**Mean glitazone exposure duration (total), days (SD)**	593 (672)	901 (730)	0.84 (0.76, 0.91) **	0.000007

Supplemental table 6: Case-control results for brain tumour cases with glitazone exposure for latency periods of 6, 12, and 24 months.

For peer review only

STROBE 2007 (v4) checklist of items to be included in reports of observational studies in epidemiology*
Checklist for cohort, case-control, and cross-sectional studies (combined)

Section/Topic	Item #	Recommendation	Reported on page #
**Title and abstract**	1	(*a*) Indicate the study's design with a commonly used term in the title or the abstract	1
		(*b*) Provide in the abstract an informative and balanced summary of what was done and what was found	2
**Introduction**			
Background/rationale	2	Explain the scientific background and rationale for the investigation being reported	5
Objectives	3	State specific objectives, including any pre-specified hypotheses	5
**Methods**			
Study design	4	Present key elements of study design early in the paper	6
Setting	5	Describe the setting, locations, and relevant dates, including periods of recruitment, exposure, follow-up, and data collection	6
Participants	6	(*a*) *Cohort study*—Give the eligibility criteria, and the sources and methods of selection of participants. Describe methods of follow-up<br>*Case-control study*—Give the eligibility criteria, and the sources and methods of case ascertainment and control selection. Give the rationale for the choice of cases and controls<br>*Cross-sectional study*—Give the eligibility criteria, and the sources and methods of selection of participants	6-7
		(*b*) *Cohort study*—For matched studies, give matching criteria and number of exposed and unexposed<br>*Case-control study*—For matched studies, give matching criteria and the number of controls per case	6-7
Variables	7	Clearly define all outcomes, exposures, predictors, potential confounders, and effect modifiers. Give diagnostic criteria, if applicable	6-8
Data sources/ measurement	8*	For each variable of interest, give sources of data and details of methods of assessment (measurement). Describe comparability of assessment methods if there is more than one group	6-8
Bias	9	Describe any efforts to address potential sources of bias	6-7
Study size	10	Explain how the study size was arrived at	6
Quantitative variables	11	Explain how quantitative variables were handled in the analyses. If applicable, describe which groupings were chosen and why	8
Statistical methods	12	(*a*) Describe all statistical methods, including those used to control for confounding	9-10
		(*b*) Describe any methods used to examine subgroups and interactions	9-10
		(*c*) Explain how missing data were addressed	9-10
		(*d*) *Cohort study*—If applicable, explain how loss to follow-up was addressed<br>*Case-control study*—If applicable, explain how matching of cases and controls was addressed	

For peer review only

		Cross-sectional study—If applicable, describe analytical methods taking account of sampling strategy	
		(*e*) Describe any sensitivity analyses	9-10
**Results**			
Participants	13*	(a) Report numbers of individuals at each stage of study—eg numbers potentially eligible, examined for eligibility, confirmed eligible, included in the study, completing follow-up, and analysed	10
		(b) Give reasons for non-participation at each stage	N/A
		(c) Consider use of a flow diagram	N/A
Descriptive data	14*	(a) Give characteristics of study participants (eg demographic, clinical, social) and information on exposures and potential confounders	10 & 18
		(b) Indicate number of participants with missing data for each variable of interest	11
		(c) *Cohort study*—Summarise follow-up time (eg, average and total amount)	
Outcome data	15*	*Cohort study*—Report numbers of outcome events or summary measures over time	
		Case-control study—Report numbers in each exposure category, or summary measures of exposure	18
		Cross-sectional study—Report numbers of outcome events or summary measures	
Main results	16	(*a*) Give unadjusted estimates and, if applicable, confounder-adjusted estimates and their precision (eg, 95% confidence interval). Make clear which confounders were adjusted for and why they were included	19-20
		(*b*) Report category boundaries when continuous variables were categorized	18-20
		(*c*) If relevant, consider translating estimates of relative risk into absolute risk for a meaningful time period	
Other analyses	17	Report other analyses done—eg analyses of subgroups and interactions, and sensitivity analyses	11-12
**Discussion**			
Key results	18	Summarise key results with reference to study objectives	12
Limitations	19	Discuss limitations of the study, taking into account sources of potential bias or imprecision. Discuss both direction and magnitude of any potential bias	13
Interpretation	20	Give a cautious overall interpretation of results considering objectives, limitations, multiplicity of analyses, results from similar studies, and other relevant evidence	14
Generalisability	21	Discuss the generalisability (external validity) of the study results	14
**Other information**			
Funding	22	Give the source of funding and the role of the funders for the present study and, if applicable, for the original study on which the present article is based	3-4

*Give information separately for cases and controls in case-control studies and, if applicable, for exposed and unexposed groups in cohort and cross-sectional studies.

Note: An Explanation and Elaboration article discusses each checklist item and gives methodological background and published examples of transparent reporting. The STROBE checklist is best used in conjunction with this article (freely available on the Web sites of PLoS Medicine at http://www.plosmedicine.org/, Annals of Internal Medicine at http://www.annals.org/, and Epidemiology at http://www.epidem.com/). Information on the STROBE Initiative is available at www.strobe-statement.org.

For peer review only
